# α‐Functionalisation of Cyclic Sulfides Enabled by Lithiation Trapping

**DOI:** 10.1002/anie.202314423

**Published:** 2023-12-07

**Authors:** Nico Seling, Masakazu Atobe, Kevin Kasten, James D. Firth, Peter B. Karadakov, Frederick W. Goldberg, Peter O'Brien

**Affiliations:** ^1^ Department of Chemistry University of York York YO10 5DD UK; ^2^ Modulus Discovery, Inc. Daiichi Hibiya Building 7th Floor1-18-21 Shimbashi Minato-ku Tokyo 105-0004 Japan; ^3^ Oncology R&D AstraZeneca 1 Francis Crick Ave Cambridge CB2 0AA UK

**Keywords:** Cyclic Sulfides, Organolithium, α-Functionalization

## Abstract

A general and straightforward procedure for the lithiation trapping of cyclic sulfides such as tetrahydrothiophene, tetrahydrothiopyran and a thiomorpholine is described. Trapping with a wide range of electrophiles is demonstrated, leading to more than 50 diverse α‐substituted saturated sulfur heterocycles. The methodology provides access to a range of α‐substituted cyclic sulfides that are not easily synthesised by the currently available methods.

## Graphical abstract

Lithiation and trapping at convenient temperatures (0/−10 °C) of five cyclic sulfides such as tetrahydrothiophene, tetrahydrothiopyrans and a thiomorpholine enables straightforward α‐functionalisation. Trapping with a wide range of electrophiles generates more than 50 diverse α‐substituted saturated sulfur heterocycles that are not easily synthesised by the currently available methods. TMEDA=*N,N,N′,N′*‐tetramethylethylenediamine

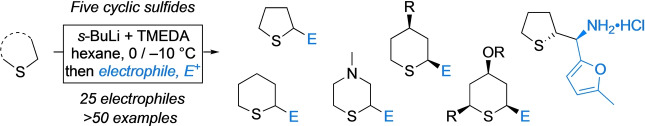

α‐Substituted five‐ and six‐membered ring saturated sulfur heterocycles such as tetrahydrothiophene, tetrahydrothiopyran and thiomorpholine feature in natural products, chiral catalysts and potential pharmaceuticals (Figure 1). Famous natural products include Fleming's original antibiotic penicillin G^[1]^ and biotin (vitamin B_7_)^[2]^ which is widely used for protein biotinylation in biochemical assays. Enantiopure five‐membered ring sulfide **1** and related sulfides have been used in a range of asymmetric reactions.[^3^, ^4^, ^5^] Furthermore, tetrahydrothiophene derivative **2** is a potent CXCR (Chemokine receptor) antagonist in inflammation models and is being developed to treat acne;^[6]^ disubstituted thiomorpholine **3** is an orexin antagonist and has been explored for use in the treatment of neurological disorders.^[7]^

**Figure 1:**
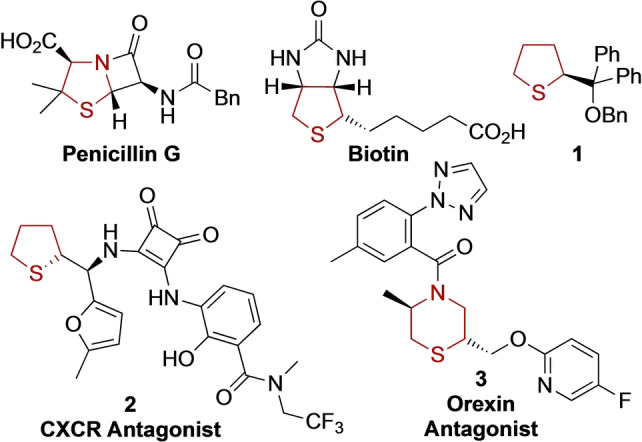
Natural products, chiral catalysts and potential pharmaceuticals containing α‐substituted saturated sulfur heterocycles. CXCR=Chemokine receptor.

The saturated sulfur heterocycles in **1**, **2** and **3** were all crafted by cyclisation of a pre‐functionalised substrate with the early introduction of the substituent α to sulfur. This is one of the most common routes to α‐substituted sulfur heterocycles. In contrast, approaches where an α‐substituent is directly appended onto an intact saturated sulfur heterocycle, with the potential for synthetically versatile later‐stage functionalisation, is a much less‐represented approach. Such methods include the generation of radical intermediates and subsequent addition to alkenes^[8]^ or alkynes,^[9]^ or cross‐dehydrogenative coupling (CDC),^[10]^ including photocatalysis^[11]^ (Scheme 1A).

**Scheme 1:**
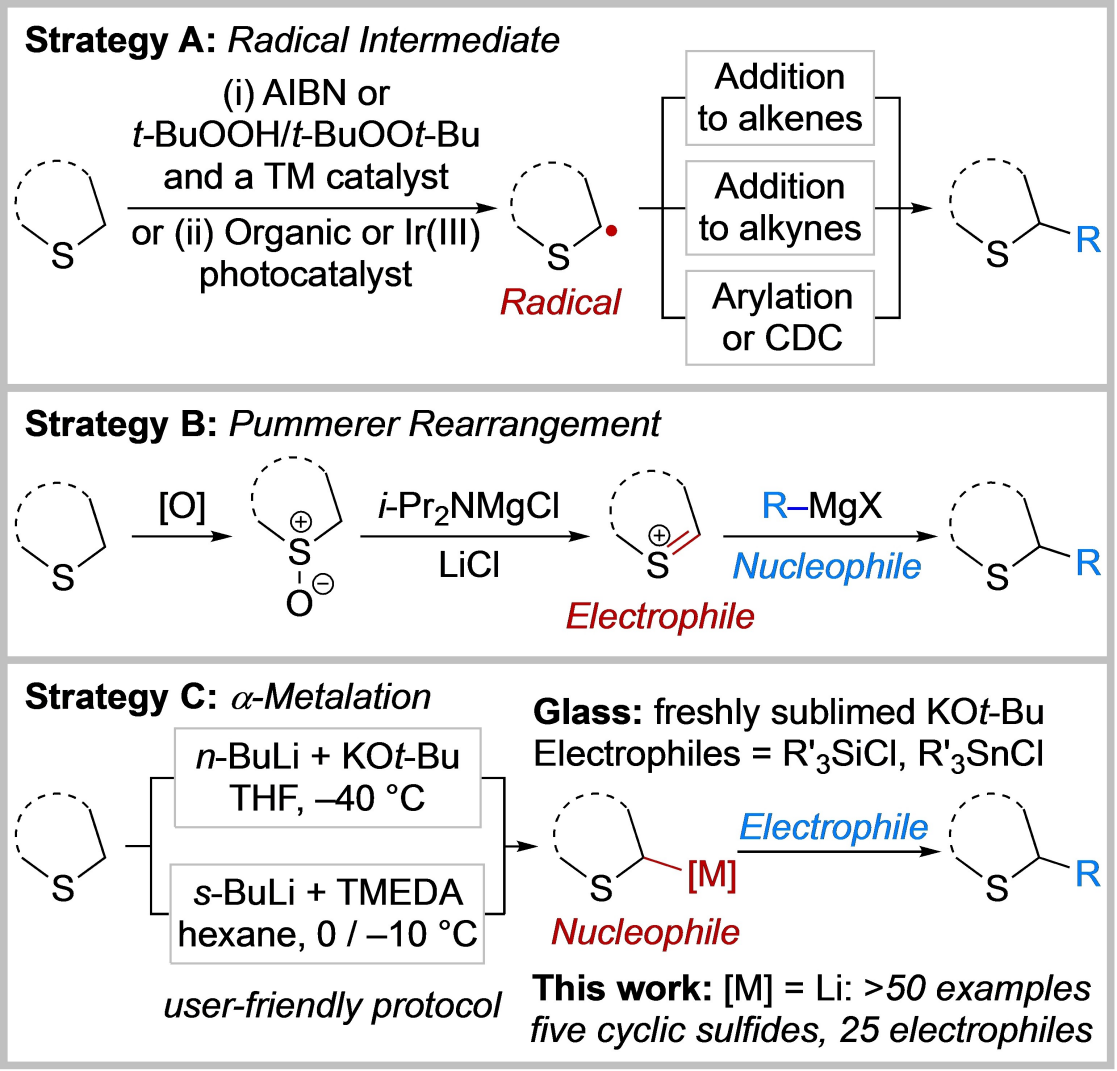
Strategies for the synthesis of α‐substituted sulfur heterocycles from the corresponding cyclic sulfides. Strategy A: Radical intermediate. Strategy B: Pummerer Rearrangement. Strategy C: α‐metalation. Azobisisobutyronitrile (AIBN); transition metal (TM); cross‐dehydrogenative coupling (CDC); *N,N,N′,N′*‐tetramethylethylene‐diamine (TMEDA).

Alternatively, α‐substituted thioethers can be accessed via oxidation of the cyclic sulfide to a sulfoxide and subsequent Pummerer type rearrangement using turbo‐Hauser bases and Grignard reagents as nucleophiles (Scheme 1B).[^12^, ^13^] Finally, Schlosser's base‐mediated direct α‐metalation/trapping of cyclic sulfides was reported by Liu and Glass (Scheme 1C).^[14]^ This method required use of freshly sublimed potassium *tert*‐butoxide, had a limited electrophile scope and was carried out at −40 °C due to the instability of the metalated tetrahydrothiophene (see below). In two different metalation routes, Mulvey et al. prepared and characterised (X‐ray crystallography/NMR spectroscopy) magnesiated^[15]^ and aluminated tetrahydrothiophene^[16]^ although electrophilic trapping proved challenging. Such metalation approaches^[17]^ build on the pioneering work by Gilman, Wittig, Corey, Seebach and Peterson in the 1940–60s on the α‐lithation of dimethylsulfide and thioanisole.^[18]^ Allylic and cyclopropyl‐containing acyclic sulfides have also been successfully lithiated and used in synthesis.^[19]^ However, it is notable that the metalation of cyclic sulfides is limited to those described by Liu and Glass^[14]^ and Mulvey et al.[^15^, ^16^] Building on our experience with the α‐lithiation/trapping of *N*‐Boc heterocycles,[^20^, ^21^] a general and experimentally simple lithiation/trapping protocol at near‐ambient temperatures (0/−10 °C) is now reported (Scheme 1C). We present more than 50 examples across five different cyclic sulfides and 25 electrophiles, including application to the core scaffold present in CXCR antagonist **2**.

To start, the lithiation/trapping of tetrahydrothiophene **4** was explored using 1.3 eq. *s*‐BuLi/TMEDA (*N,N,N′,N′*‐tetramethylethylenediamine) in hexane at the operationally simple temperature of 0 °C for 1 h. Subsequent trapping with PhMe_2_SiCl delivered α‐silyl tetrahydrothiophene **7 a** in 86 % yield (Scheme 2A, entry 1, optimised conditions). Hexane was selected as the reaction solvent due to its inertness towards lithiation compared to typical ethereal solvents (e.g. THF) and toluene, given that relatively high temperatures were employed. Reducing the reaction time to 20 min resulted in a lower conversion to **7 a** (66 %, entry 2) and use of *n*‐BuLi/TMEDA (0 °C, 1 h) was unsuccessful (entry 3), presumably as a result of its lower basicity. The high yield of **7 a** at 0 °C indicates that lithiated tetrahydrothiophene has good stability at this temperature in hexane over 1 h (entry 1). Conversely, Glass showed that use of Schlosser's base in THF at 5 °C for 1 h resulted in complete decomposition of the metalated tetrahydrothiophene by a retro‐[3+2] ring fragmentation^[14]^ which may be a result of the formation of an unstable *potassiated* tetrahydrothiophene.

**Scheme 2:**
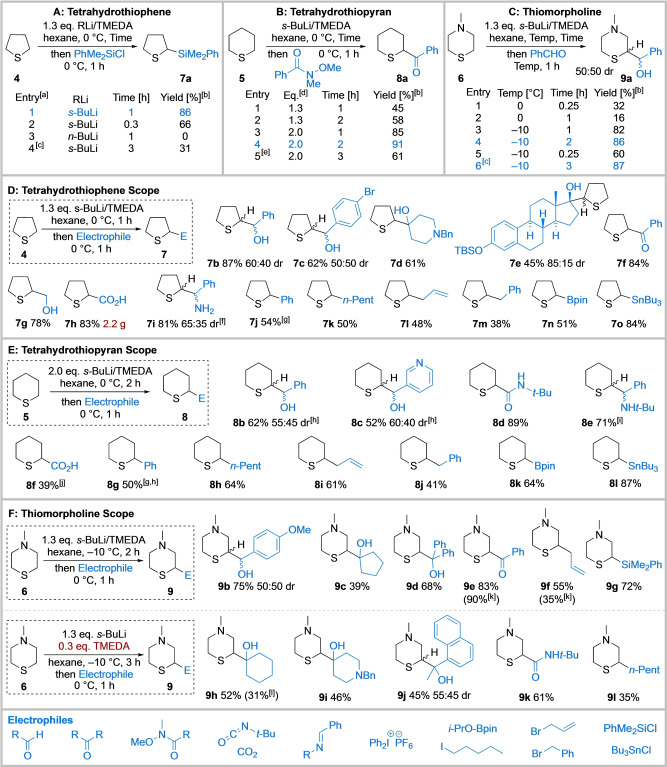
Functionalisation of tetrahydrothiophene **4**, tetrahydrothiopyran **5** and *N*‐methyl morpholine **6**. A: Tetrahydrothiophene. B: Tetrahydrothiopyran. C: Thiomorpholine. D: Tetrahydrothiophene Scope. E: Tetrahydrothiopyran Scope. F: Thiomorpholine Scope*. N,N,N′,N′*‐tetramethylethylene‐diamine (TMEDA); benzyl (Bn); *tert*‐butyldimethylsilyl (TBS); pinacolate (pin). ^[a]^ PhCHO as electrophile for entries 3 and 4. ^[b]^ Yield after chromatography. ^[c]^ 0.3 eq. TMEDA used. ^[d]^ Eq. of *s*‐BuLi/TMEDA. ^[e]^ Reaction carried out at −10 °C. ^[f]^ % yield of **7 i** ⋅ HCl over 2 steps; corresponding Ellman's *t‐*butyl sulfinamide used followed by deprotection with HCl_(aq)_. ^[g]^ Transmetalation to Cu using CuCN ⋅ 2LiCl prior to addition of Ph_2_IPF_6_. ^[h]^ 1.3 eq. *s*‐BuLi/TMEDA, hexane, 0 °C, 2 h then Electrophile, 0 °C, 1 h. ^[i]^ Single unidentified diastereomer. ^[j]^ 2.5 eq. of **5**. ^[k]^ Lithiation conditions: 1.3 eq. *s*‐BuLi, 0.3 eq. TMEDA, hexane, −10 °C, 3 h. ^[l]^ Lithiation conditions: 1.3 eq. *s*‐BuLi, 1.3 eq. TMEDA, hexane, −10 °C, 2 h.

Optimisation of the lithiation/trapping of tetrahydrothiopyran **5** (Scheme 2B) commenced using the optimal conditions for lithiating tetrahydrothiophene **4**, namely 1.3 eq. *s*‐BuLi/TMEDA in hexane at 0 °C for 1 h. Trapping with a phenyl Weinreb amide gave α‐keto tetrahydrothiopyran **8 a** in 45 % yield (entry 1). Increasing the lithiation time to 2 h gave a slight improvement (58 % of **8 a**, entry 2). These results suggested that lithiation was likely incomplete at 0 °C for 2 h; increasing the amount of *s*‐BuLi/TMEDA to 2.0 eq. gave **8 a** in much improved yields of 85 % and 91 % at 1 h and 2 h lithiation times respectively (entries 3 and 4). Lithiation at 0 °C for 2 h represents the optimised conditions (entry 4).

Finally, we investigated the lithiation/trapping of *N*‐methyl thiomorpholine **6** (Scheme 2C). Using 1.3 eq. *s*‐BuLi/TMEDA in hexane at 0 °C for 1 h, and trapping with benzaldehyde, gave a 50 : 50 mixture of diastereomeric α‐hydroxy thiomorpholines **9 a** in only 16 % yield (entry 2). The lithiated species appeared to be unstable; a 32 % yield of **9 a** was obtained after lithiation at 0 °C for a shorter lithiation time (15 min) (entry 1). Thus, we speculated that lithiation at a lower temperature may increase the stability of the lithiated intermediate. Indeed, reducing the lithiation temperature to −10 °C (1 h) significantly improved the yield of **9 a** to 82 % (entry 3). Although **9 a** was formed as a 50 : 50 mixture of diastereomers, we were successful in growing a crystal of only one diastereomer, *syn*‐**9 a**, for X‐ray crystallographic analysis^[22]^ (see Supporting Information). This revealed that the lithiation was completely regioselective—there was no lithiation α to nitrogen. Increasing the lithiation time to 2 h gave optimal conditions with **9 a** isolated in 86 % yield (entry 4), whereas **9 a** was obtained in only 60 % yield with a lithiation time of 15 min, (entry 5). A comparison between lithiation of tetrahydrothiopyran **5** and *N*‐methyl thiomorpholine **6** at −10 °C revealed a faster rate of lithiation of thiomorpholine **6** based on the yields of trapped products **8 a** (61 %, Scheme 2B, entry 5) and **9 a** (86 %, Scheme 2C, entry 4) respectively. A similar activating effect of an amino group can be identified from a comparison of the relative rates of lithiation of *N*‐Boc piperidine and *N*‐Boc piperazines.[^21b^, ^21f^] Of note, use of a substoichiometric amount (0.3 eq.) of TMEDA with a longer lithiation time of 3 h gave **9 a** in 87 % yield (entry 6). The successful use of substoichiometric TMEDA is remarkable as the diamine ligand (TMEDA in this case) usually remains coordinated to the lithiated species and is unavailable for further lithiation processes.^[23]^ We propose that lithiated *N*‐methyl thiomorpholine can dimerise (or oligomerise) to generate a stable higher order aggregate which frees up TMEDA and this is then available to coordinate to more *s*‐BuLi for further lithiation events. Alternatively, the amine in the thiomorpholine may also act as a ligand to aid deaggregation. Such effects appear to be specific to thiomorpholine **6** as the same effect was not observed with tetrahydrothiophene **4**: use of 0.3 eq. of TMEDA gave the trapped adduct in 31 % yield showing that TMEDA was not turned over in this reaction (Scheme 2A, entry 4).

With optimised reactions in hand, the electrophile scope of the lithiation/trapping of **4**–**6** was investigated. Trapping of lithiated tetrahydrothiophene **4** with a range of electrophiles worked well. Electrophiles with a carbonyl group were well tolerated with benzaldehydes giving alcohols **7 b** (87 %, 60 : 40 dr) and **7 c** (62 %, 50 : 50 dr). *N*‐Benzyl piperidinone gave tertiary alcohol **7 d** in 61 % yield, and silyl protected estrone gave **7 e** (85 : 15 dr, unidentified relative stereochemistry α to sulfur).^[24]^ Primary alcohol **7 g** (78 % yield) was obtained using paraformaldehyde. Use of phenyl Weinreb amide gave ketone **7 f** in 84 % yield. When trapping with CO_2_, 2.2 g (83 % yield) of acid **7 h** was isolated. In this case, trapping with CO_2_ was initially carried out at −78 °C to mitigate any potential exotherms. Trapping with a sulfinamide, followed by acidic hydrolysis, gave amine **7 i** in 81 % yield (65 : 35 dr). Next, α‐aryl tetrahydrothiophene **7 j** was obtained in 54 % yield after transmetalation of the organolithium to an organocuprate using CuCN ⋅ 2LiCl, followed by reaction with diphenyliodonium hexafluorophosphate (PhI_2_PF_6_).^[25]^ Nucleophilic substitution of alkyl halides (*n*‐pentyl iodide, allyl bromide and benzyl bromide) gave alkylated products **7 k**, **7 l** and **7 m** in 50 %, 48 % and 38 % yields respectively. Finally, trapping with *i*‐PrOBpin and Bu_3_SnCl gave **7 n** (51 % yield) and **7 o** (84 % yield) respectively.

Similarly, lithiation/trapping of tetrahydrothiopyran **5** worked well with a range of electrophiles (Scheme 2E), giving **8 a**–**8 l** in 39–91 % yields. Trapping with aryl aldehydes showed negligible diastereoselectivity to give alcohols **8 b** (62 %, 55 : 45 dr) and **8 c** (52 %%, 60 : 40 dr) respectively. In contrast, use of *N*‐benzylidene *tert*‐butylamine gave a single unidentified amine **8 e** in 71 % yield. Carbonyl‐containing products were obtained with a Weinreb amide, *t*‐butyl isocyanate and CO_2_. Ketone **8 a** (91 %) and amide **8 d** (89 %) were formed in high yields. With CO_2_, 2.5 eq. tetrahydrothiopyran **5** was used to aid separation from by‐products; this gave a moderate yield of acid **8 f** (39 %). Transmetalation of the lithiated species to boron and tin gave **8 k** (64 %) and **8 l** (87 %) in good yields; transmetalation to form a cuprate using CuCN ⋅ 2LiCl, and subsequent coupling with PhI_2_PF_6_ effected α‐arylation to give **8 g** in 50 % yield.

Next, the scope of the lithiation/trapping of *N*‐thiomorpholine **6** using both stoichiometric and substoichiometric TMEDA was studied (Scheme 2F). With stoichiometric TMEDA, trapping with ketones/aldehydes gave **9 a**–**9 e** (39–83 %). Allyl thiomorpholine **9 f** was isolated in 55 % yield using allyl bromide and silane **9 g** was generated in 72 % yield with PhMe_2_SiCl. Under substoichiometric conditions, **9 h**–**9 k** were obtained in 45–61 % yields and *n*‐pentyl thiomorpholine **9 l** was isolated in 35 % yield from trapping with *n*‐pentyl iodide.

To explore the potential for diastereoselectivity, the lithiation and trapping of 4‐phenyl and 4‐OTIPS tetrahydrothiopyrans **10 a** and **10 b** was investigated. Using the conditions developed for the lithiation/trapping of unsubstituted tetrahydrothiopyran **5**, disubstituted *cis*‐2,4‐tetrahydrothiopyrans **11** were isolated as single diastereomers (Scheme 3). Trapping with a range of electrophiles including benzaldehyde, *N*‐benzyl piperidin‐4‐one, CO_2_, phenyl Weinreb amide, paraformaldehyde, Me_2_SO_4_, allyl bromide, *i*‐PrOBpin and Bu_3_SnCl worked well to give 2,4‐*cis* adducts **11 a**–**11 j** in 20–87 % yields. Using phenyl Weinreb amide as the electrophile, ketone **11 d** was obtained in 68 % yield and the 2,4‐*cis* relative stereochemistry was confirmed by X‐ray crystallography.^[22]^ The stereochemistry of the other disubstituted tetrahydrothiopyrans was assigned by analogy and confirmed in most cases through analysis of the ^3^ *J* values in the ^1^H NMR spectra. We postulate that the high degree of *cis*‐stereoselectivity results from **10 a** and **10 b** adopting a chair conformation with the 4‐substituent in the equatorial position together with the preferential lithiation of an equatorial proton α to sulfur followed by retentive trapping. A similar model is well‐established for the lithiation/trapping of 4‐substituted *N*‐Boc piperidines.^[26]^ However, as the lithiation of tetrahydrothiopyrans **10 a** and **10 b** was carried out at 0 °C, an axial deprotonation and subsequent equilibration to an equatorially‐disposed thermodynamically preferred lithiated species (due to a configurationally unstable lithiated species) cannot be ruled out. Finally, *cis*‐2,4,6‐trisubstituted tetrahydropyran **13** was isolated in 41 % yield as a single diastereomer from *cis*‐2,4‐disubstituted substrate **12** (obtained from hydrogenation of **11 i**, see Supporting Information) and trapping with a Weinreb amide. Presumably, the 2,4,6‐*cis* selectivity follows a similar model to that for forming 2,4‐*cis* **11**, with lithiation occurring at the least hindered α ‐position.

**Scheme 3:**
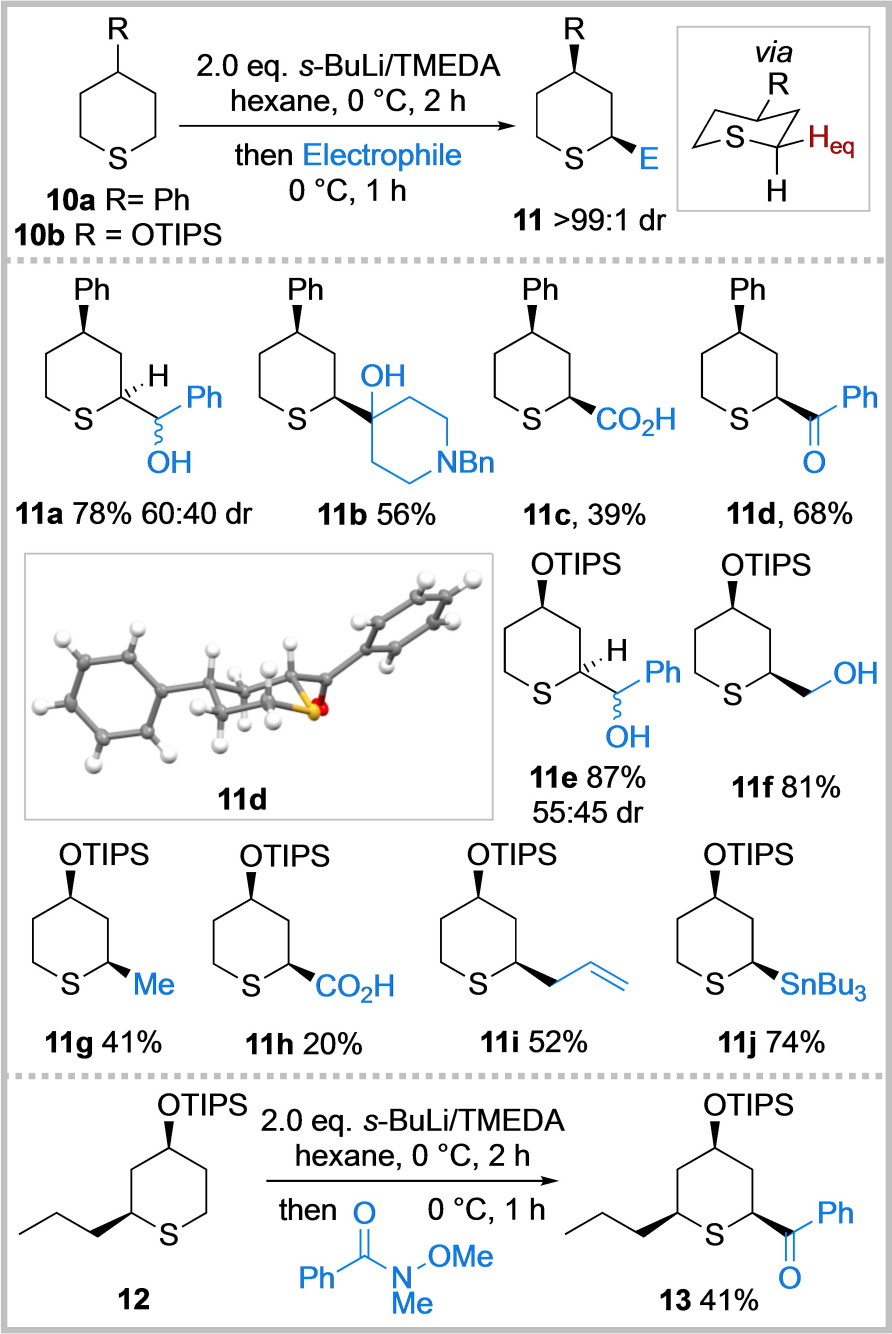
Diastereoselective functionalisation of 4‐substituted tetrahydrothiopyrans **10**. tri‐*iso*‐propylsilyl (TIPS); *N,N,N′,N′*‐tetramethylethylenediamine (TMEDA); benzyl (Bn).

Finally, to demonstrate the value of the products obtained from the direct lithiation/trapping of saturated cyclic sulfides, a target synthesis and two functionalisations of the trapped products were explored. For example, we applied the methodology to a new route to amino sulfide **15**, a key intermediate in the synthesis of CXCR antagonist **2** (see Figure 1).^[6]^ Thus, tetrahydrothiophene **4** was lithiated under standard conditions and trapped with the Weinreb amide of 2‐methyl furan to give ketone **7 p** in 72 % yield (Scheme 4A). Condensation with Ellman's sulfinamide gave separable diastereomeric sulfinyl imines **14 a** and **14 b** in 40 % and 38 % yields respectively. Next, reduction of the imine functionality of **14 b** using 9‐BBN (9‐borabicyclo[3.3.1]nonane)^[6]^ occurred with complete diastereoselectivity and subsequent amine deprotection under acidic conditions gave **15** (97 : 3 er) in 88 % yield over the two steps. The relative stereochemistry was established since **15** had identical ^1^H and ^13^C NMR spectroscopic data to those reported^[6]^ and they were different to those data for the HCl salt derived from **14 a**. In terms of distinct reactions, our approach is more concise than the previous approaches although those routes did deliver kg‐scale quantities of **15**.^[6]^ To show the value of the lithiation/trapping products, pinacol boronate **7 n** was arylated using Aggarwal's transition‐metal free stereospecific cross‐coupling of secondary boronic esters with aryl organolithiums (Scheme 4B).^[27]^ Addition of boronate **7 n** to 2‐lithiofuran followed by reaction with NBS (*N*‐bromosuccinimide) delivered α‐furyl tetrahydrothiophene **7 q** in 42 % yield. Similarly, α‐*N*‐methyl indole tetrahydrothiophene **7 r** was obtained in 38 % yield. In addition, MacMillan's photoredox‐mediated decarboxylative arylation of amino acids^[28]^ using aryl cyanides was deployed to access additional arylated cyclic sulfides (Scheme 4C). Exposure of tetrahydrothiophene carboxylic acid **7 h** and aryl nitriles to a compact fluorescent light source in the presence of an iridium photocatalyst and CsF gave aryl sulfides **7 s** and **7 t** in 69 % and 71 % yields respectively. Similarly, tetrahydrothiopyran **8 f** gave the α‐pyridyl analogue **8 m** in 39 % yield.

**Scheme 4:**
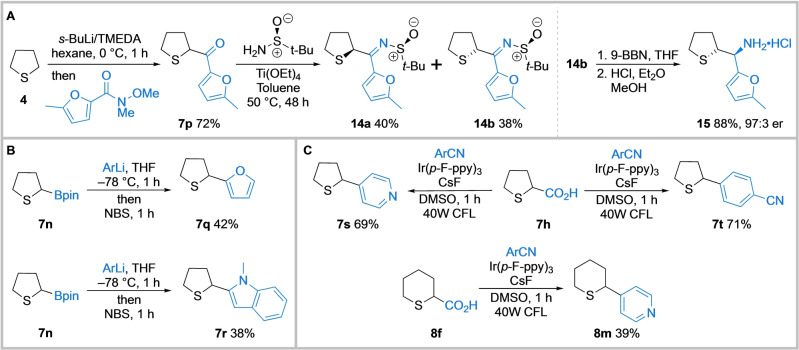
A: Preparation of a key intermediate in the synthesis of CXCR antagonist **2**. B and C: Further functionalisation of α‐substituted tetrahydrothiophenes and tetrahydrothiopyrans. *N,N,N′,N′*‐tetramethylethylene‐diamine (TMEDA); 9‐borabicyclo[3.3.1]nonane (9‐BBN); pinacolate (pin) *N*‐bromosuccinimide (NBS); tris(2‐(4‐fluorophenyl)pyridine (Ir(*p*‐F‐ppy)_3_); dimethylsulfoxide (DMSO); Compact fluorescent light (CFL).

In summary, we have developed a general and experimentally simple method (at 0/−10 °C) for the lithiation/trapping of tetrahydrothiophene **4**, tetrahydrothiopyrans **5** and **10** and *N*‐methyl thiomorpholine **6**. The efficient lithiation/trapping of *N*‐methyl thiomorpholine **6** using substoichiometric TMEDA is a rare example of the use of substoichiometric diamine in lithiations. In total, more than 50 examples of functionalising five different sulfur heterocycles are presented. Synthesis of an advanced intermediate of the CXCR antagonist **2** and arylation of trapped products highlights the synthetic utility of the readily accessible α‐substituted five‐ and six‐membered ring saturated cyclic sulfides. Our methodology provides access to a range of α‐substituted cyclic sulfides that are not easily synthesised by the currently available methods, especially those that proceed via a radical α to sulfur (Scheme 1A).

## Supporting Information

The authors have cited additional references within the Supporting Information.[^29^, ^30^, ^31^, ^32^, ^33^, ^34^, ^35^, ^36^, ^37^, ^38^, ^39^]

## Conflict of interest

The authors declare no conflict of interest.

## Supporting information

As a service to our authors and readers, this journal provides supporting information supplied by the authors. Such materials are peer reviewed and may be re‐organized for online delivery, but are not copy‐edited or typeset. Technical support issues arising from supporting information (other than missing files) should be addressed to the authors.

Supporting Information

## Data Availability

The data that support the findings of this study are available in the supplementary material of this article.
