# Supplementary material for: α‐Functionalisation of Cyclic Sulfides Enabled by Lithiation Trapping
Source: Angew Chem Int Ed Engl. 2023 Dec 7;63(2):e202314423. doi: 10.1002/anie.202314423 (PMC10952194; doi:10.1002/anie.202314423)
Supplement: Supplementary file 1 — Supporting Information [file ANIE-63-0-s001.pdf]

Angewandte Chemie
Eine Zeitschrift der Gesellschaft Deutscher Chemiker

Supporting Information

α-Functionalisation of Cyclic Sulfides Enabled by Lithiation Trapping

*N. Seling, M. Atobe, K. Kasten, J. D. Firth, P. B. Karadakov, F. W. Goldberg, P. O'Brien**

Index

1. Experimental Details S3

1.1 General Information S3

1.2 General Procedures S4

1.3 Experimental Procedures and Characterisation Data S6

2. ^{1}H and ^{13}C NMR Spectra S57

4. CSP-HPLC S128

4. References S129

1. Experimental Details

1.1 General Information

All-non aqueous reactions were carried out under oxygen free Ar or N_2 using flame-dried glassware. Hexane is *n*-hexane and was freshly distilled over CaH_2. Alkyllithiums were titrated against *N*-benzylbenzamide before use. Electrophiles (Me_2SO_4, PhCHO, PhC(O)N(OMe)Me) and TMEDA used in lithiation reactions were distilled over CaH_2 before use. Electrophiles (allyl bromide, benzyl bromide, *N*-Boc piperidin-4-one, Bu_3SnCl, $(CH_2O)_n$, *i*-PrOBpin, pentyl iodide, $PhSiMe_2Cl$) were used without further purification. When CO_2 was used as an electrophile, dry ice was filled in a flame-dried flask and the gas, which was evolving during sublimation was cannulated through flask of $CaCl_2$ before cannulated into the reaction flask. Brine refers to a saturated solution. Water is distilled water.
Flash column chromatography was carried out using Fluka Chemie GmbH silica (220-440 mesh). Thin layer chromatography was carried out using commercially available Merck F254 aluminium backed silica plates. Proton (400 MHz) and carbon (100.6 MHz) NMR spectra were recorded on a Jeol ECX-400 instrument using an internal deuterium lock. For samples recorded in $CDCl_3$, chemical shifts are quoted in parts per million relative to $CHCl_3$ (δ_H 7.26 ppm) and $CDCl_3$ (δ_C 77.16 ppm, central line of triplet). Carbon NMR spectra were recorded with broad band proton decoupling and assigned using DEPT experiments and/or HMQC/HMBC. Coupling constants (*J*) are quoted in Hertz and are rounded to the nearest 0.5 Hz. The ratios of diastereomers and rotamers are given out of 100 and are rounded to the nearest 5. Melting points were carried out on a Gallenkamp melting point apparatus. Infrared spectra were recorded on an ATI Mattson Genesis FT-IR spectrometer. Electrospray high and low resonance mass spectra were recorded at room temperature on a Bruker Daltronics microOTOF spectrometer. Optical rotations were recorded at room temperature on a Jasco DIP-370 polarimeter (using sodium D line, 589 nm) and $[\alpha]_D$ given in units of 10^{-1} deg cm^3 g^{-1}.

1.2 General Procedures

General Procedure A: Diamine-mediated lithiation-trapping of sulfur heterocycles using *s*-BuLi/TMEDA at temperatures between 0 °C and −78 °C.

s-BuLi (1.3 M solution in 92/8 cyclohexane/hexanes, 1.3 eq. – 2.0 eq.) was added dropwise to a stirred solution of the cyclic thioether (1.0 mmol, 1.0 eq.) and TMEDA (0.3 eq. – 2.0 eq.) in hexane (5.0 mL) at a specified temperature under Ar. The resulting solution was stirred at the specified temperature for the specified time (2 min – 4 h). Then, the electrophile (2.0 eq. – 3.0 eq.) was added. The resulting solution was stirred at the specificed temperature for 1 – 3 h and then allowed to warm to rt. Then, saturated $NH_4Cl_{(aq)}$ (10 mL) and 10% $NaHCO_{3(aq)}$ (15 mL) were added and the two layers were separated. The aqueous layer was extracted with Et_2O (3 × 10 mL). The combined organic layers were dried ($MgSO_4$) and evaporated under reduced pressure to give the crude product.

General Procedure B: Diamine-mediated lithiation-trapping of sulfur heterocycles using *s*-BuLi/TMEDA at temperatures between 0 °C and −78 °C and CO_2.

s-BuLi (1.3 M solution in 92/8 cyclohexane/hexanes, 1.3 eq.) was added dropwise to a stirred solution of the cyclic thioether (1.0 – 3.3 eq.) and TMEDA (1.3 eq.) in hexane (5.0 mL) at a specfied temperature under Ar. The resulting solution was stirred at the specified temperature for the specified time (2 min – 4 h). Then, a stream of CO_2 was bubbled into the solution for 1 h and the resulting solution was allowed to warm up to rt. Then, 1 M $HCl_{(aq)}$ (10 mL) and 10% was added and the two layers were separated. The aqueous layer was extracted with Et_2O (3 × 10 mL). The combined organic layers were dried ($MgSO_4$) and evaporated under reduced pressure to give the crude product.

General Procedure C: Decarboxylative Arylation of Thioether 2-Carboxylic Acid.

To a stirred solution of Ir(*p*-F(*t*-Bu)-ppy)$_3$ (2.1 mg, 2.4 µmol, 0.02 eq.), thioether-2-carboxylic acid (0.36 mmol, 3.0 eq.) and CsF (55 mg, 0.4 mmol, 3.0 eq.) in DMSO (6.0 mL) was added 1,4-dicyano benzene (17.3 mg, 0.12 mmol, 1.0 eq.). The solution was degassed with Ar for 10 min and then irradiated with a 40 W fluorescent lamp (approximately 2 cm away from the light source) for 48 h at rt. Then, saturated $NaHCO_{3(aq)}$ (10 mL) was added and the two layers were separated. The aqueous layer was extracted with EtOAc (3 × 15 mL). The combined organic layers were dried ($MgSO_4$) and evaporated under reduced pressure to give the crude product.

General Procedure for the Preparation of CuCN·2LiCl in THF

LiCl (126 mg, 3.0 mmol, 2.0 eq.) was stirred and heated at 200 °C for 5 h under Ar. After cooling down to rt, THF (3 mL) was added and the mixture was stirred at rt for 30 min to give a clear solution. Then, CuCN (267 mg, 1.50 mmol, 1.0 eq.) was added and the resulting solution was stirred at rt for 30 min. This gave a 0.5 M solution of CuCN·2LiCl in THF.

1.3 Experimental Procedures and Characterisation Data

Dimethyl(phenyl)(thiolan-2-yl)silane 7a

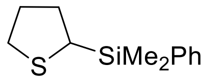

7a

(Scheme 2A, entry 1)

Using general procedure A, tetrahydrothiophene **4** (88 mg, 1.0 mmol, 1.0 eq.), *s*-BuLi (1.0 mL of a 1.3 M solution in 92/8 cyclohexane/hexane, 1.3 mmol, 1.3 eq.) and TMEDA (195 µL, 1.3 mmol, 1.3 eq.) in hexane (5 mL) at 0 °C for 1 h and $PhSiMe_2Cl$ (340 mg, 355 µL, 2.0 mmol, 2.0 eq.) at 0 °C for 1 h gave the crude product. Purification by flash column chromatography on silica with 80:20 hexane-CH_2Cl_2 as eluent gave silane **7a** (191 mg, 86%) as a colourless oil, R_F (75:25 hexane-CH_2Cl_2) 0.4; IR (ATR) 2932, 2860, 1452, 1441, 1263, 1189, 1020, 762, 734, 698, 529, 556 cm^{-1}; ^1H NMR (400 MHz, $CDCl_3$) δ 7.59-7.55 (m, 2H, Ph), 7.39-7.33 (m, 3H, Ph), 2.91 (ddd, J = 11.0, 8.0, 2.5 Hz, 1H, SCH), 2.69 (ddd, J = 11.0, 11.0 , 6.0 Hz, 1H, SCH), 2.59 (dd, J = 11.5, 6.0 Hz, 1H, SCH), 2.24-2.15 (m, 2H, CH), 1.74-1.60 (m, 1H, CH), 1.49 (dddd, J = 11.5, 11.5, 11.5, 6.0 Hz, 1H, CH), 0.37 (s, 3H, SiMe), 0.36 (s, 3H, SiMe); $^{13}C\{^1H\}$ NMR (100.6 MHz, $CDCl_3$) δ 138.2 (*ipso*-Ph), 134.4 (Ph), 129.7 (Ph), 128.2 (Ph), 34.1 (CH_2), 33.6 (SCH), 33.0 (CH_2), 32.9 (CH_2), –3.6 (SiMe), –4.3 (SiMe). Attempted characterisation by MS was unsuccessful.

Lab Book Reference: NS3-21

(Scheme 2A, entry 2)

Using general procedure A, tetrahydrothiophene **4** (88 mg, 1.0 mmol, 1.0 eq.), *s*-BuLi (1.0 mL of a 1.3 M solution in 92/8 cyclohexane/hexane, 1.3 mmol, 1.3 eq.) and TMEDA (195 µL, 1.3 mmol, 1.3 eq.) in hexane (5 mL) at 0 °C for 20 min and $PhSiMe_2Cl$ (340 mg, 355 µL, 2.0 mmol, 2.0 eq.) at 0 °C for 1 h gave the crude product. Purification by flash column chromatography on silica with 80:20 hexane-CH_2Cl_2 as eluent gave silane **7a** (145 mg, 66%) as a colourless oil.

Lab Book Reference: NS3-20

Phenyl(thiolan-2-yl)methanol 7b

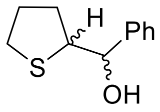

7b

Using general procedure A, tetrahydrothiophene **4** (88 mg, 1.0 mmol, 1.0 eq.), *s*-BuLi (1.0 mL of a 1.3 M solution in 92/8 cyclohexane/hexane, 1.3 mmol, 1.3 eq.) and TMEDA (195 µL, 1.3 mmol, 1.3 eq.) in hexane (5 mL) at 0 °C for 1 h and benzaldehyde (210 mg, 201 µL, 2.0 mmol, 2.0 eq.) at 0 °C for 1 h gave the crude product which contained a 55:45 mixture (by ^{1}H NMR spectroscopy) of alcohols **7b**. Purification by flash column chromatography on silica with 95:5 CH_2Cl_2-acetone then 80:20 CH_2Cl_2-acetone as eluent gave a 60:40 mixture of alcohols **7b** (168 mg, 87%) as a colourless oil, R_F (90:10 CH_2Cl_2-acetone) 0.3; IR (ATR) 3402 (OH), 2932, 2860, 1452, 1020, 698 cm^{-1}; ^{1}H NMR (400 MHz, $CDCl_3$) δ 7.41 – 7.26 (m, 5H, Ph), 4.74 (dd, J = 5.0, 2.0 Hz, 0.6H, C*H*OH), 4.54 (dd, J = 6.0, 6.0 Hz, 0.4H, C*H*OH), 3.84 – 3.74 (m, 1H, SCH), 2.95 – 2.82 (m, 2H, CH), 2.80 (d, J = 5.5 Hz, 0.4H, OH), 2.70 (d, J = 2.0 Hz, 0.6H, OH), 2.18 – 2.05 (m, 1H, CH), 2.00 – 1.72 (m, 3H, CH); ^{13}C{^{1}H} NMR (100.6 MHz, $CDCl_3$) δ 143.0 (*ipso*-Ph), 142.1 (*ipso*-Ph), 128.6 (Ph), 128.5 (Ph), 128.0 (Ph), 127.8 (Ph), 126.3 (Ph), 126.2 (Ph), 76.8 (HOCH), 74.8 (HOCH), 57.9 (SCH), 56.5 (SCH), 34.2 (CH_2), 33.3 (CH_2), 32.7 (CH_2), 31.4 (CH_2), 31.1 (CH_2), 30.9 (CH_2); HRMS (ESI) *m/z* calcd for $C_{11}H_{14}OS$ (M + Na)$^+$ 217.0658, found 217.0653 (+2.1 ppm error).

Lab Book Reference: NS3-24

(Scheme 2A, entry 3)

Using general procedure A, tetrahydrothiophene **4** (88 mg, 1.0 mmol, 1.0 eq.), *n*-BuLi (0.65 mL of a 1.7 M solution in hexane, 1.3 mmol, 1.3 eq.) and TMEDA (195 µL, 1.3 mmol, 1.3 eq.) in hexane (5 mL) at 0 °C for 1 h and benzaldehyde (210 mg, 201 µL, 2.0 mmol, 2.0 eq.) at 0 °C for 1 h gave the crude product which did not contain the desired alcohols **7b** (by ^{1}H NMR spectroscopy).

Lab Book Reference: NS3-32

(Scheme 2A, entry 4)

Using general procedure A, tetrahydrothiophene **4** (88 mg, 1.0 mmol, 1.0 eq.), *s*-BuLi (1.0 mL of a 1.3 M solution in hexane, 1.3 mmol, 1.3 eq.) and TMEDA (45 µL, 0.33 mmol, 0.3 eq.) in hexane (5 mL) at 0 °C for 3 h and benzaldehyde (210 mg, 201 µL, 2.0 mmol, 2.0 eq.) at 0 °C for 1 h gave the crude product which contained a 55:45 mixture (by ^{1}H NMR spectroscopy) of alcohols **7b**. Purification by flash column chromatography on silica with 95:5 CH_2Cl_2-acetone then 80:20 CH_2Cl_2-acetone as eluent gave a 60:40 mixture of alcohols **7b** (60 mg, 31%) as a colourless oil.

Lab Book Reference: NS3-37

(4-Bromophenyl)(thiolan-2-yl)methanol 7c

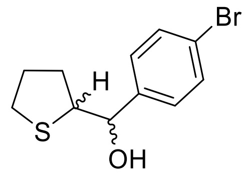

7c

Using general procedure A, tetrahydrothiophene **4** (191 mg, 1.0 mmol, 1.0 eq.), *s*-BuLi (0.77 mL of a 1.3 M solution in 92/8 cyclohexane/hexane, 1.0 mmol, 1.0 eq.) and TMEDA (195 µL, 1.3 mmol, 1.3 eq.) in hexane (5 mL) at 0 °C for 1 h and 4-bromobenzaldehyde (270 mg, 2.0 mmol, 2.0 eq.) at 0 °C for 1 h gave the crude product which contained a 50:50 mixture (by ^{1}H NMR spectroscopy) of alcohols **7c**. Purification by flash column chromatography on silica with 99:1 to 98:2 CH_2Cl_2-acetone as eluent gave a 50:50 mixture of alcohols **7c** (168 mg, 62%) as a colourless oil, R_F (98:2 CH_2Cl_2-acetone) 0.6; IR (ATR) 3402 (OH), 2946, 2859, 1487, 1263, 1184, 1070, 1008, 818, 736, 531, 506 cm^{-1}; ^{1}H NMR (400 MHz, $CDCl_3$) δ 7.50-7.44 (m, 2H, Ar), 7.28-7.19 (m, 2H, Ar), 4.73 (dd, J = 7.0, 4.5 Hz, 0.5H, HOC*H*), 4.51 (dd, J = 6.0, 6.0 Hz, 0.5H, HOC*H*), 3.78-3.67 (m, 1H, SCH), 2.98-2.87 (m, 2.5H, CH, OH), 2.78-2.75 (m, 0.5H, CH), 2.65-2.57 (m, 1H, CH), 2.28-2.19 (m, 1H, CH), 2.18-2.10 (m, 1H, CH), 2.02-1.93 (m, 1H, CH); ^{13}C{^{1}H} NMR (100.6 MHz, $CDCl_3$) δ 142.0 (*ipso*-Ar), 141.0 (*ipso*-Ar), 131.7 (*ipso*-Ar), 131.6 (*ipso*-Ar), 128.0 (Ar), 127.9 (Ar), 121.8 (Ar), 121.5 (Ar), 75.9 (OCH), 74.0 (OCH), 57.8 (SCH), 56.3 (SCH), 34.1 (CH_2), 33.3 (CH_2), 32.7 (CH_2), 31.4 (CH_2), 30.92 (CH_2), 30.91 (CH_2); HRMS (ESI) *m/z* calcd for $C_{11}H_{13}{}^{79}BrOS$ (M(^{79}Br) + Na)$^+$ 294.9763, found 294.9764 (–0.6 ppm error).

Lab Book Reference: NS5-83

1-Benzyl-4-(thiolan-2-yl)piperidin-4-ol 7d

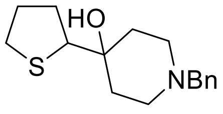

7d

Using general procedure A, tetrahydrothiophene 4 (88 mg, 1.0 mmol, 1.0 eq.), *s*-BuLi (1.0 mL of a 1.3 M solution in 92/8 cyclohexane/hexane, 1.3 mmol, 1.3 eq.) and TMEDA (195 μL, 1.3 mmol, 1.3 eq.) in hexane (5 mL) at 0 °C for 1 h and 1-benzylpiperidin-4-one (378 mg, 371 μL, 2.0 mmol, 2.0 eq.) at 0 °C for 1 h gave the crude product. Purification by flash column chromatography on silica with 500:9:1 CH_2Cl_2-MeOH-$NH_4OH_{(aq)}$ then 100:9:1 CH_2Cl_2-MeOH-$NH_4OH_{(aq)}$ as eluent gave alcohol **7d** (198 mg, 61%) as a red oil, R_F (500:9:1 CH_2Cl_2-MeOH-$NH_4OH_{(aq)}$) 0.3; IR (ATR) 3468 (OH), 2932, 2859, 2810, 1453, 1316, 1185, 1145, 1063, 1047, 811, 735, 697, 466 cm^{-1}; ^1H NMR (400 MHz, $CDCl_3$) δ 7.36-7.22 (m, 5H, Ph), 3.52 (s, 2H, NCH_2Ph), 3.47 (dd, J = 9.5, 5.5 Hz, 1H, SCH), 2.86-2.79 (m, 1H, SCH), 2.75 (ddd, J = 9.5, 5.5, 5.5 Hz, 1H, SCH), 2.72-2.59 (m, 2H, CH), 2.40-2.30 (m, 2H, CH), 2.24 (br s, 1H, OH), 2.22-2.18 (m, 1H, CH), 2.05-1.99 (m, 1H, CH), 1.87-1.55 (m, 5H, CH), 1.50 (dddd, J = 13.5, 3.0, 3.0, 3.0, Hz, 1H, CH); $^{13}C\{^1H\}$ NMR (100.6 MHz, $CDCl_3$) δ 138.6 (*ipso*-Ph), 129.3 (Ph), 128.3 (Ph), 127.1 (Ph), 69.2 (OC), 63.3 (N*C*H_2Ph), 61.7 (SCH), 49.7 (NCH_2), 49.4 (NCH_2), 39.6 (CH_2), 34.8 (CH_2), 32.3 (SCH_2), 31.9 (CH_2), 30.5 (CH_2); HRMS (ESI) *m/z* calcd for $C_{16}H_{23}NOS$ $(M + H)^+$ 278.1573, found 278.1572 (–0.3 ppm error).

Lab Book Reference: NS3-48

(1*S*,3*bR*,11*aS*)-7-[(*tert*-Butyldimethylsilyl)oxy]-11a-methyl-1-(thiolan-2-yl)-1H,2H,3H,3aH,3bH,4H,5H,9bH,10H,11H,11aH-cyclopenta[a]phenanthren-1-ol 7e

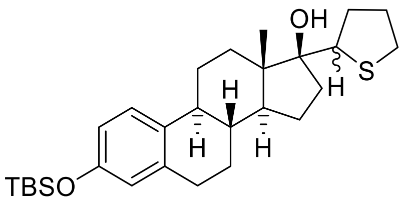

7e

Using general procedure A, tetrahydrothiophene **4** (44 mg, 0.5 mmol, 1.0 eq.), *s*-BuLi (0.5 mL of a 1.3 M solution in 92/8 cyclohexane/hexane, 0.65 mmol, 1.3 eq.) and TMEDA (98 µL, 0.65 mmol, 1.3 eq.) in hexane (2.5 mL) at 0 °C for 1 h and TBS-estrone[1] (385 mg, 1.0 mmol, 2.0 eq.) at 0 °C for 1 h gave the crude product. Purification by flash column chromatography on silica with 99:1 CH_2Cl_2-acetone then 95:5 CH_2Cl_2-acetone gave an 85:15 mixture of alcohols **7e** (103 mg, 45%) as a colourless oil, R_F (CH_2Cl_2) 0.6; IR (ATR) 3410 (OH), 2929, 2857, 1496, 1285, 1254, 953, 837, 817, 779 cm^{-1}; ^{1}H NMR (400 MHz, $CDCl_3$) δ 7.10 (d, J = 8.5 Hz, 1H, Ar), 6.60 (dd, J = 8.5, 2.5 Hz, 1H, Ar), 6.54 (d, J = 2.5 Hz, 1H, Ar), 3.81 (dd, J = 10.0, 6.5 Hz, 0.85H, CH), 3.05 (s, 0.85H, OH), 2.85-2.77 (m, 2.30H, CH), 2.73 (ddd, J = 10.5, 10.5, 5.0 Hz, 1H, CH), 2.36-2.19 (m, 3H, CH), 2.13 (ddd, J = 11.5, 11.0, 4.0 Hz, 1H, CH), 2.06-1.59 (m, 7H, CH, OH), 1.59-1.12 (m, 7H, CH), 0.98 (s, 9H, CMe_3), 0.89 (s, 3H, Me), 0.19 (s, 6H, $SiMe_2$); ^{13}C{^{1}H} NMR (100.6 MHz, $CDCl_3$) δ for major diastereomer 153.5 (*ipso*-Ar), 137.9 (*ipso*-Ar), 133.1 (*ipso*-Ar), 126.3 (Ar), 120.0 (Ar), 117.3 (Ar), 84.2 (HOC), 57.7 (CH), 50.9 (CH), 47.9 (C), 43.7 (CH), 39.7 (CH), 34.6 (CH_2), 33.9 (CH_2), 33.6 (CH_2), 31.4 (CH_2), 29.8 (CH_2), 27.7 (CH_2), 26.7 (CH_2), 25.9 (C*Me*$_3$), 23.4 (CH_2), 18.3 (*C*Me$_3$), 15.0 (CH_2), –4.24 (SiMe); HRMS (ESI) *m/z* calcd for $C_{28}H_{44}O_2SSi$ $(M + Na)^+$ 495.272349, found 495.271406 (–1.9 ppm error).

Lab Book Reference: NS3-74

Phenyl(thiolan-2-yl)methanone 7f

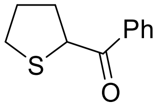

7f

Using general procedure A, tetrahydrothiophene **4** (191 mg, 1.0 mmol, 1.0 eq.), *s*-BuLi (1.0 mL of a 1.3 M solution in 92/8 cyclohexane/hexane, 1.3 mmol, 1.3 eq.) and TMEDA (195 µL, 1.3 mmol, 1.3 eq.) in hexane (5 mL) at 0 °C for 1 h and *N*-methoxy-*N*-methylbenzamide (304 µL, 2.0 mmol, 2.0 eq.) at 0 °C

for 1 h gave the crude product. Purification by flash column chromatography on silica 50:50 hexane-CH_2Cl_2 as eluent gave ketone **7f** (162 mg, 84%) as a white solid, mp 55-56 °C; R_F (50:50 hexane-CH_2Cl_2) 0.3; IR (ATR) 3059, 2934, 2861, 1679 (C=O), 1596, 1447, 1219, 688 cm^{-1}; ^1H NMR (400 MHz, $CDCl_3$) δ 7.97-7.92 (m, 2H, Ph), 7.58-7.53 (m, 1H, Ph), 7.49-7.44 (m, 2H, Ph), 4.73 (dd, J = 7.0, 4.5 Hz, 1H, SCH), 2.98-2.87 (m, 2H, SCH), 2.65-2.57 (m, 1H, CH), 2.28-2.19 (m, 1H, CH), 2.18-2.10 (m, 1H, CH), 2.02-1.93 (m, 1H, CH); $^{13}C\{^1H\}$ NMR (100.6 MHz, $CDCl_3$) δ 196.5 (C=O), 136.0 (*ipso*-Ph), 133.2 (Ph), 128.7 (Ph), 128.7 (Ph), 49.4 (SCH), 34.1 (SCH_2), 31.4 (CH_2), 31.3 (CH_2); HRMS (ESI) *m/z* calcd for $C_{11}H_{12}OS$ $(M + H)^+$ 193.0682, found 193.0685 (–0.6 ppm error). Spectroscopic data consistent with those reported in the literature.[2]
Lab Book Reference: NS3-26

(Thiolan-2-yl)methanol 7g

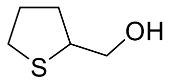

7g

Using general procedure A, tetrahydrothiophene **4** (88 mg, 1.0 mmol, 1.0 eq.), *s*-BuLi (1.0 mL of a 1.3 M solution in 92/8 cyclohexane/hexane, 1.3 mmol, 1.3 eq.) and TMEDA (195 µL, 1.3 mmol, 1.3 eq.) in hexane (5 mL) at 0 °C for 1 h and paraformaldehyde (60 mg, 2.0 mmol, 2.0 eq.) at 0 °C for 1 h gave the crude product. Purification by flash column chromatography on silica with 500:9:1 CH_2Cl_2-MeOH-$NH_4OH_{(aq)}$ then 300:9:1 CH_2Cl_2-MeOH-$NH_4OH_{(aq)}$ as eluent gave alcohol **7g** (92 mg, 78%) as a colourless oil, R_F (500:9:1 CH_2Cl_2-MeOH-$NH_4OH_{(aq)}$) 0.3; IR (ATR) 3351 (OH), 2934, 2861, 1441, 1019, 732 cm^{-1}; ^1H NMR (400 MHz, $CDCl_3$) δ 3.66-3.56 (m, 2H, OCH, SCH), 3.52 (dd, J = 10.0, 5.0 Hz, 1H, OCH), 2.89-2.81 (m, 2H, SCH), 2.09 (br s, 1H, OH), 2.05-1.95 (m, 3H, CH), 1.88-1.77 (m, 1H, CH); $^{13}C\{^1H\}$ NMR (100.6 MHz, $CDCl_3$) δ 65.7 (OCH_2), 51.1 (SCH), 33.2 (CH_2), 32.6 (CH_2), 30.7 (CH_2); HRMS (ESI) *m/z* calcd for $C_5H_{10}OS$ $(M + Na)^+$ 141.0345, found 141.0351 (+4.6 ppm error).
Lab Book Reference: NS3-25

Thiolane-2-carboxylic acid 7h

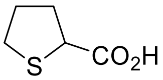

7h

Using general procedure B, tetrahydrothiophene **4** (1.76 g, 20 mmol, 1.0 eq.), *s*-BuLi (20 mL of a 1.3 M solution in 92/8 cyclohexane/hexane, 26 mmol, 1.3 eq.) and TMEDA (3.90 mL, 26 mmol, 1.3 eq.) in hexane (100 mL) at 0 °C for 1 h and CO_2 at −78 °C for 30 min and then at rt for 30 min gave the crude product. Purification by flash column chromatography on silica with CH_2Cl_2 then 99:1 CH_2Cl_2-AcOH gave acid **7h** (2.19 g, 83%) as a viscous oil, R_F (CH_2Cl_2) 0.1, IR (ATR) 2935, 1698 (C=O), 1440, 1416, 1225, 925, 798, 680, 537, 501 cm^{-1}; ^{1}H NMR (400 MHz, $CDCl_3$) δ 11.11 (br s, 1H, CO_2H), 3.92 (dd, *J* = 7.0, 4.5 Hz, 1H, SCH), 2.97 (ddd, *J* = 10.0, 6.5, 5.5 Hz, 1H, SCH), 2.89 (ddd, *J* = 10.0, 7.5, 6.5 Hz, 1H, SCH), 2.28 (dddd, *J* = 12.5, 5.5, 5.5, 4.5 Hz, 1H, CH), 2.16 (ddddd, *J* = 12.5, 8.5, 6.5, 6.5, 5.5 Hz, 1H, CH), 2.07 (dddd, *J* = 12.5, 6.0, 6.0, 6.0 Hz, 1H, CH), 1.97 (dddd, *J* = 12.5, 8.5, 7.0, 5.5 Hz, 1H, CH); ^{13}C{^{1}H} NMR (100 MHz, $CDCl_3$) δ 180.1 (C=O), 47.5 (SCH), 33.2 (CH_2), 33.3 (SCH_2), 30.9 (CH_2); HRMS (ESI) *m/z* calcd for $C_5H_8O_2S$ (M + Na)$^+$ 155.0137, found 155.0138 (–0.8 ppm error). Spectroscopic data consistent with those reported in the literature.[3]

Lab Book Reference: NS11-53

(*S*)-2-Methyl-*N*-[phenyl(thiolan-2-yl)methyl]propane-2-sulfinamide (*S*)-S1

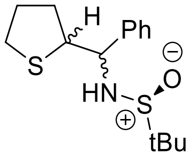

(*S*)-**S1**

Using general procedure A, tetrahydrothiophene **4** (176 mg, 2.0 mmol, 1.0 eq.), *s*-BuLi (2.0 mL of a 1.3 M solution in 92/8 cyclohexane/hexane, 2.6 mmol, 1.3 eq.) and TMEDA (390 µL, 2.6 mmol, 1.3 eq.) in hexane (10 mL) at 0 °C for 1 h and (*S*)-*N*-benzylidene-2-methylpropane-2-sulfinamide (836 mg, 4.0 mmol, 2.0 eq.) at 0 °C for 1 h gave the crude product which contained a 40:25:25:10 mixture (by ^{1}H NMR spectroscopy) of sulfinamides (*S*)-**S1**. Purification by flash column chromatography on silica with 98:2 CH_2Cl_2-acetone then 400:9:1 CH_2Cl_2-MeOH-$NH_4OH_{(aq)}$ as eluent gave a 40:25:25:10 mixture of sulfinamides (*S*)-**S1** (529 mg, 89%) as a yellow oil, R_F (98:2 CH_2Cl_2-acetone) 0.4; IR (ATR) 3221 (NH), 2952, 2863, 1454, 1363, 1055, 909, 700 cm^{-1}; ^{1}H NMR (400 MHz, $CDCl_3$) δ 7.43 – 7.27 (m, 5H, Ph), 4.50 (dd, *J* = 6.0, 2.0 Hz, 0.15), 4.41 (dd, *J* = 8.0, 5.0 Hz, 0.30H), 4.39 (br d, *J* = 3.0 Hz, 0.15H), 4.34

(dd, *J* = 8.0, 7.0 Hz, 0.4H), 4.19 (dd, *J* = 8.5, 3.0 Hz, 0.15H), 4.11 (br d, *J* = 2.0 Hz, 0.15H), 4.08 (d, *J* = 8.0 Hz, 0.30H), 4.01 (ddd, *J* = 7.0, 7.0, 7.0 Hz, 0.30H), 3.87 – 3.76 (m, 0.40H), 3.66 – 3.62 (m, 0.40H), 2.98 – 2.65 (m, 1.85H), 2.22 – 1.58 (m, 4.45H), 1.25 – 1.17 (m, 9H, CMe3); ^{13}C NMR (100.6 MHz, $CDCl_3$) δ 142.0 (*ipso*-Ph), 140.82 (*ipso*-Ph), 140.78 (*ipso*-Ph), 140.0 (*ipso*-Ph), 128.8 (Ph), 128.65 (Ph), 128.63 (Ph), 128.4 (Ph), 128.30 (Ph), 128.27 (Ph), 128.17 (Ph), 128.13 (Ph), 128.08 (Ph), 128.06 (Ph), 128.0 (Ph), 127.1 (Ph), 64.3 (NCH), 63.8 (NCH), 62.7 (NCH), 60.6 (NCH), 56.9 (*C*Me3), 56.8 (*C*Me3), 56.49 (*C*Me3), 56.46 (*C*Me3), 55.9 (SCH), 55.69 (SCH), 55.65 (SCH), 54.1 (SCH), 34.8 (CH_2), 34.14 (CH_2), 34.12 (CH_2), 32.9 (CH_2), 32.7 (CH_2), 32.5 (CH_2), 32.3 (CH_2), 31.02 (CH_2), 30.99 (CH_2), 30.6 (CH_2), 29.8 (CH_2), 22.83 (C*Me*3), 22.75 (C*Me*3), 22.73 (C*Me*3), 22.72 (C*Me*3) (one resonance not resolved); HRMS (ESI) *m/z* calcd for $C_{15}H_{23}NOS_2$ $(M + H)^+$ 320.1113, found 320.1112 (+0.5 ppm error).
Lab Book Reference: NS5-82

1-Phenyl-1-(thiolan-2-yl)methanamine 7i•HCl

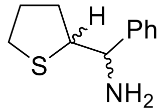

7i

Concentrated $HCl_{(aq)}$ (2.0 mL, 24 mmol, 24 eq.) was added to a stirred solution of a 40:25:25:10 mixture of sulfinamides (*S*)-**S1** (297 mg, 1.0 mmol, 1.0 eq.) in MeOH (3 mL) at rt. The resulting solution was stirred at rt for 14 h. Then, the solvent was evaporated under reduced pressure to give the crude product which contained a 65:35 mixture (by ^{1}H NMR spectroscopy) of amines **7i**·HCl. Purification by flash column chromatography on silica with 75:25 hexane-Et_2O as eluent gave a 65:35 mixture of amines **7i**·HCl (175 mg, 91%, 81% from **4**) as a colourless oil, R_F (75:25 hexane-Et_2O) 0.2; IR (ATR) 3026, 2943, 2859, 1661, 1451, 1440, 1295, 1178, 887, 848, 762, 699, 599 cm^{-1}; ^{1}H NMR (400 MHz, $CDCl_3$) δ 7.47-7.04 (m, 5H, Ph), 4.02 (d, *J* = 7.0 Hz, 0.35H, NCH), 3.81 (d, *J* = 8.0 Hz, 0.65H, NCH), 3.76-3.72 (m, 0.35H, SCH), 3.68-3.63 (m, 0.65H, SCH), 2.96-2.77 (m, 2H, SCH), 2.52-2.30 (br s, 3H, NH_3), 2.13-1.99 (m, 1.35H, CH), 1.95-1.81 (m, 1.30H, CH), 1.81-1.67 (m, 0.70H, CH), 1.66-1.52 (m, 0.65H, CH); $^{13}C\{^1H\}$ NMR (100.6 MHz, $CDCl_3$) δ 144.7 (*ipso*-Ph), 144.3 (*ipso*-Ph), 128.6 (Ph), 128.5 (Ph), 127.6 (Ph), 127.5 (Ph), 127.0 (Ph), 126.9 (Ph), 62.0 (NCH), 59.9 (NCH), 57.9 (SCH), 56.3 (SCH), 34.8 (SCH_2), 32.9 (SCH_2), 32.7 (CH_2), 31.3 (CH_2), 31.1 (CH_2), 30.8 (CH_2); HRMS (ESI) *m/z* calcd for $C_{11}H_{16}N$ $(M + H)^+$ 194.0998, found 194.0998 (–0.1 ppm error).
Lab Book Reference: NS5-85

2-Phenylthiolane 7j

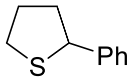

7j

s-BuLi (0.5 mL of a 1.3 M solution in 92/8 cyclohexane/hexane, 0.65 mmol, 1.3 eq.) was added dropwise to a stirred solution of tetrahydrothiophene **4** (50 µL, 0.50 mmol, 1.0 eq.), and TMEDA (97 µL, 0.65 mmol, 1.3 eq.) in hexane (2.5 mL) at 0 °C under Ar. The resulting solution was stirred at 0 °C for 1 h. Then, the solution was cooled to −78 °C and a freshly prepared solution of CuCN·2LiCl in THF (prepared from CuCN (58 mg, 0.65 mmol) and LiCl (55 mg, 1.3 mmol) in THF (1.3 mL)) was added. The resulting solution was allowed to warm to −35 °C and stirred at −35 °C for 15 min. Then, Ph_2IPF_6 (277 mg, 0.65 mmol, 1.3 eq.) was added in one portion and the resulting solution was stirred at −35 °C for 15 min and then allowed to warm to rt over 4 h. Saturated $NH_4OH_{(aq)}$ (20 mL) was added and the two layers were separated. The aqueous layer was extracted with Et_2O (3 × 20 mL). The combined organic layers were dried ($MgSO_4$) and evaporated under reduced pressure to give the crude product. Purification by flash column chromatography on silica with hexane then 9:1 hexane-CH_2Cl_2 gave α-phenyl sulfide **7j** (44 mg, 54%) as a colourless oil, R_F (80:20 hexane-CH_2Cl_2) 0.5; IR (ATR) 3026, 2945, 2859, 1490, 1451, 1440, 1260, 1156, 1099, 758, 697, 521 cm^{-1}; ^{1}H NMR (400 MHz, $CDCl_3$) δ 7.44-7.39 (m, 2H, Ph), 7.33-7.28 (m, 2H, Ph), 7.25-7.18 (m, 1H, Ph), 4.52 (dd, J = 8.5, 6.0 Hz, 1H, SCH), 3.16 (ddd, J = 10.5, 8.5, 6.5 Hz,1H, SCH), 3.02 (ddd, J = 10.5, 6.5, 4.0 Hz, 1H, SCH), 2.43-2.35 (m, 1H, CH), 2.32-2.24 (m, 1H, CH), 2.07-1.88 (m, 2H, CH); ^{13}C{^{1}H} NMR (100.6 MHz, $CDCl_3$) δ 143.1 (*ipso*-Ph), 128.5 (Ph), 127.8 (Ph), 127.1 (Ph), 52.9 (SCH), 40.7 (SCH_2), 33.6 (CH_2), 31.2 (CH_2); HRMS (APCI) *m/z* calcd for $C_{10}H_{12}S$ $(M + H)^+$ 165.0732, found 165.0729 (−2.3 ppm error). Spectroscopic data consistent with those reported in the literature.[5]

Lab Book Reference: NS7-18

2-Pentylthiolane 7k

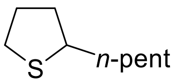

7k

Using general procedure A, tetrahydrothiophene **4** (176 mg, 2.0 mmol, 1.0 eq.), *s*-BuLi (2.0 mL of a 1.3 M solution in 92/8 cyclohexane/hexane, 2.6 mmol, 1.3 eq.) and TMEDA (390 µL, 2.6 mmol, 1.3 eq.) in hexane (10 mL) at 0 °C for 1 h and *n*-pentyl iodide (522 µL, 4.0 mmol, 2.0 eq.) at 0 °C for 1 h gave the crude product. Purification by flash column chromatography on silica with 90:10 hexane-CH_2Cl_2

gave pentyl tetrahydrothiophene **7k** (158 mg, 50%) as a colourless oil, R_F (90:10 hexane-CH_2Cl_2) 0.4; IR (ATR) 2926, 2857, 1442, 1260 cm^{-1}; ^{1}H NMR (400 MHz, $CDCl_3$) δ 3.32 (ddd, J = 14.0, 7.5, 6.0 Hz, 1H, SCH), 2.91-2.80 (m, 2H, CH), 2.14-2.01 (m, 2H, CH), 1.93-1.81 (m, 1H, CH), 1.70-1.44 (m, 3H, CH), 1.41-1.23 (m, 6H, CH), 0.88 (t, J = 7.0 Hz, 3H, CH_2*Me*); ^{13}C{^{1}H} NMR (100.6 MHz, $CDCl_3$) δ 49.6 (SCH), 37.9 (CH_2), 37.5 (CH_2), 32.2 (CH_2), 31.9 (CH_2), 30.5 (CH_2), 29.0 (CH_2), 22.7 (CH_2), 14.2 (Me); HRMS (APCI) *m/z* calcd for $C_9H_{18}S$ (M + H)$^+$ 159.1202, found 159.1197 (–2.9 ppm error). Spectroscopic data consistent with those reported in the literature.[5]

Lab Book Reference: NS3-75

2-(Prop-2-en-1-yl)thiolane 7l

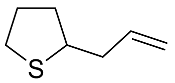

7l

Using general procedure A, tetrahydrothiophene **4** (176 mg, 2.0 mmol, 1.0 eq.), *s*-BuLi (2.0 mL of a 1.3 M solution in 92/8 cyclohexane/hexane, 2.6 mmol, 1.3 eq.) and TMEDA (390 µL, 2.6 mmol, 1.3 eq.) in hexane (10 mL) at 0 °C for 1 h and allyl bromide (346 µL, 4.0 mmol, 2.0 eq.) at 0 °C for 1 h gave the crude product. Purification by flash column chromatography on silica with 90:10 hexane-CH_2Cl_2 as eluent gave allyl tetrahydrothiophene **7l** (124 mg, 48%) as a colourless oil, R_F (90:10 hexane-CH_2Cl_2) 0.2; IR (ATR) 2958, 2925, 1461, 1381, 1259, 916 cm^{-1}; ^{1}H NMR (400 MHz, $CDCl_3$) δ 5.81 (dddd, J = 17.0, 10.5, 7.0, 7.0 Hz, 1H, C*H*=CH_2), 5.12-5.01 (m, 2H, CH=C*H*$_2$), 3.46-3.36 (m, 1H, SCH), 2.95-2.81 (m, 2H, CH), 2.44-2.26 (m, 2H, CH), 2.13-2.01 (m, 2H, CH), 1.97-1.82 (m, 1H, CH), 1.65-1.56 (m, 1H, CH); ^{13}C{^{1}H} NMR (100.6 MHz, $CDCl_3$) δ 136.9 (*C*H=CH_2), 116.4 (CH=*C*H$_2$), 48.4 (SCH), 41.8 (CH_2), 36.9 (CH_2), 32.5 (CH_2), 30.4 (CH_2). Spectroscopic data consistent with those reported in the literature.[6]

Lab Book Reference: NS3-76

2-Benzylthiolane 7m

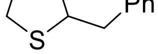

7m

Using general procedure A, tetrahydrothiophene **4** (176 mg, 2.0 mmol, 1.0 eq.), *s*-BuLi (2.0 mL of a 1.3 M solution in 92/8 cyclohexane/hexane, 2.6 mmol, 1.3 eq.) and TMEDA (390 µL, 2.6 mmol, 1.3 eq.) in hexane (10 mL) at 0 °C for 1 h and benzyl bromide (486 µL, 4.0 mmol, 2.0 eq.) at 0 °C for 1 h gave

the crude product. Purification by flash column chromatography on silica with 90:10 hexane-CH_2Cl_2 as eluent gave benzyl tetrahydrothiophene **7m** (137 mg, 38%) as a colourless oil, R_F (90:10 hexane-CH_2Cl_2) 0.3; IR (ATR) 3061, 3026, 2941, 2859, 1495, 1453, 1441, 749, 697 cm^{-1}; ^{1}H NMR (400 MHz, $CDCl_3$) δ 7.35 -7.16 (m, 5H, Ph), 3.67-3.58 (m, 1H, CH), 2.95 (ddd, J = 10.5, 8.0, 6.5 Hz, 1H, CH), 2.91 – 2.83 (m, 3H CH), 2.16-2.07 (m, 1H, CH), 2.07 – 1.99 (m, 1H, CH), 1.96-1.84 (m, 1H, CH), 1.70-1.60 (m, 1H, CH); $^{13}C\{^1H\}$ NMR (100.6 MHz, $CDCl_3$) δ 140.6 (*ipso*-Ph), 129.0 (Ph), 128.5 (Ph), 126.4 (Ph), 50.4 (SCH), 43.8 (CH_2), 36.9 (CH_2), 32.6 (CH_2), 30.2 (CH_2); HRMS (ESI) *m/z* calcd for $C_{11}H_{14}S$ $(M)^+$ 178.8016, found 178.0821 (+0.5 ppm error).

Lab Book Reference: NS3-62

4,4,5,5-Tetramethyl-2-(thiolan-2-yl)-1,3,2-dioxaborolane 7n

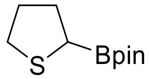

7n

Using general procedure A, tetrahydrothiophene **4** (261 mg, 3.0 mmol, 1.0 eq.), *s*-BuLi (3.0 mL of a 1.3 M solution in 92/8 cyclohexane/hexane, 3.9 mmol, 1.3 eq.) and TMEDA (585 µL, 3.9 mmol, 1.3 eq.) in hexane (15 mL) at 0 °C for 1 h and *i*-PrOBpin (1.11 g, 1.22 mL, 6.0 mmol, 2.0 eq.) at 0 °C for 1 h gave the crude product. Purification by flash column chromatography on silica with 95:5 hexane-Et_2O then 90:10 hexane-Et_2O as eluent gave boronate **7n** (200 mg, 51%) as a colourless oil, R_F (90:10 hexane-Et_2O) 0.4; IR (ATR) 2977, 1407, 1372, 1327, 1215, 1142, 972, 851, 678 cm^{-1}; ^{1}H NMR (400 MHz, $CDCl_3$) δ 2.92-2.78 (m, 2H, SCH), 2.61 (dd, J = 9.0, 6.5 Hz, 1H, SCH), 2.23-2.11 (m, 2H, CH), 1.84-1.74 (m, 2H, CH), 1.26 (s, 12H, Me); $^{13}C\{^1H\}$ NMR (100.6 MHz, $CDCl_3$) δ 83.8 (O*C*Me_2), 33.6 (CH_2), 32.6 (CH_2), 32.5 (CH_2), 24.8 (Me), 24.7 (Me) (SCHB resonance not resolved); HRMS (ESI) *m/z* calcd for $C_{10}H_{19}BO_2S$ $(M + Na)^+$ 237.1093, found 237.1083 (+4.1 ppm error).

Lab Book Reference: NS3-40

Tributyl(thiolan-2-yl)stannane 7o

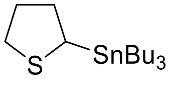

7o

Using general procedure A, tetrahydrothiophene **4** (264 mg, 3.0 mmol, 1.0 eq.), *s*-BuLi (3.0 mL of a 1.3 M solution in 92/8 cyclohexane/hexane, 3.9 mmol, 1.3 eq.) and TMEDA (585 µL, 3.9 mmol, 1.3 eq.)

in hexane (15 mL) at 0 °C for 1 h and Bu_3SnCl (1.95 g, 1.63 mL, 6.0 mmol, 2.0 eq.) at 0 °C for 1 h gave the crude product. Purification by flash column chromatography on silica with 50:50 hexane-CH_2Cl_2 as eluent gave stannane **7o** (949 mg, 84%) as a colourless oil, R_F (hexane) 0.3; IR (ATR) 2954, 2924, 2870, 2853, 1463, 1073, 873, 688, 662, 596, 506 cm^{-1}; ^1H NMR (400 MHz, $CDCl_3$) δ 2.89 (ddd, J = 10.5, 8.0, 2.5 Hz, 1H, SCH), 2.70 (ddd, J = 10.5, 10.0, 7.0 Hz, 1H, SCH), 2.48-2.34 (m, 2H, CH), 2.29-2.15 (m, 1H, CH), 1.67-1.41 (m, 8H, CH), 1.36-1.25 (m, 6H, CH), 1.02-0.78 (m, 15H, CH); $^{13}C\{^1H\}$ NMR (100.6 MHz, $CDCl_3$) δ 37.1 (CH_2), 32.0 (CH_2), 31.5 (CH_2), 29.3 (CH_2), 28.0 (SCH), 27.5 (CH_2), 13.8 (Me), 9.1 (CH_2); HRMS (ESI) *m/z* calcd for $C_{16}H_{34}S^{120}Sn$ $(M(^{120}Sn) + H)^+$ 379.1477, found 379.1469 (+2.1 ppm error).

Lab Book Reference: NS5-91

Phenyl(thian-2-yl)methanone 8a

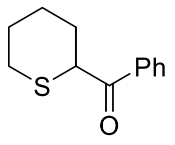

8a

(Scheme 2B, entry 4)

Using general procedure A, tetrahydrothiopyran **5** (101 μL, 1.0 mmol, 1.0 eq.), *s*-BuLi (1.53 mL of a 1.3 M solution in 92/8 cyclohexane/hexane, 2.0 mmol, 2.0 eq.) and TMEDA (300 μL, 2.0 mmol, 2.0 eq.) in hexane (5 mL) at 0 °C for 2 h and *N*-methoxy-*N*-methylbenzamide (456 μL, 3.0 mmol, 3.0 eq.) at 0 °C for 1 h gave the crude product. Purification by flash column chromatography on silica with 67:33 hexane-CH_2Cl_2 as eluent gave ketone **8a** (188 mg, 91%) as a white solid, mp 68-70 °C; R_F (50:50 hexane-CH_2Cl_2) 0.3; R_F (90:10 hexane-CH_2Cl_2) 0.4; IR (ATR) 3059, 2927, 2848, 1678 (C=O), 1596, 1448, 1246, 1222, 1198, 1006, 927, 721, 688 cm^{-1}; ^1H NMR (400 MHz, $CDCl_3$) δ 8.03-7.94 (m, 2H, Ph), 7.61-7.51 (m, 1H, Ph), 7.51-7.41 (m, 2H, Ph), 4.39 (dd, J = 7.0, 3.5 Hz, 1H, SCH), 2.81-2.67 (m, 2H, SCH), 2.19-2.00 (m, 3H, CH), 1.96-1.88 (m, 1H, CH), 1.88 – 1.72 (m, 1H, CH), 1.65-1.48 (m, 1H, CH); $^{13}C\{^1H\}$ NMR (100.6 MHz, $CDCl_3$) δ 197.7 (C=O), 135.6 (*ipso*-Ph), 133.3 (Ph), 128.8 (Ph), 128.7 (Ph), 45.1 (SCH), 29.1 (CH_2), 28.6 (CH_2), 26.8 (CH_2), 24.3 (CH_2); HRMS (ESI) *m/z* calcd for $C_{12}H_{14}OS$ $(M + Na)^+$ 229.0658, found 229.0648 (–4.0 ppm error).

Lab Book Reference: NS10-35

(Scheme 2B, entry 1)

Using general procedure A, tetrahydrothiopyran **5** (101 µL, 1.0 mmol, 1.0 eq.), *s*-BuLi (1.0 mL of a 1.3 M solution in hexane, 1.3 mmol, 1.3 eq.) and TMEDA (195 µL, 1.3 mmol, 1.3 eq.) in hexane (5 mL) at 0 °C for 1 h and *N*-methoxy-*N*-methylbenzamide (304 µL, 2.0 mmol, 2.0 eq.) at 0 °C for 1 h gave the crude product. Purification by flash column chromatography on silica with 67:33 hexane-CH_2Cl_2 as eluent gave ketone **8a** (92 mg, 45%) as a white solid.

Lab Book Reference: NS10-33

(Scheme 2B, entry 2)

Using general procedure A, tetrahydrothiopyran **5** (101 µL, 1.0 mmol, 1.0 eq.), *s*-BuLi (1.0 mL of a 1.3 M solution in hexane, 1.3 mmol, 1.3 eq.) and TMEDA (195 µL, 1.3 mmol, 1.3 eq.) in hexane (5 mL) at 0 °C for 2 h and *N*-methoxy-*N*-methylbenzamide (304 µL, 2.0 mmol, 2.0 eq.) at 0 °C for 1 h gave the crude product. Purification by flash column chromatography on silica with 85:15 hexane-CH_2Cl_2 and then 67:33 hexane-CH_2Cl_2 as eluent gave ketone **8a** (120 mg, 58%) as a white solid.

Lab Book Reference: NS3-14

(Scheme 2B, entry 3)

Using general procedure A, tetrahydrothiopyran **5** (101 µL, 1.0 mmol, 1.0 eq.), *s*-BuLi (1.53 mL of a 1.3 M solution in hexane, 2.0 mmol, 2.0 eq.) and TMEDA (300 µL, 2.0 mmol, 2.0 eq.) in hexane (5 mL) at 0 °C for 1 h and *N*-methoxy-*N*-methylbenzamide (456 µL, 3.0 mmol, 3.0 eq.) at 0 °C for 1 h gave the crude product. Purification by flash column chromatography on silica with 67:33 hexane-CH_2Cl_2 as eluent gave ketone **8a** (175 mg, 85%) as a white solid.

Lab Book Reference: NS10-34

(Scheme 2B, entry 5)

Using general procedure A, tetrahydrothiopyran **5** (101 µL, 1.0 mmol, 1.0 eq.), *s*-BuLi (1.53 mL of a 1.3 M solution in hexane, 2.0 mmol, 2.0 eq.) and TMEDA (300 µL, 2.0 mmol, 2.0 eq.) in hexane (5 mL) at –10 °C for 2 h and *N*-methoxy-*N*-methylbenzamide (456 µL, 3.0 mmol, 3.0 eq.) at 0 °C for 1 h gave the crude product. Purification by flash column chromatography on silica with 67:33 hexane-CH_2Cl_2 as eluent gave ketone **8a** (188 mg, 61%) as a white solid.

Lab Book Reference: NS10-36

Phenyl(thian-2-yl)methanol 8b

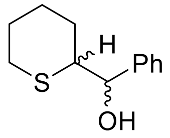

8b

Using general procedure A, tetrahydrothiopyran **5** (101 μL, 1.0 mmol, 1.0 eq.), *s*-BuLi (1.0 mL of a 1.3 M solution in 92/8 cyclohexane/hexane, 1.3 mmol, 1.3 eq.) and TMEDA (195 μL, 1.3 mmol, 1.3 eq.) in hexane (5 mL) at 0 °C for 2 h and benzaldehyde (210 μL, 2.0 mmol, 2.0 eq.) at 0 °C for 1 h gave the crude product which contained a 55:45 mixture (by ^{1}H NMR spectroscopy) of alcohols **8b**. Purification by flash column chromatography on silica with 85:15 hexane-CH_2Cl_2 then 55:45 hexane-CH_2Cl_2 then 300:9:1 CH_2Cl_2-MeOH-$NH_4OH_{(aq)}$ as eluent gave a 50:50 mixture of alcohols **8b** (129 mg, 62%) as a colourless oil, R_F (200:9:1 CH_2Cl_2-MeOH-$NH_4OH_{(aq)}$) 0.2; IR (ATR) 3409 (OH), 3061, 3029, 2925, 2848, 1492, 1452, 1439, 1257, 1062, 1021, 761, 699, 611 cm^{-1}; ^{1}H NMR (400 MHz, $CDCl_3$) δ 7.46-7.18 (m, 5H, Ph), 4.80-4.56 (m, 1H, HOC*H*), 3.08 (ddd, J = 8.5, 5.5, 2.5 Hz, 0.5H, SCH), 2.95 (ddd, J = 8.5, 8.5, 2.5 Hz, 0.5H, SCH), 2.91 (s, 0.5H, OH), 2.78 (ddd, J = 10.5, 7.5, 2.5 Hz, 0.5H, CH), 2.73-2.68 (m, 0.5H, CH), 2.67-2.59 (m, 0.5H, CH), 2.59-2.52 (m, 0.5H, CH), 2.32–2.18 (m, 0.5H, OH), 2.04 (ddd, J = 13.5, 3.5, 3.5 Hz, 0.5H, CH), 1.95-1.66 (m, 3H, CH), 1.64-1.21 (m, 2.5H, CH); $^{13}C\{^1H\}$ NMR (100.6 MHz, $CDCl_3$) δ 141.6 (*ipso*-Ph), 141.4 (*ipso*-Ph), 128.6 (Ph), 128.4 (Ph), 128.2 (Ph), 128.0 (Ph), 126.9 (Ph), 126.5 (Ph), 76.7 (OCH), 75.2 (OCH), 49.7 (SCH), 49.4 (SCH), 29.4 (CH_2), 28.5 (CH_2), 27.1 (CH_2), 27.0 (CH_2), 26.9 (CH_2), 25.9 (CH_2), 24.0 (CH_2) (one CH_2 resonance not resolved); HRMS (ESI) *m/z* calcd for $C_{12}H_{16}OS$ $(M + Na)^+$ 231.0814, found 231.0814 (–0.2 ppm error).
Lab Book Reference: NS3-9

(Pyridin-3-yl)(thian-2-yl)methanol 8c

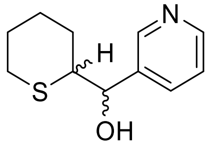

8c

Using general procedure A, tetrahydrothiopyran **5** (101 μL, 1.0 mmol, 1.0 eq.), *s*-BuLi (1.0 mL of a 1.3 M solution in 92/8 cyclohexane/hexane, 1.3 mmol, 1.3 eq.) and TMEDA (195 μL, 1.3 mmol, 1.3 eq.) in hexane (5 mL) at 0 °C for 2 h and nicotine aldehyde (200 μL, 2.0 mmol, 2.0 eq.) at 0 °C for 1 h gave the crude product which contained a 60:40 mixture (by ^{1}H NMR spectroscopy) of alcohols **8c**. Purification by flash column chromatography on silica with 500:9:1 CH_2Cl_2-MeOH-$NH_4OH_{(aq)}$ as eluent

gave a 70:30 mixture of alcohols **8c** (105 mg, 52%) as a colourless oil, R_F (500:9:1 CH_2Cl_2-MeOH-$NH_4OH_{(aq)}$) 0.2; IR (ATR) 3340 (OH), 2970, 2930, 1966, 1466, 1427, 1379, 1161, 1128, 950, 816, 713, 628 cm^{-1}; ^{1}H NMR (400 MHz, $CDCl_3$) δ 8.54-8.44 (m, 2H, Ar), 7.78-7.73 (m, 1H, Ar), 7.34-7.27 (m, 1H, Ar), 4.74-4.70 (m, 1H, OCH), 4.14-3.74 (m, 1H, OH), 3.07 (ddd, J = 11.0, 6.0, 2.5 Hz, 0.7H, SCH), 2.96 (ddd, J = 7.5, 7.5, 2.5 Hz, 0.3H, SCH), 2.86-2.54 (m, 2H, CH), 2.13-2.04 (m, 0.7H, CH), 1.98-1.84 (m, 1.7H, CH), 1.83-1.67 (m, 1H, CH), 1.66-1.25 (m, 2.6H, CH); ^{13}C{^{1}H} NMR (100.6 MHz, $CDCl_3$) δ 149.2 (Ar), 148.8 (Ar), 148.5 (Ar), 148.2 (Ar), 137.6 (*ipso*-Ar), 137.4 (*ipso*-Ar), 134.6 (Ar), 134.5 (Ar), 123.6 (Ar), 123.4 (Ar), 74.3 (OCH), 73.0 (OCH), 49.6 (SCH), 49.1 (SCH), 29.36 (CH_2), 29.32 (CH_2), 28.7 (CH_2), 27.1 (CH_2), 27.04 (CH_2), 27.02 (CH_2), 25.7 (CH_2), 24.1 (CH_2); HRMS (ESI) *m/z* calcd for $C_{11}H_{16}NOS$ (M + H)$^+$ 210.0947, found 210.0943 (+2.2 ppm error).
Lab Book Reference: NS3-54

N-tert-Butylthiane-2-carboxamide 8d

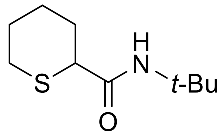

8d

Using general procedure A, tetrahydrothiopyran **5** (101 µL, 1.0 mmol, 1.0 eq.), *s*-BuLi (1.53 mL of a 1.3 M solution in 92/8 cyclohexane/hexane, 2.0 mmol, 2.0 eq.) and TMEDA (300 µL, 2.0 mmol, 2.0 eq.) in hexane (5 mL) at 0 °C for 2 h and *tert*-butyl isocyanate (343 µL, 3.0 mmol, 3.0 eq.) gave the crude product. Purification by flash column chromatography on silica with 95:5 hexane-Et_2O as eluent gave amide **8d** (179 mg, 89%) as a white solid, mp 106-108 °C, R_F (90:10 hexane-Et_2O) 0.3; IR (ATR) 3289 (NH), 2966, 2927, 1643 (CO), 1549, 1508, 1455, 1363, 1223, 917, 735, 655 cm^{-1}; ^{1}H NMR (400 MHz, $CDCl_3$) 85:15 mixture of rotamers δ 7.10 (br s, 0.15H, NH), 6.79 (s, 0.85H, NH), 3.25 (dd, J = 6.5, 4.0 Hz, 0.85H, CH), 2.64 (ddd, J = 12.5, 8.5, 3.5 Hz, 0.85H, CH), 2.58-2.48 (m, 1.15H, CH), 2.34-2.21 (m, 1.15H, CH), 2.00 – 1.89 (m, 0.85H, CH), 1.86-1.68 (m, 2.15H, CH), 1.65-1.49 (m, 2.0H, CH), 1.36 (s, 7.65H, CMe_3), 1.32 (s, 1.35H, CMe_3); ^{13}C{^{1}H} NMR (100.6 MHz, $CDCl_3$) rotamers δ 169.8 (C=O), 168.7 (C=O), 63.3 (SCH), 51.6 (*C*Me), 51.3 (*C*Me), 44.7 (SCH), 35.5 (CH_2), 29.1 (CH_2), 28.7 (C*Me*$_3$), 28.5 (C*Me*$_3$), 28.2 (CH_2), 28.0 (CH_2), 26.7 (CH_2), 25.8 (CH_2), 23.9 (CH_2), 23.6 (CH_2); HRMS (ESI) *m/z* calcd for $C_{10}H_{19}NOS$ (M + Na)$^+$ 224.1080, found 224.1079 (+0.3 ppm error).
Lab Book Reference: NS10-39

tert-Butyl[phenyl(thian-2-yl)methyl]amine 8e

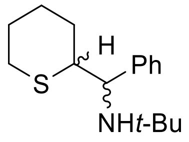

8e

Using general procedure M, tetrahydrothiopyran **5** (101 μL, 1.0 mmol, 1.0 eq.), *s*-BuLi (1.53 mL of a 1.3 M solution in 92/8 cyclohexane/hexane, 2.0 mmol, 2.0 eq.) and TMEDA (300 μL, 2.0 mmol, 2.0 eq.) in hexane (5 mL) at 0 °C for 2 h and *tert*-butyl(phenylmethylidene)amine (534 μL, 3.0 mmol, 3.0 eq.) gave the crude product. Purification by flash column chromatography on silica with 90:10 hexane-Et_2O then 80:20 hexane-CH_2Cl_2 as eluent gave a single unidentified diastereomeric amine **8e** (187 mg, 71%) as a colourless oil, R_F (80:20 hexane-Et_2O) 0.5; IR (ATR) 2960, 2928, 1451, 1364, 1225, 1107, 1027, 756, 703, 592, 496 cm^{-1}; ^{1}H NMR (400 MHz, $CDCl_3$) δ 7.40-7.37 (m, 2H, Ph), 7.33-7.26 (m, 2H, Ph), 7.24-7.14 (m, 1H, Ph), 3.83 (d, J = 5.5 Hz, 1H, NCH), 2.89 (ddd, J = 11.5, 5.5, 2.5 Hz, 1H, CH), 2.66 (dddd, J = 13.5, 12.0, 3.0, 1.0 Hz, 1H, CH), 2.61-2.53 (m, 1H, CH), 2.02 (dddd, J = 13.5, 3.5, 3.5, 3.0 Hz, 1H, CH), 1.93-1.79 (m, 2H, CH), 1.58 – 1.24 (m, 3H, CH, NH), 1.27-1.10 (m, 1H, CH), 0.97 (s, 9H, CMe_3); $^{13}C\{^1H\}$ NMR (100.6 MHz, $CDCl_3$) δ 145.4 (*ipso*-Ph), 127.88 (Ph), 127.86 (Ph), 126.8 (Ph), 60.9 (NCH), 51.3 (*C*Me), 50.8 (SCH), 30.2 (C*Me*$_3$), 29.9 (CH_2), 29.5 (CH_2), 27.3 (CH_2), 26.8 (CH_2); HRMS (ESI) *m/z* calcd for $C_{16}H_{25}NS$ $(M + H)^+$ 264.1780, found 264.1778 (+1.0 ppm error).
Lab Book Reference: NS10-42

Thiane-2-carboxylic acid 8f

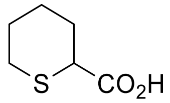

8f

Using general procedure B, tetrahydrothiopyran **5** (500 mg, 5.0 mmol, 2.5 eq.), *s*-BuLi (1.54 mL of a 1.3 M solution in 92/8 cyclohexane/hexane, 2.0 mmol, 1.0 eq.) and TMEDA (975 μL, 5.0 mmol, 2.5 eq.) in hexane (10 mL) at 0 °C for 2 h and CO_2 at −78 °C for 1 h gave the crude product. Purification by flash column chromatography on silica with 99:1 CH_2Cl_2-acetone as eluent gave acid **8f** (102 mg, 39%) as a colourless oil, IR (ATR) 2929, 2850, 1698 (C=O), 1439, 1419, 1250, 1190, 931, 897, 841, 693 cm^{-1}; ^{1}H NMR (400 MHz, $CDCl_3$) δ 10.93 (br s, 1H, CO_2H), 3.52 (dd, J = 8.0, 3.5 Hz, 1H, SCH), 2.79 (ddd, J = 13.5, 7.5, 3.5 Hz, 1H, SCH), 2.57 (ddd, J = 13.5, 8.0, 3.0 Hz, 1H, SCH), 2.11 (dddd, J = 13.5, 7.5, 3.5, 3.5 Hz, 1H, CH), 1.98 (dddd, J = 13.5, 9.0, 7.5, 3.0 Hz, 1H, CH), 1.93-1.69 (m, 3H, CH), 1.56-1.43 (m,

1H, CH); $^{13}C\{^1H\}$ NMR (100.6 MHz, $CDCl_3$) δ 178.5 (C=O), 42.9 (SCH), 29.4 (CH_2), 27.9 (CH_2), 26.5 (CH_2), 23.8 (CH_2); HRMS (ESI) *m/z* calcd for $C_6H_{10}O_2S$ $(M + Na)^+$ 169.0294, found 169.0297 (−1.7 ppm error).

Lab Book Reference: NS5-22

2-Phenylthiane 8g

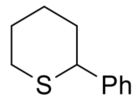

8g

s-BuLi (0.5 mL of a 1.3 M solution in 92/8 cyclohexane/hexane, 0.65 mmol, 1.3 eq.) was added dropwise to a stirred solution of tetrahydrothiopyran **5** (50 µL, 0.50 mmol, 1.0 eq.), and TMEDA (150 µL, 1.0 mmol, 2.0 eq.) in hexane (2.5 mL) at 0 °C under Ar. The resulting solution was stirred at 0 °C for 2 h. Then, the solution was cooled to −78 °C and a freshly prepared solution of CuCN·2LiCl in THF (prepared from CuCN (107 mg, 1.2 mmol) and LiCl (102 mg, 2.4 mmol) in THF (2.4 mL)) was added. The resulting solution was allowed to warm to −35 °C and stirred at −35 °C for 15 min. Then, Ph_2IPF_6 (511 mg, 1.2 mmol, 2.4 eq.) was added in one portion and the resulting solution was stirred at −35 °C for 15 min and then allowed to warm to rt over 4 h. Saturated $NH_4OH_{(aq)}$ (20 mL) was added and the two layers were separated. The aqueous layer was extracted with Et_2O (3 × 20 mL). The combined organic layers were dried ($MgSO_4$) and evaporated under reduced pressure to give the crude product. Purification by flash column chromatography on silica with 95:5 hexane-CH_2Cl_2 as eluent gave α-phenyl sulfide **8g** (45 mg, 50%) as a colourless oil, R_F (90:10 hexane-CH_2Cl_2) 0.2; IR (ATR) 3028, 2926, 2847, 1491, 1451, 1439, 1260, 886, 756, 697, 607, 517 cm^{-1}; ^1H NMR (400 MHz, $CDCl_3$) δ 7.37-7.27 (m, 4H, Ph), 7.24-7.20 (m, 1H, Ph), 3.84 (dd, J = 11.5, 2.5 Hz, 1H, SCH), 2.87 (ddd, J = 13.5, 12.5, 2.5 Hz, 1H, SCH), 2.66 (ddd, J = 13.5, 3.5, 2.5 Hz, 1H, SCH), 2.19-2.07 (m, 1H, CH), 2.04-1.88 (m, 3H, CH), 1.73-1.61 (m, 1H, CH), 1.52-1.41 (m, 1H, CH).; $^{13}C\{^1H\}$ NMR (100.6 MHz, $CDCl_3$) δ 143.1 (*ipso*-Ph), 128.7 (Ph), 127.6 (Ph), 127.4 (Ph), 47.6 (SCH), 35.3 (CH_2), 31.0 (CH_2), 27.2 (CH_2), 26.9 (CH_2); HRMS (ESI) *m/z* calcd for $C_{11}H_{14}$ $(M + H)^+$ 179.0889, found 179.0888 (−0.7 ppm error). Spectroscopic data consistent with those reported in the literature.[7]

Lab Book Reference: NS10-49

2-Pentylthiane 8h

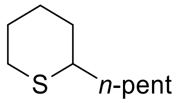

8h

Using general procedure A, tetrahydrothiopyran **5** (101 µL, 1.0 mmol, 1.0 eq.), *s*-BuLi (1.53 mL of a 1.3 M solution in 92/8 cyclohexane/hexane, 2.0 mmol, 2.0 eq.) and TMEDA (300 µL, 2.0 mmol, 2.0 eq.) in hexane (5 mL) at 0 °C for 2 h and *n*-pentyl iodide (392 µL, 3.0 mmol, 3.0 eq.) gave the crude product. Purification by flash column chromatography on silica with 90:10 hexane-CH_2Cl_2 then as eluent gave pentyl tetrahydrothiopyran **8h** (110 mg, 64%) as a colourless oil, R_F (hexane) 0.2; IR (ATR) 2955, 2924, 2854, 1454, 1424, 1439, 1274, 1259, 726 cm^{-1}; ^1H NMR (400 MHz, $CDCl_3$) δ 2.73-2.53 (m, 3H, SCH, CH), 2.02-1.79 (m, 3H, CH), 1.63-1.20 (m, 11H, CH), 0.88 (t, J = 7.0 Hz, 3H, CH_2Me); $^{13}C\{^1H\}$ NMR (100.6 MHz, $CDCl_3$) δ 42.9 (SCH), 36.4 (CH_2), 35.0 (CH_2), 31.9 (CH_2), 29.3 (CH_2), 27.6 (CH_2), 26.6 (CH_2), 26.4 (CH_2), 22.7 (CH_2), 14.2 (Me); HRMS (APCI) *m/z* calcd for $C_{10}H_{21}S$ $(M + H)^+$ 173.1359, found 173.1354 (+2.7 ppm error).

Lab Book Reference: NS10-44

2-(Prop-2-en-1-yl)thiane 8i

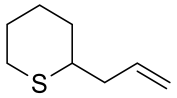

8i

Using general procedure A, tetrahydrothiopyran **5** (101 µL, 1.0 mmol, 1.0 eq.), *s*-BuLi (1.53 mL of a 1.3 M solution in 92/8 cyclohexane/hexane, 2.0 mmol, 2.0 eq.) and TMEDA (300 µL, 2.0 mmol, 2.0 eq.) in hexane (5 mL) at 0 °C for 2 h and allyl bromide (259 µL, 3.0 mmol, 3.0 eq.) gave the crude product. Purification by flash column chromatography on silica with hexane then 95:5 to 85:15 hexane-CH_2Cl_2 as eluent gave allyl tetrahydrothiopyran **8i** (87 mg, 61%) as a colourless oil, R_F (85:15 hexane-CH_2Cl_2) 0.3; R_F (85:15 hexane- CH_2Cl_2) 0.3; IR (ATR) 3076, 2977, 2924, 2846, 1438, 1272, 991, 912, 805, 682, 653, 619 cm^{-1}; ^1H NMR (400 MHz, $CDCl_3$) δ 5.90-5.73 (m, 1H, CH=CH$_2$), 5.16-4.97 (m, 2H, CH=CH_2), 2.80-2.55 (m, 3H, CH, SCH), 2.26-2.20 (m, 2H, CH), 2.04-1.81 (m, 3H, CH), 1.64-1.50 (m, 1H, CH), 1.46-1.28 (m, 2H, CH); $^{13}C\{^1H\}$ NMR (100.6 MHz, $CDCl_3$) δ 135.6 (CH=CH$_2$), 116.9 (CH=CH$_2$), 42.3 (SCH), 40.7 (CH_2), 34.3 (CH_2), 29.4 (CH_2), 27.3 (CH_2), 26.3 (CH_2). Attempted characterisation by MS was unsuccessful.

Lab Book Reference: NS10-40

2-Benzylthiane 8j

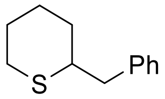

8j

Using general procedure A, tetrahydrothiopyran **5** (101 µL, 1.0 mmol, 1.0 eq.), *s*-BuLi (1.0 mL of a 1.3 M solution in 92/8 cyclohexane/hexane, 1.3 mmol, 1.3 eq.) and TMEDA (195 µL, 1.3 mmol, 1.3 eq.) in hexane (5 mL) at 0 °C for 2 h and benzyl bromide (243 µL, 2.0 mmol, 2.0 eq.) at 0 °C for 1 h gave the crude product. Purification by flash column chromatography on silica with hexane then 95:5 hexane-CH_2Cl_2 as eluent gave benzyl tetrahydrothiopyran **8j** (78 mg, 41%) as a colourless oil, R_F (95:5 hexane-CH_2Cl_2) 0.8; IR (ATR) 3026, 2924, 2849, 1495, 1438, 1453, 1258, 1046, 750, 700, 541 cm^{-1}; ^{1}H NMR (400 MHz, $CDCl_3$) δ 7.33-7.27 (m, 2H, Ph), 7.25-7.18 (m, 3H, Ph), 2.94 (dddd, J = 10.5, 8.0, 7.0, 2.5 Hz, 1H, SCH), 2.80 (dd, J = 13.5, 7.0 Hz, 1H CH), 2.73 (dd, J = 13.5, 8.0 Hz, 1H, CH), 2.67 (ddd, J = 13.5, 11.5, 3.0 Hz, 1H, SCH), 2.59 (dddd, J = 13.5, 4.0, 4.0, 1.5 Hz, 1H, SCH), 2.01-1.89 (m, 2H, CH), 1.89 – 1.80 (m, 1H, CH), 1.65-1.53 (m, 1H, CH), 1.43 (dddd, J = 12.5, 12.5, 10.5, 3.0 Hz, 1H, CH), 1.29 (ddddd, J = 13.5, 12.5, 12.5, 3.0, 3.0 Hz, 1H, CH); ^{13}C{^{1}H} NMR (100.6 MHz, $CDCl_3$) δ 139.2 (*ipso*-Ph), 129.3 (Ph), 128.4 (Ph), 126.5 (Ph), 44.1 (SCH), 42.7 (CH_2), 34.1 (CH_2), 29.5 (CH_2), 27.3 (CH_2), 26.2 (CH_2); HRMS (ESI) *m/z* calcd for $C_{12}H_{17}S$ $(M + OH)^+$ 209.0995, found 209.0998 (–1.8 ppm error). Lab Book Reference: NS3-56

4,4,5,5-Tetramethyl-2-(tetrahydro-2*H*-thiopyran-2-yl)-1,3,2-dioxaborolane 8k

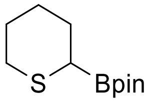

8k

Using general procedure A, tetrahydrothiopyran **5** (101 µL, 1.0 mmol, 1.0 eq.), *s*-BuLi (1.53 mL of a 1.3 M solution in 92/8 cyclohexane/hexane, 2.0 mmol, 2.0 eq.) and TMEDA (300 µL, 2.0 mmol, 2.0 eq.) in hexane (5 mL) at 0 °C for 2 h and boronic ester (612 µL, 3.0 mmol, 3.0 eq.) gave the crude product. Purification by flash column chromatography on silica with 95:5 hexane-Et_2O then 90:10 hexane-Et_2O as eluent gave boronic ester **8k** (146 mg, 64%) as a colourless oil, R_F (90:10 hexane-Et_2O) 0.3; IR (ATR) 2977, 2924, 1367, 1324, 1250, 1135, 973, 875, 846 cm^{-1}; ^{1}H NMR (400 MHz, $CDCl_3$) δ 2.68-2.52 (m, 2H, SCH), 2.38 (dd, J = 10.5, 2.5 Hz, 1H, SCH), 2.02-1.93 (m, 1H, CH), 1.93-1.83 (m, 1H, CH), 1.83-1.65 (m, 3H, CH), 1.44-1.31 (m, 1H, CH), 1.25 (s, 12H, Me); ^{13}C{^{1}H} NMR (100.6 MHz, $CDCl_3$) δ 83.9

(CO), 29.4 (CH_2), 29.1 (CH_2), 27.5 (CH_2), 26.6 (CH_2), 24.8 (Me) (SCHB resonance not resolved); HRMS (ESI) *m/z* calcd for $C_{11}H_{21}BO_2S$ $(M + H)^+$ 229.1430, found 229.1427 (+1.6 ppm error).
Lab Book Reference: NS10-43

Tributyl(thian-2-yl)stannane 8l

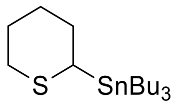

8l

Using general procedure A, tetrahydrothiopyran **5** (101 µL, 102 mg, 1.0 mmol, 1.0 eq.), *s*-BuLi (1.53 mL of a 1.3 M solution in 92/8 cyclohexane/hexane, 2.0 mmol, 2.0 eq.) and TMEDA (300 µL, 2.0 mmol, 2.0 eq.) in hexane (5 mL) at 0 °C for 2 h and Bu_3SnCl (814 µL, 977 mg, 3.0 mmol, 3.0 eq.) gave the crude product. Purification by flash column chromatography on silica with hexane then 90:10 hexane-CH_2Cl_2 as eluent gave stannane **8l** (340 mg, 87%) as a colourless oil, R_F (hexane) 0.3; IR (ATR) 2955, 2846, 2919, 1463, 1376, 1264, 1072, 1034, 875, 689, 665, 596, 504 cm^{-1}; ^1H NMR (400 MHz, $CDCl_3$) δ 2.67 (ddd, J = 13.0, 12.0, 3.0 Hz, 1H, SCH), 2.58 (dd, J = 12.0, 2.5 Hz, 1H, SCH), 2.41 (ddd, J = 13.0, 3.5 Hz, 1H, SCH), 2.05-1.90 (m, 2H, CH), 1.85-1.69 (m, 3H, CH), 1.56-1.41 (m, 7H, CH), 1.35-1.25 (m, 6H, CH), 0.98-0.79 (m, 15H, CH); $^{13}C\{^1H\}$ NMR (100.6 MHz, $CDCl_3$) δ 32.7 (CH_2), 31.8 (SCH_2), 29.3 (CH_2), 28.8 (CH_2), 28.2 (CH_2), 27.6 (CH_2), 26.1 (SCH), 13.8 (Me), 8.6 (CH_2); HRMS (ESI) *m/z* calcd for $C_{17}H_{36}S^{120}Sn$ $(^{120}M + H)^+$ 393.1634, found 393.1654 (+4.0 ppm error).
Lab Book Reference: NS10-41

4-Methylthiomorpholine 6

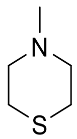

6

$NaBH(OAc)_3$ (9.23 g, 44 mmol, 1.5 eq.) was added to a stirred solution of thiomorpholine (3.00 g, 29 mmol, 1.0 eq.), 37% aqueous formaldehyde (23.6 mL, 29 mmol, 10.0 eq.) and MgSO4 (9.75 g) in 4:1 CH_2Cl_2-AcOH (142 mL) at rt under Ar. The resulting mixture was stirred at rt for 14 h. Then, 37% $NH_4OH_{(aq)}$ (150 mL) was added and the two layers were separated. The aqueous layer was extracted with CH_2Cl_2 (3 × 200 mL). The combined organic layers were dried ($MgSO_4$) and evaporated under reduced pressure (care, product is volatile) to give the crude product. Purification by flash column

chromatography on silica with 300:9:1 CH_2Cl_2-MeOH-$NH_4OH_{(aq)}$ as eluent gave *N*-methyl thiomorpholine **6** (2.36 g, 69%) as a colourless oil, R_F (90:9:1 CH_2Cl_2-MeOH-$NH_4OH_{(aq)}$) 0.7; IR (ATR) 2911, 2841, 2790, 2678, 1456, 1285, 1132, 1120, 1069, 1000, 956, 763, 667 cm^{-1}; ^{1}H NMR (400 MHz, $CDCl_3$) δ 2.72-2.53 (m, 8H, CH_2), 2.25 (s, 3H, NMe); $^{13}C\{^1H\}$ NMR (100.6 MHz, $CDCl_3$) δ 56.7 (NCH_2), 47.4 (NMe), 28.1 (SCH_2); HRMS (ESI) *m/z* calcd for $C_5H_{11}NS$ $(M + H)^+$ 118.0682, found 118.0685 (+2.5 ppm error).
Lab Book Reference: NS2-71

(4-Methylthiomorpholin-2-yl)(phenyl)methanol 9a

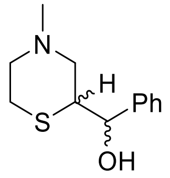

9a

(Scheme 2C, entry 6)

Using general procedure A, *N*-methyl thiomorpholine **6** (118 mg, 1.0 mmol, 1.0 eq.), *s*-BuLi (1.0 mL of a 1.3 M solution in 92/8 cyclohexane/hexane, 1.3 mmol, 1.3 eq.) and TMEDA (45 µL, 0.3 mmol, 0.3 eq.) in hexane (5.0 mL) at −10 °C for 3 h and benzaldehyde (210 µL, 2.0 mmol, 2.0 eq.) at −10 °C for 30 min gave the crude product which contained a 55:45 mixture (by ^{1}H NMR spectroscopy) of alcohols **9a**. Purification by flash column chromatography on silica with CH_2Cl_2-acetone (45:5 → 40:10 → 30:20 → 25:25 → 10:40 → 0:50) as eluent gave alcohol **9aa** (25.3 mg, 11%) as a colourless solid, mp 104-106 °C; R_F (1:1 CH_2Cl_2-acetone) 0.28; IR (ATR) 3380 (OH), 2921, 2800, 1453, 1290, 1064, 1010, 907, 767, 727, 699, 602 cm^{-1}; ^{1}H NMR (400 MHz, $CDCl_3$) δ 7.42-7.26 (m, 5H, Ph), 5.06 (d, J = 6.5 Hz, 1H, HOC*H*), 4.16 (br s, 1H, OH), 3.16(ddd, J = 13.5, 10.0, 3.0 Hz, 1H, CH), 2.89-2.81 (m, 2H, CH), 2.74 (dd, J = 12.0, 4.5 Hz, 1H, CH), 2.59 (dd, J = 12.0, 3.0 Hz, 1H, CH), 2.52-2.41 (m, 2H, CH), 2.21 (s, 3H, NMe); $^{13}C\{^1H\}$ NMR (100.6 MHz, $CDCl_3$) δ 141.9 (*ipso*-Ph), 128.3 (Ph), 127.3 (Ph), 126.7 (Ph), 76.2 (HOCH), 59.7 (SCH_2), 56.3 (NCH_2), 47.2 (NMe), 47.1 (SCH), 25.9 (NCH_2); HRMS (ESI) *m/z* calcd for $C_{12}H_{17}NOS$ $(M + H)^+$ 224.1104, found 224.1106 (–1.0 ppm error), a mixture of alcohols **9aa** and **9ab** (97.1 mg, 43%) as a colourless solid and alcohol **9ab** (73.5 mg, 33%) as a colourless solid, R_F (1:1 CH_2Cl_2-acetone) 0.28; mp 107-108 °C, IR (ATR) 3380 (OH), 2916, 2801, 1454, 1288, 1053, 977, 910, 763, 729, 699, 591 cm^{-1}; ^{1}H NMR (400 MHz, $CDCl_3$) δ 7.39-7.25 (m, 5H, Ph), 5.08 (d, J = 4.0 Hz, 1H, HOC*H*), 4.73 (br s, 1H, OH), 3.30-3.18 (m, 1H, CH), 2.99-2.90 (m, 1H, CH), 2.82-2.71 (m, 2H, CH), 2.70-2.59 (m, 2H, CH), 2.54 (ddd, J = 12.0, 9.0, 3.0 Hz, 1H, CH), 2.23 (s, 3H, NMe); $^{13}C\{^1H\}$ NMR

(100.6 MHz, $CDCl_3$) δ 143.6 (*ipso*-Ph), 128.5 (Ph), 127.6 (Ph), 125.9 (Ph), 80.0 (HOCH), 57.2 (SCH_2), 56.2 (NCH_2), 47.1 (NMe), 44.4 (SCH), 27.0 (NCH_2); HRMS (ESI) *m/z* calcd for $C_{12}H_{17}NOS$ $(M + H)^+$ 224.1104, found 224.1106 (–1.0 ppm error). Total yield of alcohols **9a** 195.9 mg (87%). The relative stereochemistry of **9aa** and **9ab** is unknown.

(Scheme 2C, entry 1)

Using general procedure A, *N*-methyl thiomorpholine **6** (234 mg, 2.0 mmol, 1.0 eq.) *s*-BuLi (2.0 mL of a 1.3 M solution in cyclohexane, 2.6 mmol, 1.3 eq.), TMEDA (389 µL, 2.6 mmol, 1.3 eq.) at 0 °C for 15 min and benzaldehyde (406 µL, 4.0 mmol, 2.0 eq.) in hexane (10 mL) gave the crude product which contained a 50:50 mixture (by ^{1}H NMR spectroscopy) of alcohols **9a**. Purification by flash column chromatography on silica with 200:9:1 CH_2Cl_2-MeOH-$NH_4OH_{(aq)}$ as eluent gave a 50:50 mixture of alcohols **9a** (141 mg, 32%) as a light yellow solid. From the solid product, crystallisation occurred to give crystals that were suitable for analysis by X-ray crystallography (CCDC 2294608). This confirmed the regioselectivity of the lithiation.

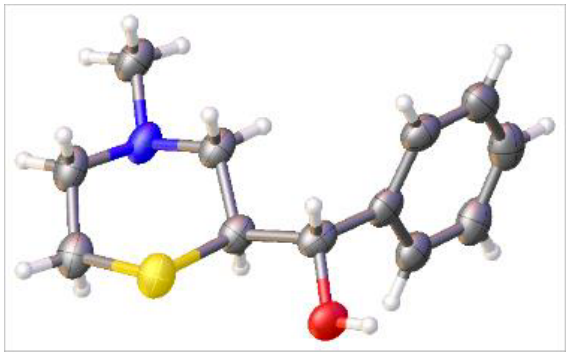

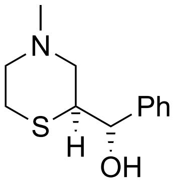

syn-**9a**

Lab Book Reference: MA 5-63

(Scheme 2C, entry 2)

Using general procedure A, *N*-methyl thiomorpholine **6** (234 mg, 2.0 mmol, 1.0 eq.) *s*-BuLi (2.0 mL of a 1.3 M solution in cyclohexane, 2.6 mmol, 1.3 eq.), TMEDA (389 µL, 2.6 mmol, 1.3 eq.) at 0 °C for 1 h and benzaldehyde (406 µL, 4.0 mmol, 2.0 eq.) in hexane (10 mL) gave the crude product which contained a 50:50 mixture (by ^{1}H NMR spectroscopy) of alcohols **9a**. Purification by flash column chromatography on silica with 200:9:1 CH_2Cl_2-MeOH-$NH_4OH_{(aq)}$ as eluent gave a 50:50 mixture of alcohols **9a** (73 mg, 16%) as a light yellow solid.

Lab Book Reference: MA 5-57

(Scheme 2C, entry 3)

Using general procedure A, *N*-methyl thiomorpholine **6** (234 mg, 2.0 mmol, 1.0 eq.) *s*-BuLi (1.84 mL of a 1.4 M solution in cyclohexane, 2.6 mmol, 1.3 eq.), TMEDA (389 μL, 2.6 mmol, 1.3 eq.) at –10 °C for 1 h and benzaldehyde (406 μL, 4.0 mmol, 2.0 eq.) in hexane (10 mL) gave the crude product which contained a 50:50 mixture (by ^{1}H NMR spectroscopy) of alcohols **9a**. Purification by flash column chromatography on silica with 200:9:1 CH_2Cl_2-MeOH-$NH_4OH_{(aq)}$ as eluent gave a 50:50 mixture of alcohols **9a** (366 mg, 82%) as a light yellow solid and recovered *N*-methyl thiomorpholine **6** (23 mg, 10%) as a colourless oil.

Lab Book Reference: MA 6-53

(Scheme 2C, entry 4)

Using general procedure A, *N*-methyl thiomorpholine **6** (117 mg, 1.0 mmol, 1.0 eq.) *s*-BuLi (0.94 mL of a 1.4 M solution in 92/8 cyclohexane/hexane, 1.3 mmol, 1.3 eq.), TMEDA (195 μL, 1.3 mmol, 1.3 eq.) at −10 °C for 2 h and benzaldehyde (203 μL, 2.0 mmol, 2.0 eq.) in hexane (5 mL) gave the crude product which contained a 50:50 mixture (by ^{1}H NMR spectroscopy) of alcohols **9a**. Purification by flash column chromatography on silica with 200:9:1 CH_2Cl_2-MeOH-$NH_4OH_{(aq)}$ as eluent gave a 50:50 mixture of alcohols **9a** (191 mg, 86%) as a light yellow solid.

Lab Book Reference: MA 6-55

(Scheme 2C, entry 5)

Using general procedure A, *N*-methyl thiomorpholine **6** (117 mg, 1.0 mmol, 1.0 eq.) *s*-BuLi (0.94 mL of a 1.4 M solution in cyclohexane, 1.3 mmol, 1.3 eq.), TMEDA (195 μL, 1.3 mmol, 1.3 eq.) at −10 °C for 2 h and benzaldehyde (203 μL, 2.0 mmol, 2.0 eq.) in hexane (5 mL) gave the crude product which contained a 50:50 mixture (by ^{1}H NMR spectroscopy) of alcohols **9a**. Purification by flash column chromatography on silica with 200:9:1 CH_2Cl_2-MeOH-$NH_4OH_{(aq)}$ as eluent gave a 50:50 mixture of alcohols **9a** (163 mg, 60%) as a light yellow solid and recovered *N*-methyl thiomorpholine **6** (30 mg, 38%) as a colourless oil.

Lab Book Reference: MA 6-52

(4-Methoxyphenyl)(4-methylthiomorpholin-2-yl)methanol 9b

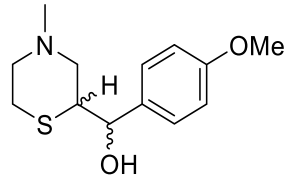

9b

Using general procedure A, *N*-methyl thiomorpholine **6** (117 mg, 1.0 mmol, 1.0 eq.), *s*-BuLi (0.94 mL of a 1.4 M solution in 92/8 cyclohexane/hexane, 2.6 mmol, 1.3 eq.) and TMEDA (195 µL, 1.3 mmol, 1.3 eq.) in hexane (5.0 mL) at −10 °C for 2 h and 4-methoxybenzaldehyde (243 µL, 2.0 mmol, 2.0 eq.) at −10 °C for 1 h gave the crude product. Purification by flash column chromatography on silica with 200:9:1 CH_2Cl_2-MeOH-$NH_4OH_{(aq)}$ as eluent gave a 50:50 mixture (by ^{1}H NMR spectroscopy) of diastereomeric thiomorpholines **9b** (193 mg, 75%) as a cream-yellow solid, R_F (100:9:1 CH_2Cl_2-MeOH-$NH_4OH_{(aq)}$) 0.6; IR (ATR) 3172 (OH), 2910, 2799, 1510, 1456 cm^{-1}; ^{1}H NMR (400 MHz, $CDCl_3$) 7.31 (d, *J* = 8.5 Hz, 1H, Ar), 7.27 (d, *J* = 8.5 Hz, 1H, Ar), 6.89 (d, *J* = 8.5 Hz, 2H, Ar), 5.00 (d, *J* = 7.0 Hz, 0.5H, HOC*H*), 4.98 (d, *J* = 5.0 Hz, 0.5H, HOC*H*), 3.80 (s, 3H, OMe), 3.22-3.10 (m, 1H, CH), 2.93-2.41 (m, 6H, CH), 2.24 (s, 1.5H, NMe), 2.20 (s, 1.5H, NMe); $^{13}C\{^1H\}$ NMR (100.6 MHz, $CDCl_3$) δ 159.1 (*ipso*-Ar), 159.0 (*ipso*-Ar), 135.6 (*ipso*-Ar), 134.0 (*ipso*-Ar), 127.8 (Ar), 127.1 (Ar), 113.8 (Ar), 113.6 (Ar), 79.1 (HOCH), 75.7 (HOCH), 59.6 (CH_2), 57.4 (CH_2), 56.3 (CH_2), 56.2 (CH_2), 55.30 (OMe), 55.29 (OMe), 47.3 (NMe), 47.2 (NMe), 47.1 (SCH), 44.7 (SCH), 27.1 (CH_2), 25.9 (CH_2); HRMS (ESI) *m/z* calcd for $C_{13}H_{19}NO_2S$ $(M + H)^+$ 254.1209, found 254.1211 (−0.9 ppm error).
Lab Book Reference: MA 6-62

1-(4-Methylthiomorpholin-2-yl)cyclopentan-1-ol 9c

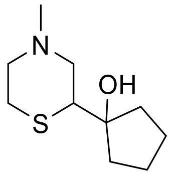

9c

Using general procedure A, *N*-methyl thiomorpholine **6** (117 mg, 1.0 mmol, 1.0 eq.), *s*-BuLi (0.94 mL of a 1.4 M solution in 92/8 cyclohexane/hexane, 2.6 mmol, 1.3 eq.) and TMEDA (195 µL, 1.3 mmol, 1.3 eq.) in hexane (5.0 mL) at −10 °C for 2 h and cyclopentanone (177 µL, 2.0 mmol, 2.0 eq.) at −10 °C for 1 h gave the crude product. Purification by flash column chromatography on silica with 200:9:1 CH_2Cl_2-MeOH-$NH_4OH_{(aq)}$ as eluent gave thiomorpholine **9c** (78 mg, 39%) as a light yellow solid, mp 58-60°C;

R_F (100:9:1 CH_2Cl_2-MeOH-$NH_4OH_{(aq)}$) 0.4; IR (ATR) 3170 (OH), 2955, 2804, 1458 cm^{-1}; ^{1}H NMR (400 MHz, $CDCl_3$) 3.23-3.12 (m, 1H, CH), 2.92 (dd, J = 12.0, 6.0 Hz, 1H, CH), 2.84 (dd, J = 12.0, 3.0 Hz, 1H, CH), 2.75-2.63 (m, 2H, CH), 2.63-2.54 (m, 2H, CH), 2.27 (s, 3H, NMe), 1.93-1.73 (m, 4H, CH), 1.73-1.53 (m, 4H, CH); $^{13}C\{^1H\}$ NMR (100.6 MHz, $CDCl_3$) δ 86.5 (HOC), 59.6 (CH_2), 56.4 (CH_2), 48.0 (SCH), 47.3 (NMe), 40.0 (NCH_2), 39.9 (CH_2), 27.0 (CH_2), 24.3 (CH_2), 23.7 (CH_2); HRMS (ESI) *m/z* calcd for $C_{10}H_{19}NOS$ $(M + H)^+$ 202.1260, found 202.1260 (+0.3 ppm error).
Lab Book Reference: MA 6-59

(4-Methylthiomorpholin-2-yl)diphenylmethanol 9d

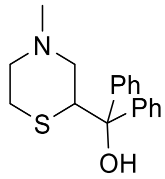

9d

Using general procedure A, *N*-methyl thiomorpholine **6** (117 mg, 1.0 mmol, 1.0 eq.), *s*-BuLi (0.94 mL of a 1.4 M solution in 92/8 cyclohexane/hexane, 2.6 mmol, 1.3 eq.) and TMEDA (195 µL, 1.3 mmol, 1.3 eq.) in hexane (5.0 mL) at −10 °C for 2 h and benzophenone (364 mg, 2.0 mmol, 2.0 eq.) gave the crude product. Purification by flash column chromatography on silica with 200:9:1 CH_2Cl_2-MeOH-$NH_4OH_{(aq)}$ as eluent gave morpholine **9d** (203 mg, 68%) as a cream-yellow solid, R_F (100:9:1 CH_2Cl_2-MeOH-$NH_4OH_{(aq)}$) 0.7; IR (ATR) 3172 (OH), 2804, 1488, 1445 cm^{-1}; ^{1}H NMR (400 MHz, $CDCl_3$) 7.58-7.50 (m, 4H, Ph), 7.34-7.27 (m, 4H, Ph), 7.21-7.15 (m, 2H, Ph), 3.68 (dd, J = 3.5, 3.5 Hz, 1H, CH), 3.46 (ddd, J = 13.7, 11.4, 3.5 Hz, 1H, CH), 2.98 (ddd, J = 12.0, 4.0, 4.0 Hz, 1H, CH), 2.93 (ddd, J = 12.0, 3.5, 1.0 Hz, 1H, CH), 2.62 (dd, J = 12.0, 3.0 Hz, 1H, NCH), 2.47-2.37 (m, 2H, NCH), 2.14 (s, 3H, NMe); $^{13}C\{^1H\}$ NMR (100.6 MHz, $CDCl_3$) δ 147.7 (*ipso*-Ph), 146.6 (*ipso*-Ph), 128.4 (Ph), 128.0 (Ph), 126.6 (Ph), 126.5 (Ph), 125.6 (Ph), 125.3 (Ph), 83.8 (HOC), 58.5 (CH_2), 56.1 (CH_2), 47.0 NMe), 44.9 (SCH), 26.6 (CH_2); HRMS (ESI) *m/z* calcd for $C_{18}H_{22}NOS$ $(M + H)^+$ 300.1417, found 300.1406 (+3.4 ppm error).
Lab Book Reference: MA 6-63

2-Benzoyl-4-methylthiomorpholine 9e

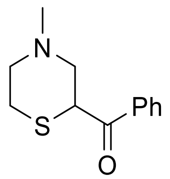

9e

Using general procedure A, *N*-methyl thiomorpholine **6** (117 mg, 1.0 mmol, 1.0 eq.), *s*-BuLi (0.94 mL of a 1.4 M solution in 92/8 cyclohexane/hexane, 2.6 mmol, 1.3 eq.) and TMEDA (195 µL, 1.3 mmol, 1.3 eq.) in hexane (5.0 mL) at −10 °C for 3 h and *N*-methoxy-*N*-methylbenzamide (330 mg, 2.0 mmol, 2.0 eq.) at −10 °C for 1 h gave the crude product. Purification by flash column chromatography on silica with 200:9:1 CH_2Cl_2-MeOH-$NH_4OH_{(aq)}$ as eluent gave morpholine **9e** (184 mg, 83%) as a yellow oil, R_F (100:9:1 CH_2Cl_2-MeOH-$NH_4OH_{(aq)}$) 0.7; IR (ATR) 2922, 2795, 1676 (C=O), 1595, 1447 cm^{-1}; ^1H NMR (400 MHz, $CDCl_3$) 8.04-7.98 (m, 2H, Ph), 7.61-7.55 (m, 1H, Ph), 7.50-7.44 (m, 2H, Ph), 4.61 (dd, J = 9.0, 2.5 Hz, 1H, CH), 3.17 (dd, J = 12.0, 2.5 Hz, 1H, CH), 3.08-2.96 (m, 2H, CH), 2.74 (ddd, J = 14.0, 4.5, 2.5 Hz, 1H, CH), 2.61 (dd, J = 12.0, 9.5 Hz, 1H, CH), 2.37 (ddd, J = 12.0, 11.0, 3.0 Hz, 1H, CH), 2.34 (s, 3H, NMe); $^{13}C\{^1H\}$ NMR (100.6 MHz, $CDCl_3$) δ 196.6 (C=O), 135.1 (*ipso*-Ph), 133.6 (Ph), 128.9 (Ph), 128.8 (Ph), 58.4 (CH_2), 56.0 (CH_2), 47.2 (NMe), 46.4 (SCH), 28.5 (CH_2); HRMS (ESI) *m/z* calcd for $C_{12}H_{15}NOS$ $(M + H)^+$ 222.0947, found 222.0950 (−0.8 ppm error).
Lab Book Reference: MA 6-61

Using general procedure A, *N*-methyl thiomorpholine **6** (118 mg, 1.0 mmol, 1.0 eq.), *s*-BuLi (1.0 mL of a 1.3 M solution in 92/8 cyclohexane/hexane, 1.3 mmol, 1.3 eq.) and TMEDA (45 µL, 0.30 mmol, 0.3 eq.) in hexane (5.0 mL) at −10 °C for 3 h and *N*-methoxy-*N*-methylbenzamide (330 mg, 2.0 mmol, 2.0 eq.) at −10 °C for 1 h gave the crude product. Purification by flash column chromatography on silica with 90:10 CH_2Cl_2-acetone to 80:20 CH_2Cl_2-acetone then 200:9:1 CH_2Cl_2-MeOH-$NH_4OH_{(aq)}$ to 100:9:1 CH_2Cl_2-MeOH-$NH_4OH_{(aq)}$ as eluent gave morpholine **9e** (187 mg, 90%) as a yellow oil.
Lab Book Reference: KK 2-76

4-Methyl-2-(prop-2-en-1-yl)thiomorpholine 9f

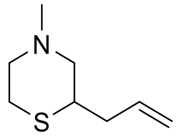

9f

Using general procedure A, *N*-methyl thiomorpholine **6** (117 mg, 1.0 mmol, 1.0 eq.), *s*-BuLi (0.94 mL of a 1.4 M solution in 92/8 cyclohexane/hexane, 2.6 mmol, 1.3 eq.) and TMEDA (195 μL, 1.3 mmol, 1.3 eq.), in hexane (5.0 mL) at −10 °C for 2 h and allyl bromide (173 μL, 2.0 mmol, 2.0 eq.) at −10 °C for 30 min gave the crude product. Purification by flash column chromatography on silica with 200:9:1 CH_2Cl_2-MeOH-$NH_4OH_{(aq)}$ as eluent gave thiomorpholine **9f** (87 mg, 55%) as a yellow oil, R_F (100:9:1 CH_2Cl_2-MeOH-$NH_4OH_{(aq)}$) 0.6; IR (ATR) 3075, 2914, 2789, 1453 cm^{-1}; ^1H NMR (400 MHz, $CDCl_3$) δ 5.79 (dddd, J = 17.0, 10.0, 7.0, 7.0 Hz, 1H, C*H*=CH_2), 5.12-5.03 (m, 2H, =CH), 3.00-2.91 (m, 3H, CH), 2.86 (ddd, J = 13.5, 11.0, 3.0 Hz, 1H, CH), 2.57 (ddd, J = 13.5, 4.0, 2.5 Hz, 1H, CH), 2.29 – 2.17 (m, 6H, CH, NMe, CH), 1.99 (dd, J = 11.5, 9.5 Hz, 1H CH); $^{13}C\{^1H\}$ NMR (100.6 MHz, $CDCl_3$) δ 134.9 (=CH), 117.3 (=CH_2), 62.5 (CH_2), 56.3 (CH_2), 47.1 (NMe), 40.5 (SCH), 38.0 (CH_2), 28.1 (CH_2); HRMS (ESI) *m/z* calcd for $C_8H_{15}NS$ $(M + H)^+$ 158.0998, found 158.1004 (−3.3 ppm error).
Lab Book Reference: MA 6-58

Using general procedure A, *N*-methyl thiomorpholine **6** (118 mg, 1.0 mmol, 1.0 eq.), *s*-BuLi (1.0 mL of a 1.3 M solution in 92/8 cyclohexane/hexane, 1.3 mmol, 1.3 eq.) and TMEDA (45 μL, 0.3 mmol, 0.3 eq.) in hexane (5.0 mL) at −10 °C for 3 h and allyl bromide (175 μL, 2.02 mmol, 2.0 eq.) at −10 °C for 1 h gave the crude product. Purification by flash column chromatography on silica with 200:9:1 CH_2Cl_2-MeOH-$NH_4OH_{(aq)}$ as eluent gave thiomorpholine **9f** (64.6 mg, 35%) as a yellow oil.
Lab Book Reference: KK2-62

2-[Dimethyl(phenyl)silyl]-4-methylthiomorpholine 9g

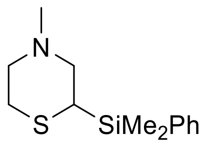

9g

Using general procedure A, *N*-methyl thiomorpholine **6** (117 mg, 1.0 mmol, 1.0 eq.), *s*-BuLi (0.94 mL of a 1.4 M solution in 92/8 cyclohexane/hexane, 2.6 mmol, 1.3 eq.) and TMEDA (195 µL, 1.3 mmol, 1.3 eq.), in hexane (5.0 mL) at −10 °C for 2 h and $PhMe_2SiCl$ (330 µL, 2.0 mmol, 2.0 eq.) at −10 °C for 30 min gave the crude product. Purification by flash column chromatography on silica with 400:9:1 CH_2Cl_2-MeOH-$NH_4OH_{(aq)}$ as eluent gave thiomorpholine **9g** (180 mg, 72%) as a yellow oil, R_F (100:9:1 CH_2Cl_2-MeOH-$NH_4OH_{(aq)}$) 0.6; IR (ATR) 2916, 2864, 2787, 1453 cm^{-1}; ^1H NMR (400 MHz, $CDCl_3$) δ 7.56-7.52 (m, 2H, Ph), 7.41-7.35 (m, 3H, Ph), 3.03 (ddd, J = 11.5, 3.0, 3.0 Hz, 1H, NCH), 2.94-2.84 (m, 2H, SCH), 2.59 (dd, J = 11.5, 2.5 Hz, 1H, NCH), 2.48 (ddd, J = 13.0, 3.0, 3.0 Hz, 1H, SCH), 2.20 (ddd, J = 12.0 12.0, 2.5 Hz, 1H, NCH), 2.20 (s, 3H, NMe), 2.08 (dd, J = 12.0, 12.0 Hz, 1H, NCH), 0.375 (s, 3H, SiMe), 0.372 (s, 3H, SiMe); $^{13}C\{^1H\}$ NMR (100.6 MHz, $CDCl_3$) δ 136.0 (*ipso*-Ph), 134.1 (Ph), 129.6 (Ph), 127.9 (Ph), 58.1 (CH_2), 56.6 (CH_2), 47.3 (NMe), 29.5 (CH_2), 28.8 (SCH), –4.45 (Me), –4.96 (Me); HRMS (ESI) *m/z* calcd for $C_{13}H_{21}NSSi$ $(M + H)^+$ 252.1237, found 252.1232 (+2.2 ppm error).
Lab Book Reference: MA 6-57

1-(4-Methylthiomorpholin-2-yl)cyclohexan-1-ol 9h

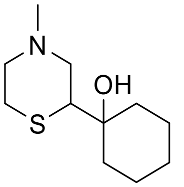

9h

Using general procedure A, *N*-methyl thiomorpholine **6** (117 mg, 1.0 mmol, 1.0 eq.), *s*-BuLi (0.94 mL of a 1.4 M solution in 92/8 cyclohexane/hexane, 2.6 mmol, 1.3 eq.) and TMEDA (195 µL, 1.3 mmol, 1.3 eq.) in hexane (5.0 mL) at −10 °C for 2 h, and cyclohexanone (207 µL, 2.0 mmol, 2.0 eq.) at −10 °C for 1 h gave the crude product. Purification by flash column chromatography on silica with 200:9:1 CH_2Cl_2-MeOH-$NH_4OH_{(aq)}$ as eluent gave morpholine **9h** (66 mg, 31%) as light yellow crystals, mp 92-93 °C; R_F (100:9:1 CH_2Cl_2-MeOH-$NH_4OH_{(aq)}$) 0.5; IR (ATR) 3179 (OH), 2920, 2799, 1458 cm^{-1}; ^1H NMR (400 MHz, $CDCl_3$) δ 3.10 (ddd, J = 13.0, 8.0, 3.5 Hz, 1H, NCH), 2.91 (dd, J = 12.0, 6.0 Hz, 1H, NCH), 2.85

(dd, J = 12.0, 3.0 Hz, 1H NCH), 2.70 (dd, J = 6.0, 3.0 Hz, 1H, CH), 2.68-2.35 (m, 3H, CH), 2.26 (s, 3H, NMe), 1.95-1.84 (m, 1H, CH), 1.77-1.44 (m, 8H, CH), 1.31-1.18 (m, 1H, CH); $^{13}C\{^1H\}$ NMR (100.6 MHz, $CDCl_3$) δ 74.6 (HOC), 57.8 (CH_2), 56.5 (CH_2), 47.3 (NMe or SCH), 36.9 (CH_2), 36.7 (CH_2), 26.9 (CH_2), 25.8 (CH_2), 22.4 (CH_2), 22.1 (CH_2) (NMe or SCH resonance not resolved); HRMS (ESI) *m/z* calcd for $C_{11}H_{21}NOS$ $(M + H)^+$ 216.1417, found 216.1415 (+0.9 ppm error).
Lab Book Reference: MA 6-60

Using general procedure A, *N*-methyl thiomorpholine **6** (118 mg, 1.0 mmol, 1.0 eq.), *s*-BuLi (1.0 mL of a 1.3 M solution in 92/8 cyclohexane/hexane, 1.3 mmol, 1.3 eq.) and TMEDA (45 µL, 0.30 mmol, 0.3 eq.) in hexane (5.0 mL) at −10 °C for 3 h and cyclohexanone (220 µL, 2.34 mmol, 2.0 eq.) at −10 °C for 1 h gave the crude product. Purification by flash column chromatography on silica with 80:20 CH_2Cl_2-acetone to pure acetone as eluent gave thiomorpholine **9h** (112 mg, 52%) as a colourless solid.

Lab Book Reference: KK2-80

1-Benzyl-4-(4-methylthiomorpholin-2-yl)piperidin-4-ol 9i

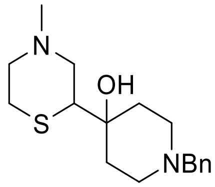

9i

Using general procedure A, *N*-methyl thiomorpholine **6** (117 mg, 1.0 mmol, 1.0 eq.), *s*-BuLi (1.0 mL of a 1.3 M solution in 92/8 cyclohexane/hexane, 1.3 mmol, 1.3 eq.) and TMEDA (45 µL, 0.3 mmol, 0.3 eq.) in hexane (5.0 mL) at −10 °C for 3 h and *N*-benzylpiperidin-4-one (350 µL, 1.96 mmol, 2.0 eq.) at −10 °C for 1 h gave the crude product. Purification by flash column chromatography on silica with 90:10 CH_2Cl_2-acetone to 10:40 CH_2Cl_2-acetone then 80:18:2 CH_2Cl_2-MeOH-$NH_4OH_{(aq)}$ as eluent gave morpholine **9i** (142 mg, 46%) as a yellow solid, mp 146 °C; R_F (80:18:2 CH_2Cl_2-MeOH-$NH_4OH_{(aq)}$) 0.6; IR (ATR) 3197 (OH), 2937, 2804, 1494, 1454, 1291, 1120, 911, 811, 731, 698 cm^{-1}; ^1H NMR (400 MHz, $CDCl_3$) 7.35-7.28 (m, 4H, Ph), 7.27-7.21 (m, 1H, Ph), 4.63 (br s, 1H, OH), 3.54 (s, 2H, NCH_2Ph), 3.21 (ddd, J = 13.0, 10.0, 3.5 Hz, 1H, CH), 3.06 (dd, J = 12.0, 5.0 Hz, 1H CH), 2.82-2.74 (m, 1H, NCH), 2.76 (dd, J = 12.0, 3.0 Hz, 1H, CH), 2.73-2.63 (m, 2H, CH), 2.57-2.48 (m, 2H, CH), 2.48 (ddd, J = 13.0, 5.5 Hz, 3.0 Hz, 1H CH), 2.41 (ddd, J = 12.0 Hz, 3.0 Hz, 1H CH), 2.35 (ddd, J = 12.0 Hz, 3.0 Hz, 1H, CH),

2.24 (s, 3H, NMe), 2.11 (ddd, *J* = 13.0, 3.0 Hz, 1H, CH) , 1.77 (ddd, *J* = 13.0, 3.0 Hz, 1H, CH), 1.71-1.54 (m, 2H, CH); $^{13}C\{^1H\}$ NMR (100.6 MHz, $CDCl_3$) δ 138.2 (*ipso*-Ph), 129.4 (Ph), 128.3 (Ph), 127.1 (Ph), 73.2 (HOC), 63.2 (N*C*H_2Ph), 57.7 (CH_2), 56.5 (CH_2), 49.5 (CH_2), 47.9 (SCH) 47.3 (NMe), 36.9 (CH_2), 36.6 (CH_2), 26.6 (CH_2); HRMS (ESI) *m/z* calcd for $C_{17}H_{26}N_2OS$ $(M + H)^+$ 307.1839, found 307.1843 (−1.0 ppm error).
Lab Book Reference: KK2-79

1-(4-Methylthiomorpholin-2-yl)-1-(naphthalen-1-yl)ethan-1-ol 9j

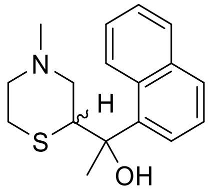

9j

Using general procedure A, *N*-methyl thiomorpholine **6** (118 mg, 1.0 mmol, 1.0 eq.), *s*-BuLi (1.0 mL of a 1.3 M solution in 92/8 cyclohexane/hexane, 1.3 mmol, 1.3 eq.) and TMEDA (45 µL, 0.30 mmol, 0.3 eq.) in hexane (5.0 mL) at −10 °C for 3 h and PhCHO (350 µl, 1.97 mmol, 2.0 eq.) at −10 °C for 1 h gave the crude product which contained a 55:45 mixture (by ^1H NMR spectroscopy) of alcohols **9j**. Purification by flash column chromatography on silica with 90:10 CH_2Cl_2:acetone to 20:80 CH_2Cl_2:acetone then 400:9:1 CH_2Cl_2-MeOH-$NH_4OH_{(aq)}$ as eluent gave alcohol **9ja** (47.2 mg, 16%) as a white solid, mp 140-142 °C, R_F (60:40 CH_2Cl_2:acetone) 0.6; IR (ATR) 3170 (OH), 3048, 2977, 2934, 2803, 1509, 1457, 1374, 1274, 1249, 1115, 1093, 907, 778, 729 cm^{-1}; ^1H NMR (400 MHz, $CDCl_3$) δ 8.10 (br d, *J* = 8.0 Hz, 1H, Ar), 8.06 (d, *J* = 7.5 Hz, 1H, Ar), 7.93-7.86 (m, 1 H, Ar), 7.80-7.73 (m, 1H, Ar), 7.53-7.41 (m, 3H, Ar), 6.83 (br s, 1H, OH), 3.65 (dd, *J* = 3.5, 3.5 Hz, 1H, SCH), 3.57 (ddd, *J* = 13.0, 11.5, 3.5 Hz, 1H, SCH), 3.02 (ddd, *J* = 11.5, 3.5, 3.5 Hz, 1H, NCH), 2.58 (dd, *J* = 12.5, 3.5 Hz, 1H, NCH), 2.48 (ddd, *J* = 13.5, 3.5, 3.5 Hz, 1H, SCH), 2.40-2.29 (m, 2H, NCH), 2.04 (s, 3H, NMe), 2.03 (CMe); $^{13}C\{^1H\}$ NMR (100.6 MHz, $CDCl_3$) δ 144.4 (*ipso*-Ar), 135.0 (*ipso*-Ar), 129.73 (Ar), 129.68 (*ipso*-Ar), 128.4 (Ar), 125.6 (Ar), 125.42 (Ar), 125.41 (Ar), 125.0 (Ar), 124.5 (Ar), 81.4 (OCH), 58.7 (NCH_2), 56.3 (NCH_2), 46.9 (NMe), 45.1 (SCH), 28.8 (C*Me*), 26.6 (SCH_2); HRMS (ESI) *m/z* calcd for $C_{17}H_{21}NOS$ $(M + H)^+$ 288.1417, found 288.1416 (+0.0 ppm error) and alcohol **9jb** (41.3 mg, 14%) as a white solid, mp 142-144 °C; R_F (60:40 CH_2Cl_2:acetone) 0.3; IR (ATR) 3046 (OH), 2977, 2948, 280, 1460, 1447, 1415, 1296, 1190, 1134, 1110, 1013, 975, 954, 907, 810, 780, 734, 617, 572, 500, 490 cm^{-1}; ^1H NMR (400 MHz, $CDCl_3$) δ 8.41 (br d, *J* = 7.5 Hz, 1H, Ar), 7.90-7.85 (m, 1H, Ar), 7.85 (br d *J* =

7.0 Hz, 1H, Ar), 7.79 (br d , *J* = 8.0 Hz, 1H, Ar), 7.50-7.42 (m, 3H, Ar), 4.95 (br s, 1H, OH), 3.87 (dd, *J* = 6.5 Hz, 3.0 Hz, 1H, SCH), 3.02-2.90 (m, 2H, NCH, SCH), 2.85 (dd, *J* = 12.0 Hz, 3.0 Hz, 1H, SCH), 2.69-2.49 (m, 3H, SCH, NCH), 2.27 (s, 3H, NMe), 1.94 (CMe); $^{13}C\{^1H\}$ NMR (100.6 MHz, $CDCl_3$) δ 142.2 (*ipso*-Ar), 134.8 (*ipso*-Ar), 130.3 (*ipso*-Ar), 129.6 (Ar), 128.7 (Ar), 125.9 (Ar), 125.6 (Ar), 125.0 (Ar), 124.9 (Ar), 124.5 (Ar), 79.3 (OCH), 58.4 (NCH_2), 56.2 (NCH_2), 48.0 (SCH), 47.2 (NMe), 28.6 (C*Me*), 27.3 (SCH_2); HRMS (ESI) *m/z* calcd for $C_{17}H_{21}NOS$ $(M + H)^+$ 288.1417, found 288.1416 (+0.0 ppm error) and a mixture of alcohols **9ja** and **9jb** (41.1 mg, 15%) as a white solid. Total yield of alcohols **9j** 129.6 mg (45%). The relative stereochemistry of **9ja** and **9jb** is unknown.

Lab Book Reference: KK2-78

N-tert-Butyl-4-methylthiomorpholine-2-carboxamide 9k

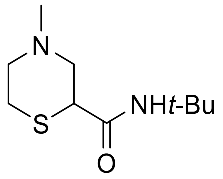

9k

Using general procedure A, *N*-methyl thiomorpholine **6** (117 mg, 1.0 mmol, 1.0 eq.), *s*-BuLi (1.0 mL of a 1.3 M solution in 92/8 cyclohexane/hexane, 1.3 mmol, 1.3 eq.) and TMEDA (45 µL, 0.3 mmol, 0.3 eq.) in hexane (5.0 mL) at −10 °C for 3 h and *t*-butyl isocyanate (200 µL, 1.75 mmol, 2.0 eq.) at −10 °C for 1 h gave the crude product. Purification by flash column chromatography on silica with 95:5 CH_2Cl_2-acetone to 50:50 CH_2Cl_2-acetone as eluent gave thiomorpholine **9k** (131 mg, 61%) as a colourless solid, mp 101-104 °C; R_F (80:210 CH_2Cl_2-acetone) 0.1; IR (ATR) 3313 (NH), 3055, 2964, 2921, 2801, 1652 (C=O), 1550, 1453, 1391, 1377, 1362, 1300, 1287, 1224, 1132, 1123, 1073, 1023 cm^{-1}; ^1H NMR (400 MHz, $CDCl_3$) 7.87 (br s, 1H, NH), 3.15 (dd, *J* = 4.0, 3.0 Hz, 1H, SCH), 3.09 (dd, *J* = 12.0, 4.0 Hz, 1H, NCH), 2.96 (ddd, *J* = 13.0, 10.5, 3.0 Hz, 1H, SCH), 2.91 (ddd, *J* = 11.5, 4.5, 3.0 Hz, 1H, NCH), 2.57 (dd, *J* = 12.0, 3.0 Hz, 1H, NCH), 2.54-2.46 (m, 1H, SCH), 2.38 (ddd, *J* = 11.5, 10.5, 2.5 Hz, 1H, NCH), 2.26 (s, 3H, NMe), 1.35 (s, 9H, CMe_3); $^{13}C\{^1H\}$ NMR (100.6 MHz, $CDCl_3$) δ 170.1 (C=O), 57.4 (NCH_2), 55.9 (NCH_2), 51.0 (N*C*Me_3), 46.9 (NMe), 42.8 (SCH), 28.9 (C*Me*$_3$), 26.1 (SCH_2); HRMS (ESI) *m/z* calcd for $C_{10}H_{20}N_2OS$ $(M + H)^+$ 217.1369, found 217.1371 (−1.0 ppm error).

Lab Book Reference: KK2-77

4-Methyl-2-pentylthiomorpholine 9l

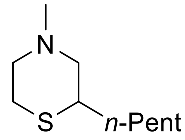

9l

Using general procedure A, *N*-methyl thiomorpholine **6** (117 mg, 1.0 mmol, 1.0 eq.), *s*-BuLi (1.0 mL of a 1.3 M solution in 92/8 cyclohexane/hexane, 1.3 mmol, 1.3 eq.) and TMEDA (45 μL, 0.3 mmol, 0.3 eq.) in hexane (5.0 mL) at −10 °C for 3 h and pentyl iodide (260 μL, 1.99 mmol, 2.0 eq.) at −10 °C for 1 h gave the crude product. Purification by flash column chromatography on silica with 490:9:1 CH_2Cl_2-MeOH-$NH_4OH_{(aq)}$ to 90:9:1 CH_2Cl_2-MeOH-$NH_4OH_{(aq)}$ as eluent gave morpholine **9l** (64.6 mg, 35%) as a yellow oil, R_F (90:9:1 CH_2Cl_2-MeOH-$NH_4OH_{(aq)}$) 0.5; IR (ATR) 2956, 2924, 2856, 2790, 1454, 1414, 1377, 1289, 1185, 1123, 1060, 978, 909, 789, 727 cm^{-1}; ^{1}H NMR (400 MHz, $CDCl_3$) δ 3.02-2.95 (m, 1H, NCH), 2.97 (dd, J = 11.5, 3.5 Hz, 1H, NCH), 2.93-2.85 (m, 1H, SCH), 2.87 (ddd, J = 14.0, 11.0, 3.0 Hz, 1H, SCH), 2.56 (ddd, J = 13.5, 4.0, 2.5 Hz, 1H, SCH), 2.29 (s, 3H, NMe), 2.23 (ddd, J = 11.5, 2.5 Hz, 1H, NCH), 2.01 (dd, J = 11.5, 10.0 Hz, 1H, NCH), 1.54-1.34 (m, 4H, $(CH_2)_2(CH_2)_2Me$), 1.34-1.20 (m, 4H, $(CH_2)_2Me$), 0.87 (t, J = 7.0 Hz, 3H, CH_2Me); $^{13}C\{^1H\}$ NMR (100.6 MHz, $CDCl_3$) δ 63.4 (NCH_2), 56.6 (NCH_2), 47.2 (NMe), 41.3 (SCH), 33.6 ($CH_2(CH_2)_3Me$), 31.8 (CH_2CH_2Me), 28.2 (SCH_2), 26.6 ($CH_2(CH_2)_2Me$), 22.6 (CH_2Me), 14.2 (CH_2Me); HRMS (ESI) *m/z* calcd for $C_{10}H_{21}NS$ $(M + H)^+$ 188.1467, found 188.1473 (−3.2 ppm error).
Lab Book Reference: KK2-62

4-Phenyl-3,6-dihydro-2*H*-thiopyran S3

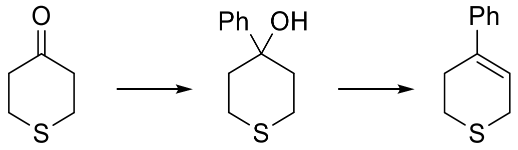

S2 **S3**

PhMgBr (10.0 mL of a 3.0 M solution in Et_2O, 30 mmol, 1.5 eq.) was added dropwise to a stirred solution of tetrahydro-4*H*-thiopyran-4-one (2.34 g, 20 mmol, 1.0 eq.) in Et_2O (60 mL) at 0 °C under Ar. After being allowed to warm to rt, the resulting solution was stirred and heated at reflux for 1 h. Then, the mixture was cooled to 0 °C and ice-water (100 mL) was added. The two layers were separated and the aqueous layer was extracted with Et_2O (2 × 80 mL). The combined organic layers were dried ($MgSO_4$) and evaporated under reduced pressure to give crude alcohol **S2**. Crude alcohol **S2** was dissolved in THF

(40 mL) and the solution was cooled to 0 °C. Then, BF_3•OEt_2 (10 mL, 81 mmol, 2.7 eq.) was added and the solution was allowed to warm to rt and stirred at rt for 2 h. Then, the solvent was evaporated under reduced pressure and saturated $NaHCO_{3(aq)}$ (20 mL) and EtOAc (40 mL) were added. The two layers were separated and the aqueous layer was extracted with EtOAc (2 × 80 mL). The combined organic layers were dried ($MgSO_4$) and evaporated under reduced pressure to give the crude product. Purification by flash column chromatography on silica with 95:5 hexane-EtOAc as eluent gave 4-phenyl-3,6-dihydro-2*H*-thiopyran **S3** (2.01 g, 57%) as a colourless oil, R_F (50:50 hexane-EtOAc) 0.5; IR (ATR) 3057, 2919, 1661 (C=C), 1495, 1446, 1138, 1034, 871, 767, 743, 697 cm^{-1}; ^{1}H NMR (400 MHz, $CDCl_3$) δ 7.38-7.23 (m, 5H, Ph), 6.20-6.15 (m, 1H, C=CH), 3.34 (dt, J = 4.5, 2.5 Hz, 2H, SCH_2), 2.89 (t, J = 5.5, Hz, 2H, SCH_2), 2.74-2.68 (m, 2H, CH_2); $^{13}C\{^1H\}$ NMR (100.6 MHz, $CDCl_3$) δ 143.0 (*ipso*-Ph), 138.3 (*C*=CH), 128.4 (Ph), 127.3 (Ph), 125.6 (Ph), 121.7 (C=*C*H), 28.7 (CH_2), 26.3 (CH_2), 25.5 (CH_2); HRMS (APCI) *m/z* calcd for $C_{11}H_{12}S$ (M + H)$^+$ 177.0732, found 177.0728 (–2.4 ppm error). Spectroscopic data consistent with those reported in the literature.[8]
Lab Book Reference: NS2-100

4-Phenylthiane 10a

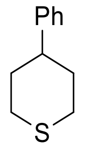

10a

10% Pd/C (1.11 g, 1.1 mmol, 10 mol%) was added to a stirred solution of 4-phenyl-3,6-dihydro-2*H*-thiopyran **S3** (1.83 g, 11 mmol, 1.0 eq.) in MeOH (80 mL). Then, the reaction flask was evacuated under reduced pressure and back-filled with Ar three times. After a final evacuation, a balloon of H_2 was attached and the reaction mixture was stirred vigorously under a balloon of H_2 at rt for 24 h. The solids were removed by filtration through Celite® and washed with 10:1 CH_2Cl_2-MeOH (50 mL). The filtrate was evaporated under reduced pressure to give 4-phenylthiane **10a** (1.83 g, 98%) as a yellow oil, R_F (95:5 hexane-CH_2Cl_2) 0.4; IR (ATR) 3032, 2907, 2892, 2924, 1494, 1425, 1269, 948, 759, 695 cm^{-1}; ^{1}H NMR (400 MHz, $CDCl_3$) δ 7.38 (dd, J = 7.5, 7.5 Hz, 2H, Ph), 7.30-7.25 (m, 3H, Ph), 2.91 (ddd, J = 12.5, 12.5, 3.5 Hz, 2H, SCH), 2.77 (br d, J = 12.5 Hz, 2H, SCH), 2.59 (tt, J = 12.5, 3.5 Hz, 1H, C*H*Ph), 2.25-2.17 (m, 2H, CH), 1.93 (dddd, J = 12.5, 12.5, 12.5, 3.5 Hz, 2H, CH); $^{13}C\{^1H\}$ NMR (100.6 MHz, $CDCl_3$) δ 147.0 (*ipso*-Ph), 128.6 (Ph), 126.9 (Ph), 126.4 (Ph), 44.4 (*C*HPh), 35.2 (CH_2), 29.4 (SCH_2); HRMS

(APCI) *m/z* calcd for $C_{11}H_{14}S$ $(M + H)^+$ 179.0889, found 179.0886 (+1.6 ppm error). Spectroscopic data consistent with those reported in the literature.[9]
Lab Book Reference: NS3-4

Thian-4-ol S4

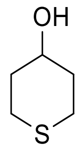

S4

$NaBH_4$ (1.51 g, 40 mmol, 2.0 eq.) was added to a stirred solution of tetrahydro-4*H*-thiopyran-4-one (2.52 g, 20 mmol, 1.0 eq.) in MeOH (100 mL) at 0 °C under Ar. The resulting mixture was allowed to warm to rt and stirred at rt for 16 h. The solvent was evaporated under reduced pressure to give the crude product. Purification by flash column chromatography on silica with 50:50 hexane-EtOAc as eluent gave alcohol **S4** (2.16 g, 97%) as a white solid, mp 48-49 °C (lit.,[10] 48-50 °C), R_F (50:50 hexane-EtOAc) 0.2; ^{1}H NMR (400 MHz, $CDCl_3$) δ 3.64 (tt, J = 9.5, 3.5 Hz, 1H, OCH), 2.80-2.71 (m, 2H, SCH), 2.58 (ddd, J = 13.5, 10.0, 3.0 Hz, 2H, SCH), 2.20 – 2.10 (m, 2H CH), 1.69 (dddd, J = 13.0, 7.5, 9.5, 3.0 Hz, 3H, OH, CH); $^{13}C\{^1H\}$ NMR (100.6 MHz, $CDCl_3$) δ 68.9 (OCH), 36.2 (SCH_2), 26.6 (CH_2). Spectroscopic data consistent with those reported in the literature.[10]
Lab Book Reference: NS2-44

Tris(propan-2-yl)(thian-4-yloxy)silane 10b

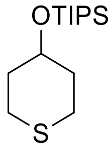

10b

Pyridine (1.36 mL, 17 mmol, 2.0 eq.) and TIPSOTf (4.56 mL, 17 mmol, 2.0 eq.) were added to a stirred solution of alcohol **S4** (1.0 g, 8.5 mmol, 1.0 eq.) in CH_2Cl_2 (50 mL) at rt under Ar. The resulting solution was stirred at rt for 30 min. Then, saturated $NH_4Cl_{(aq)}$ (10 mL) was added and the two layers were separated. The aqueous layer was extracted with CH_2Cl_2 (3 × 10 mL). The combined organic layers were washed with 15% $CuSO_{4(aq)}$ (30 mL) and with saturated $EDTA_{(aq)}$ (30 mL), dried ($MgSO_4$) and evaporated under reduced pressure to give the crude product. Purification by flash column

chromatography on silica with 95:5 hexane-Et_2O as eluent gave TIPS-protected tetrahydrothiopyran **10b** (2.26 g, 90%) as a colourless oil, R_F (95:5 hexane-Et_2O) 0.2; IR (ATR) 2940, 2865, 2892, 1463, 1425, 1105, 1083, 1067, 996, 824, 788, 678, 657, 627 cm^{-1}; ^1H NMR (400 MHz, $CDCl_3$) δ 3.90 (tt, J = 7.0, 3.0 Hz, 1H, OCH), 2.94 (ddd, J = 12.5, 9.0, 3.0, 2H, SCH), 2.43 (ddd, J = 13.5, 8.0, 3.0 Hz, 2H, SCH), 2.00 (dddd, J = 12.0, 9.0, 3.0, 3.0 Hz, 2H, CH), 1.84 (dddd, J = 13.5, 7.5, 7.5, 3.0 Hz, 2H, CH), 1.054-1.049 (m, 21H, $CHMe_2$); $^{13}C\{^1H\}$ NMR (100.6 MHz, $CDCl_3$) δ 67.9 (OCH), 36.2 (SCH_2), 25.1 (CH_2), 18.2 (CH*Me*$_2$), 12.4 (*C*HMe$_2$); HRMS (ESI) *m/z* calcd for $C_{14}H_{30}OSSi$ $(M + Na)^+$ 297.1679, found 297.1674 (+1.7 ppm error).

Lab Book Reference: NS2-46

Phenyl[2,4-*cis*-4-phenylthian-2-yl]methanol 11a

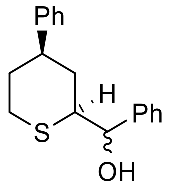

11a

Using general procedure A, 4-phenylthiane **10a** (178 mg, 1.0 mmol, 1.0 eq.), *s*-BuLi (1.0 mL of a 1.3 M solution in 92/8 cyclohexane/hexane, 1.3 mmol, 1.3 eq.) and TMEDA (195 µL, 1.3 mmol, 1.3 eq.) in hexane (5 mL) at 0 °C for 2 h and benzaldehyde (201 µL, 2.0 mmol, 2.0 eq.) at 0 °C for 1 h gave the crude product which contained a 60:40 mixture (by ^1H NMR spectroscopy) of alcohols **11a**. Purification by flash column chromatography on silica with 85:15 hexane-CH_2Cl_2 then 50:50 hexane-CH_2Cl_2 then 90:9:1 CH_2Cl_2-MeOH-$NH_4OH_{(aq)}$ gave a 60:40 mixture of alcohols **11a** (221 mg, 78%) as a colourless oil, R_F (50:50 hexane-CH_2Cl_2) 0.1; IR (ATR) 3403 (OH), 3060, 3027, 2925, 2903, 1493, 1452, 1024, 760, 699 cm^{-1}; ^1H NMR (400 MHz, $CDCl_3$) δ 7.43-7.25 (m, 7H, Ph), 7.26-7.12 (m, 3H, Ph), 4.78 (d, J = 5.0 Hz, 0.4H, OCH), 4.65 (d, J = 7.5 Hz, 0.6H, OCH), 3.35-3.26 (m, 1H, SCH), 2.95-2.75 (m, 2H, SCH_2), 2.55-2.44 (m, 1.4H, CH), 2.26 (br d, J = 13.5 Hz, 0.6H, CH), 2.18-2.09 (m, 1H, CH), 1.88 (br d, J = 13.0 Hz, 0.6H, CH), 1.85-1.67 (m, 2.0H, CH, OH), 1.63 (br s, 0.4H, OH); $^{13}C\{^1H\}$ NMR (100.6 MHz, $CDCl_3$) δ 146.6 (*ipso*-Ph), 146.5 (*ipso*-Ph), 141.40 (*ipso*-Ph), 141.39 (*ipso*-Ph), 128.7 (Ph), 128.6 (Ph), 128.5 (Ph), 128.3 (Ph), 128.1 (Ph), 127.0 (Ph), 126.9 (Ph), 126.8 (Ph), 126.52 (Ph), 126.47 (Ph), 77.9 (HOCH), 76.7 (HOCH), 51.3 (SCH), 50.3 (SCH), 44.5 (*C*HPh), 44.4 (*C*HPh), 38.2 (CH_2), 36.2 (CH_2), 34.9 (CH_2), 34.8 (CH_2), 29.7 (CH_2), 29.3 (CH_2) (two Ph resonance not resolved); HRMS (ESI) *m/z* calcd for $C_{18}H_{20}OSS$ $(M + Na)^+$ 307.1127, found 307.1113 (–4.5 ppm error).

Lab Book Reference: NS3-5

1-Benzyl-4-[2,4-*cis*-4-phenylthian-2-yl]piperidin-4-ol 11b

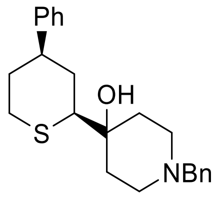

11b

Using general procedure A, 4-phenylthiane **10a** (178 mg, 1.0 mmol, 1.0 eq.), *s*-BuLi (1.0 mL of a 1.3 M solution in 92/8 cyclohexane/hexane, 1.3 mmol, 1.3 eq.) and TMEDA (195 µL, 1.3 mmol, 1.3 eq.) in hexane (5 mL) at 0 °C for 2 h and 1-benzylpiperidin-4-one (371 µL, 2.0 mmol, 2.0 eq.) at 0 °C for 1 h gave the crude product. Purification by flash column chromatography on silica with 98.5:1.4:0.1 CH_2Cl_2-MeOH-$NH_4OH_{(aq)}$ then 97.5:2:0.5 CH_2Cl_2-MeOH-$NH_4OH_{(aq)}$ gave alcohol **11b** (205 mg, 56%) as a colourless oil, R_F (98.5:1.4:0.1 CH_2Cl_2-MeOH-$NH_4OH_{(aq)}$) 0.2; IR (ATR) 3445 (OH), 3027, 2919, 2813, 1467, 1494, 1453, 1366, 1109, 1074, 1028, 762, 735, 699 cm^{-1}; ^1H NMR (400 MHz, $CDCl_3$) δ 7.47-7.17 (m, 10H, Ph), 3.55 (s, 2H, NC*H*₂Ph), 3.01 (dd, J = 12.0, 2.5 Hz, 1H, SCH), 2.92-2.85 (m, 2H, CH), 2.76-2.69 (m, 2H, CH), 2.56 (dddd, J = 12.5, 12.5, 3.0, 3.0 Hz, 1H, CH), 2.36 (dddd, 2H, J = 12.0, 12.0, 3.0, 3.0 Hz, CH), 2.28 (ddd, J = 13.0, 3.0, 3.0 Hz, 1H, CH), 2.16 (dddd, J = 13.0, 3.0, 3.0, 3.0 Hz, 1H, CH), 1.93-1.68 (m, 6H, CH); $^{13}C\{^1H\}$ NMR (100.6 MHz, $CDCl_3$) δ 146.7 (*ipso*-Ph), 138.6 (*ipso*-Ph), 129.2 (Ph), 128.7 (Ph), 128.3 (Ph), 127.1 (Ph), 126.9 (Ph), 126.6 (Ph), 71.2 (HOC), 63.2 (N*C*H_2Ph), 55.0 (SCH), 49.3 (CH_2), 44.9 (*C*HPh), 35.8 (CH_2), 35.4 (CH_2), 34.8 (CH_2), 34.3 (CH_2), 29.4 (CH_2); HRMS (ESI) *m/z* calcd for $C_{23}H_{29}NOS$ $(M + H)^+$ 368.2043, found 368.2043 (+0.2 ppm error).
Lab Book Reference: NS4-48

2,4-*cis*-4-Phenylthiane-2-carboxylic acid 11c

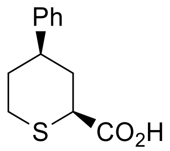

11c

Using general procedure B, 4-phenylthiane **10a** (178 mg, 1.0 mmol, 1.0 eq.), *s*-BuLi (1.0 mL of a 1.3 M solution in 92/8 cyclohexane/hexane, 1.3 mmol, 1.3 eq.) and TMEDA (195 µL, 1.3 mmol, 1.3 eq.) in hexane (5 mL) at 0 °C for 2 h and CO_2 at 0 °C for 1 h gave the crude product. Purification by flash column chromatography on silica with 500:9:1 CH_2Cl_2-MeOH-$NH_4OH_{(aq)}$ gave acid **11c** (86 mg, 39%) as a colourless oil, R_F (500:9:1 CH_2Cl_2-MeOH-$NH_4OH_{(aq)}$) 0.1; IR (ATR) 3443 (OH), 3027, 2923, 2811,

1717 (C=O), 1494, 1454, 1349, 1118, 1072, 1028, 811, 738, 699 cm^{-1}; ^{1}H NMR (400 MHz, $CDCl_3$) δ 9.92 (br s, 1H, CO_2H), 7.50-7.16 (m, 5H, Ph), 3.82 (dd, J = 13.0, 3.0 Hz, 1H, SCH), 2.96-2.88 (m, 1H, CH), 2.83 (ddd, J = 13.0, 3.0, 3.0 Hz, 1H, CH), 2.59 (dddd, J = 13.0, 13.0, 3.0, 3.0 Hz, 1H, CH), 2.48 (br d, J = 13.0 Hz, 1H, CH), 2.16 (br d, J = 13.0 Hz, 1H, CH), 2.00 (ddd, J = 13.0, 13.0, 13.0 Hz, 1H, CH), 1.84 (dddd, J = 13.0, 13.0, 13.0, 3.0 Hz, 1H, CH); $^{13}C\{^{1}H\}$ NMR (100.6 MHz, $CDCl_3$) δ 177.5 (C=O) , 145.6 (*ipso*-Ph), 128.8 (Ph), 126.9 (Ph), 126.8 (Ph), 46.2 (SCH), 44.2 (*C*HPh), 37.9 (CH_2), 34.2 (CH_2), 29.7 (CH_2); HRMS (ESI) *m/z* calcd for $C_{12}H_{14}O_2S$ $(M + H)^+$ 223.0787, found 223.0790 (+1.3 ppm error).

Lab Book Reference: NS4-47

Phenyl[2,4-*cis*-4-phenylthian-2-yl]methanone 11d

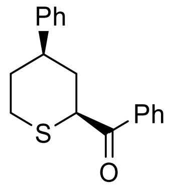

11d

Using general procedure A, 4-phenylthiane **10a** (178 mg, 1.0 mmol, 1.0 eq.), *s*-BuLi (1.0 mL of a 1.3 M solution in 92/8 cyclohexane/hexane, 1.3 mmol, 1.3 eq.) and TMEDA (195 µL, 1.3 mmol, 1.3 eq.) in hexane (5 mL) at 0 °C for 2 h and *N*-methoxy-*N*-methylbenzamide (304 µL, 2.0 mmol, 2.0 eq.) at 0 °C for 1 h gave the crude product. Purification by flash column chromatography on silica with 85:15 hexane-CH_2Cl_2 then 50:50 hexane-CH_2Cl_2 gave ketone **11d** (192 mg, 68%) as a white solid, mp 73 °C; R_F (50:50 hexane-CH_2Cl_2) 0.4; IR (ATR) 3059, 3027, 2906, 2842, 1679 (C=O), 1597, 1448, 1281, 1258, 1226, 1004, 987, 759, 722, 699, 688 cm^{-1}; ^{1}H NMR (400 MHz, $CDCl_3$) δ 8.05 (d, J = 7.5 Hz, 2H, Ph), 7.60-7.55 (m, 1H, Ph), 7.47 (dd, J = 7.5, 7.5 Hz, 2H, Ph), 7.34-7.29 (m, 2H, Ph), 7.25-7.19 (m, 3H, Ph), 4.66 (dd, J = 12.5, 3.0 Hz, 1H, SCHC(O)), 3.07 (ddd, J = 12.0, 12.0, 3.0 Hz, 1H, CH), 2.91 (ddd, J = 12.0, 3.0, 3.0 Hz, 1H, CH), 2.73 (dddd, J = 12.5, 12.5, 3.0, 3.0 Hz, 1H, C*H*Ph), 2.38-2.23 (m, 2H, CH), 2.23-2.12 (m, 1H, CH), 1.89 (dddd, J = 12.5, 12.5, 12.5, 3.0 Hz, 1H, CH); $^{13}C\{^{1}H\}$ NMR (100.6 MHz, $CDCl_3$) δ 197.5 (C=O), 146.0 (*ipso*-Ph), 135.3 (Ph), 133.6 (*ipso*-Ph), 129.0 (Ph), 128.8 (Ph), 128.8 (Ph), 127.0 (Ph), 126.8 (Ph), 49.3 (SCH), 44.4 (*C*HPh), 37.5 (CH_2), 34.8 (CH_2), 30.4 (CH_2); MS (ESI) *m/z* 305 [(M + Na)$^+$, 100]; HRMS (ESI) *m/z* calcd for $C_{18}H_{18}OS$ $(M + Na)^+$ 305.0971, found 305.0965 (+1.7 ppm error). The structure was confirmed by X-Ray crystallography (CCDC 2294609).

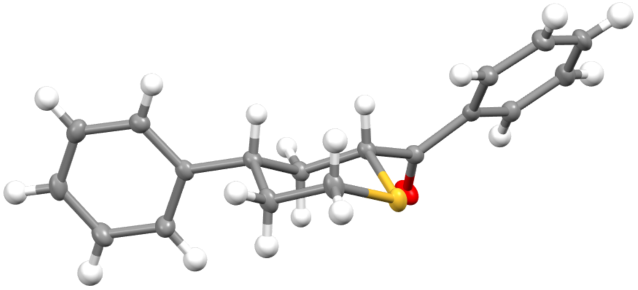

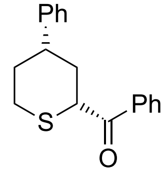

11d

Lab Book Reference: NS3-13

Phenyl[2,4-*cis*-{[tris(propan-2-yl)silyl]oxy}thian-2-yl]methanol 11e

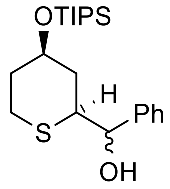

11e

Using general procedure A, TIPS-protected tetrahydrothiopyran **10b** (273 mg, 1.0 mmol, 1.0 eq.), *s*-BuLi (1.0 mL of a 1.3 M solution in 92/8 cyclohexane/hexane, 1.3 mmol, 1.3 eq.) and TMEDA (195 µL, 1.3 mmol, 1.3 eq.) in hexane (5 mL) at 0 °C for 2 h and benzaldehyde (210 µL, 2.0 mmol, 2.0 eq.) at 0 °C for 1 h gave the crude product which contained a 55:45 mixture (by ^{1}H NMR spectroscopy) of alcohols **11e**. Purification by flash column chromatography on silica with 80:20 hexane-CH_2Cl_2 then 90:10 CH_2Cl_2-MeOH as eluent gave a 55:45 mixture of alcohols **11e** (331 mg, 87%) as a colourless oil, R_F (50:50 hexane-CH_2Cl_2) 0.2; IR (ATR) 2942, 2891, 2865, 1462, 1454, 1263, 1100, 1067, 881, 699, 679 cm^{-1}; ^{1}H NMR (400 MHz, $CDCl_3$) δ 7.40-7.26 (m, 5H, Ph), 4.79 (dd, J = 5.5, 3.0 Hz, 0.6H, HOC*H*), 4.68 (dd, J = 7.5, 3.0 Hz, 0.4H, HOC*H*), 3.64-3.53 (m, 1H, SCH), 3.14-3.03 (m, 1H, OCH), 2.85 (ddd, J = 13.5, 5.0, 3.5 Hz, 0.4H, CH), 2.81-2.76 (m, 0.4H, OH), 2.76-2.72 (m, 0.6H, CH), 2.68-2.55 (m, 1H, CH), 2.42 (br d, J = 3.0 Hz, 0.6H, OH), 2.29-2.22 (m, 0.6H, CH), 2.17-2.10 (m, 1H, CH), 1.91 (ddd, J = 13.0, 3.5, 3.5 Hz, 0.4H, CH), 1.73-1.52 (m, 2H, CH), 1.06-0.92 (m, 21H, $CHMe_2$); $^{13}C\{^{1}H\}$ NMR (100.6 MHz, $CDCl_3$) δ 141.6 (*ipso*-Ph), 128.5 (Ph), 128.4 (Ph), 128.2 (Ph), 128.0 (Ph), 126.8 (Ph), 126.4 (Ph), 77.6 (HOCH), 76.4 (HOCH), 70.7 (OCH), 70.3 (OCH), 50.0 (SCH), 49.0 (SCH), 40.5 (CH_2), 38.2 (CH_2), 36.8 (CH_2), 36.6 (CH_2), 26.8 (CH_2), 26.2 (CH_2), 18.2 ($CHMe_2$), 18.1 ($CHMe_2$), 12.4 (*C*HMe$_2$), 12.3 (*C*HMe$_2$) (one *ipso*-Ar resonance not resolved); HRMS (ESI) *m/z* calcd for $C_{21}H_{36}O_2SSi$ (M + Na)$^+$ 403.2097, found 403.2084 (–3.2 ppm error).

Lab Book Reference: NS2-47

[2,4-*cis*-4-{[Tris(propan-2-yl)silyl]oxy}thian-2-yl]methanol 11f

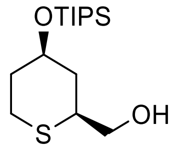

11f

Using general procedure A, TIPS-protected tetrahydrothiopyran **10b** (273 mg, 1.0 mmol, 1.0 eq.), *s*-BuLi (1.0 mL of a 1.3 M solution in 92/8 cyclohexane/hexane, 1.3 mmol, 1.3 eq.) and TMEDA (195 μL, 1.3 mmol, 1.3 eq.) in hexane (5 mL) at 0 °C for 2 h and paraformaldehyde (60 mg, 2.0 mmol, 2.0 eq.) at 0 °C for 1 h gave the crude product. Purification by flash column chromatography on silica with 90:10 hexane-CH_2Cl_2 then 47.5:47.5:5 hexane-CH_2Cl_2-MeOH as eluent gave alcohol **11f** (247 mg, 81%) as a colourless oil, R_F (90:10 hexane-CH_2Cl_2) 0.3; IR (ATR) 3392 (OH), 2940, 2865, 1463, 1368, 1255, 1104, 1067, 1013, 881, 827, 789, 678, 656 cm^{-1}; ^{1}H NMR (400 MHz, $CDCl_3$) δ 3.86-3.65 (m, 3H, OCH), 2.99-2.91 (m, 1H, CH), 2.89-2.79 (m, 1H, CH), 2.49 (ddd, J = 10.0, 10.0, 2.5 Hz, 1H, CH), 2.21-2.08 (m, 3H, OH, CH), 1.78-1.60 (m, 2H, CH), 1.05 (br s, 21H, $CHMe_2$); $^{13}C\{^1H\}$ NMR (100.6 MHz, $CDCl_3$) δ 69.3 (OCH), 66.2 (OCH_2), 44.0 (SCH) , 39.4 (CH_2), 36.5 (CH_2), 24.7 (CH_2), 18.23 (CH*Me*$_2$), 18.21 (CH*Me*$_2$), 12.4 (*CH**Me*$_2$); HRMS (ESI) *m/z* calcd for $C_{15}H_{32}O_2SSi$ $(M + Na)^+$ 327.1784, found 327.1795 (+3.2 ppm error).

Lab Book Reference: NS2-65

{[2,4-*cis*-2-Methylthian-4-yl]oxy}tris(propan-2-yl)silane 11g

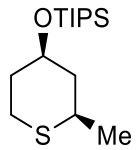

11g

Using general procedure A, TIPS-protected tetrahydrothiopyran **10b** (273 mg, 1.0 mmol, 1.0 eq.), *s*-BuLi (1.0 mL of a 1.3 M solution in 92/8 cyclohexane/hexane, 1.3 mmol, 1.3 eq.) and TMEDA (195 μL, 1.3 mmol, 1.3 eq.) in hexane (5 mL) at 0 °C for 2 h and Me_2SO_4 (189 μL, 2.0 mmol, 2.0 eq.) at 0 °C for 1 h gave the crude product. Purification by flash column chromatography on silica with 95:5 then 85:15 hexane-CH_2Cl_2 as eluent gave methyl tetrahydrothiopyran **11g** (118 mg, 41%) as a colourless oil, R_F (95:5 hexane-CH_2Cl_2) 0.4; IR (ATR) 2941, 2866, 1464, 1366, 1350, 1330, 1136, 1098, 847, 677 cm^{-1}; ^{1}H NMR (400 MHz, $CDCl_3$) δ 3.57 (dddd, J = 11.5, 11.5, 4.0, 4.0 Hz, 1H, OCH), 2.84 (dqd, J = 11.5, 7.0, 3.0 Hz, 1H, SC*H*Me), 2.72-2.58 (m, 2H, CH), 2.24-2.18 (m, 2H, CH), 1.60-1.49 (m, 1H, CH), 1.46-

1.35 (m, 1H, CH), 1.22 (d, J = 7.0 Hz, 3H, CH*Me*), 1.10-1.02 (m, 21H, $CHMe_2$); $^{13}C\{^1H\}$ NMR (100.6 MHz, $CDCl_3$) δ 71.2 (OCH), 46.9 (CH_2), 37.4 (SCH), 37.0 (CH_2), 28.0 (SCH_2), 21.7 (CH*Me*), 18.3 ($CHMe_2$), 12.5 (*C*HMe_2); HRMS (APCI) *m/z* calcd for $C_{15}H_{32}OSSi$ $(M + H)^+$ 275.1859, found 275.1862 (+1.1 ppm error).

Lab Book Reference: NS2-57

2,4-*cis*-4-{[Tris(propan-2-yl)silyl]oxy}thiane-2-carboxylic acid 11h

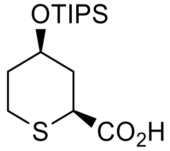

11h

Using general procedure B, TIPS-protected tetrahydrothiopyran **10b** (273 mg, 1.0 mmol, 1.0 eq.), *s*-BuLi (1.0 mL of a 1.3 M solution in 92/8 cyclohexane/hexane, 1.3 mmol, 1.3 eq.) and TMEDA (195 µL, 1.3 mmol, 1.3 eq.) in hexane (5 mL) at 0 °C for 2 h and CO_2 at 0 °C for 1 h gave the crude product. Purification by flash column chromatography on silica with 85:15 hexane-EtOAc then 75:25 hexane-EtOAc as eluent gave acid **11h** (63 mg, 20%) as a colourless oil, R_F (85:15 hexane-CH_2Cl_2) 0.2; IR (ATR) 2941, 2866, 1706 (C=O), 1463, 1261, 1197, 1083, 1065, 881, 734, 658, 679 cm^{-1}; ^1H NMR (400 MHz, $CDCl_3$) 10.04 (br s, 1H, CO_2H), 4.31-4.23 (m, 1H, OCH), 4.02 (dd, J = 9.0, 4.0 Hz, 1H, SCH), 3.06 (ddd, J = 9.0, 9.0, 6.0 Hz, 1H, CH), 2.54 (ddd, J = 13.5, 4.0, 4.0 Hz, 1H, CH), 2.21 (ddd, J = 13.5, 6.0, 4.0 Hz, 1H, CH), 2.12-2.03 (m, 1H, CH), 1.94-1.85 (m, 2H, CH), 1.11-1.00 (m, 21H, $CHMe_2$); $^{13}C\{^1H\}$ NMR (100.6 MHz, $CDCl_3$) δ 178.4 (C=O), 65.6 (OCH), 40.2 (SCH), 37.8 (CH_2), 34.6 (CH_2), 24.0 (CH_2), 18.2 ($CHMe_2$), 12.4 (*C*HMe_2); HRMS (ESI) *m/z* calcd for $C_{15}H_{30}O_3SSi$ $(M + Na)^+$ 341.1577, found 341.1572 (–1.4 ppm error).

Lab Book Reference: NS2-69

{[2,4-*cis*-2-(Prop-2-en-1-yl)thian-4-yl]oxy}tris(propan-2-yl)silane 11i

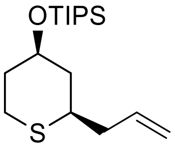

11i

Using general procedure A, TIPS-protected tetrahydrothiopyran **10b** (275 mg, 1.0 mmol, 1.0 eq.), *s*-BuLi (1.0 mL of a 1.3 M solution in 92/8 cyclohexane/hexane, 1.3 mmol, 1.3 eq.) and TMEDA (195 µL,

1.3 mmol, 1.3 eq.) in hexane (5 mL) at 0 °C for 2 h and allyl bromide (173 µL, 2.0 mmol, 2.0 eq.) at 0 °C for 1 h gave the crude product. Purification by flash column chromatography on silica with 95:5 hexane-CH_2Cl_2 as eluent gave allyl tetrahydrothiopyran **11i** (163 mg, 52%) as a colourless oil, R_F (90:10 hexane-CH_2Cl_2) 0.3; IR (ATR) 2940, 2865, 1463, 1078, 1062, 995, 881, 679, 663 cm^{-1}; ^1H NMR (400 MHz, $CDCl_3$) δ 5.88-5.73 (m, 1H, C*H*=CH_2), 5.10-5.03 (m, 2H, CH=C*H*$_2$), 4.24 (br s, 1H, OCH), 3.33-3.25 (m, 1H, SCH), 3.18 (ddd, *J* = 13.5, 13.5, 2.0 Hz, 1H, CH), 2.33 (ddd, *J* = 13.5, 3.5, 3.5 Hz, 1H, CH), 2.26-2.18 (m, 2H, CH), 2.00 (br d, *J* = 13.5 Hz, 1H, CH), 2.01-1.95 (m, 1H, CH), 1.78-1.68 (m, 1H, CH), 1.58-1.50 (m, 1H, CH), 1.08-1.03 (m, 21H, $CHMe_2$); $^{13}C\{^1H\}$ NMR (100.6 MHz, $CDCl_3$) δ 135.4 (*C*H=CH_2), 117.0 (CH=*C*H$_2$), 66.2 (OCH), 41.5 (*C*H$_2$CH=CH_2), 40.4 (CH_2), 35.8 (SCH), 34.6 (CH_2), 23.2 (SCH_2), 18.3 (CH*Me*$_2$), 12.4 (*C*HMe$_2$); HRMS (ESI) *m/z* calcd for $C_{17}H_{34}OSSi$ $(M + Na)^+$ 337.1985, found 337.1992 (–2.0 ppm error).

Lab Book Reference: NS2-54

Tris(propan-2-yl)({[2,4-*cis*-2-(tributylstannyl)thian-4-yl]oxy})silane 11j

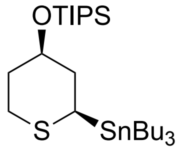

11j

Using general procedure A, TIPS-protected tetrahydrothiopyran **10b** (273 mg, 1.0 mmol, 1.0 eq.), *s*-BuLi (1.0 mL of a 1.3 M solution in 92/8 cyclohexane/hexane, 1.3 mmol, 1.3 eq.) and TMEDA (195 µL, 1.3mmol, 1.3 eq.) in hexane (5 mL) at 0 °C for 2 h and Bu_3SnCl (542 µL, 2.0 mmol, 2.0 eq.) at 0 °C for 1 h gave the crude product. Purification by flash column chromatography on silica with hexane then CH_2Cl_2 as eluent gave stannane **11j** (414 mg, 74%) as a colourless oil, R_F (90:10 hexane-CH_2Cl_2) 0.5; IR (ATR) 2955, 2924, 2865, 1463, 1098, 1068, 881, 828, 791, 679, 656 cm^{-1}; ^1H NMR (400 MHz, $CDCl_3$) δ 3.45 (dddd, *J* = 11.5, 11.5, 3.0, 3.0 Hz, 1H, OCH), 2.72 (ddd, *J* = 11.5, 11.5, 3.0 Hz, 1H, SCH), 2.59 (ddd, *J* = 11.5, 3.0, 3.0 Hz, 1H, SCH), 2.53 (dd, *J* = 11.5, 3.0 Hz, 1H, SCH), 2.29-2.19 (m, 2H, CH), 1.80-1.68 (m, 2H, CH), 1.58-1.47 (m, 6H, CH), 1.38-1.27 (m, 6H, CH), 1.08-1.04 (m, 21H, CH), 0.90 (m, 15H, CH); $^{13}C\{^1H\}$ NMR (100.6 MHz, $CDCl_3$) δ 72.7 (OCH), 42.6 (CH_2), 38.3 (CH_2), 32.2 (SCH_2), 29.3 (CH_2), 27.6 (CH_2), 24.3 (SCH), 18.2 ($CHMe_2$), 13.8 (CH$_2$*Me*), 12.5 (*C*HMe$_2$), 8.8 (CH_2); HRMS (ESI) *m/z* calcd for $C_{26}H_{56}OSSi^{120}Sn$ $(M + Na)^+$ 587.2738, found 587.2726 (+2.1 ppm error).

Lab Book Reference: NS2-59

Tris(propan-2-yl)({[2,4-*cis*-2-propylthian-4-yl]oxy})silane 12

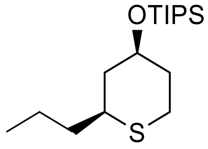

12

10% Pd/C (75 mg, 0.10 mmol, 10 mol%) was added to a stirred solution of allyl tetrahydrothiopyran **11i** (320 mg, 1.0 mmol, 1.0 eq.) in MeOH (5 mL) at rt. The reaction flask was evacuated under reduced pressure and back-filled with Ar three times. After a final evacuation, a balloon of H_2 was attached and the reaction mixture was stirred vigorously under a balloon of H_2 at rt for 3 h. The solids were removed by filtration through Celite® and washed with MeOH (10 mL). The filtrate was evaporated under reduced pressure to give propyl tetrahydrothiopyran **12** (318 mg, 98%) as a colourless oil, R_F (90:10 hexane-CH_2Cl_2) 0.5; IR (ATR) 2957, 2931, 2865, 1463, 1207, 1078, 1063, 881, 679, 662 cm^{-1}; ^1H NMR (400 MHz, $CDCl_3$) δ 4.27-4.20 (m, 1H, OCH), 3.27-3.20 (m, 1H, CH), 3.16 (ddd, J = 13.0, 13.0, 2.5 Hz, 1H, CH), 2.32 (ddd J = 13.0, 4.0, 4.0 Hz, 1H, CH), 2.08-1.94 (m, 2H, CH), 1.81-1.69 (m, 1H, CH), 1.54 (ddd, J = 13.0, 11.5, 2.5 Hz, 1H, SCH), 1.45-1.37 (m, 4H, CH), 1.07-1.03 (m, 21H, $CHMe_2$), 0.90 (t, J = 7.0 Hz, 3H, CH_2Me); $^{13}C\{^1H\}$ NMR (100.6 MHz, $CDCl_3$) δ 66.3 (OCH), 42.2 (CH_2), 38.3 (CH_2), 35.9 (SCH), 34.9 (CH_2), 23.2 (CH_2), 20.0 (CH_2), 18.3 ($CHMe_2$), 14.1 (CH_2Me), 12.4 ($CHMe_2$); HRMS (ESI) m/z calcd for $C_{17}H_{36}OSSi$ $(M + H)^+$ 317.2329, found 317.2326 (–0.8 ppm error).
Lab Book Reference: NS2-73

{[2,4,6-*cis,cis*-2-benzoyl-6-propylthian-4-yl]oxy}tris(propan-2-yl)silane 13

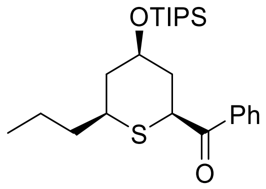

13

Using general procedure A, propyl tetrahydrothiopyran **12** (314 mg, 1.0 mmol, 1.0 eq.), *s*-BuLi (1.0 mL of a 1.3 M solution in 92/8 cyclohexane/hexane, 1.3 mmol, 1.3 eq.) and TMEDA (195 μL, 1.3 mmol, 1.3 eq.) in hexane (5 mL) at 0 °C for 4 h and *N*-methoxy-*N*-methylbenzamide (330 mg, 304 μL, 2.0 mmol, 2.0 eq.) at 0 °C for 1 h gave the crude product. Purification by flash column chromatography on silica with 75:25 hexane-CH_2Cl_2 then 50:50 hexane-CH_2Cl_2 as eluent gave ketone **13** (173 mg, 41%) as a colourless oil, R_F (50:50 hexane-CH_2Cl_2) 0.2; IR (ATR) 2941, 2865, 1683, 1463, 1448, 1271, 1077, 1059, 996, 980, 882, 684 cm^{-1}; ^1H NMR (400 MHz, $CDCl_3$) δ 8.04 (dd, J = 7.5, 1.0 Hz, 2H, Ph), 7.60-

7.54 (m, 1H, Ph), 7.46 (dd, *J* = 7.5, 7.5 Hz 2H, Ph), 5.10 (dd, *J* = 12.5, 2.5 Hz, 1H, SCHC(O)), 4.49-4.41 (m, 1H, OCH), 3.55-3.44 (m, 1H, CH), 2.26 (ddd, *J* = 12.5, 2.5, 2,5 Hz, 1H, SCH), 2.12 (ddd, *J* = 12.5, 2.5, 2.5 Hz, 1H, CH), 1.98 (ddd, *J* = 12.5, 12.5, 2.5 Hz, 1H, CH), 1.59-1.51 (m, 1H, CH), 1.51-1.40 (m, 4H, CH), 1.11-1.07 (m, 21H, $CHMe_2$), 0.92 (t, *J* = 7.0 Hz, 3H, CH_2*Me*); $^{13}C\{^1H\}$ NMR (100.6 MHz, $CDCl_3$) δ 198.5 (C=O), 135.3 (*ipso*-Ph), 133.5 (Ph), 129.0 (Ph), 128.7 (Ph), 66.8 (S*C*HC(O)), 43.3 (CH_2), 41.4 (S*C*HCH_2), 37.9 (CH_2), 37.1 (CH_2), 20.0 (CH_2), 18.3 ($CH\mathit{Me}_2$), 14.1 ($CH_2\mathit{Me}$), 12.4 (*C*HMe_2); HRMS (ESI) *m/z* calcd for $C_{24}H_{40}O_2SSi$ $(M + H)^+$ 421.2591, found 421.2589 (–0.4 ppm error).
Lab Book Reference: NS2-77

N-Methoxy-N,5-dimethylfuran-2-carboxamide S5

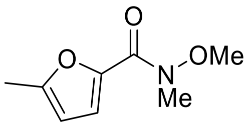

S5

5-Methylfuran-2-carbonyl chloride (2.00 g, 14 mmol, 1.0 eq.) was added to a stirred solution of *N*,*O*-dimethylhydroxylamine hydrochloride (1.35 g, 14 mmol, 1.0 eq.) and pyridine (2.46 mL, 31 mmol, 2.2 eq.) in CH_2Cl_2 (50 mL) at 0 °C under Ar. The resulting solution was allowed to warm to rt and stirred at rt for 16 h. Then, H_2O (50 mL) was added and the two layers were separated. The aqueous layer was extracted with EtOAc (3 × 30 mL). The combined organic layers were dried ($MgSO_4$) and evaporated under reduced pressure to give the crude product. Purification by flash column chromatography on silica with 50:50 hexane-EtOAc as eluent gave Weinreb amide **S5** (2.08 g, 89%) as a colourless oil, R_F (50:50 hexane-EtOAc) 0.3; IR (ATR) 3505, 2976, 1937, 1638 (C=O), 1516, 1413, 1378, 1029, 1007, 961, 861, 798, 742, 551 cm^{-1}; ^1H NMR (400 MHz, $CDCl_3$) δ 7.10-6.98 (m, 1H, Ar), 6.17-5.85 (m, 1H, Ar), 3.71 (s, 3H, OMe), 3.30 (s, 3H, NMe), 2.35 (s, 3H, Me); $^{13}C\{^1H\}$ NMR (100.6 MHz, $CDCl_3$) δ 159.4 (C=O), 156.1 (*ipso*-Ar), 144.2 (*ipso*-Ar), 118.9 (Ar), 108.3 (Ar), 61.3 (OMe), 33.3 (NMe), 14.0 (Me); HRMS (ESI) *m/z* calcd for $C_8H_{11}NO_3$ $(M + Na)^+$ 192.0631, found 192.0629 (+1.1 ppm error).
Lab Book Reference: NS10-95

2-Methyl-5-(thiolane-2-carbonyl)furan 7p

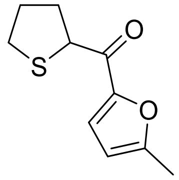

7p

Using general procedure A, tetrahydrothiophene **4** (587 µL, 6.7 mmol, 1.0 eq.), *s*-BuLi (6.66 mL of a 1.3 M solution in 92/8 cyclohexane/hexane, 8.7 mmol, 1.3 eq.) in hexane (33 mL) at 0 °C for 1 h and Weinreb amide **S5** (1.69 g, 10 mmol, 1.5 eq.) gave the crude product. Purification by flash column chromatography on silica with 90:10 hexane-Et_2O then 80:20 hexane-Et_2O as eluent gave ketone **7p** (941 mg, 72%) as a colourless oil, R_F (90:10 hexane-Et_2O) 0.2; IR (ATR) 2935, 2862, 1667 (C=O), 1513, 1440, 1370, 1266, 1207, 1065, 1027, 850, 803 cm^{-1}; ^{1}H NMR (400 MHz, $CDCl_3$) δ 7.10 (d, J = 3.5 Hz, 1H, Ar), 6.16-6.14 (m, 1H, Ar), 4.53 (dd, J = 7.0, 4.5 Hz, 1H, SCH), 3.00-2.85 (m, 2H, SCH), 2.51 (dddd, J = 12.5, 6.0, 6.0, 4.5 Hz, 1H, CH), 2.39 (s, 3H, Me), 2.31-2.18 (m, 1H, CH), 2.11 (dddd, J = 12.5, 6.5, 6.5, 1.5 Hz, 1H, CH), 1.95 (dddd, J = 12.5, 8.0, 6.5, 6.0 Hz, 1H, CH); $^{13}C\{^1H\}$ NMR (100.6 MHz, $CDCl_3$) δ 185.9 (C=O), 158.2 (*ipso*-Ar), 150.7 (*ipso*-Ar), 119.9 (Ar), 109.2 (Ar), 49.3 (SCH), 33.9 (SCH_2), 31.41 (CH_2), 31.39 (CH_2), 14.3 (Me); HRMS (ESI) *m/z* calcd for $C_{10}H_{12}O_2S$ $(M + Na)^+$ 219.0450, found 219.0448 (+0.9 ppm error). Spectroscopic data consistent with those reported in the literature.[11]
Lab Book Reference: NS10-100

(*S*)-2-Methyl-*N*-[(*E*)-(5-methylfuran-2-yl)[(2*S*)-thiolan-2-yl]methylidene]propane-2-sulfinamide 14a and (*S*)-2-Methyl-*N*-[(*E*)-(5-methylfuran-2-yl)[(2*R*)-thiolan-2-yl]methylidene]propane-2-sulfinamide 14b

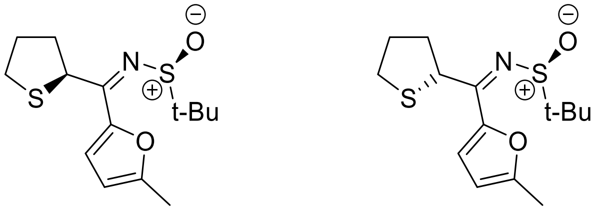

14a **14b**

$Ti(OEt)_4$ (218 µL, 233 mg, 1.0 mmol, 2.0 eq.) was added to a stirred solution of (*S*)-2-methylpropane-2-sulfinamide (93 mg, 0.51 mmol, 1.0 eq.) and ketone **7p** (100 mg, 0.51 mmol, 1.0 eq.) in toluene (5 mL) at rt under Ar. The resulting solution was stirred and heated at 50 °C for 48 h. Then, the solvent was evaporated under reduced pressure. CH_2Cl_2 (20 mL) and water (20 mL) were added and the solids were

removed by filtration through Celite®. The filtercake was washed with CH_2Cl_2 (50 mL). The two layers were separated and the organic layer was dried ($MgSO_4$) and evaporated under reduced pressure to give the crude product which contained a 50:50 mixture of sulfinyl imines **14a** and **14b** (by ^{1}H NMR spectroscopy). Purification by flash column chromatography on silica with 70:30 hexane-EtOAc as eluent gave sulfinyl imine **14b** (58 mg, 38%) as a colourless oil, $[\alpha]_D$ +90.9 (*c* 0.25 in $CHCl_3$); R_F (50:50 hexane-EtOAc) 0.5; IR (ATR) 2955, 2862, 1564, 1505, 1248, 1212, 1069, 1031, 793, 621 cm^{-1}; ^{1}H NMR (400 MHz, $CDCl_3$) δ 7.45 (d, J = 3.5 Hz, 1H, Ar), 6.18 (d, J = 3.5 Hz, 1H, Ar), 4.86 (br s, 1H, SCH), 2.99 (ddd, J = 10.5, 6.5, 6.5 Hz, 1H, SCH), 2.89 (ddd, J = 10.0, 6.5, 6.5 Hz, 1H, SCH), 2.57 (dddd, J = 12.0, 5.5, 5.5, 5.5 Hz, , 1H, CH), 2.38 (s, 3H, Me), 2.27 (ddddd, J = 13.0, 6.5, 6.5, 6.5, 6.5 Hz, 1H, CH), 2.12-1.94 (m, 2H, CH), 1.27 (s, 9H, C*Me*$_3$); ^{13}C{^{1}H} NMR (100.6 MHz, $CDCl_3$) δ 168.0 (C=N), 157.3 (Ar), 147.0 (Ar), 122.5 (Ar), 109.7 (Ar), 57.4 (S*C*Me$_3$), 51.0 (SCH), 33.9 (SCH_2), 33.0 (CH_2), 31.3 (CH_2), 22.4 (C*Me*$_3$), 14.2 (Me); HRMS (ESI) *m/z* calcd for $C_{14}H_{21}NO_2S_2$ $(M + Na)^+$ 322.0906, found 322.0900 (+1.8 ppm error) and sulfinamide **14a** (61.1 mg, 40%) as a colourless oil, $[\alpha]_D$ +140.8 (*c* 0.50 in $CHCl_3$); R_F (50:50 hexane-EtOAc) 0.4; IR (ATR) 2926, 2862, 1596, 1564, 1503, 1360, 1248, 1212, 1070, 1032, 793, 613 cm^{-1}; ^{1}H NMR (400 MHz, $CDCl_3$) δ 7.28 (d, J = 3.5 Hz, 1H, Ar), 6.20-6.16 (m, 1H, Ar), 4.88-4.73 (m, 1H, SCH), 3.01-2.85 (m, 2H, SCH), 2.72-2.59 (m, 1H, CH), 2.39 (s, 3H, Me), 2.31-2.18 (m, 1H, CH), 2.17-1.93 (m, 2H, CH), 1.28 (s, 9H, C*Me*$_3$); ^{13}C{^{1}H} NMR (100.6 MHz, $CDCl_3$) δ 168.0 (C=N), 157.3 (*ipso*-Ar), 131.0 (*ipso*-Ar), 122.5 (Ar), 109.7 (Ar), 57.4 (SCH), 50.9 (*C*Me$_3$), 33.9 (CH_2), 32.9 (SCH_2), 31.3 (CH_2), 22.4 (*C*Me$_3$), 14.2 (Me); HRMS (ESI) *m/z* calcd for $C_{14}H_{21}NO_2S_2$ $(M + Na)^+$ 322.0906, found 322.0902 (+1.2 ppm error).

Lab Book Reference: NS11-23

(*S*)-2-Methyl-*N*-[(*S*)-(5-methylfuran-2-yl)[(2*R*)-thiolan-2-yl]methyl]propane-2-sulfinamide S6

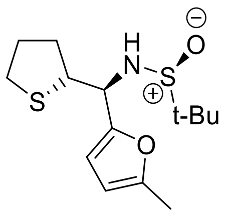

S6

9-BBN (1.0 mL of a 0.5 M solution in THF, 0.50 mmol, 2.0 eq.) was added to stirred a solution of sulfinyl imine **14b** (75 mg, 0.25 mmol, 1.0 eq.) in THF (5 mL) at 0 °C under Ar. The resulting solution was stirred at 0 °C for 4 h. Then, MeOH (5 mL) was added and the solvent was evaporated under reduced pressure to give the crude product. Purification by flash column chromatography on silica with 90:10

hexane-EtOAc to 70:30 hexane-EtOAc as eluent gave sulfinamide **S6** (69 mg, 92%) as a colourless oil, $[\alpha]_D$ +54.2 (*c* 1.00 in $CHCl_3$); R_F (70:30 hexane-EtOAc) 0.3; IR (ATR) 3222 (NH), 2950, 2863, 1561, 1442, 1364, 1222, 1051, 1021, 903, 784, 685, 594 cm^{-1}; ^1H NMR (400 MHz, $CDCl_3$) δ 6.16-6.12 (m, 1H, Ar), 5.93-5.85 (m, 1H, Ar), 4.47-4.40 (m, 1H, NCH), 3.91 (d, J = 4.5 Hz, 1H, NH), 3.90 -3.78 (m, 1H, SCH), 2.89-2.77 (m, 2H, SCH), 2.25 (s, 3H, Me), 2.16-1.83 (m, 4H, CH), 1.19 (s, 9H, CMe_3); $^{13}C\{^1H\}$ NMR (100.6 MHz, $CDCl_3$) δ 152.0 (*ipso*-Ar), 151.4 (*ipso*-Ar), 109.3 (Ar), 106.3 (Ar), 56.8 (NCH), 56.2 (*C*Me_3), 53.0 (SCH), 33.2 (CH_2), 33.1 (SCH_2), 31.1 (CH_2), 22.6 (C*Me_3*), 13.7 (Me); HRMS (ESI) *m/z* calcd for $C_{14}H_{23}NO_2S_2$ $(M + Na)^+$ 324.1062, found 324.1062 (+0.2 ppm error).
Lab Book Reference: NS11-21

(1*S*)-1-(5-Methylfuran-2-yl)-1-[(2*R*)-thiolan-2-yl]methanamine hydrochloride 15·HCl

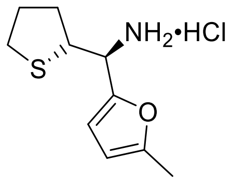

15

HCl (175 µL of a 2.0 M solution in Et_2O, 0.35 mmol, 3.0 eq.) was added to a stirred solution of sulfinamide **S6** (35 mg, 0.12 mmol, 1.0 eq.) in MeOH (2 mL) at rt. After stirring at rt for 1 h, the solvent was evaporated under reduced pressure to give the crude solid. Et_2O (3 mL) was added, the mixture was swirled to dissolve impurities and then the Et_2O was decanted. Addition of Et_2O (3 mL) and decanting was repeated a second time. The residual Et_2O was evaporated under reduced pressure to give amine **15**·HCl (27 mg, 96%) as a white solid, $[\alpha]_D$ −34.6 (*c* 1.00 in $CHCl_3$); mp 160-162 °C; IR (ATR) 3400 (NH), 2863, 2599, 1601, 1561, 1508, 1444, 1229, 1022, 788 cm^{-1}; ^1H NMR (400 MHz, d_6-DMSO) δ 8.68 (s, 3H, NH), 6.42 (d, J = 3.5 Hz, 1H, Ar), 6.23-5.92 (m, 1H, Ar), 4.26 (dd, J = 9.5, 4.5 Hz, 1H, SCH), 3.89 (ddd, J = 9.5, 6.0, 6.0 Hz, 1H, NCH), 2.82-2.69 (m, 1H, SCH), 2.26 (s, 3H, Me), 2.15-2.05 (m, 2H, SCH), 2.04-1.83 (m, 4H, CH); $^{13}C\{^1H\}$ NMR (100.6 MHz, d_6-DMSO) δ 151.9 (*ipso*-Ar), 148.5 (*ipso*-Ar), 110.6 (Ar), 106.9 (Ar), 52.2 (NCH), 49.4 (SCH), 33.4 (CH_2), 31.9 (SCH_2), 30.1 (CH_2), 13.3 (Me); HRMS (ESI) *m/z* calcd for $C_{10}H_{15}NOS$ $(M + Na)^+$ 220.0767, found 220.0768 (−0.9 ppm error). The enatiomeric ratio of amine **15**·HCl was 98:2 er as determined by conversion to **S7** and CSP-HPLC in comparison with racemic **S7**. Spectroscopic data consistent with those reported in the literature.[11]
Lab Book Reference: NS11-25

N-[(S)-(5-Methylfuran-2-yl)[(2R)-thiolan-2-yl]methyl]benzamide S7

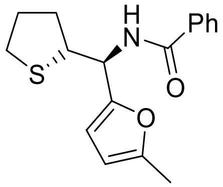

S7

Benzoyl chloride (54 µL, 0.46 mmol, 4.0 eq.) was added to a stirred solution of amine **15**·HCl (27 mg, 0.125 mmol, 1.0 eq.) and Et_3N (64 µL, 0.46 mmol, 4.0 eq.) in CH_2Cl_2 (2 mL) at 0 °C under Ar. The resulting solution was stirred at rt for 24 h. Then, CH_2Cl_2 (10 mL) and water (10 mL) were added and the two layers were separated. The aqueous layer was extracted with CH_2Cl_2 (3 × 10 mL). The combined organic layers were washed with 1 M $HCl_{(aq)}$ (30 mL), dried ($MgSO_4$) and evaporated under reduced pressure to give the crude product. Purification by flash column chromatography on silica with 80:20 hexane-EtOAc then 70:30 hexane-EtOAc as eluent gave amide **S7** (29 mg, 87%, 97:3 er) as a white solid, mp 118-120 °C, $[\alpha]_D$ +14.7 (*c* 0.25 in $CHCl_3$); R_F (70:30 hexane-EtOAc) 0.5; IR (ATR) 3301 (NH), 2949, 1635 (C=O), 1530, 1489, 1444, 1326, 1222, 1025, 786, 695 cm^{-1}; ^1H NMR (400 MHz, $CDCl_3$) δ 7.80-7.74 (m, 2H, Ph), 7.54-7.46 (m, 1H, Ph), 7.45-7.39 (m, 2H, Ph), 6.52 (d, J = 9.0 Hz, 1H, NH), 6.19 (d, J = 3.0 Hz, 1H, Ar), 5.90-5.88 (m, 1H, Ar), 5.31 (dd, J = 9.0 Hz, 1H, NCH), 3.91-3.80 (m, 1H, SCH), 2.91-2.77 (m, 2H, SCH), 2.26 (s, 3H, Me), 2.12-1.90 (m, 4H, CH); $^{13}C\{^1H\}$ NMR (100.6 MHz, $CDCl_3$) δ 166.7 (C=O), 151.9 (*ipso*-Ar), 151.4 (*ipso*-Ar), 134.4 (*ipso*-Ph), 131.8 (Ph), 128.7 (Ph), 127.2 (Ph), 108.6 (Ar), 106.4 (Ar), 52.3 (NCH), 52.2 (SCH), 33.9 (CH_2), 32.9 (SCH_2), 30.2 (CH_2), 13.7(Me); HRMS (ESI) *m/z* calcd for $C_{17}H_{19}NO_2S$ $(M + H)^+$ 324.1029, found 324.1025 (+1.2 ppm error); CSP-HPLC: Chiralcel AD-H (90:10 hexane-*i*-PrOH, 1.0 mL min^{-1}) t_R = 15.1 min (major), 20.4 min (minor).
Lab Book Reference: NS11-28

2-(Thiolan-2-yl)furan 7q

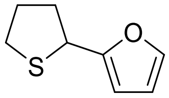

7q

n-BuLi (261 µL of a 2.3 M solution in 92/8 cyclohexane/hexane, 0.60 mmol, 1.2 eq.) was added dropwise to a stirred solution of furan (44 µL, 41 mg, 0.60 mmol, 1.2 eq.) in THF (2 mL) at −78 °C under Ar. The resulting solution was allowed to warm to rt and stirred at rt for 1 h. Then, the solution was cooled to −78 °C and a solution of boronic ester **7n** (1.0 mL of a 0.5 M solution in THF, 0.50 mmol,

1.0 eq.) was added dropwise. The resulting solution was stirred at −78 °C for 1 h. Then, a solution of NBS (2.0 mL of a 0.6 M solution in THF, 1.2 mmol, 2.4 eq.) was added dropwise and the resulting solution was stirred at −78 °C for 1 h. Saturated $Na_2S_2O_{3(aq)}$ (2 mL) was added and the mixture was stirred at −78 °C for 5 min and then allowed to warm to rt. Et_2O (10 mL) and H_2O (15 mL) were added and the two layers were separated. The aqueous layer was extracted with Et_2O (2 × 10 mL). The combined organic layers were dried ($MgSO_4$) and evaporated under reduced pressure to give the crude product. Purification by flash column chromatography on silica with 95:5 hexane-CH_2Cl_2 as eluent gave α-furanyl sulfide **7q** (32 mg, 42%) as a colourless oil, R_F (95:5 hexane-CH_2Cl_2) 0.4; IR (ATR) 2925, 2855, 1739, 1504, 1442, 1262, 1073, 1009, 804, 733 cm^{-1}; ^{1}H NMR (400 MHz, $CDCl_3$) δ 7.37-7.30 (m, 1H, Ar), 6.28 (dd, J = 3.5, 2.0 Hz, 1H, Ar), 6.17 (d, J = 3.5 Hz, 1H, Ar), 4.55 (dd, J = 6.0, 6.0 Hz, 1H, SCH), 3.08 (ddd, J = 10.0, 7.0, 6.5 Hz, 1H, SCH), 3.08 (ddd, J = 10.0, 5.5, 5.5 Hz, 1H, SCH), 2.31-2.12 (m, 3H, CH), 2.11-1.97 (m, 1H, CH); ^{13}C{^{1}H} NMR (100.6 MHz, $CDCl_3$) δ 156.3 (*ipso*-Ar), 142.0 (Ar), 110.3 (Ar), 105.8 (Ar), 44.6 (SCH), 36.6 (CH_2), 33.2 (CH_2), 30.8 (CH_2); HRMS (APCI) *m/z* calcd for $C_8H_{10}OS$ (M + H)$^+$ 155.0525, found 155.0519 (+3.9 ppm error). Spectroscopic data consistent with those reported in the literature.[12]

Lab Book Reference: NS5-84

1-Methyl-2-(thiolan-2-yl)-1H-indole 7r

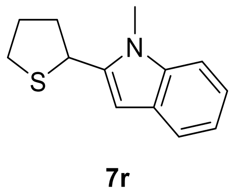

7r

n-BuLi (173 µL of a 2.3 M solution in 92/8 cyclohexane/hexane, 0.40 mmol, 1.6 eq.) was added dropwise to a stirred solution of *N*-methyl indole (50 µL, 52 mg, 0.40 mmol, 1.6 eq.) in THF (2 mL) at rt under Ar. The resulting solution was stirred and heated at reflux for 3 h. Then, the solution was cooled to −78 °C and a solution of boronic ester **7n** (1 mL of a 0.25 M solution in THF, 0.25 mmol, 1.0 eq.) was added dropwise. The resulting solution was stirred at −78 °C for 1 h. Then, a solution of NBS (1 mL of a 0.6 M solution in THF, 0.60 mmol, 2.4 eq.) was added dropwise and the resulting solution was stirred at −78 °C for 1 h. Saturated $Na_2S_2O_{3(aq)}$ (2 mL) was added and the mixture was stirred at −78 °C for 5 min and then allowed to warm to rt. EtOAc (10 mL) and H_2O (15 mL) were added and the two layers were separated. The aqueous layer was extracted with EtOAc (2 × 10 mL). The combined organic layers were dried (MgSO4) and evaporated under reduced pressure to give the crude product. Purification by

flash column chromatography on silica with 99:1 hexane-Et_2O as eluent gave 2-indolyl sulfide **7r** (21 mg, 38%) as a colourless oil, R_F (50:50 hexane-EtOAc) 0.8; IR (ATR) 3050, 2925, 2855, 1548, 1471, 1329, 1233, 1013, 761, 738 cm^{-1}; ^{1}H NMR (400 MHz, $CDCl_3$) δ 7.69 (d, J = 7.5 Hz, 1H, Ar), 7.28 (d, J = 7.5 Hz, 1H, Ar), 7.22 (dd, J = 7.5, 7.5 Hz, 1H, Ar), 7.12 (dd, J = 7.5, 7.5 Hz, 1H, Ar), 7.06 (s, 1H, Ar), 4.85 (dd, J = 7.5, 7.5 Hz, 1H, SCH), 3.74 (s, 3H, NMe), 3.15 (ddd, J = 10.5, 7.5, 6.5 Hz, 1H, SCH), 3.01 (ddd, J = 10.5, 7.5, 5.0 Hz, 1H, SCH), 2.44-2.34 (m, 1H, CH), 2.30-1.99 (m, 3H, CH); ^{13}C{^{1}H} NMR (100.6 MHz, $CDCl_3$) δ 137.6 (*ipso*-Ar), 126.9 (*ipso*-Ar), 126.6 (Ar), 121.9 (Ar), 119.6 (Ar), 119.0 (Ar), 116.7 (*ipso*-Ar), 109.5 (Ar), 44.5 (SCH_2), 38.7 (SCH), 33.0 (NMe), 32.8 (CH_2), 30.8 (CH_2); HRMS (ESI) *m/z* calcd for $C_{13}H_{15}NS$ $(M + H)^+$ 218.0998, found 218.1005 (–3.3 ppm error). Spectroscopic data consistent with those reported in the literature.[12]

Lab Book Reference: NS6-48

4-(Thiolan-2-yl)pyridine 7s

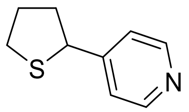

7s

Using general procedure C, Ir(*p*-F-ppy)$_3$ (2.1 mg, 2.4 µmol, 0.02 eq.), carboxylic acid **7h** (48 mg, 0.36 mmol, 3.0 eq.), CsF (55 mg, 0.40 mmol, 3.0 eq.) and 4-cyanopyridine (12.5 mg, 0.12 mmol, 1.0 eq.) in DMSO (6 mL) gave the crude product. Purification by flash column chromatography on silica with 70:30 hexane-EtOAc as eluent gave α-aryl sulfide **7s** (14 mg, 69%) as a colourless oil, R_F (70:30 hexane-EtOAc) 0.2; IR (ATR) 2949, 1597, 1441, 1412, 993, 810, 543 cm^{-1}; ^{1}H NMR (400 MHz, $CDCl_3$) δ 8.53-8.50 (m, 2H, Ar), 7.35-7.31 (m, 2H, Ar), 4.44 (dd, J = 8.0, 6.0 Hz, 1H, SCH), 3.14 (ddd, J = 10.5, 8.5, 6.5 Hz, 1H, SCH), 3.02 (ddd, J = 10.5, 6.5, 4.5 Hz, 1H, SCH), 2.41 (dddd, J = 11.0, 8.5, 8.0, 5.0 Hz, 1H, CH), 2.22 (dddd, J = 11.0, 8.5, 8.0, 6.0 Hz, 1H, CH), 2.07-1.97 (m, 1H, CH), 1.95-1.85 (m, 1H, CH); ^{13}C{^{1}H} NMR (100.6 MHz, $CDCl_3$) δ 152.7 (*ipso*-Ar), 149.9 (Ar), 123.0 (Ar), 51.5 (SCH), 40.1 (CH_2), 33.7 (CH_2), 31.1 (CH_2); HRMS (ESI) *m/z* calcd for $C_9H_{11}NS$ $(M + H)^+$ 166.0685, found 166.0685 (–0.1 ppm error).

Lab Book Reference: NS4-83

4-(Thiolan-2-yl)benzonitrile 7t

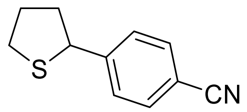

7t

Using general procedure C, Ir(p-F-ppy)$_3$ (2.1 mg, 2.4 μmol, 0.02 eq.), carboxylic acid **7h** (48 mg, 0.36 mmol, 3.0 eq.), CsF (55 mg, 0.4 mmol, 3.0 eq.) and 1,4-dicyanobenzene (17 mg, 0.12 mmol, 1.0 eq.) in DMSO (6 mL) gave the crude product. Purification by flash column chromatography on silica with 95:5 hexane-EtOAc as eluent gave α-aryl sulfide **7t** (16 mg, 71%) as a colourless oil, R_F (95:5 hexane-EtOAc) 0.2; IR (ATR) 2949, 2862, 2227 (CN), 1606, 1502, 1414, 1264, 910, 839, 822, 731, 560 cm^{-1}; ^{1}H NMR (400 MHz, $CDCl_3$) δ 7.58 (d, J = 8.5 Hz, 2H, Ar), 7.52 (d, J = 8.5 Hz, 2H, Ar), 4.51 (dd, J = 8.0, 6.5 Hz, 1H, SCH), 3.17 (ddd, J = 10.0, 8.5, 6.5 Hz, 1H, SCH), 3.03 (ddd, J = 10.0, 8.5, 6.5 Hz, 1H, SCH), 2.42 (dddd, J = 11.5, 8.5, 8.0, 5.0 Hz, 1H, CH), 2.25 (dddd, J = 10.0, 8.5, 8.0, 5.0 Hz, 1H, CH), 2.09-1.96 (m, 1H, CH), 1.94-1.83 (m, 1H, CH); ^{13}C{^{1}H} NMR (100.6 MHz, $CDCl_3$) δ 149.0 (*ipso*-Ar), 132.3 (Ar), 128.6 (Ar), 119.0 (*ipso*-Ar) , 110.8 (CN), 52.3 (SCH), 40.6 (CH_2), 33.8 (CH_2), 31.2 (CH_2); HRMS (ESI) *m/z* calcd for $C_{11}H_{11}NS$ (M + H)$^+$ 190.0685, found 190.0688 (–1.7 ppm error). Spectroscopic data consistent with those reported in the literature.[12]

Lab Book Reference: NS4-75

4-(Thian-2-yl)pyridine 8m

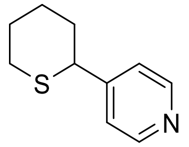

8m

Using general procedure C, Ir(p-F-ppy)$_3$ (3 mg, 3.4 μmol, 0.02 eq.), carboxylic acid **8f** (75 mg, 0.51 mmol, 3.0 eq.), CsF (78 mg, 0.51 mmol, 3.0 eq.) and 4-cyanopyridine (18 mg, 0.17 mmol, 1.0 eq.) in DMSO (6 mL) gave the crude product. Purification by flash column chromatography on silica with 70:30 hexane-EtOAc as eluent gave α-aryl sulfide **8m** (12 mg, 39%) as a white solid, mp 84-86 °C, R_F (70:30 hexane-EtOAc) 0.2; IR (ATR) 2930, 2856, 2812, 2765, 1594, 1438, 1355, 1264, 1150, 1035, 911, 803, 609, 539 cm^{-1}; ^{1}H NMR (400 MHz, $CDCl_3$) δ 8.54 (br d, J = 3.5 Hz, 2H, Ar), 7.29-7.24 (m, 2H, Ar), 3.81 (dd, J = 11.5, 2.5 Hz, 1H, SCH), 2.87 (ddd, J = 13.0, 12.5, 3.0 Hz, 1H, SCH), 2.69 (ddd, J = 13.0, 3.0, 3.0 Hz, 1H, SCH), 2.13 (ddd, J = 13.0, 7.0, 3.5 Hz, 1H, CH), 2.07-1.97 (m, 2H, CH), 1.90 (dddd, J = 13.0, 13.0, 11.5, 3.5 Hz, 1H, CH), 1.75-1.61 (m, 1H, CH), 1.55-1.42 (m, 1H, CH); ^{13}C{^{1}H} NMR

(100.6 MHz, $CDCl_3$) δ 151.7 (*ipso*-Ar), 150.1 (Ar), 122.8 (Ar), 46.3 (SCH), 34.4 (CH_2), 30.6 (CH_2), 26.7 (CH_2), 26.6 (CH_2); HRMS (ESI) *m/z* calcd for $C_{10}H_{13}NS$ $(M + H)^+$ 180.0841, found 180.0843 (–0.8 ppm error).

Lab Book Reference: NS5-26

2. ^{1}H and ^{13}C NMR Spectra

400 MHz ^{1}H NMR spectrum; 100.6 MHz ^{13}C NMR spectrum; $CDCl_3$ of **7a**

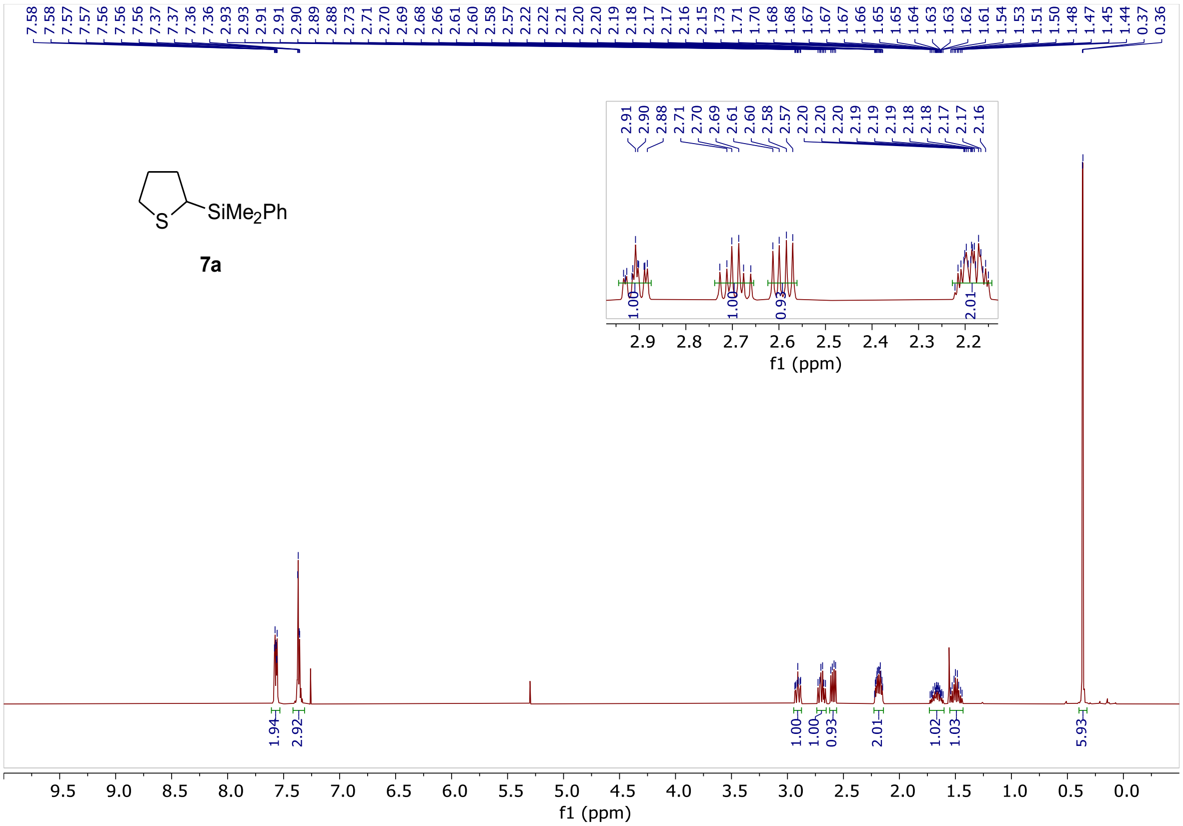

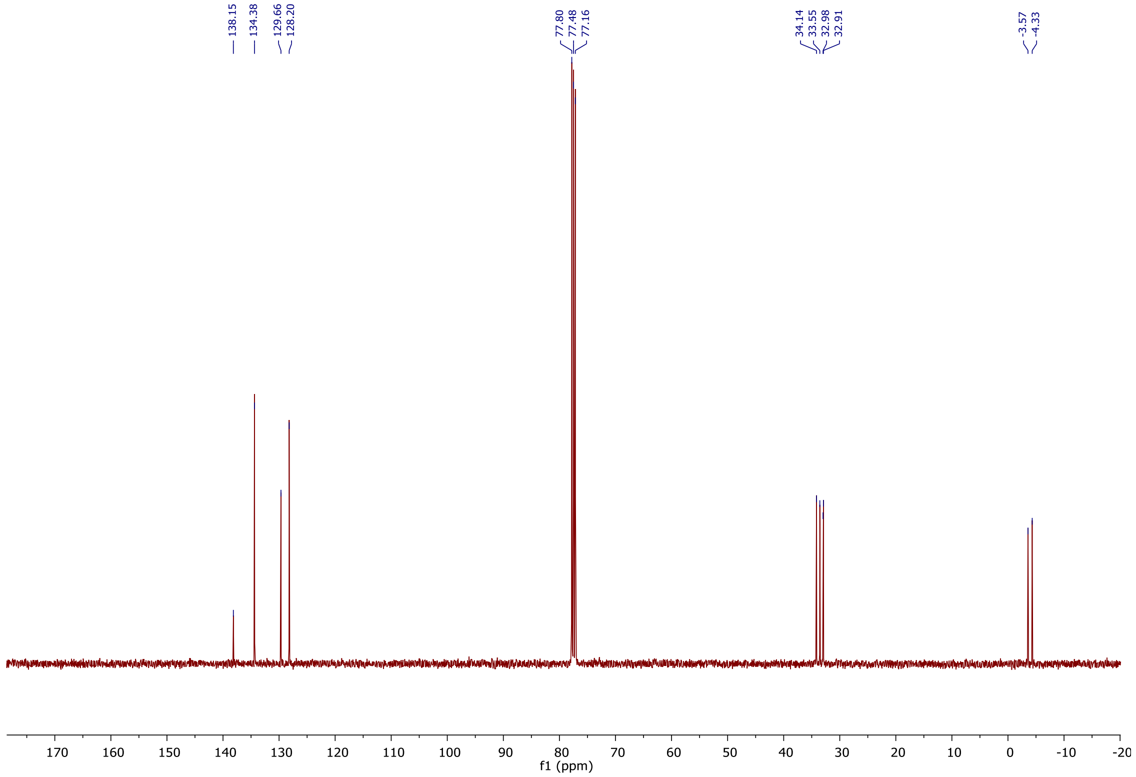

400 MHz ^{1}H NMR spectrum; 100.6 MHz ^{13}C NMR spectrum; $CDCl_3$ of **7b**

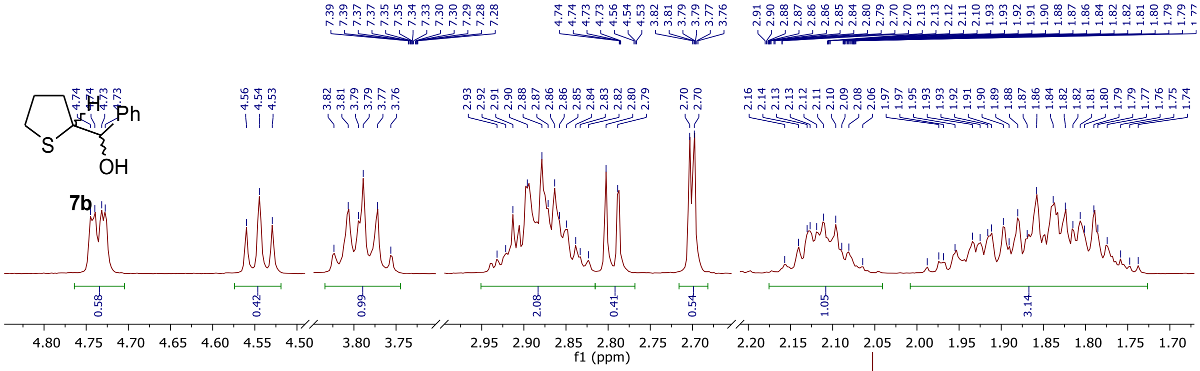

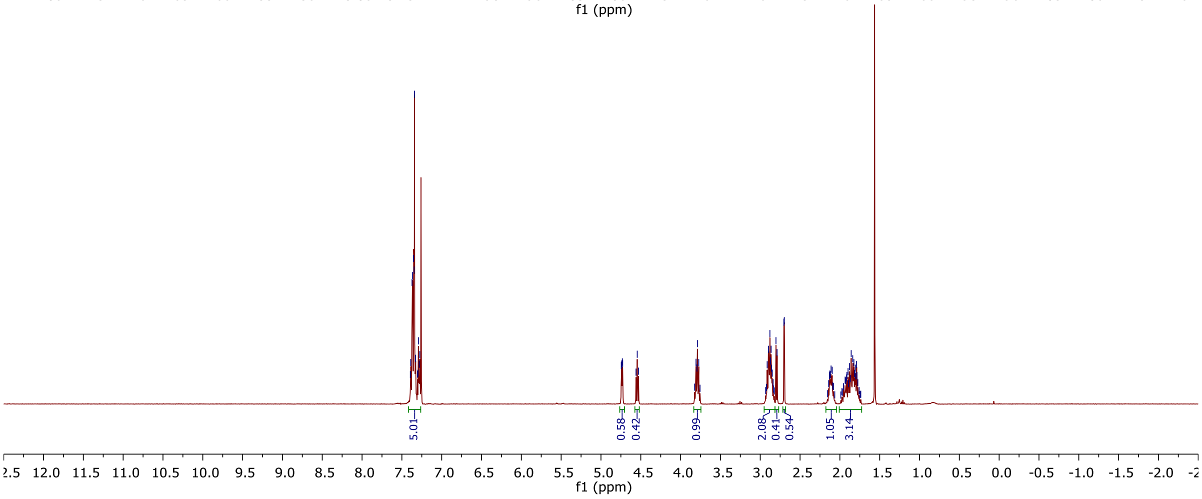

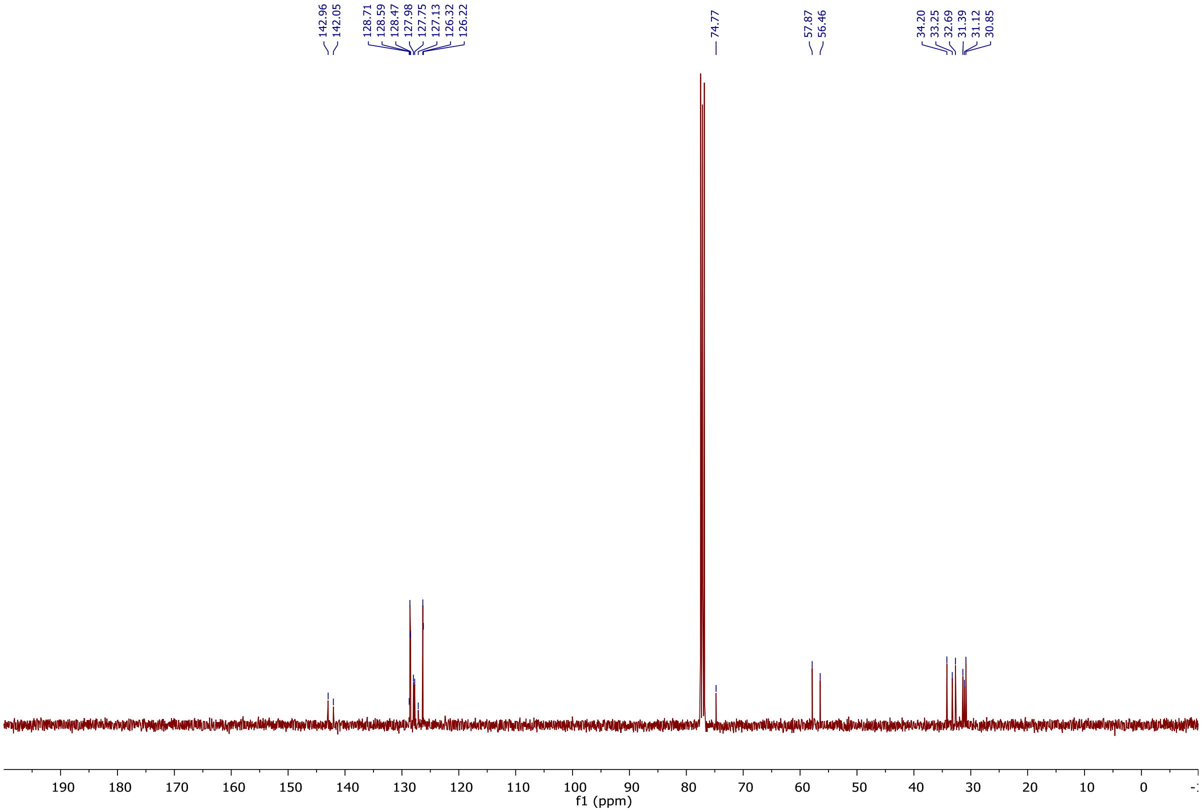

400 MHz ^{1}H NMR spectrum; 100.6 MHz ^{13}C NMR spectrum; $CDCl_3$ of **7c**

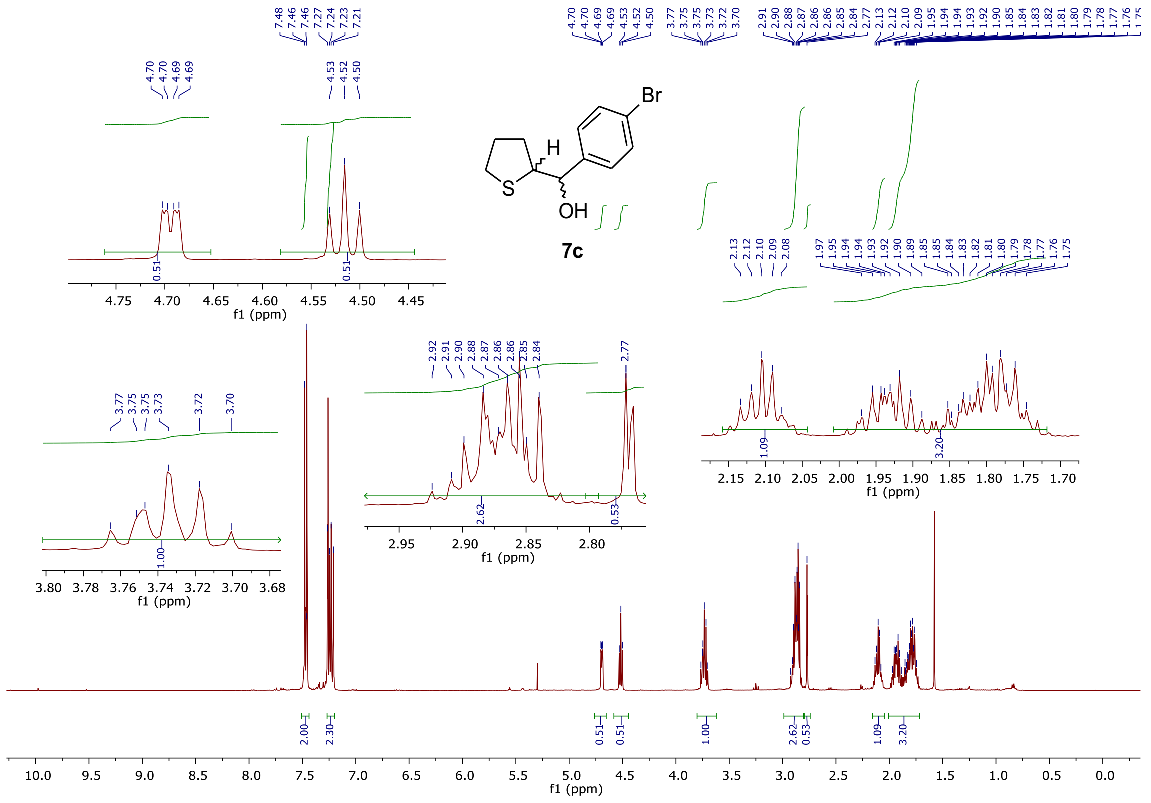

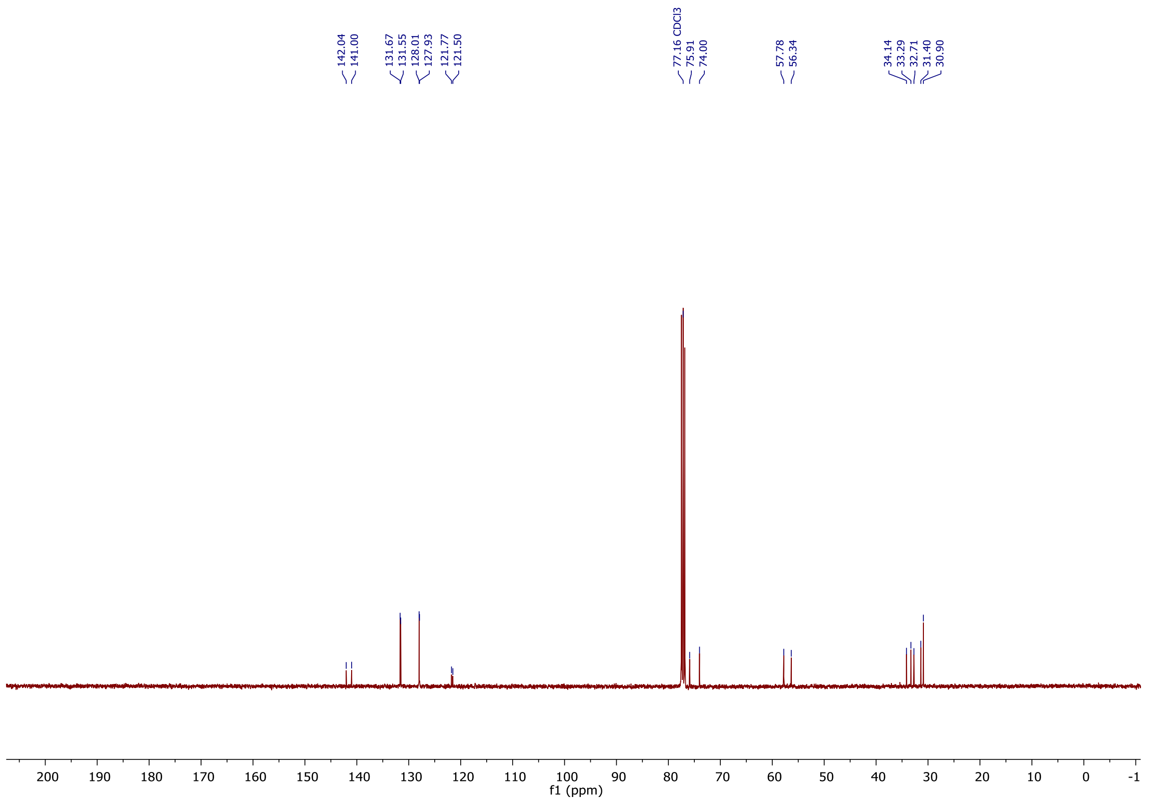

400 MHz ^{1}H NMR spectrum; 100.6 MHz ^{13}C NMR spectrum; $CDCl_3$ of **7d**

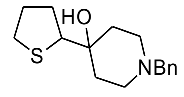

7d

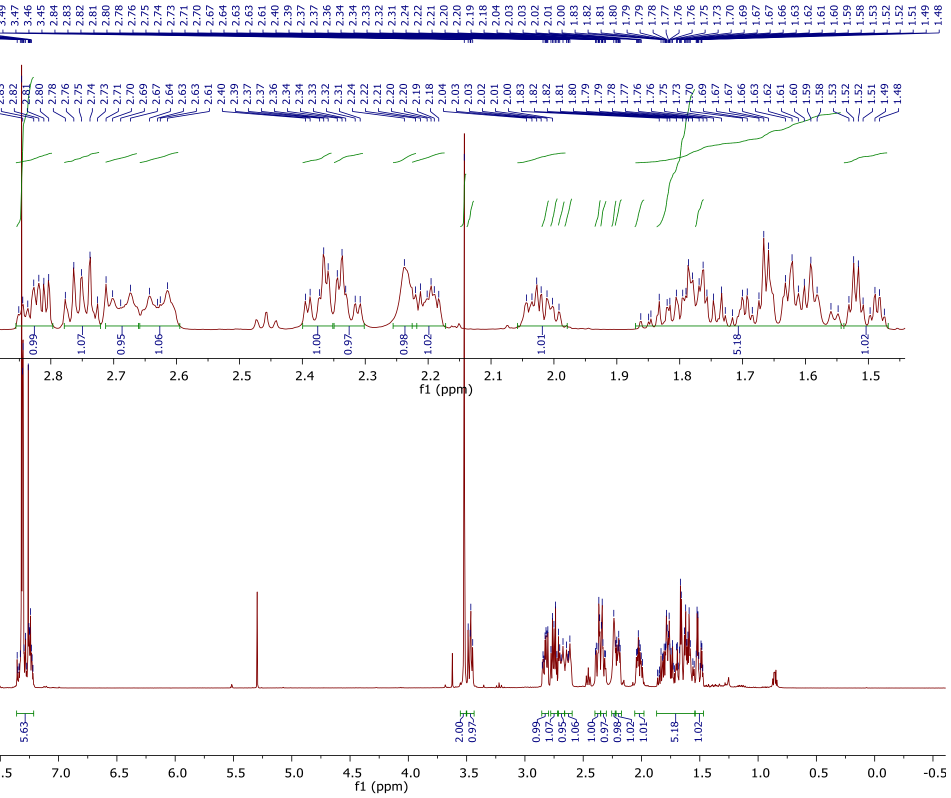

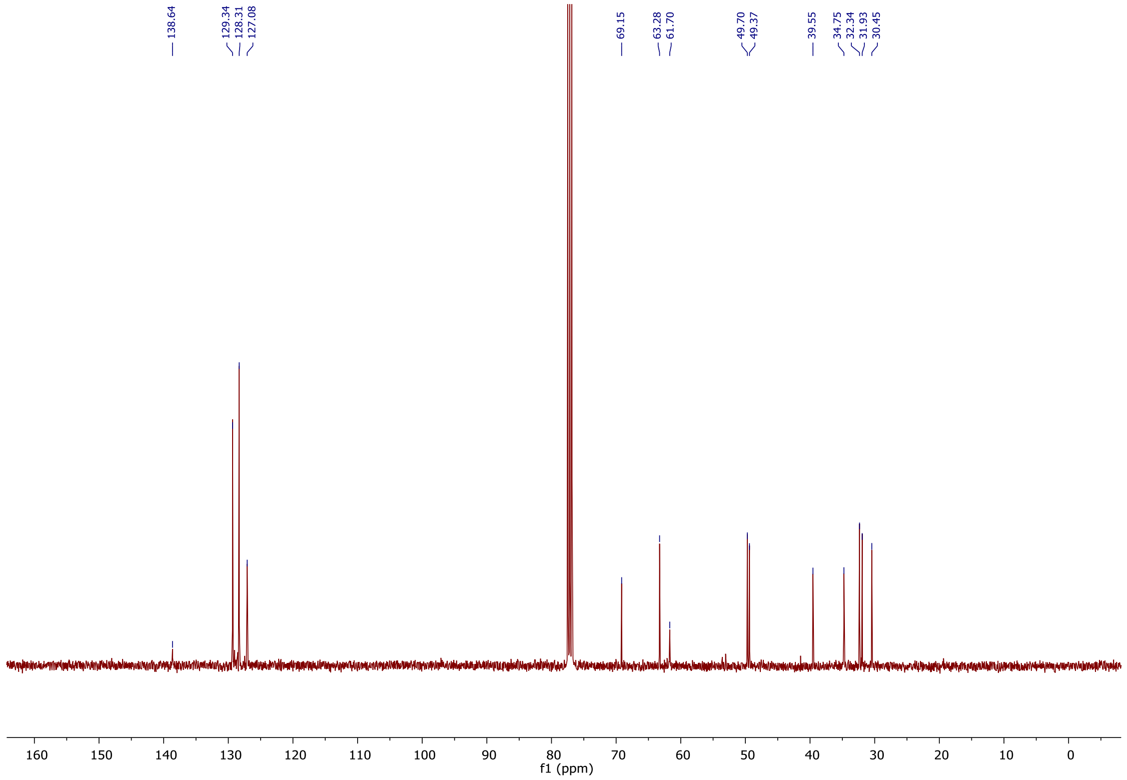

400 MHz ^1H NMR spectrum; 100.6 MHz ^{13}C NMR spectrum; $CDCl_3$ of **7e**

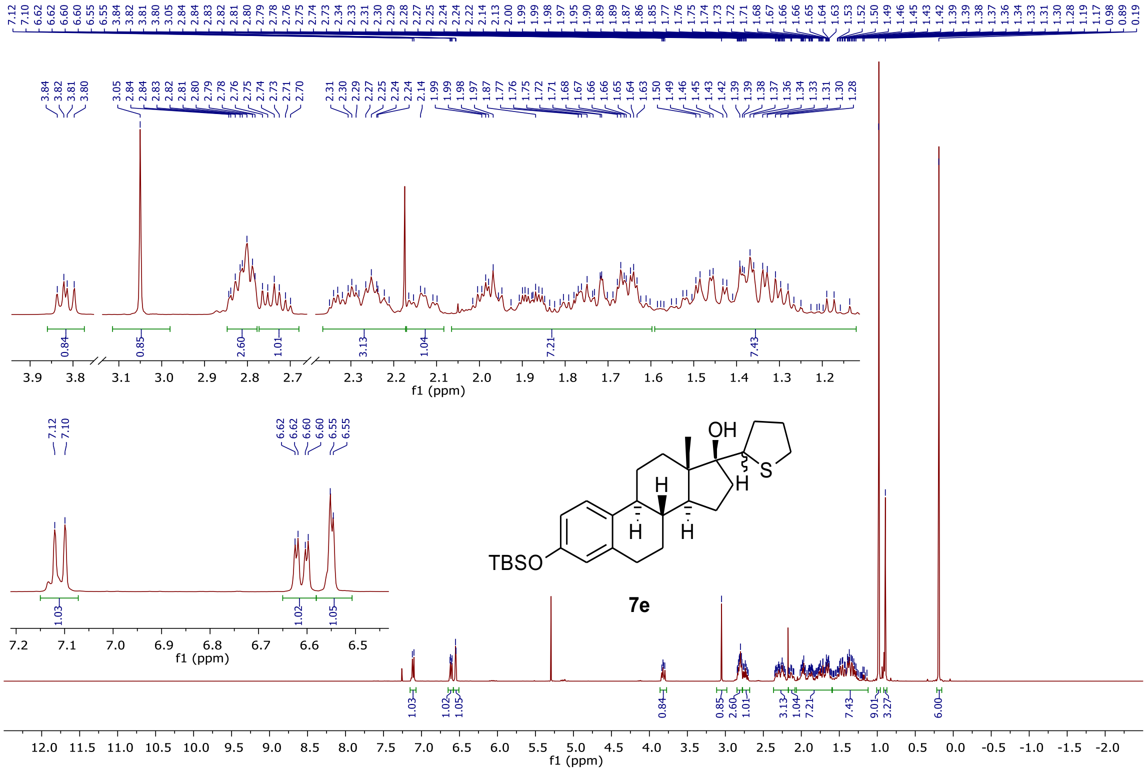

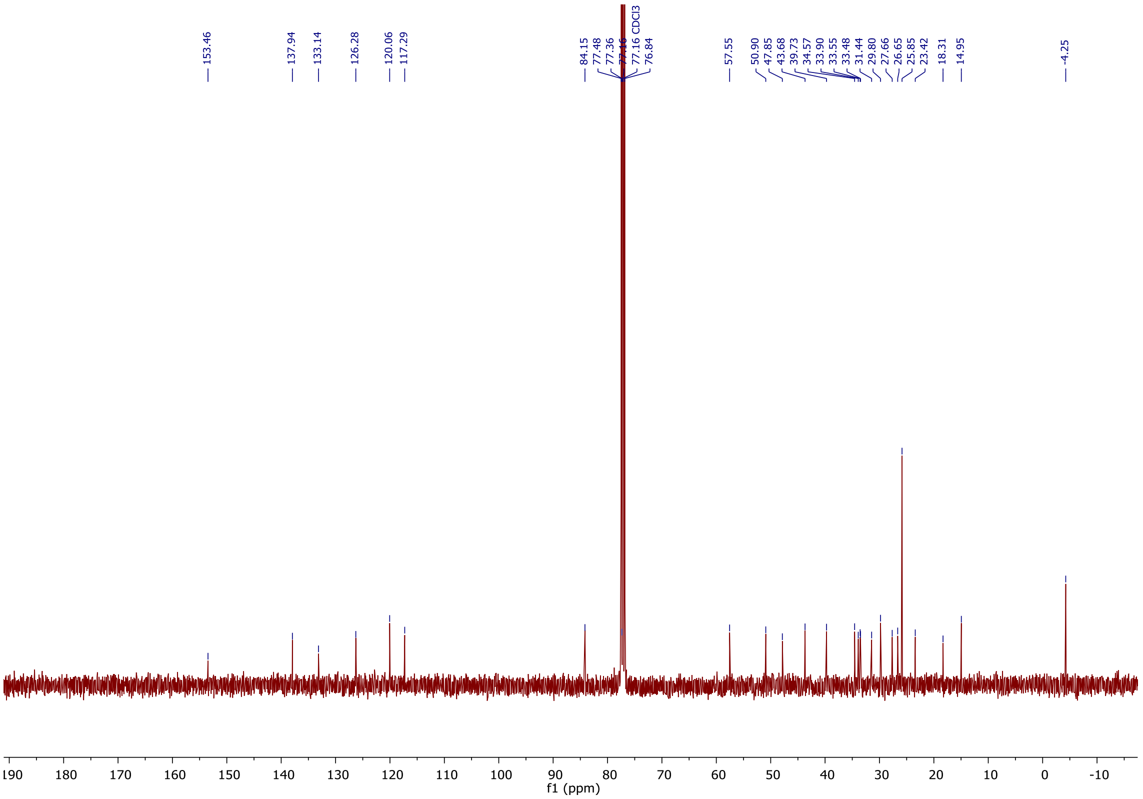

400 MHz ^{1}H NMR spectrum; 100.6 MHz ^{13}C NMR spectrum; $CDCl_3$ of **7f**

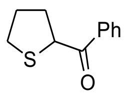

7f

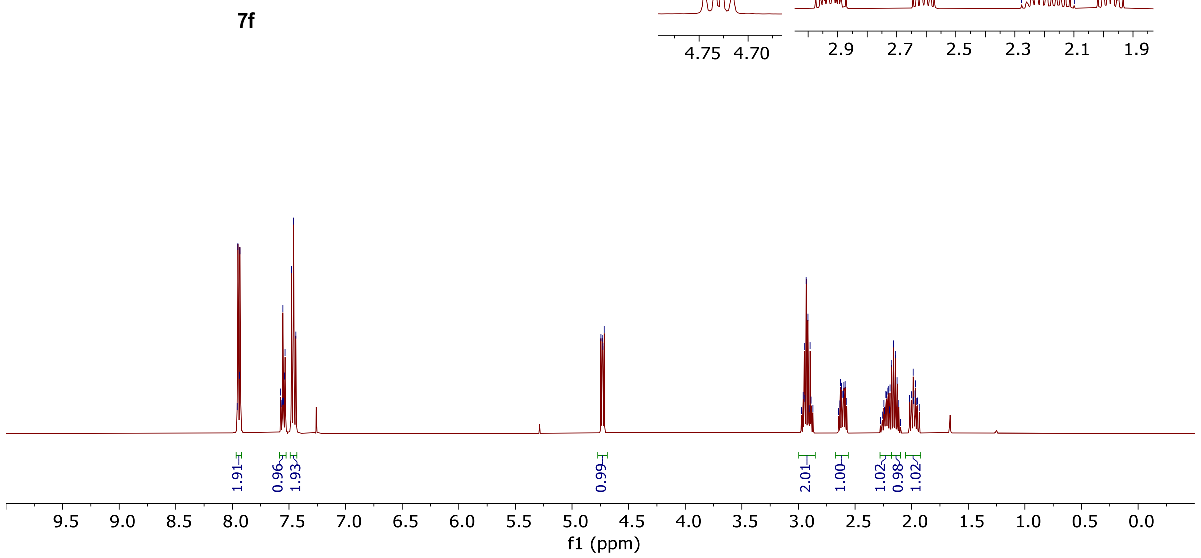

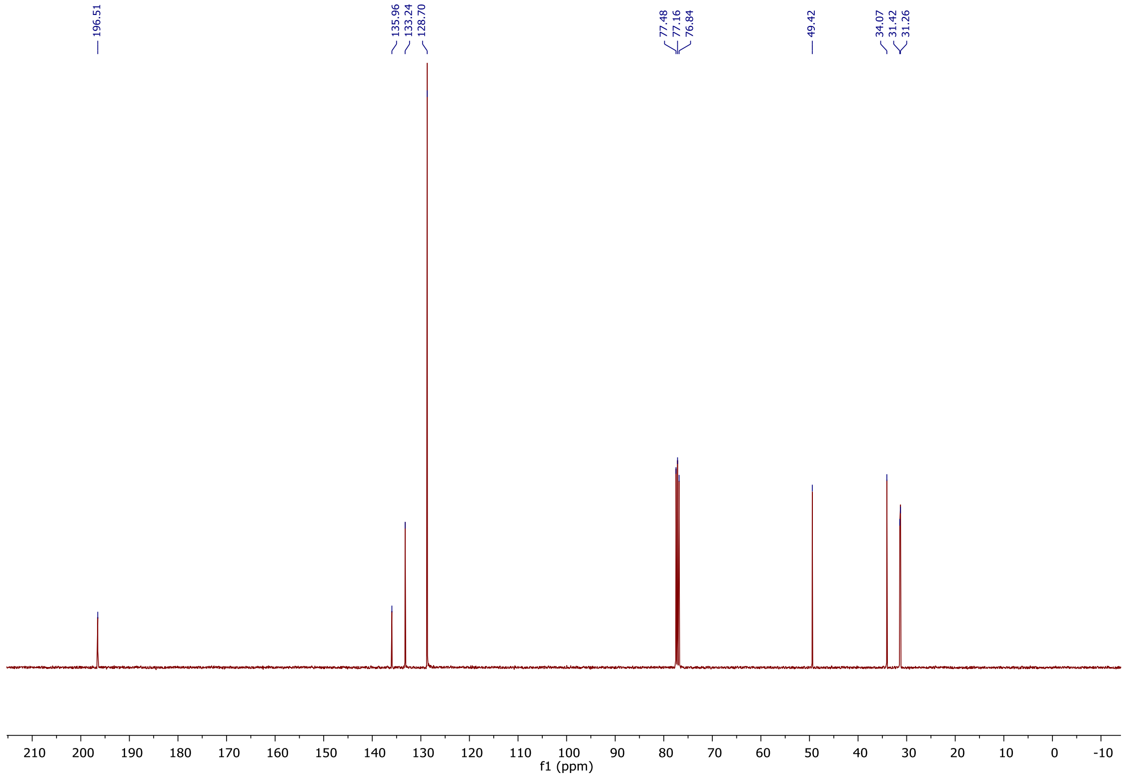

400 MHz ^1H NMR spectrum; 100.6 MHz ^{13}C NMR spectrum; $CDCl_3$ of **7g**

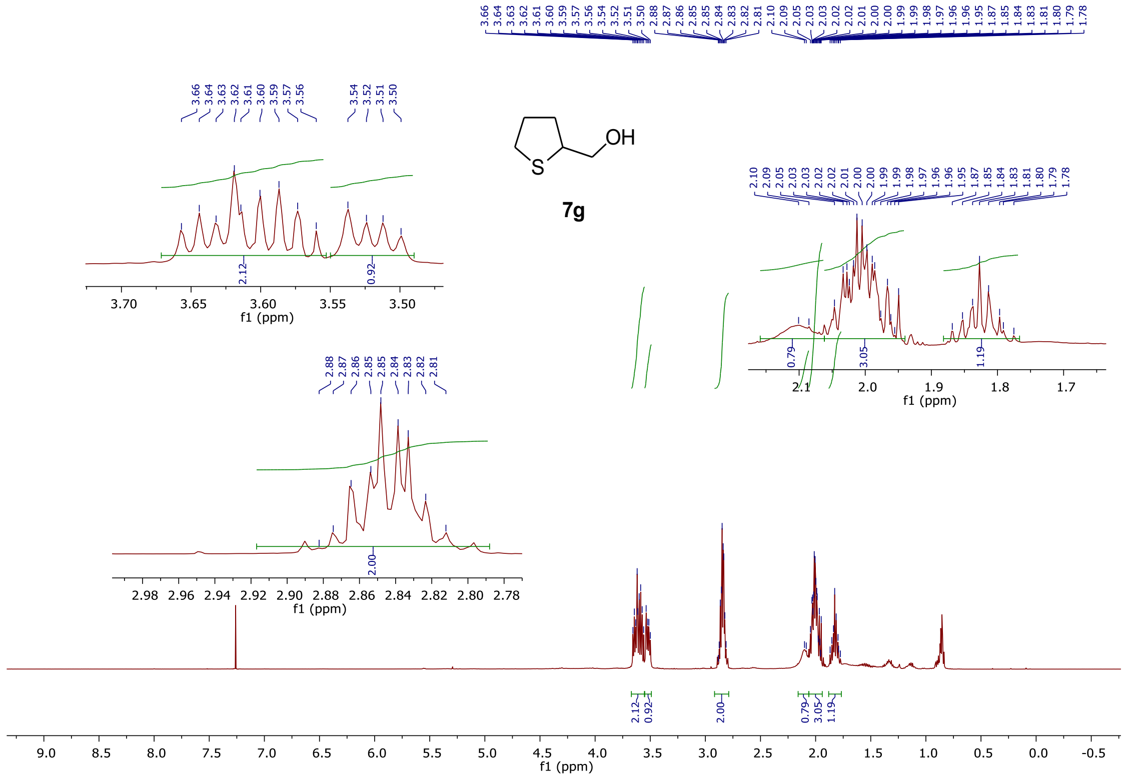

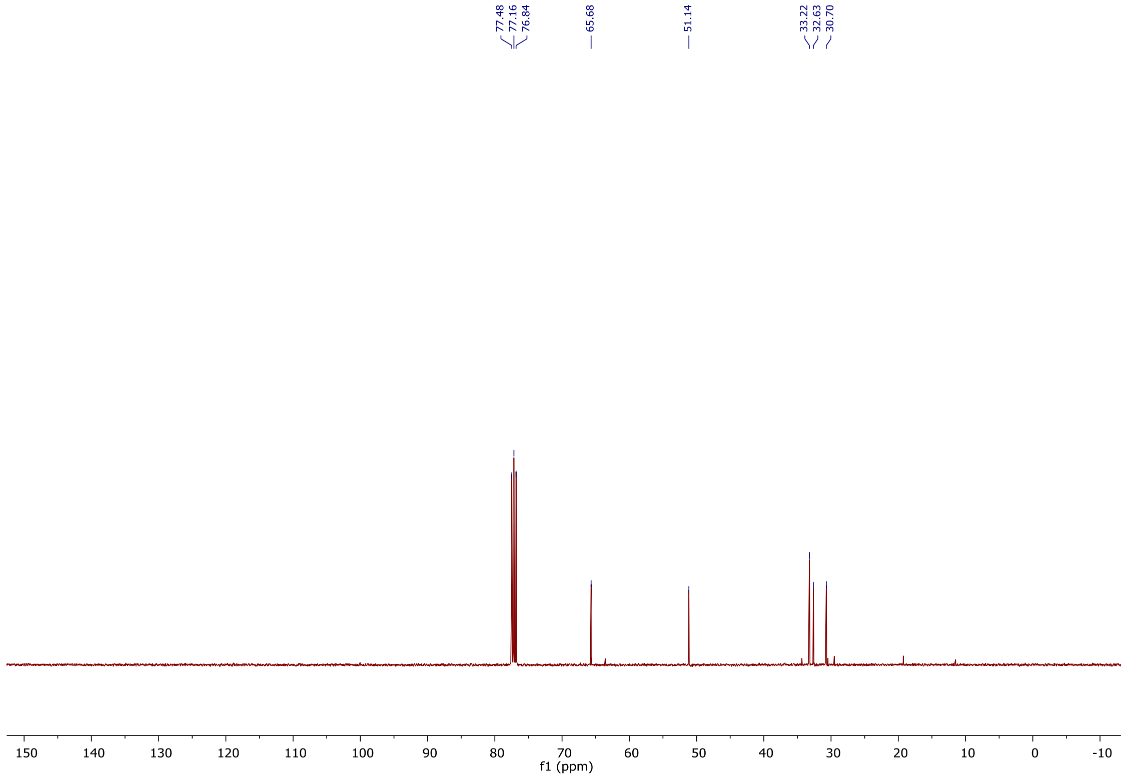

400 MHz ^1H NMR spectrum; 100.6 MHz ^{13}C NMR spectrum; $CDCl_3$ of **7h**

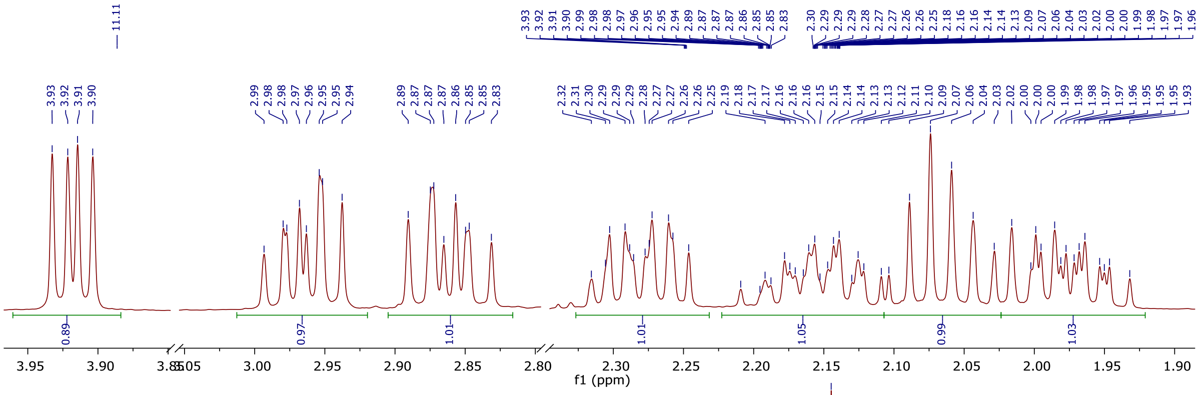

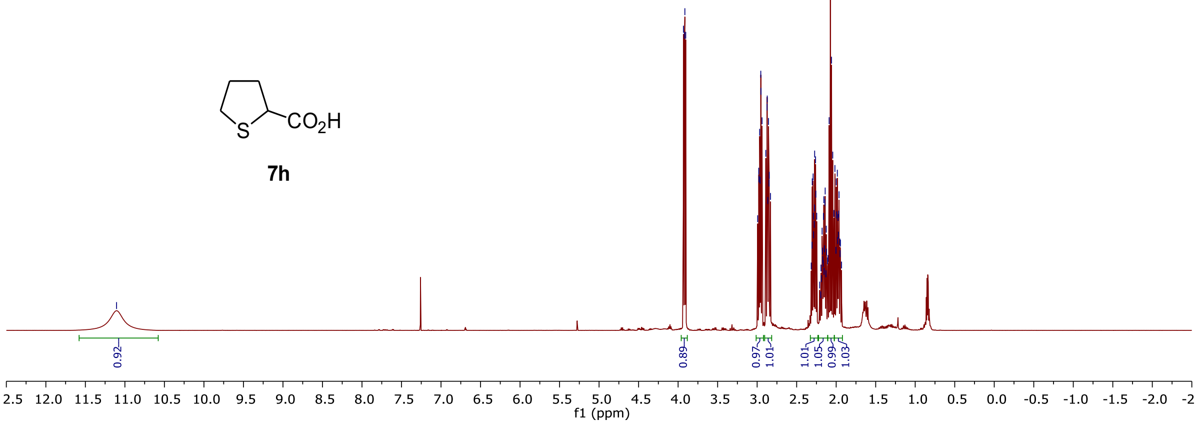

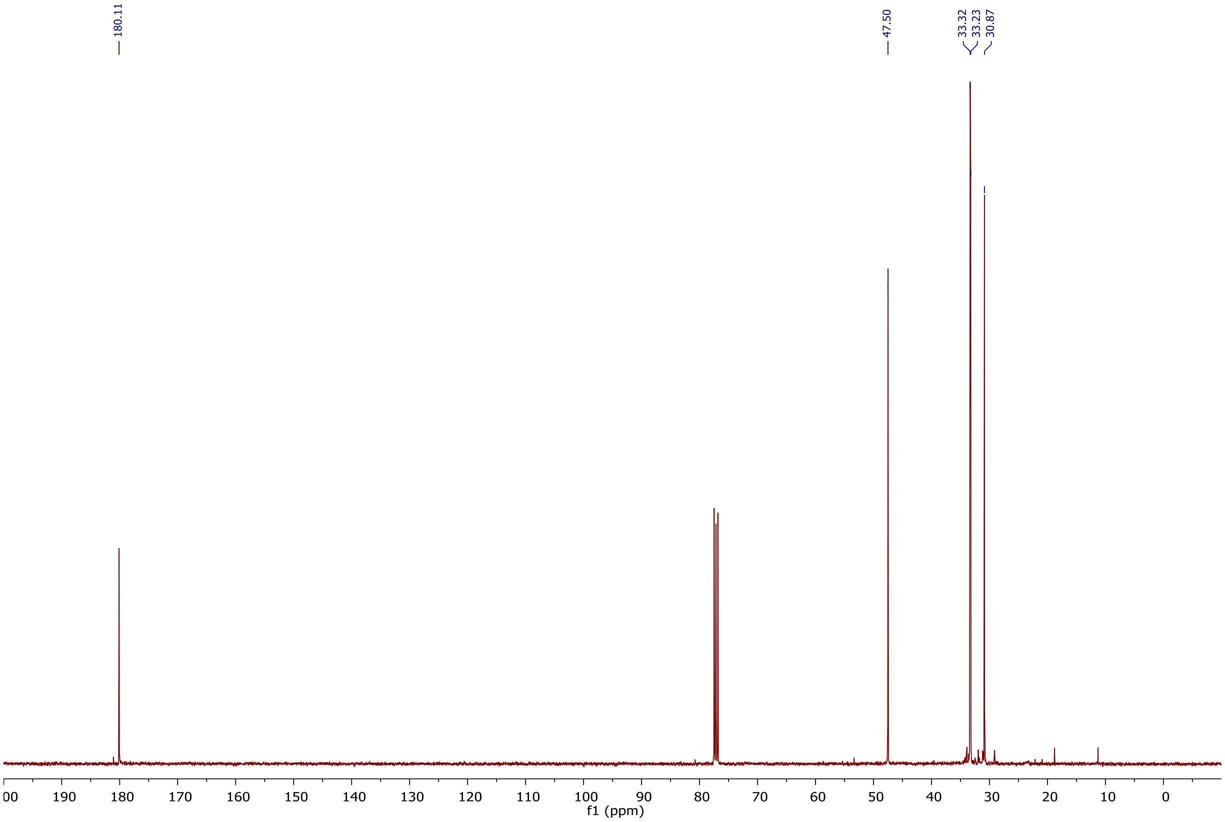

400 MHz ^{1}H NMR spectrum; 100.6 MHz ^{13}C NMR spectrum; $CDCl_3$ of (*S*)-**S1**

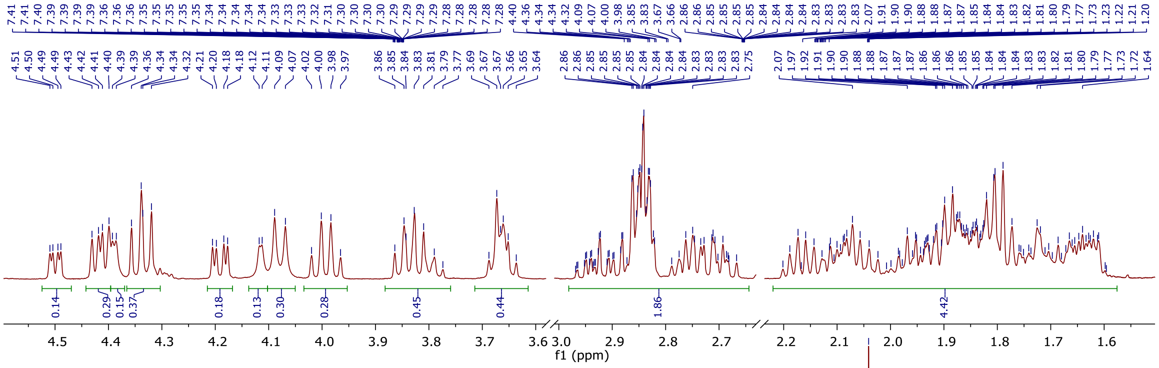

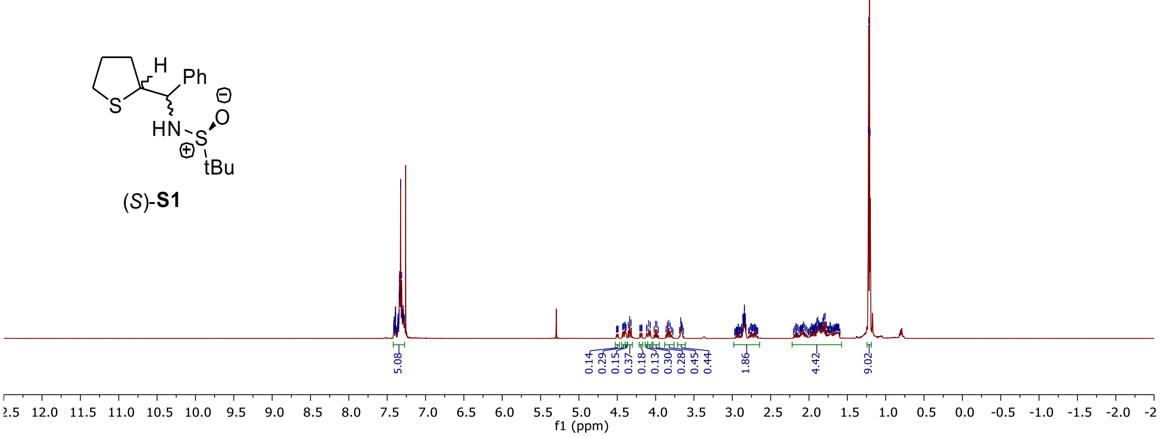

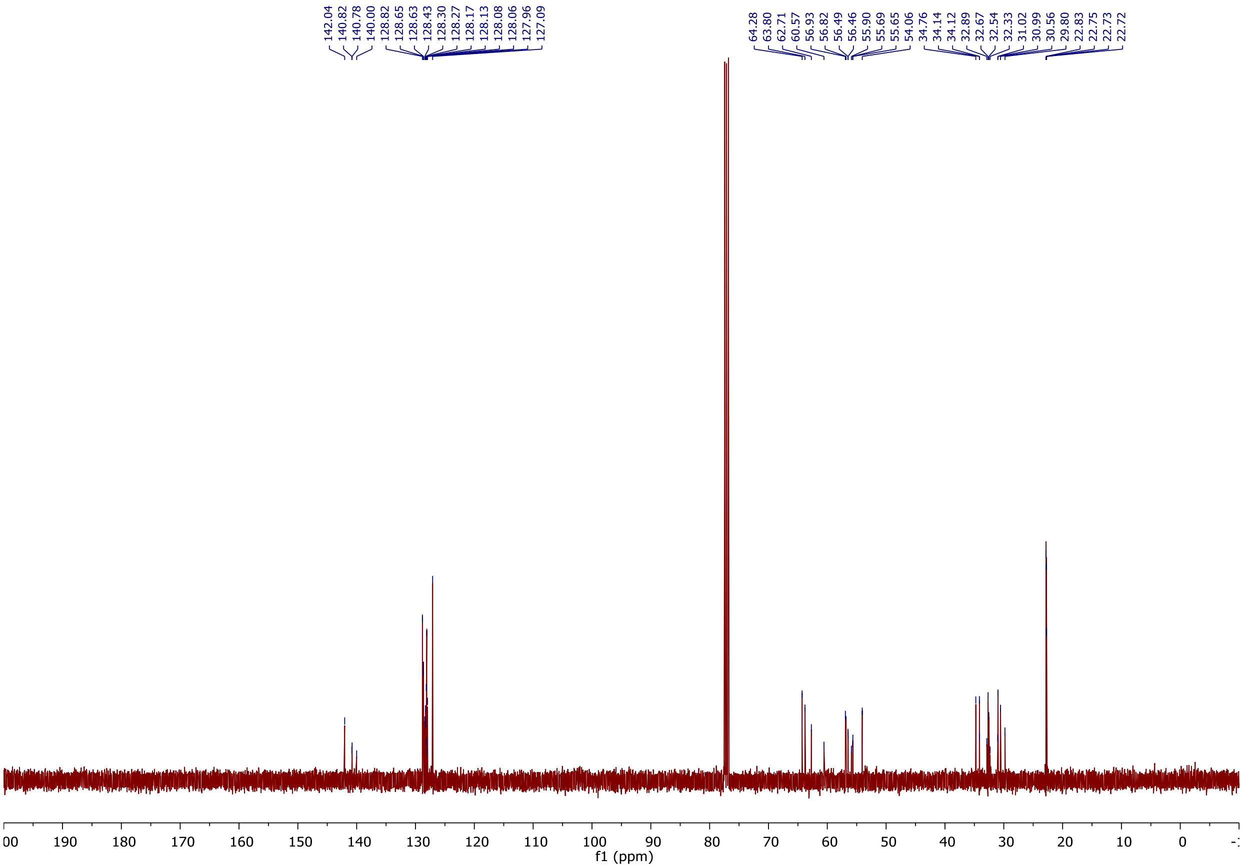

400 MHz ^{1}H NMR spectrum; 100.6 MHz ^{13}C NMR spectrum; $CDCl_3$ of **7i•HCl**

7i

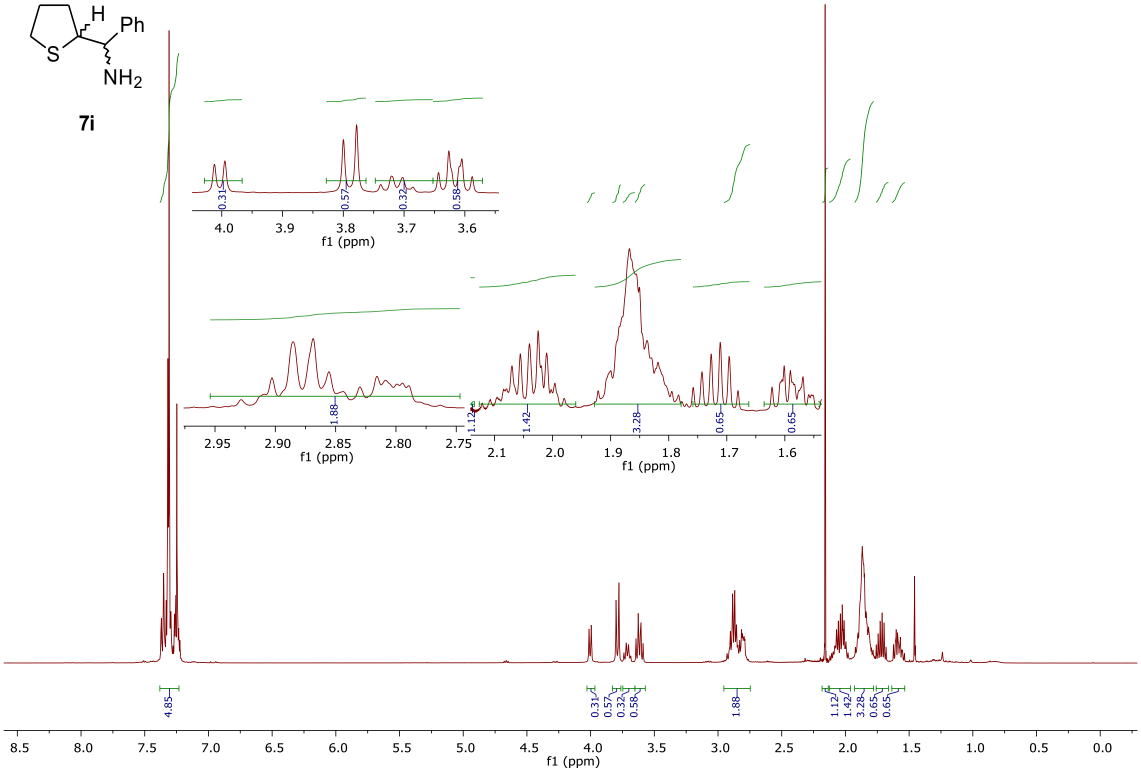

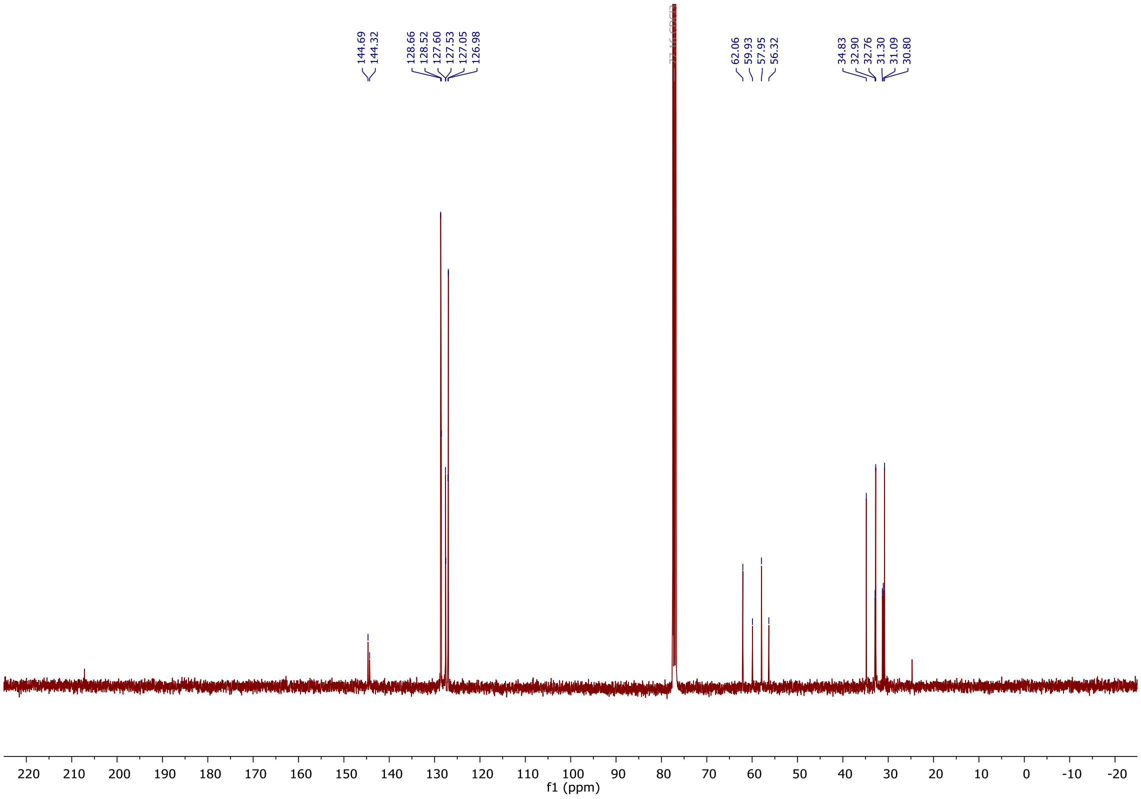

400 MHz ^{1}H NMR spectrum; 100.6 MHz ^{13}C NMR spectrum; $CDCl_3$ of **7j**

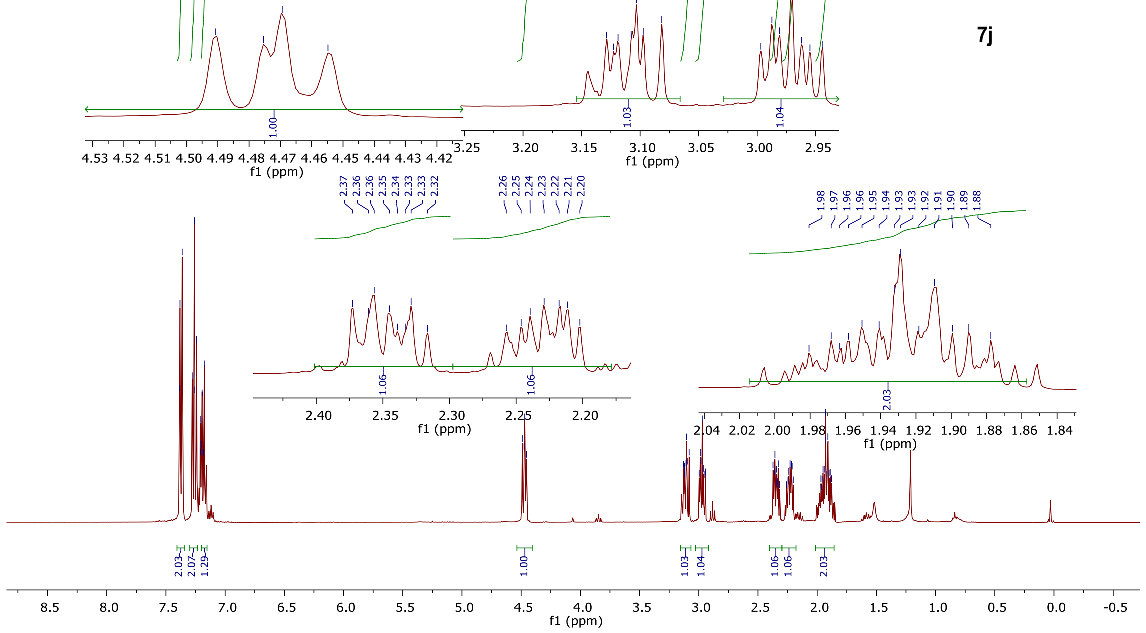

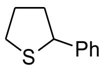

7j

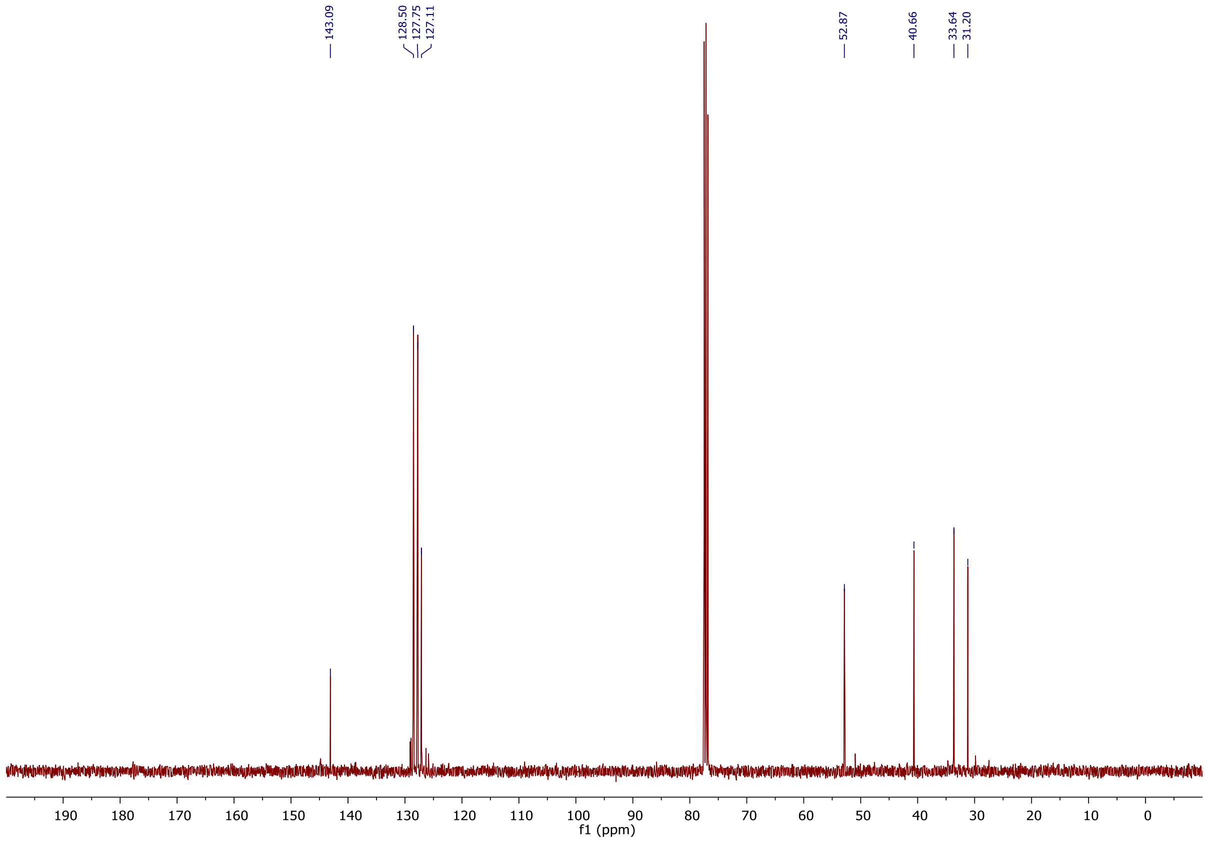

400 MHz ^{1}H NMR spectrum; 100.6 MHz ^{13}C NMR spectrum; $CDCl_3$ of **7k**

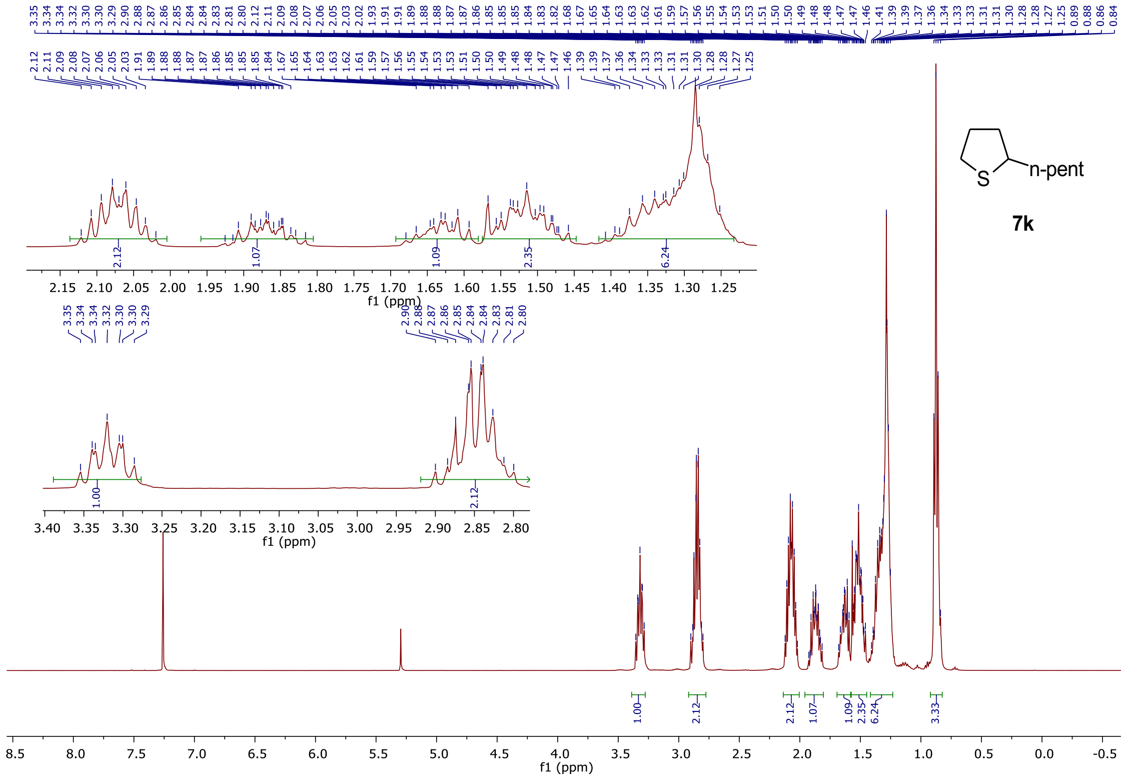

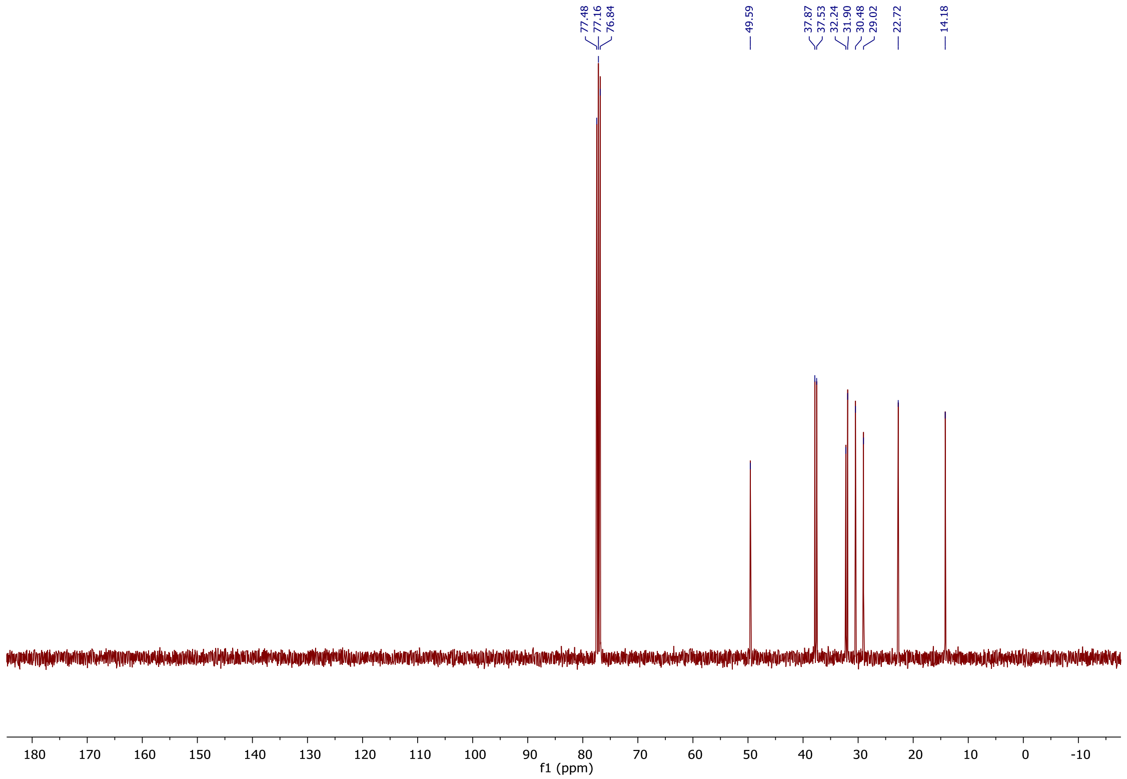

400 MHz ^1H NMR spectrum; 100.6 MHz ^{13}C NMR spectrum; $CDCl_3$ of **7l**

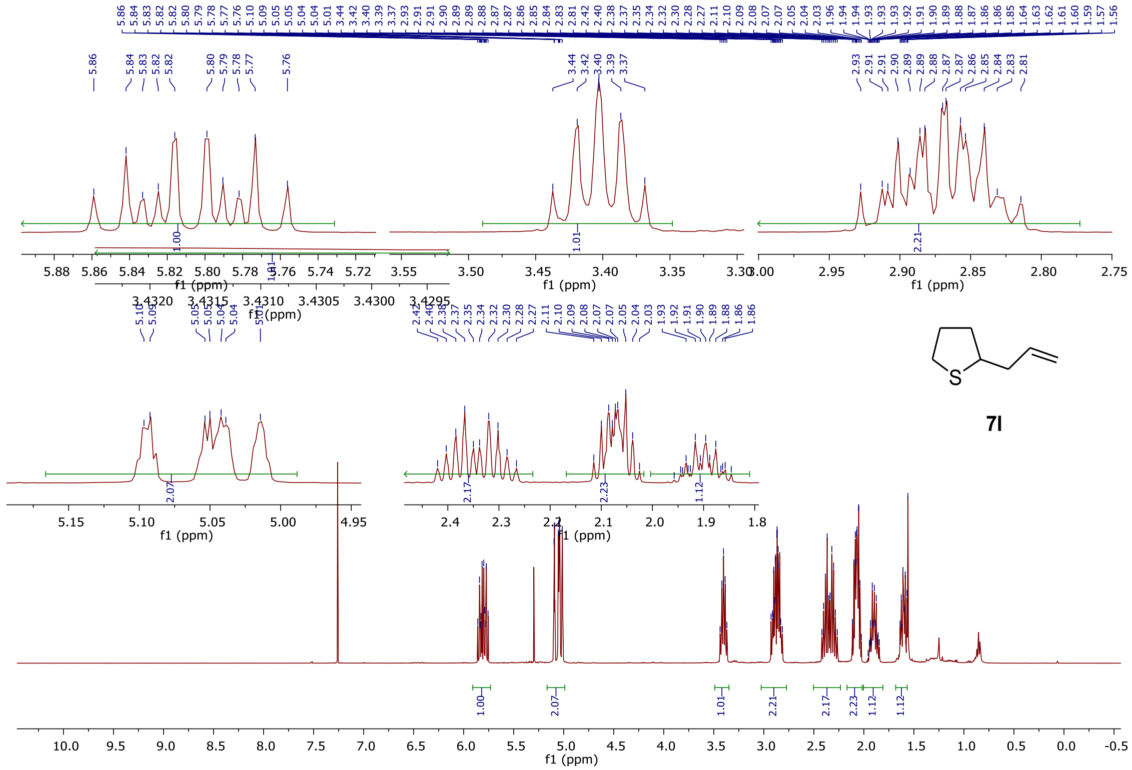

7l

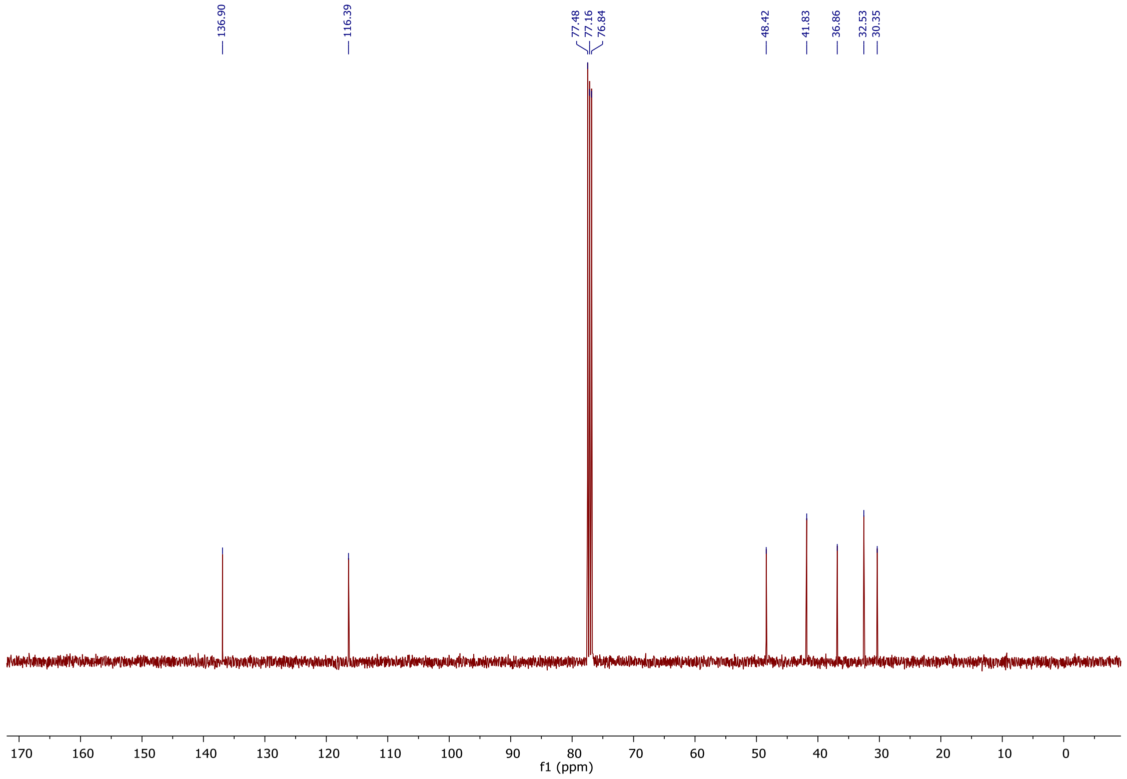

400 MHz ^{1}H NMR spectrum; 100.6 MHz ^{13}C NMR spectrum; $CDCl_3$ of **7m**

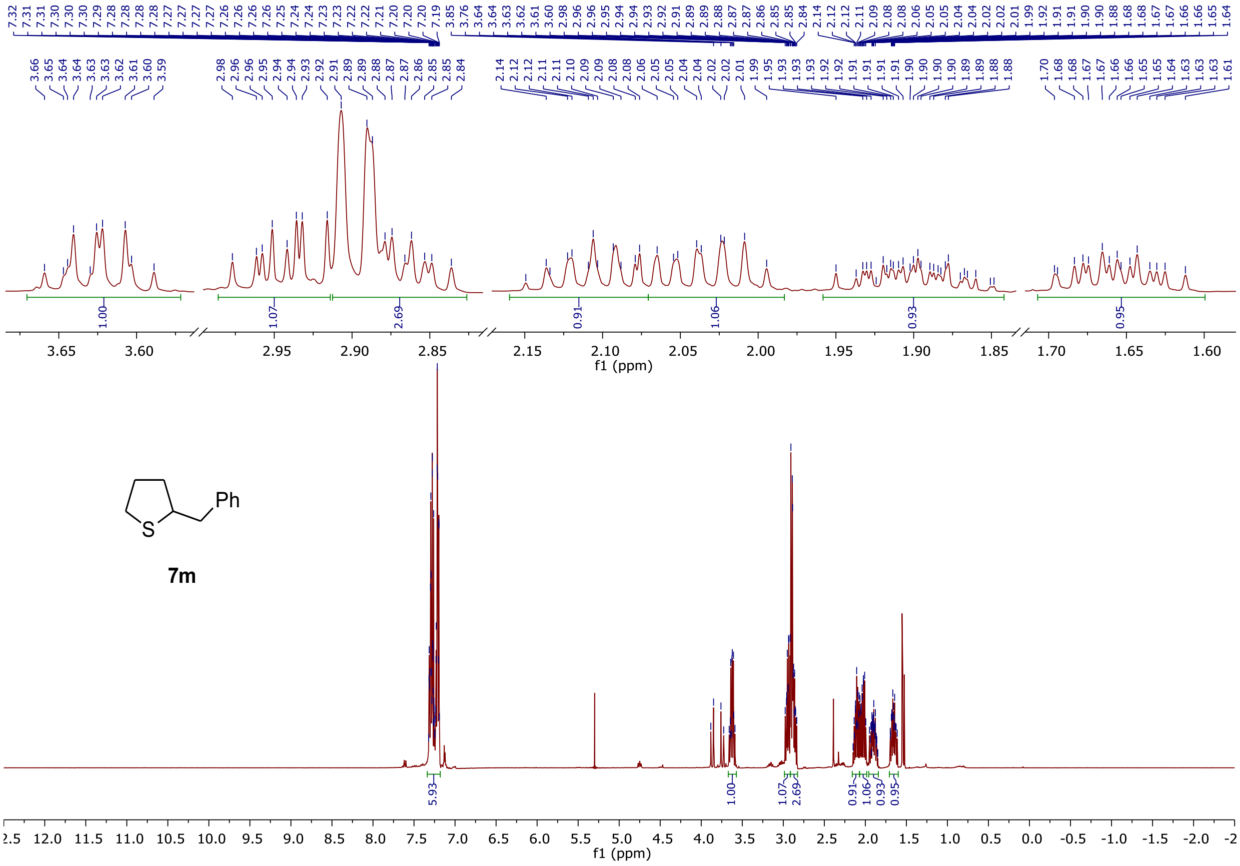

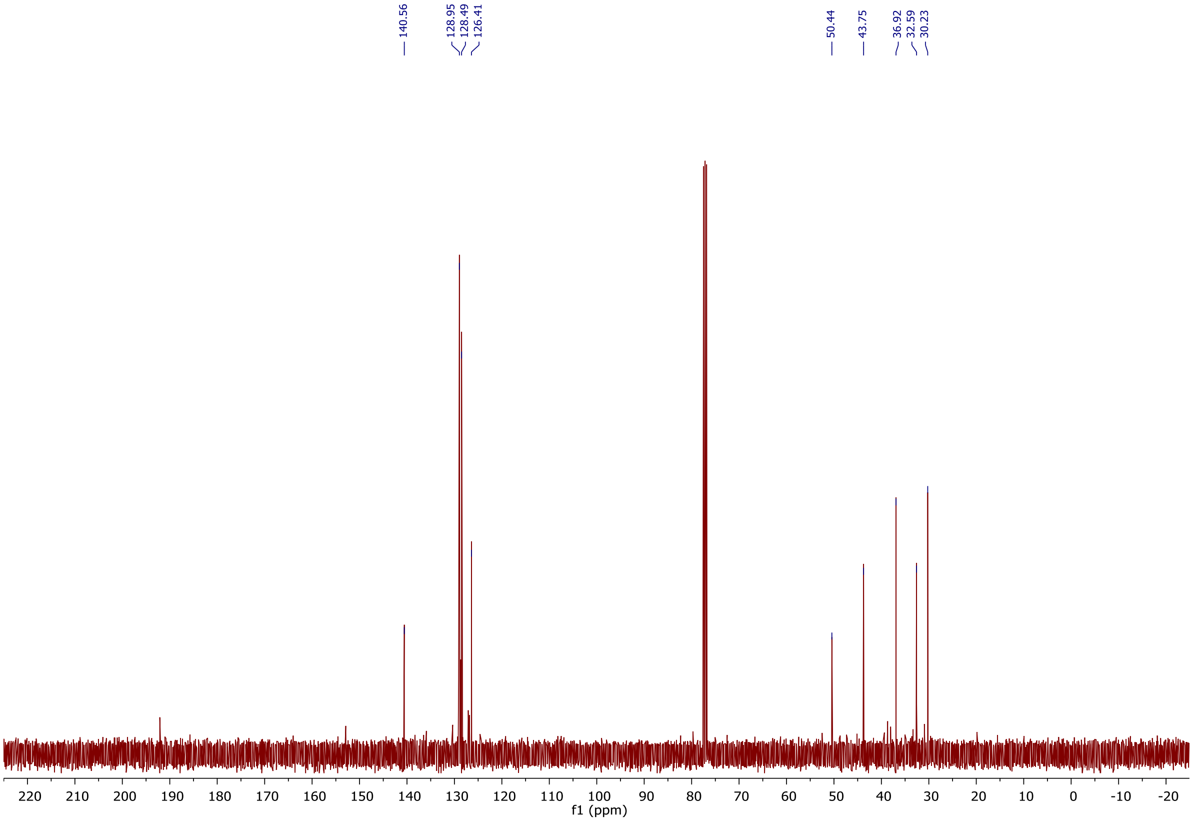

400 MHz ^{1}H NMR spectrum; 100.6 MHz ^{13}C NMR spectrum; $CDCl_3$ of **7n**

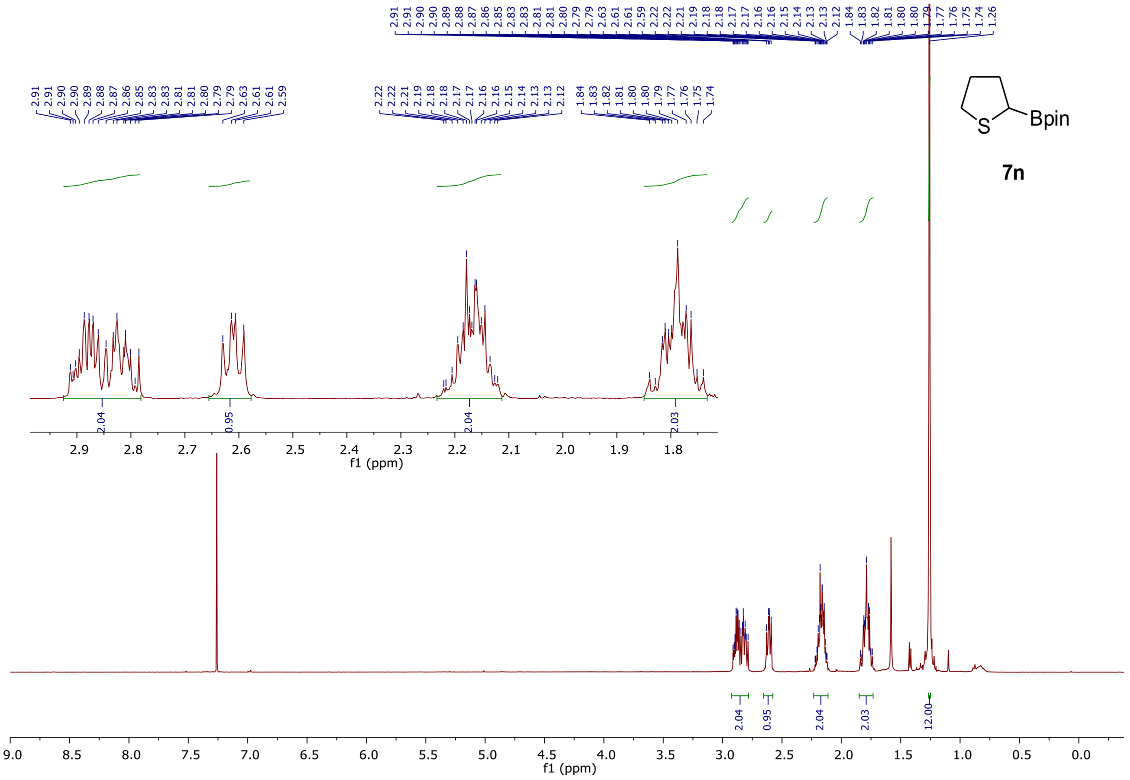

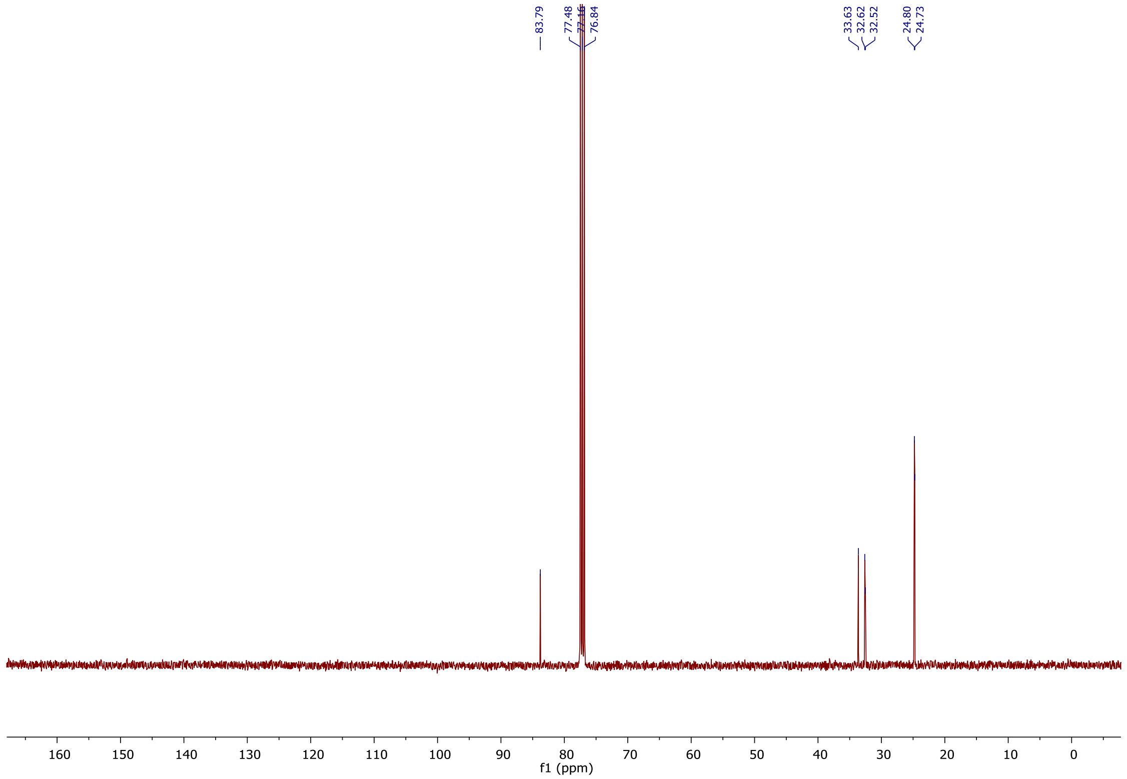

400 MHz ^{1}H NMR spectrum; 100.6 MHz ^{13}C NMR spectrum; $CDCl_3$ of **7o**

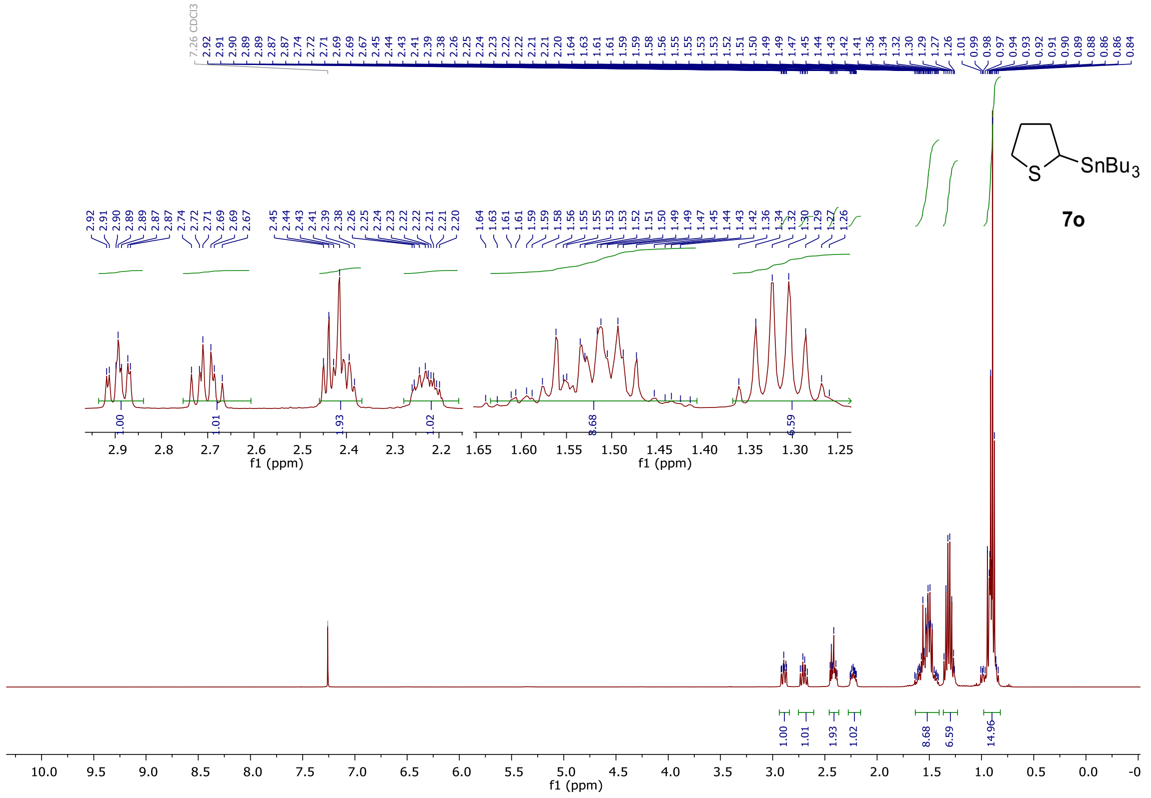

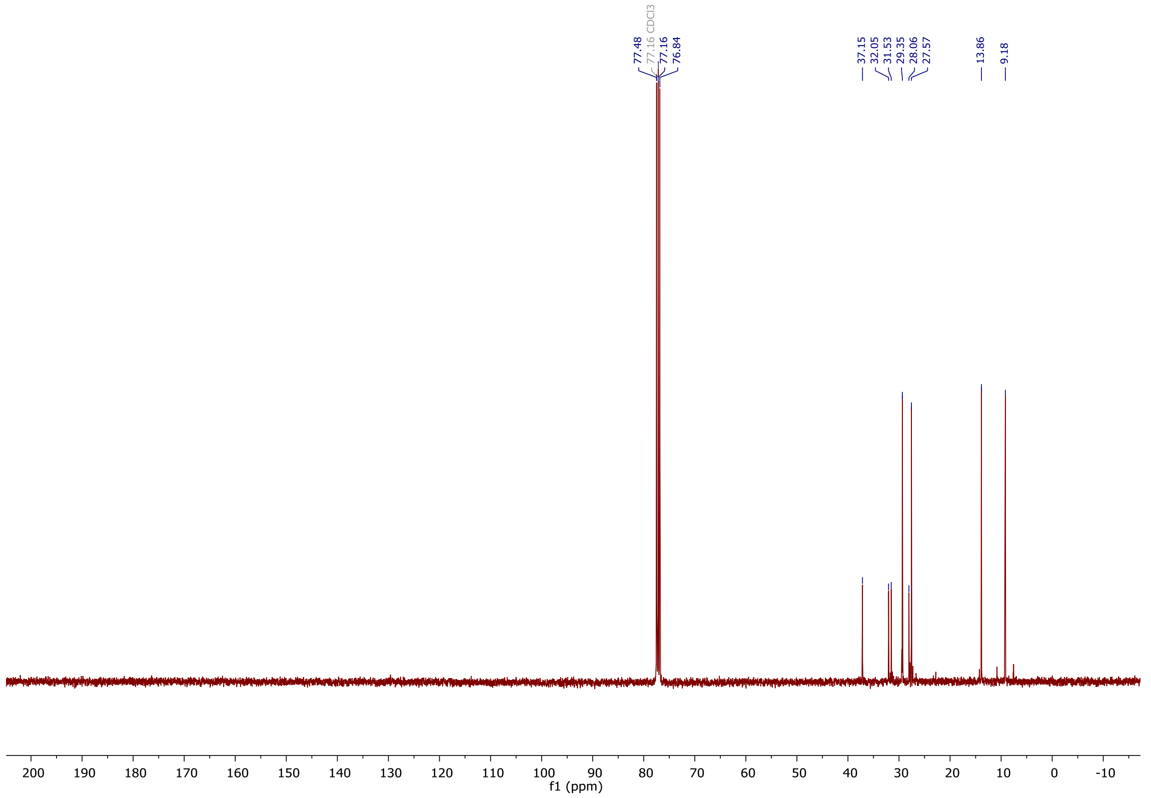

400 MHz ^{1}H NMR spectrum; 100.6 MHz ^{13}C NMR spectrum; $CDCl_3$ of **8a**

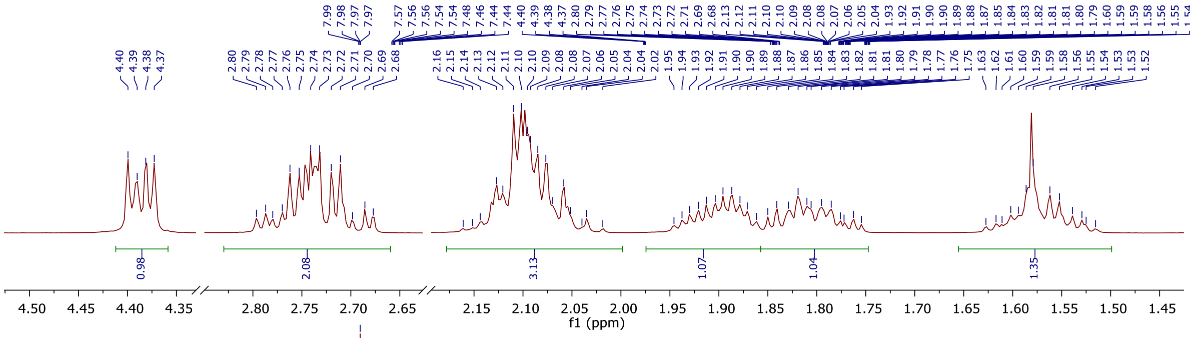

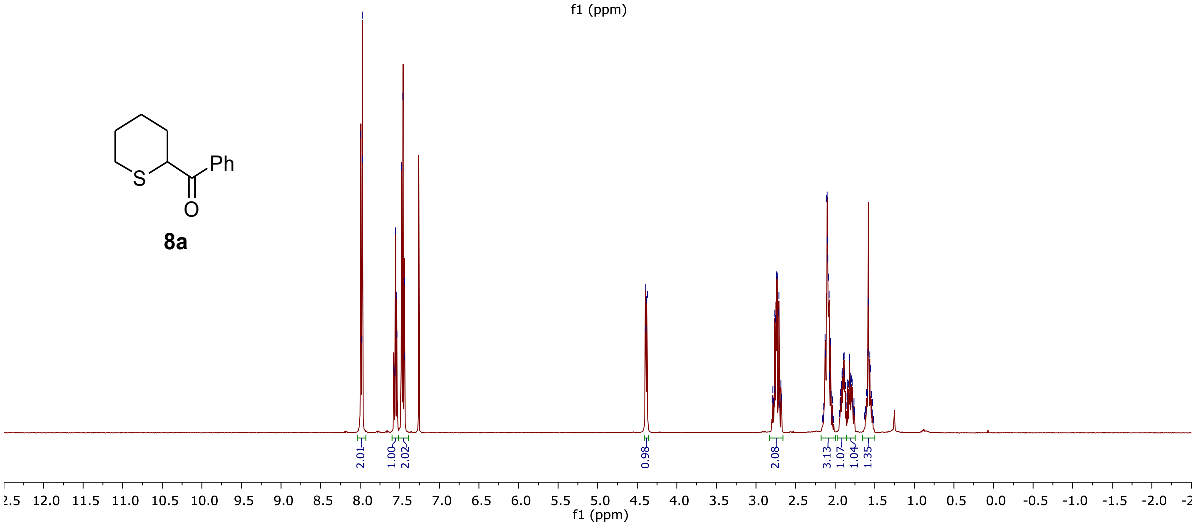

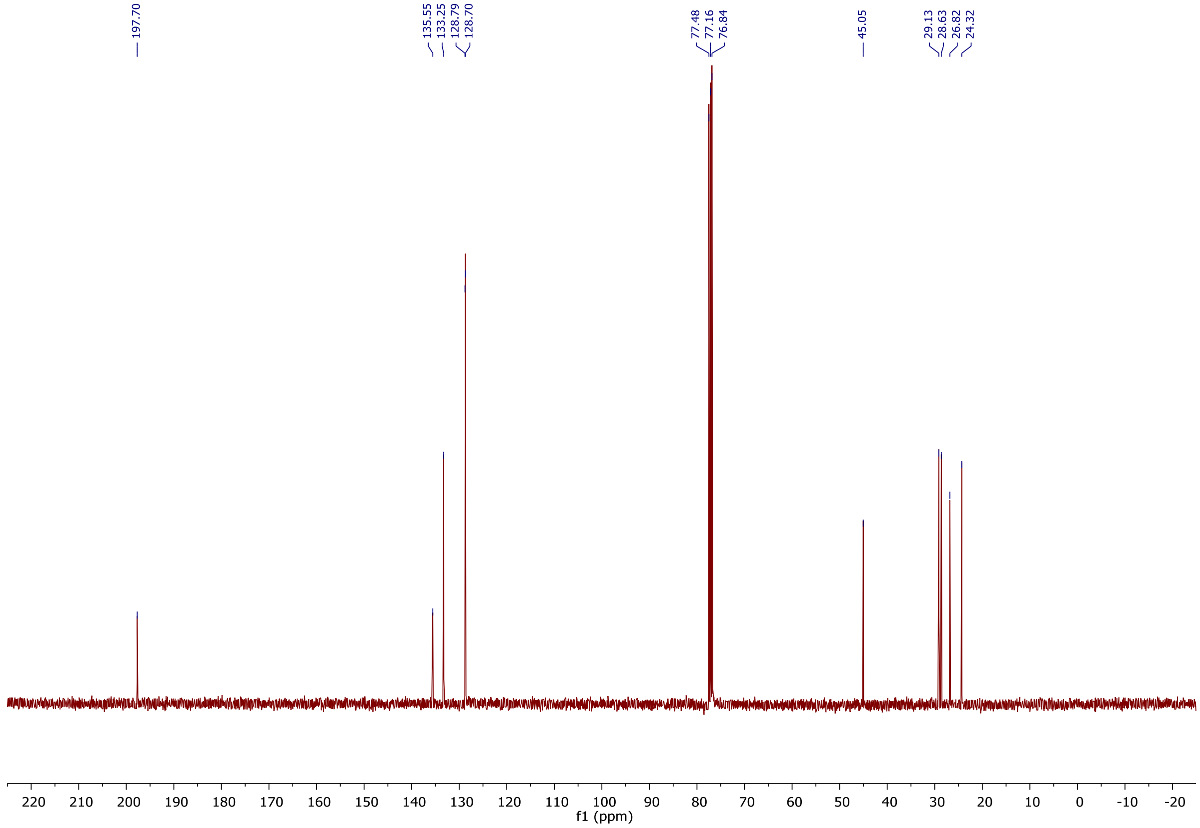

400 MHz ^{1}H NMR spectrum; 100.6 MHz ^{13}C NMR spectrum; $CDCl_3$ of **8b**

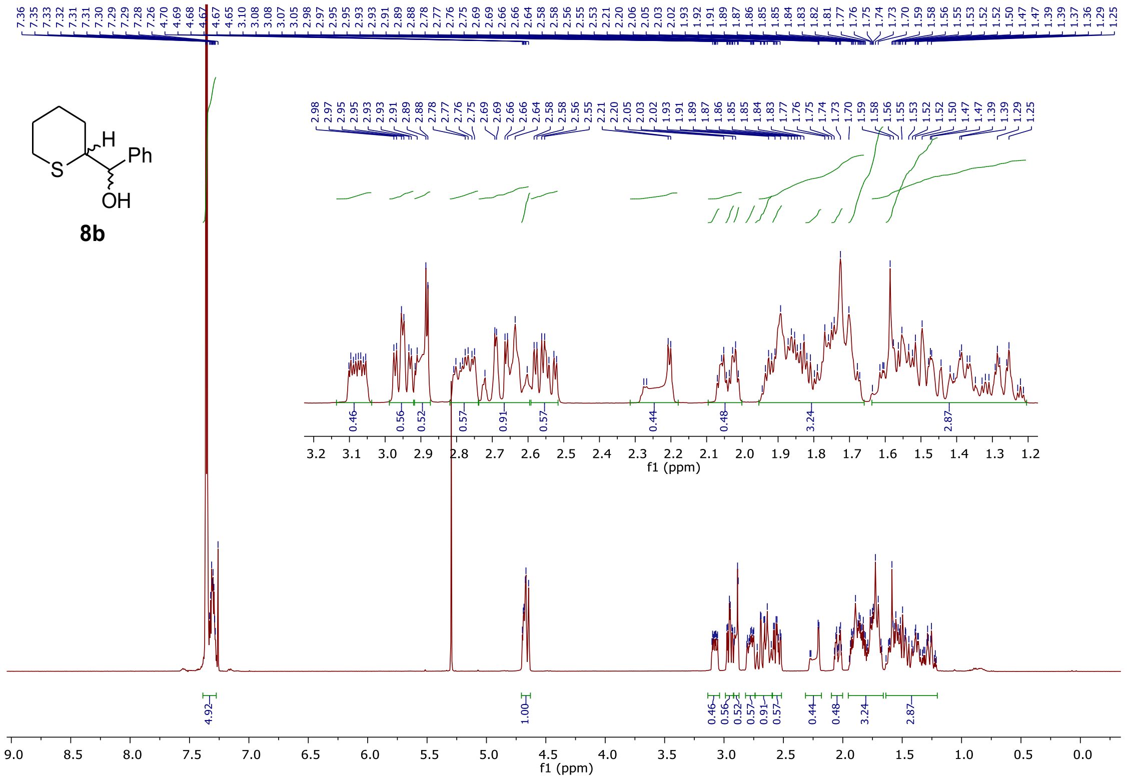

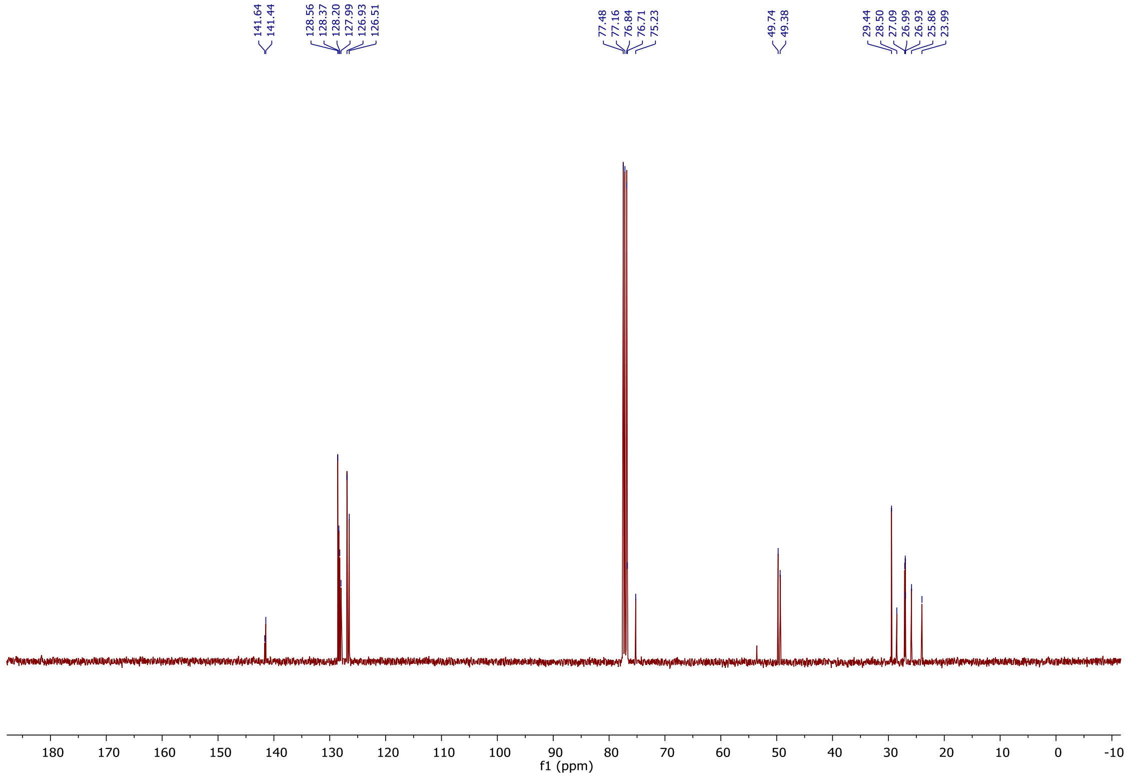

400 MHz ^{1}H NMR spectrum; 100.6 MHz ^{13}C NMR spectrum; $CDCl_3$ of **8c**

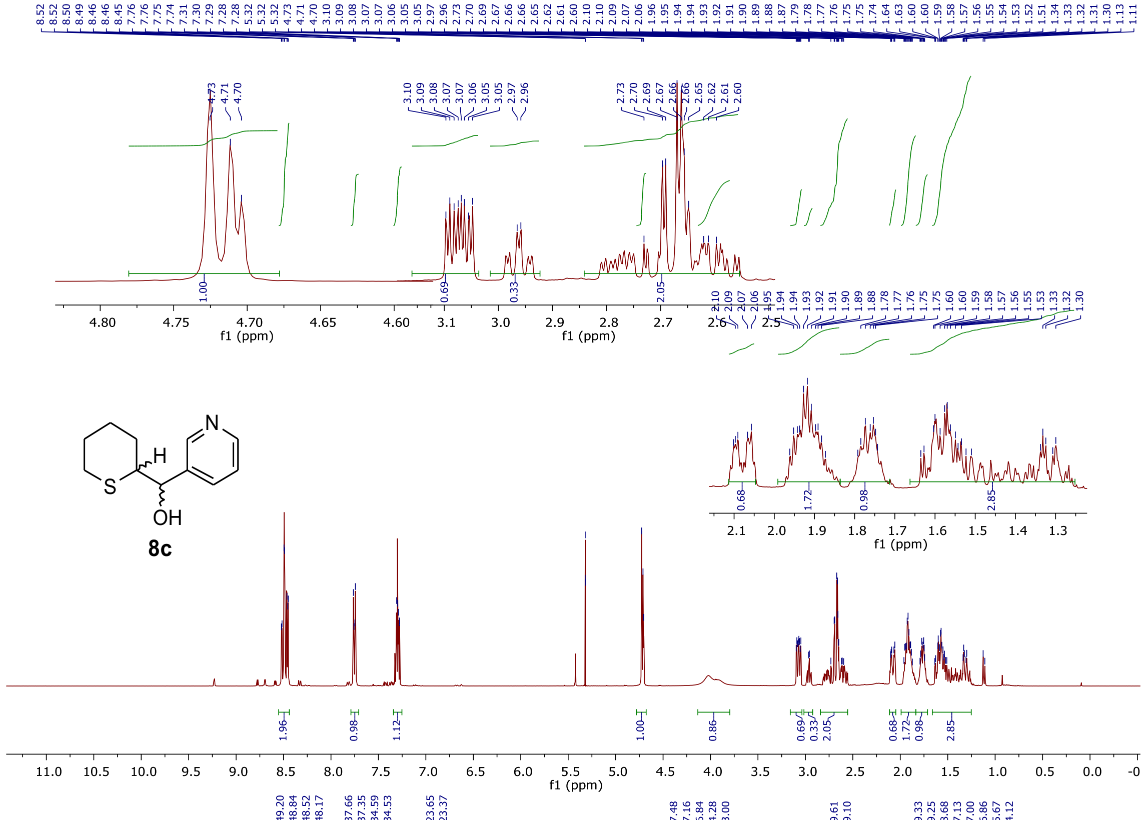

8c

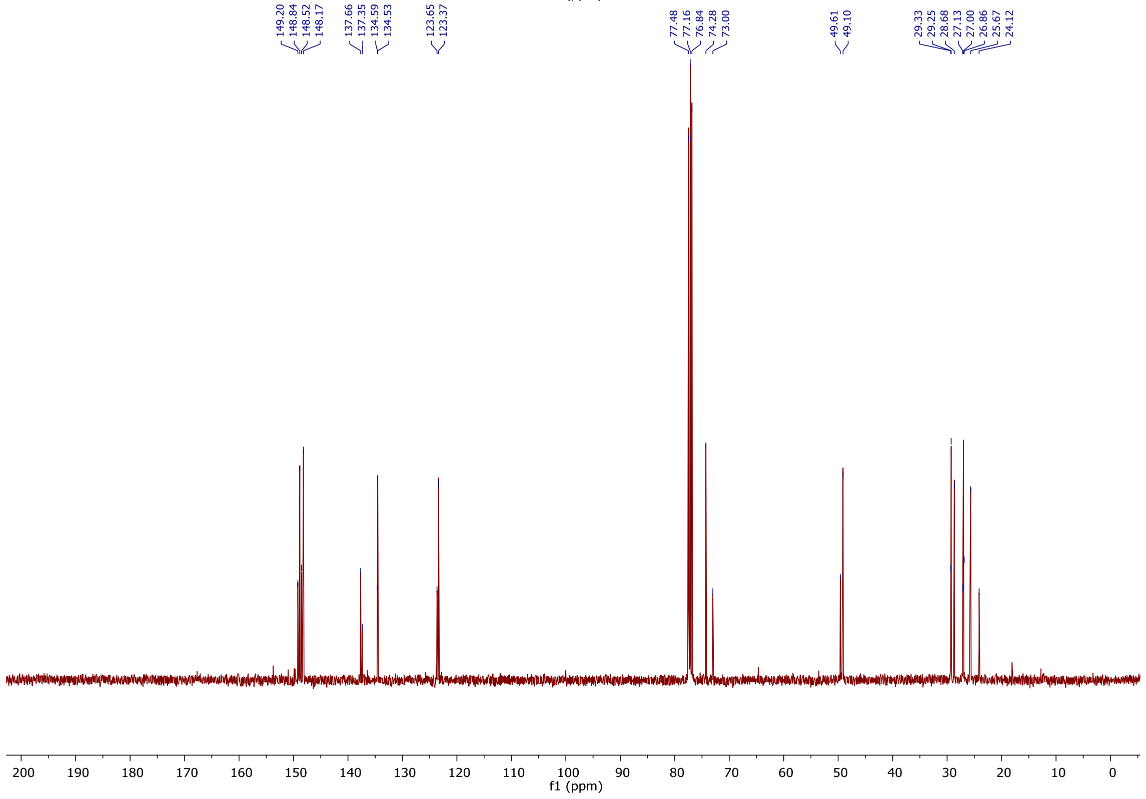

400 MHz ^{1}H NMR spectrum; 100.6 MHz ^{13}C NMR spectrum; $CDCl_3$ of **8d**

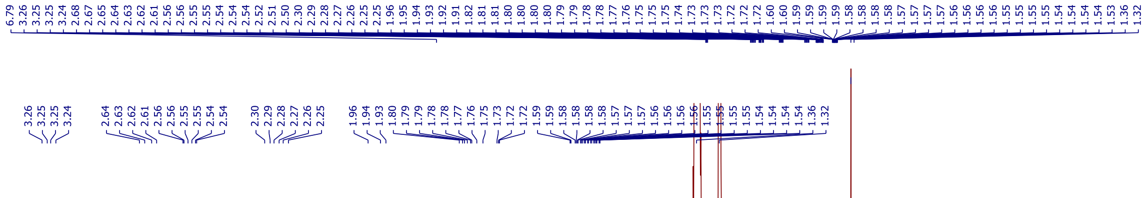

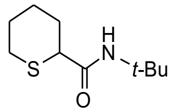

8d

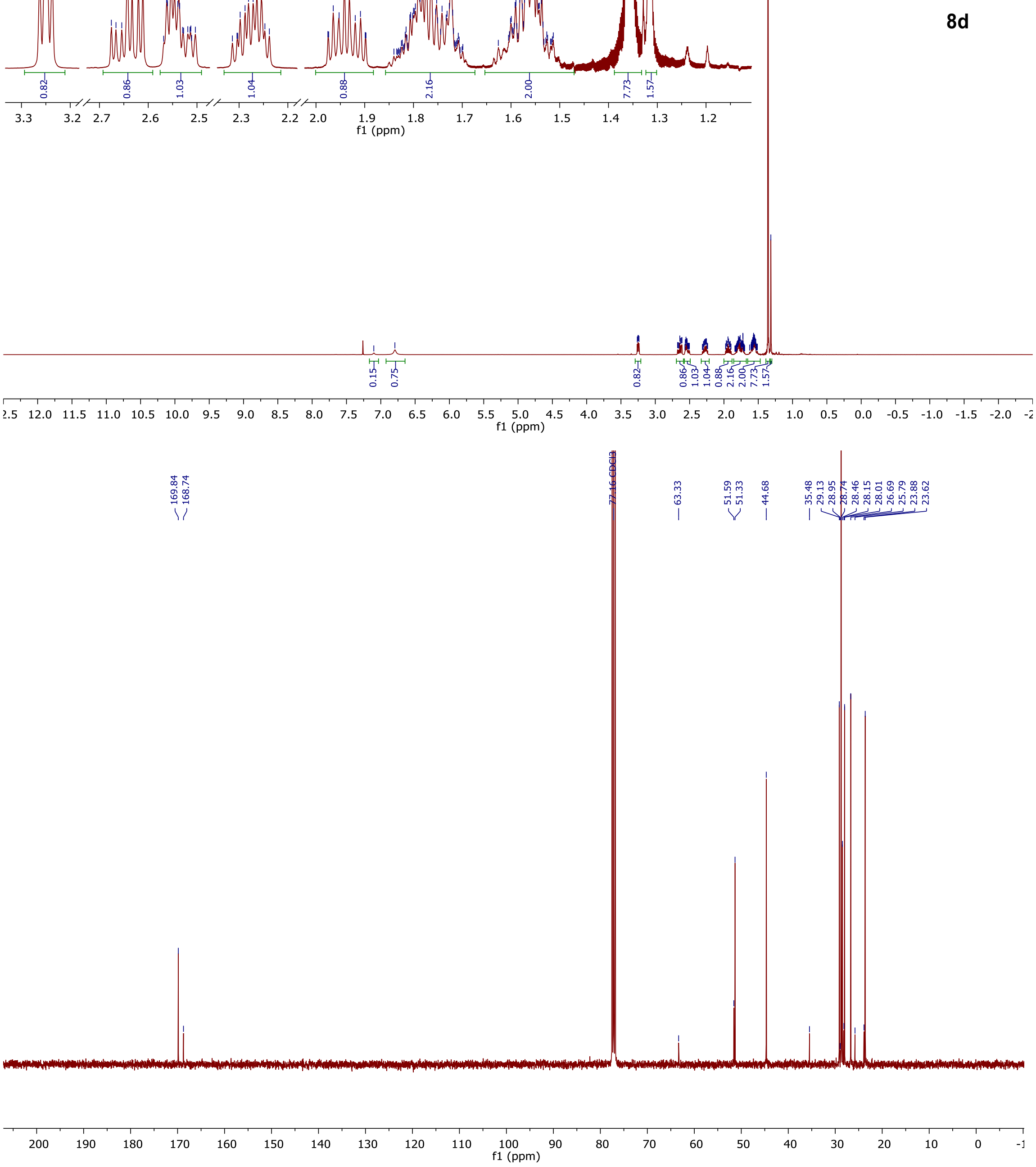

400 MHz ^1H NMR spectrum; 100.6 MHz ^{13}C NMR spectrum; $CDCl_3$ of **8e**

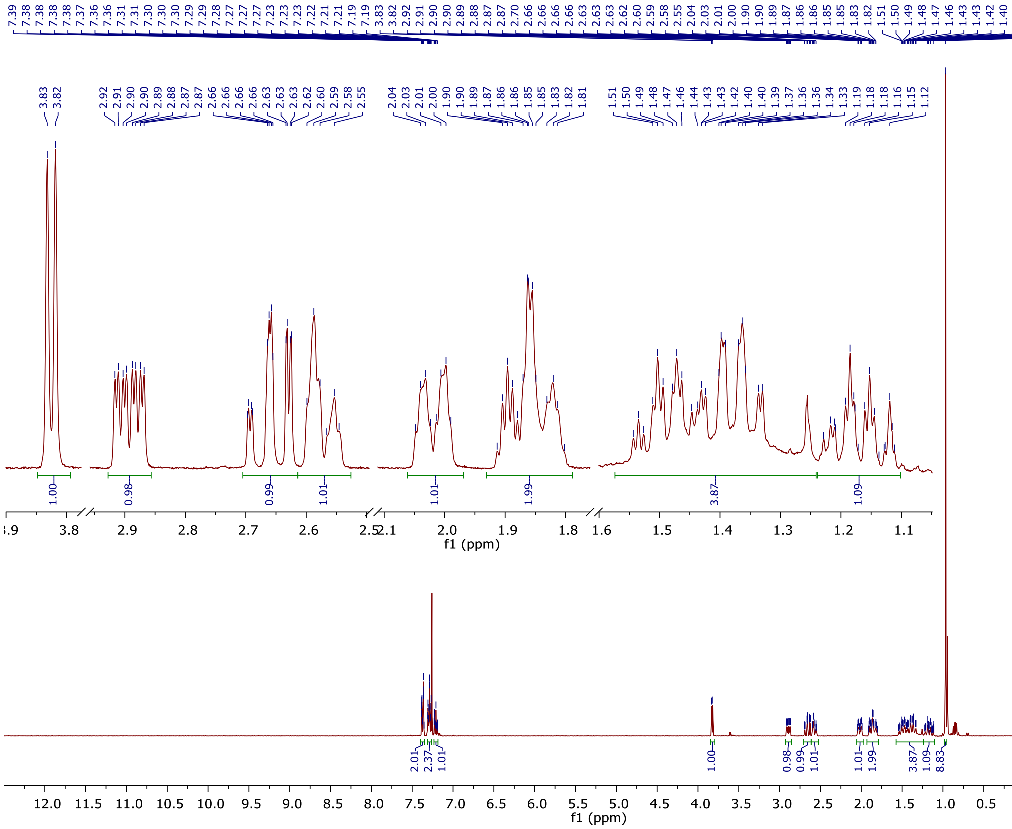

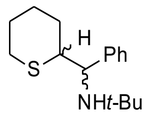

8e

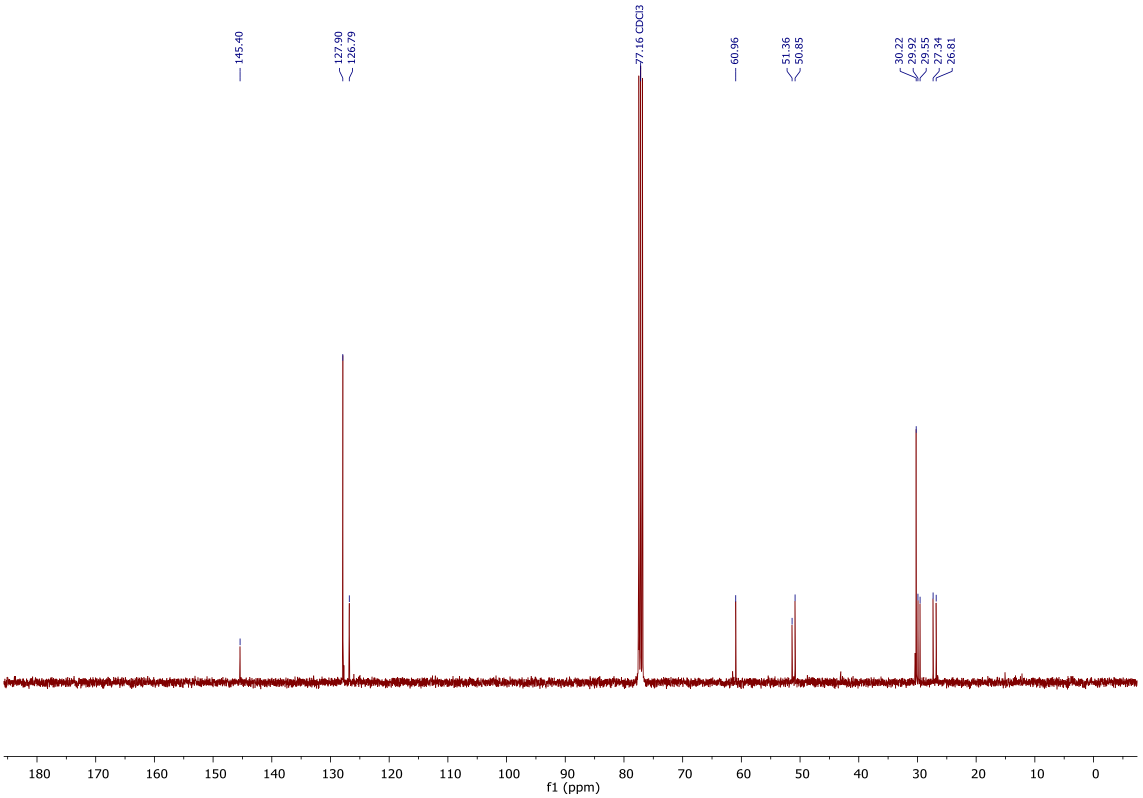

400 MHz ^{1}H NMR spectrum; 100.6 MHz ^{13}C NMR spectrum; $CDCl_3$ of **8f**

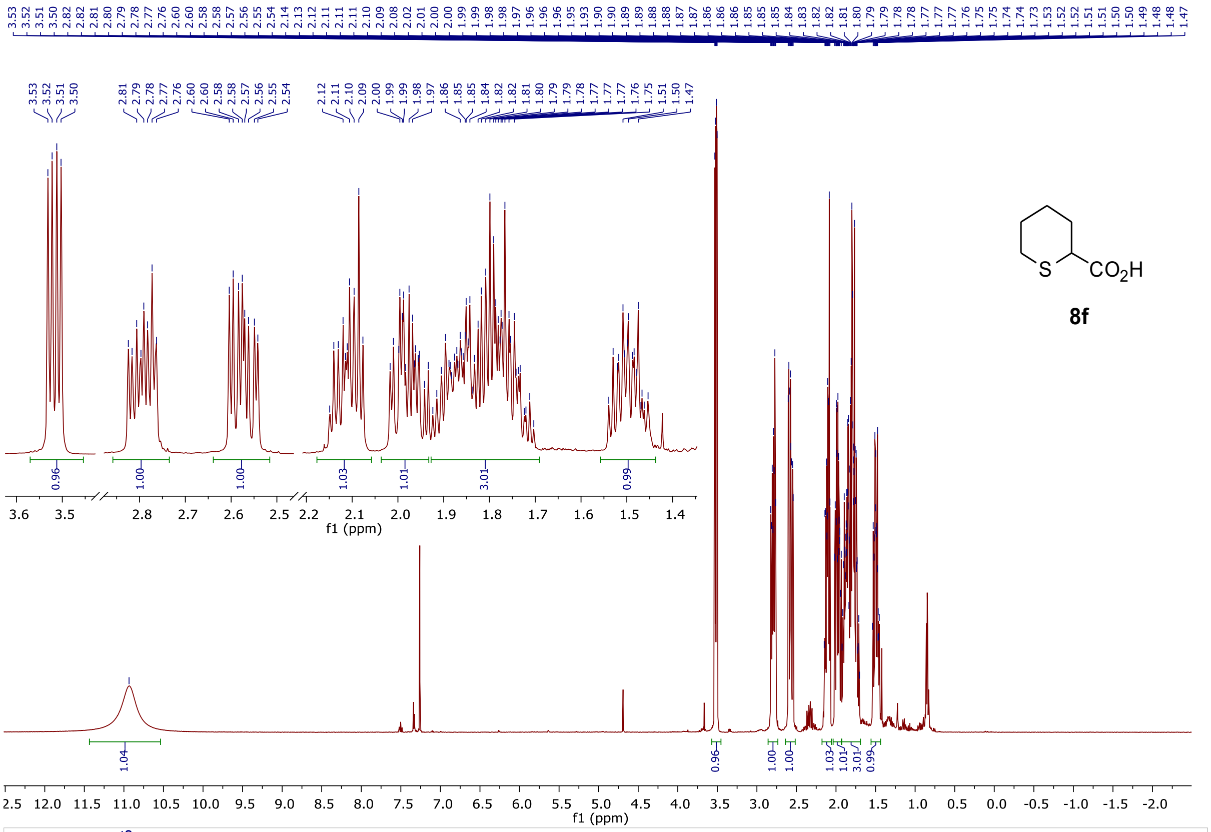

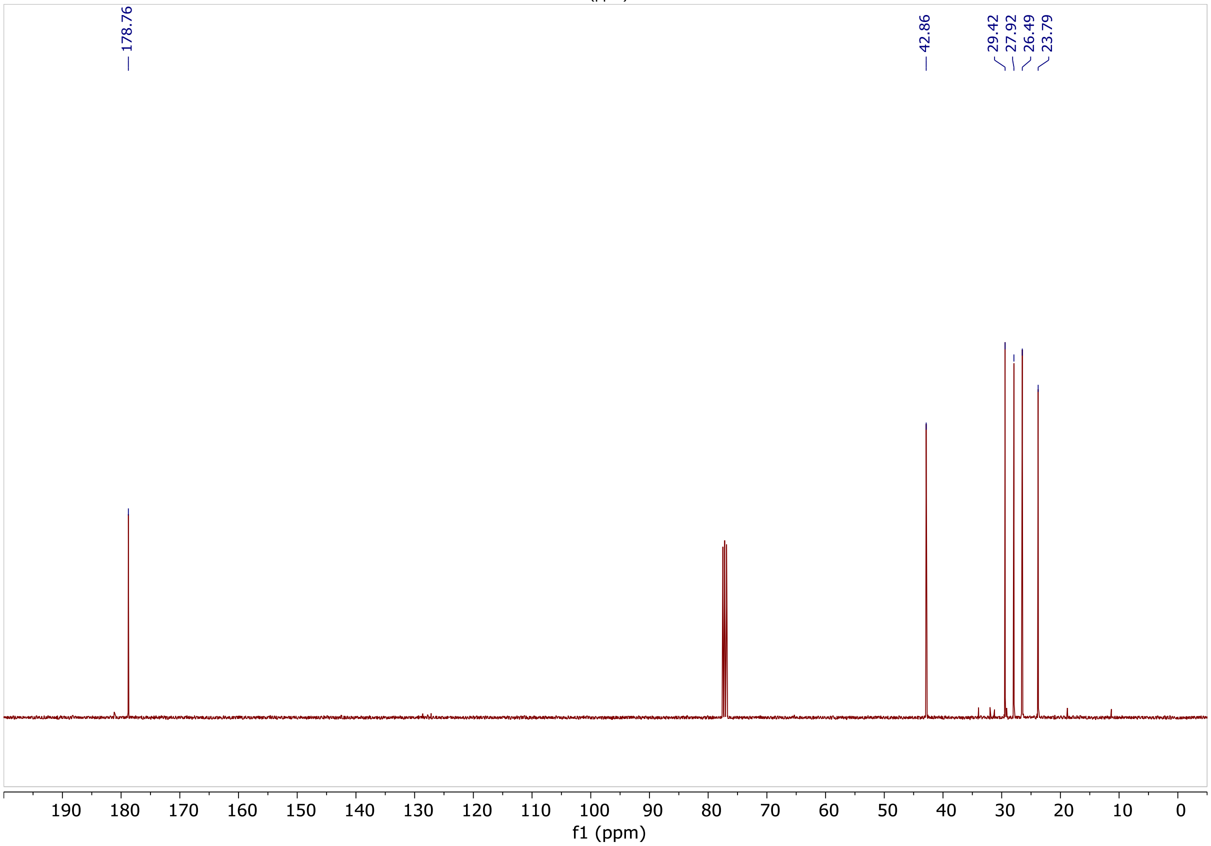

400 MHz ^{1}H NMR spectrum; 100.6 MHz ^{13}C NMR spectrum; $CDCl_3$ of **8g**

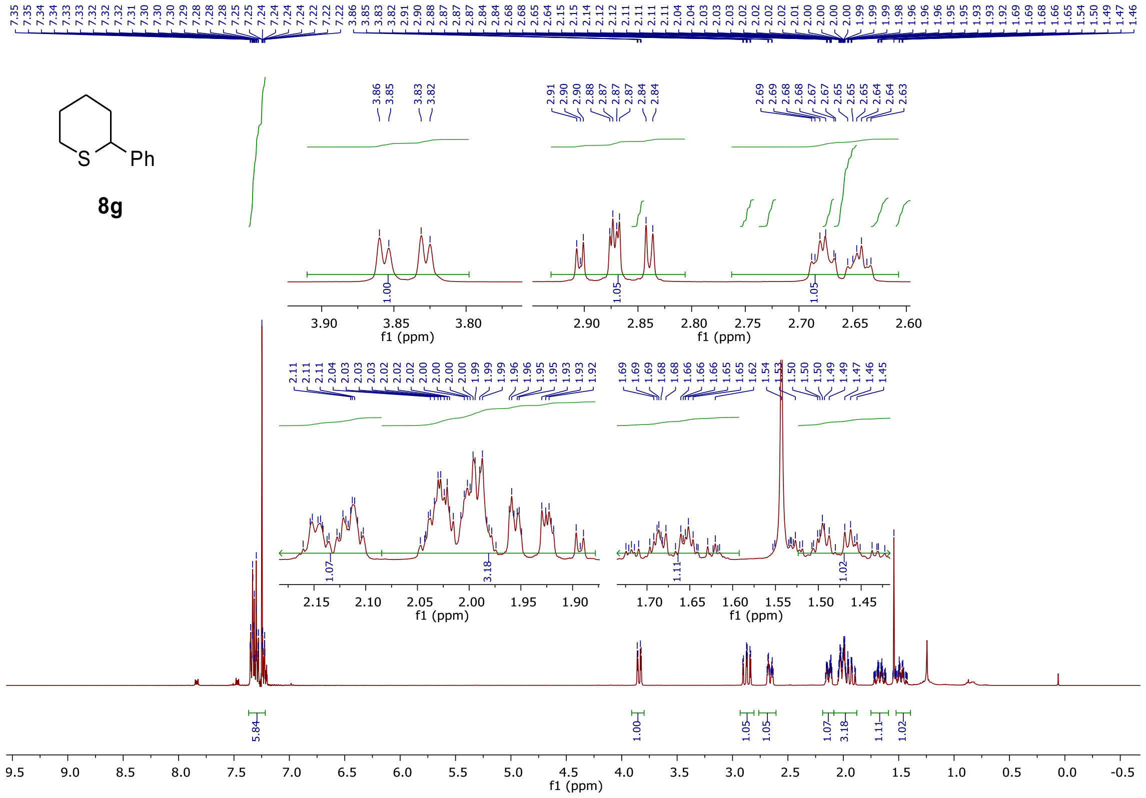

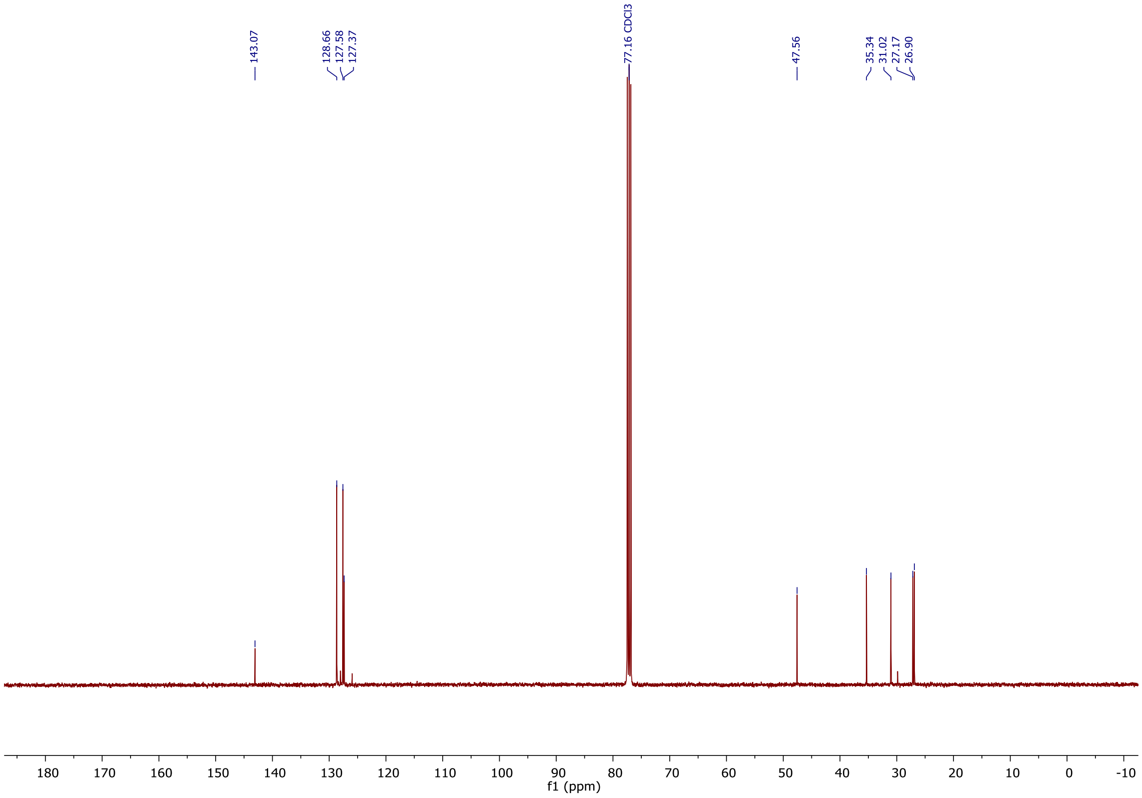

400 MHz ^{1}H NMR spectrum; 100.6 MHz ^{13}C NMR spectrum; $CDCl_3$ of **8h**

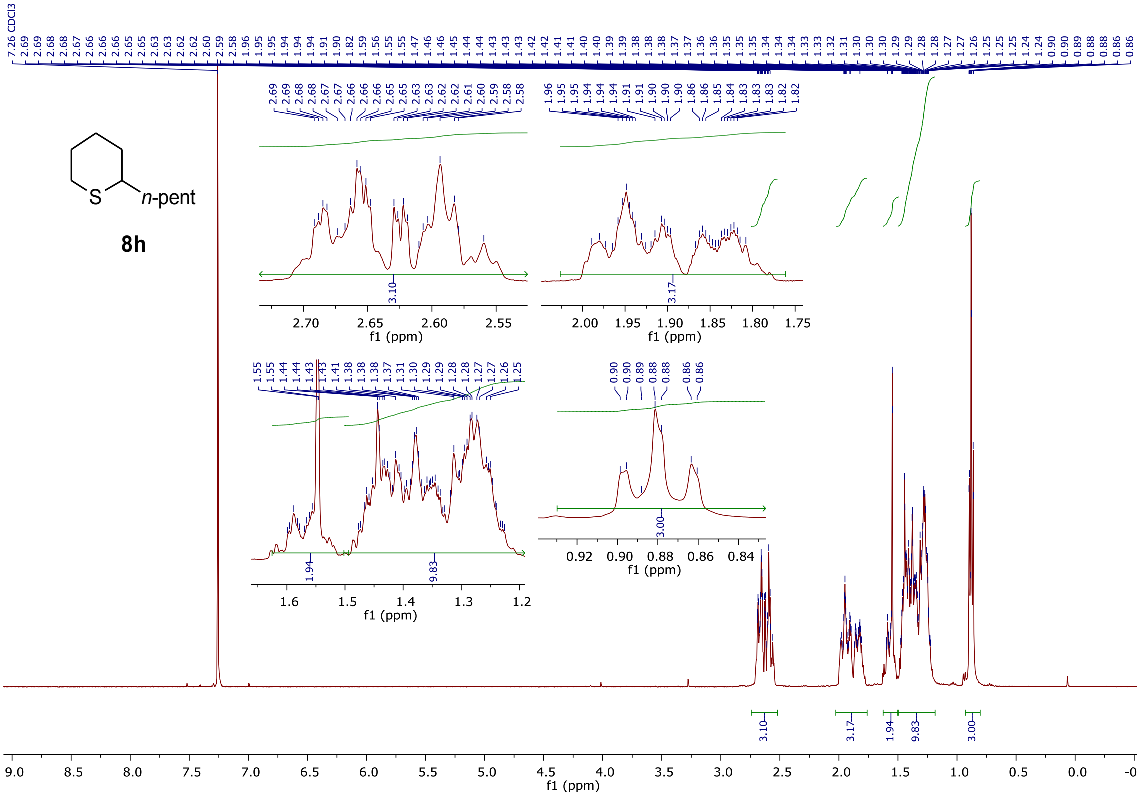

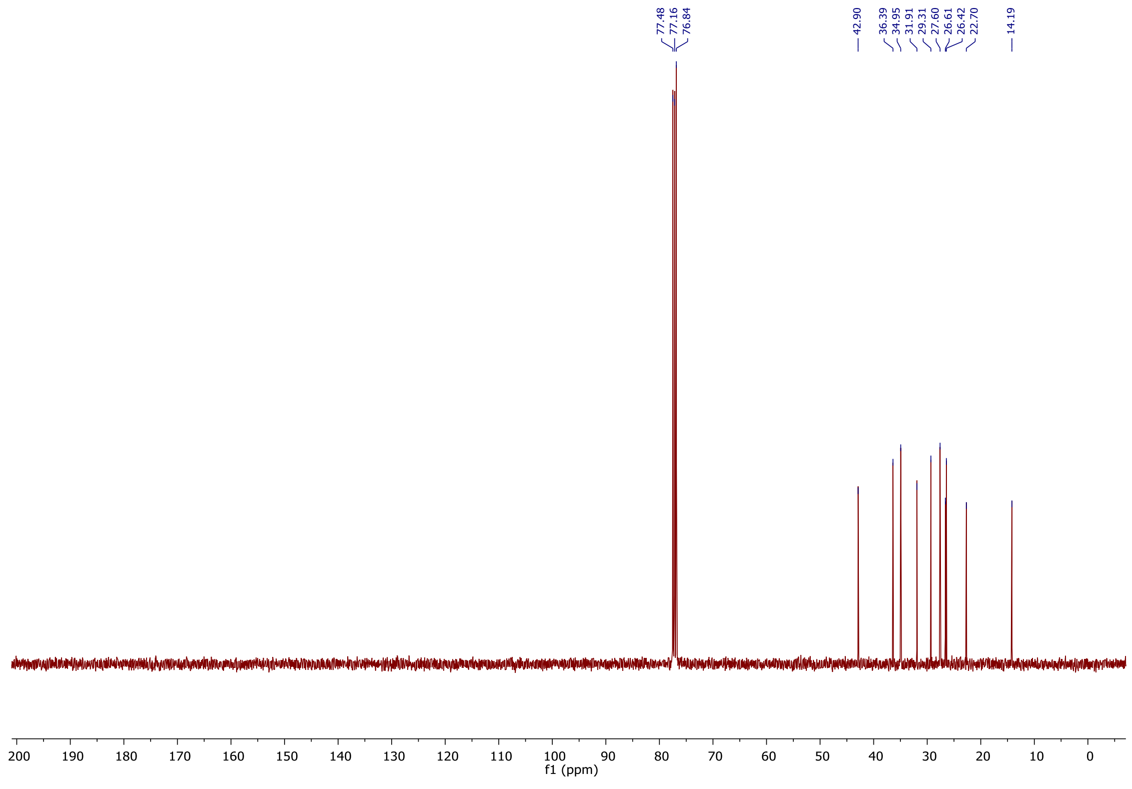

400 MHz ^{1}H NMR spectrum; 100.6 MHz ^{13}C NMR spectrum; $CDCl_3$ of **8i**

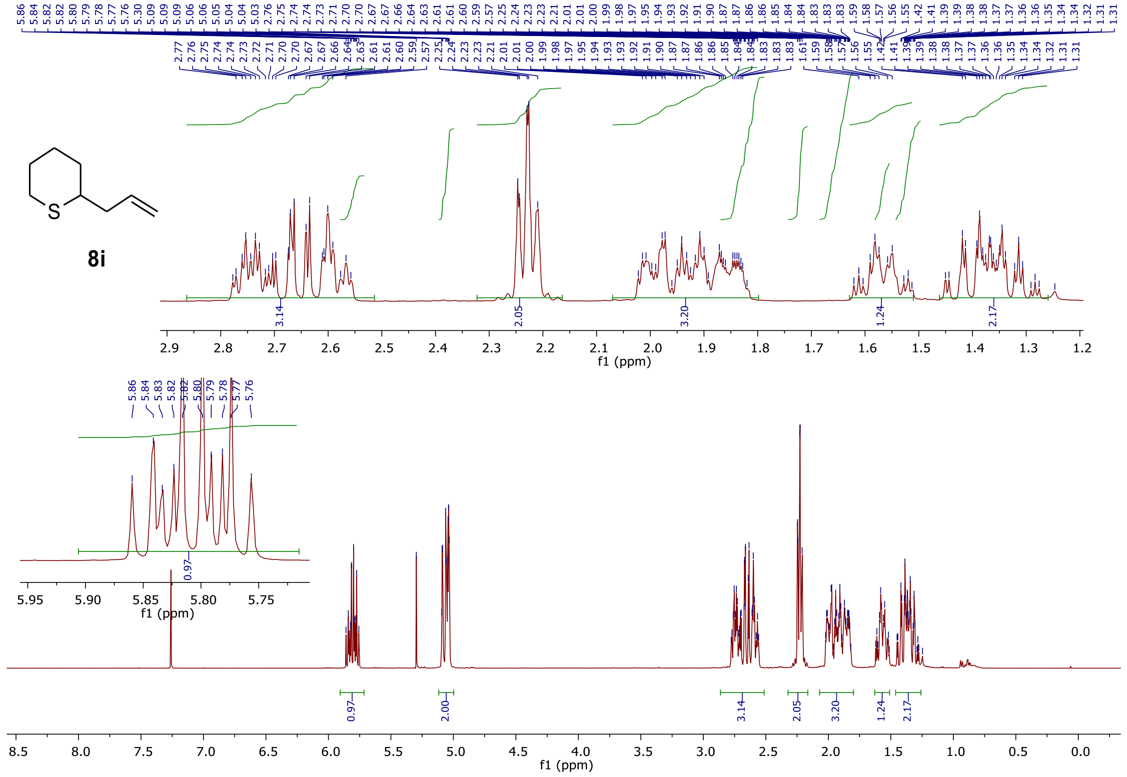

8i

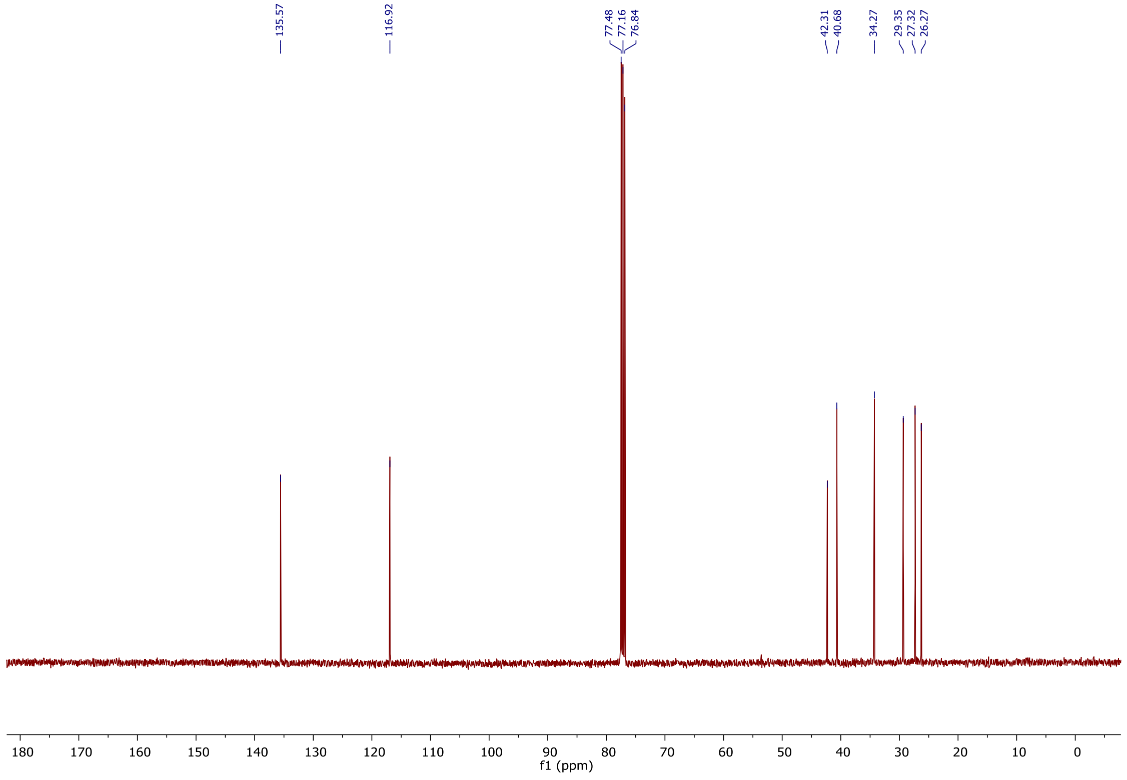

400 MHz ^{1}H NMR spectrum; 100.6 MHz ^{13}C NMR spectrum; $CDCl_3$ of **8j**

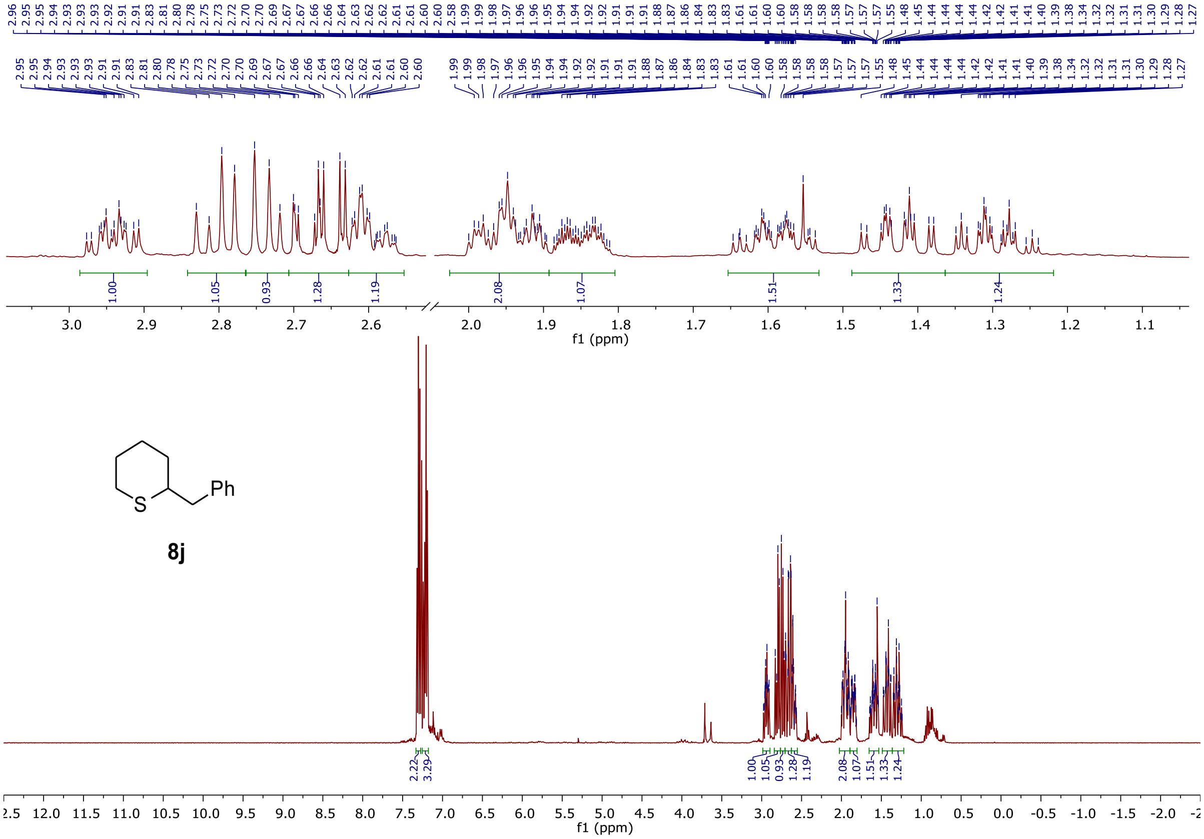

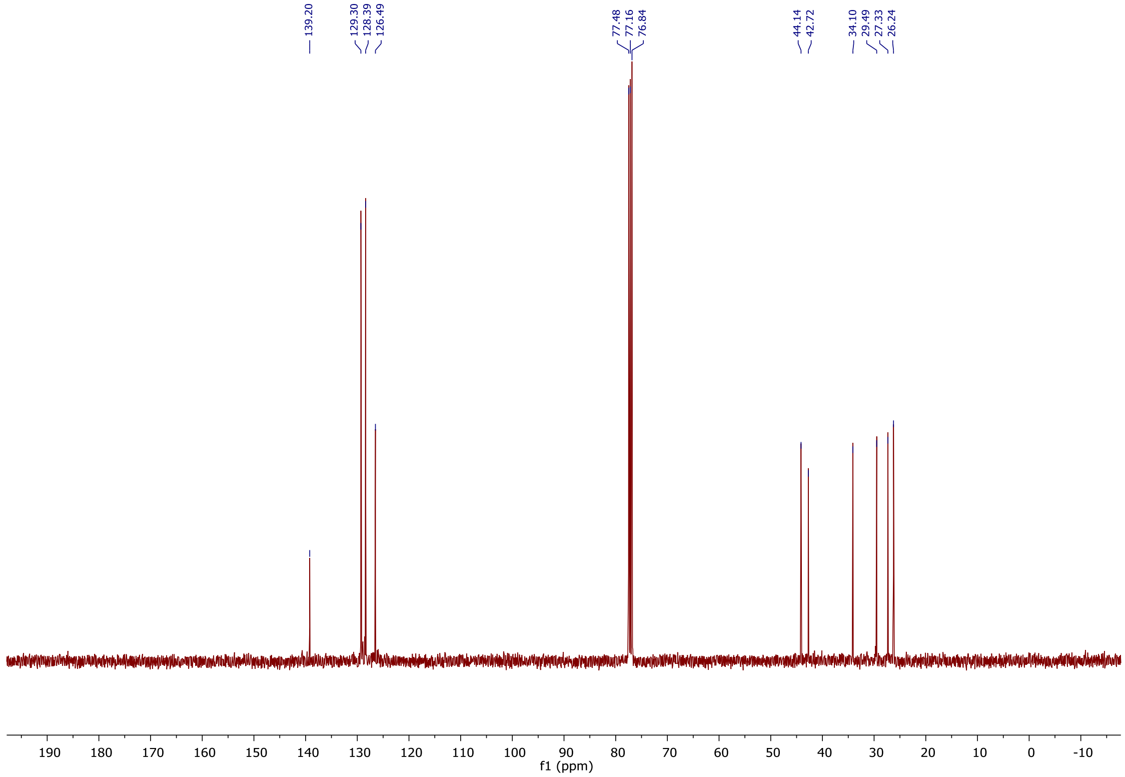

400 MHz ^{1}H NMR spectrum; 100.6 MHz ^{13}C NMR spectrum; $CDCl_3$ of **8k**

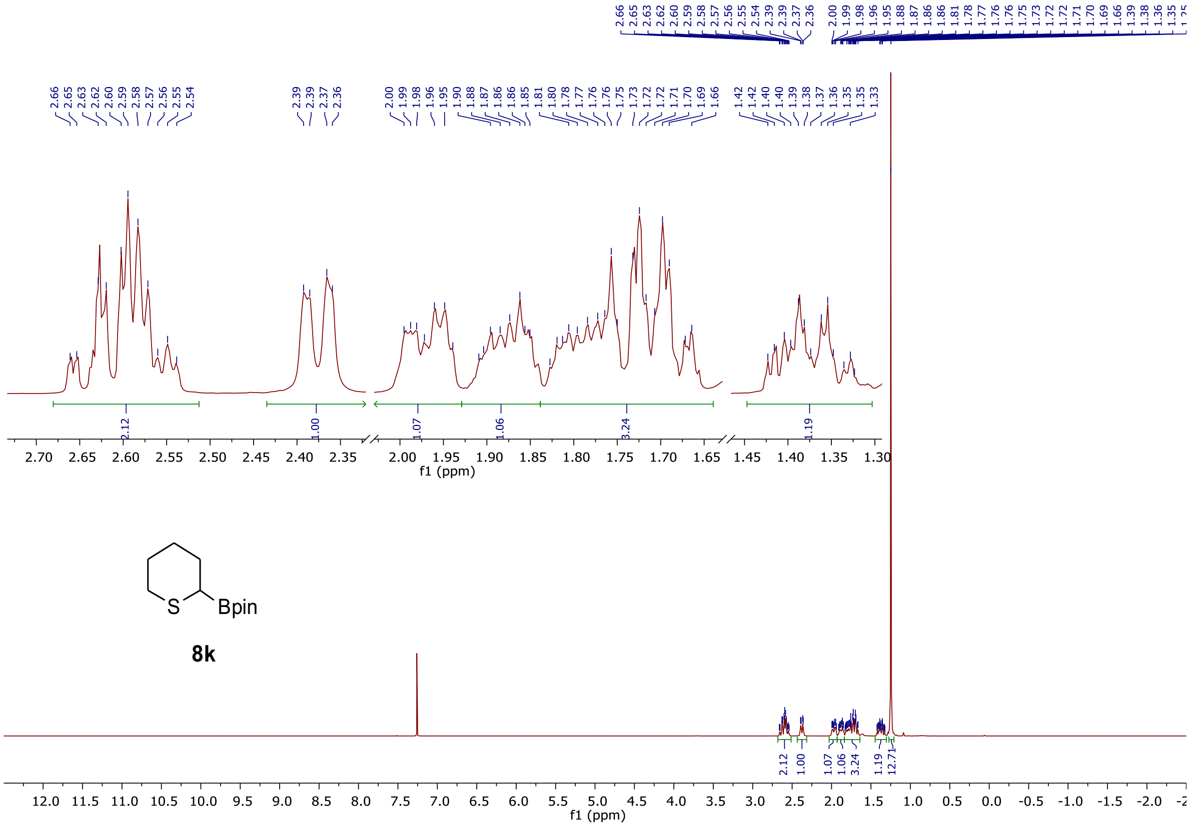

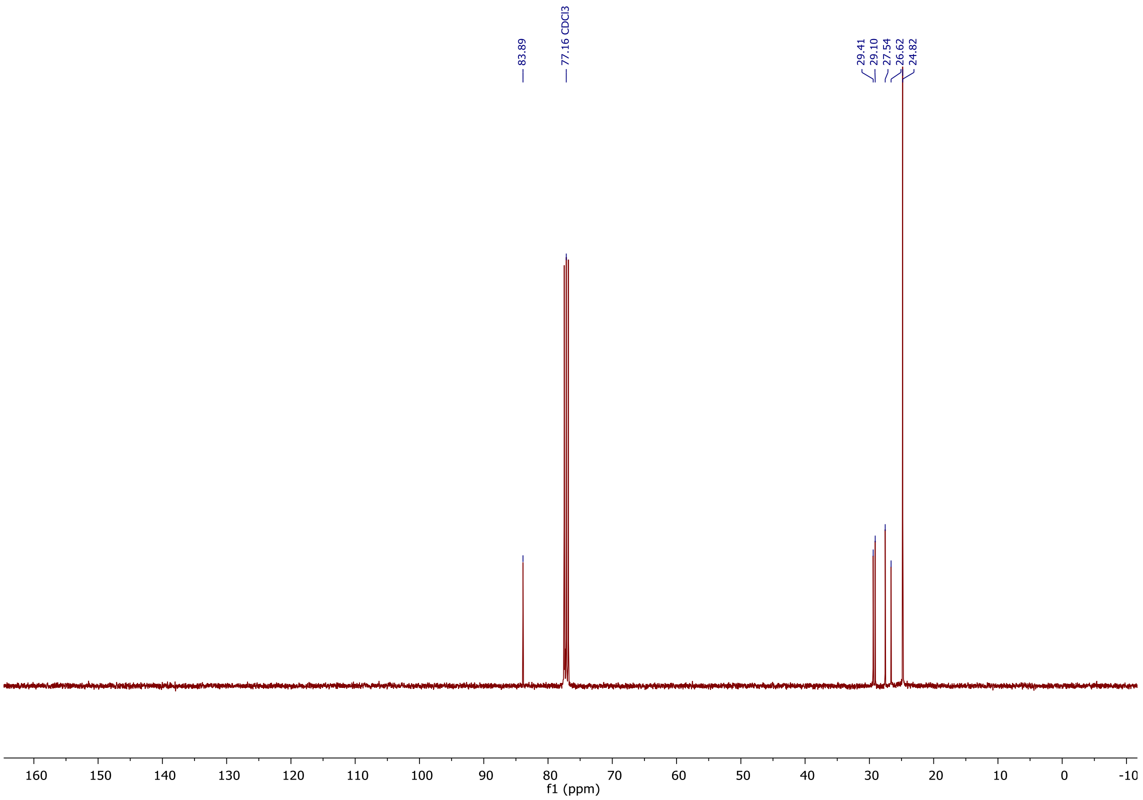

400 MHz ^1H NMR spectrum; 100.6 MHz ^{13}C NMR spectrum; $CDCl_3$ of **8l**

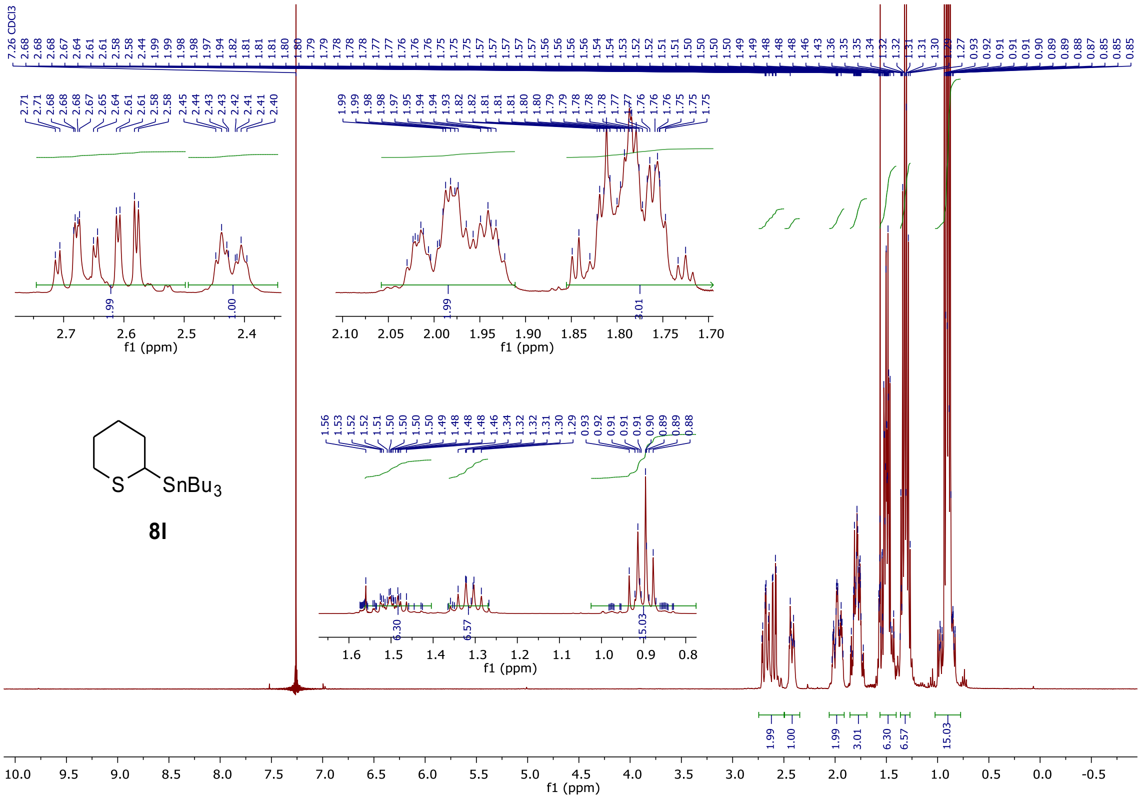

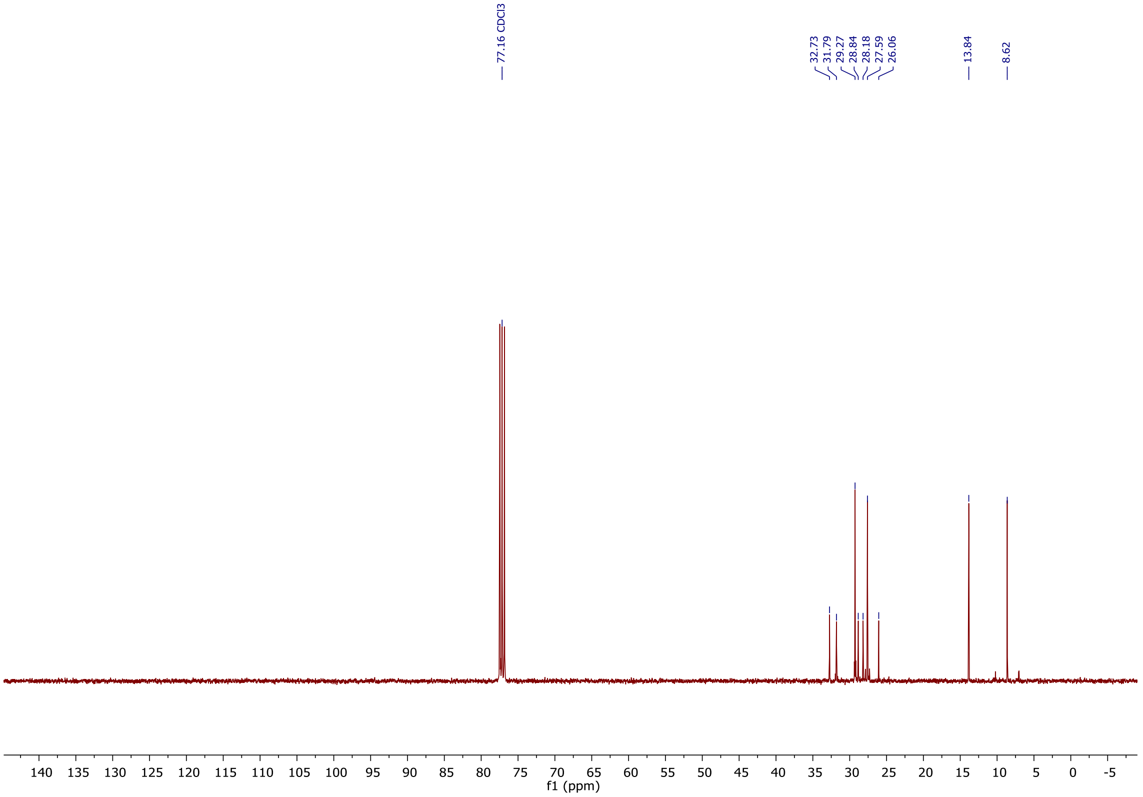

400 MHz ^{1}H NMR spectrum; 100.6 MHz ^{13}C NMR spectrum; $CDCl_3$ of **6**

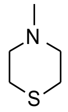

6

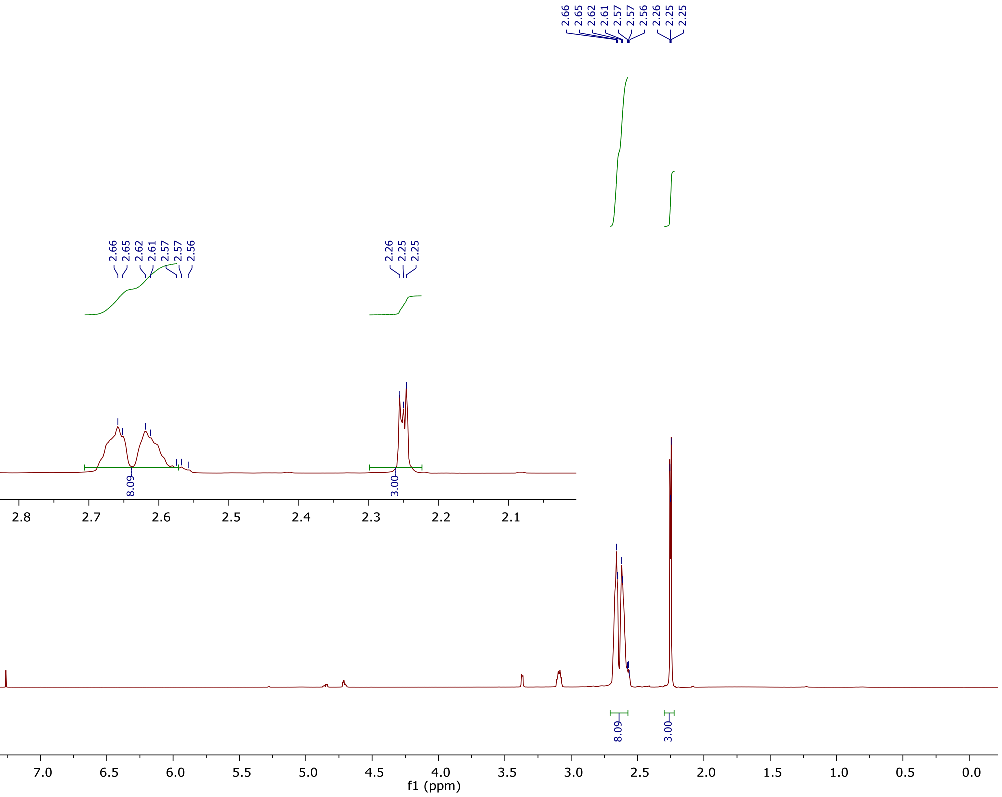

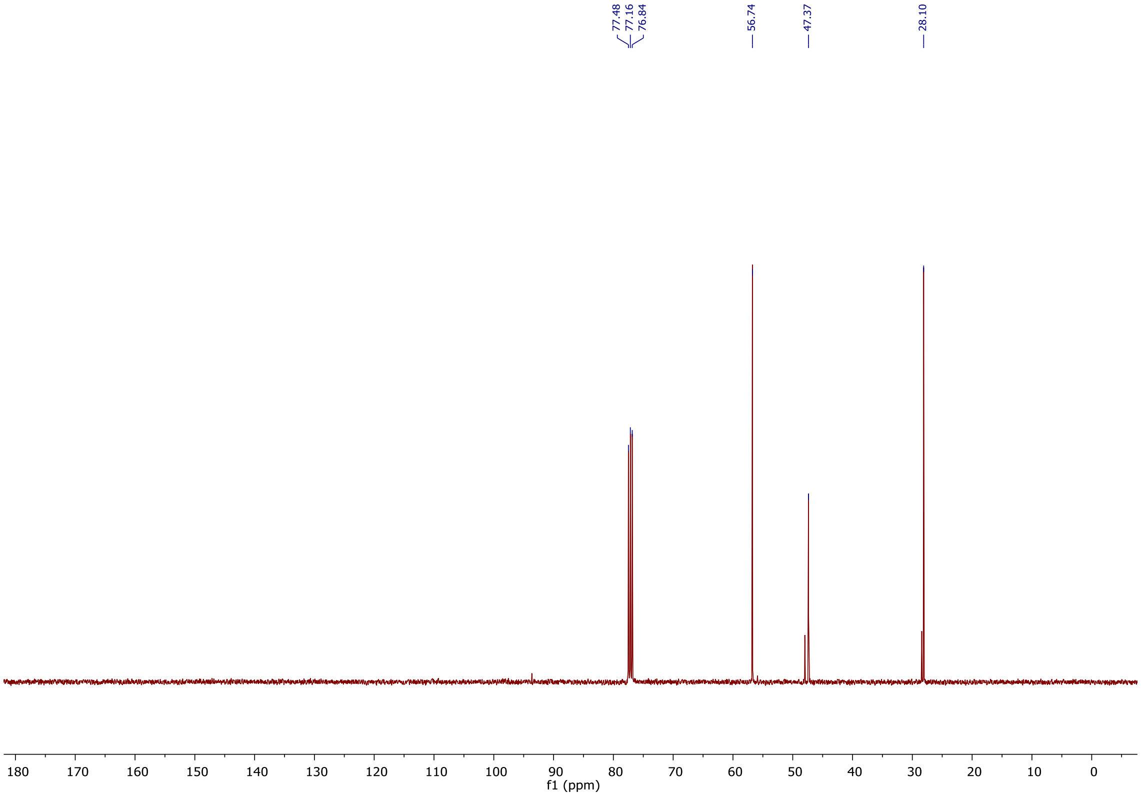

400 MHz ^{1}H NMR spectrum; 100.6 MHz ^{13}C NMR spectrum; $CDCl_3$ of **9aa**

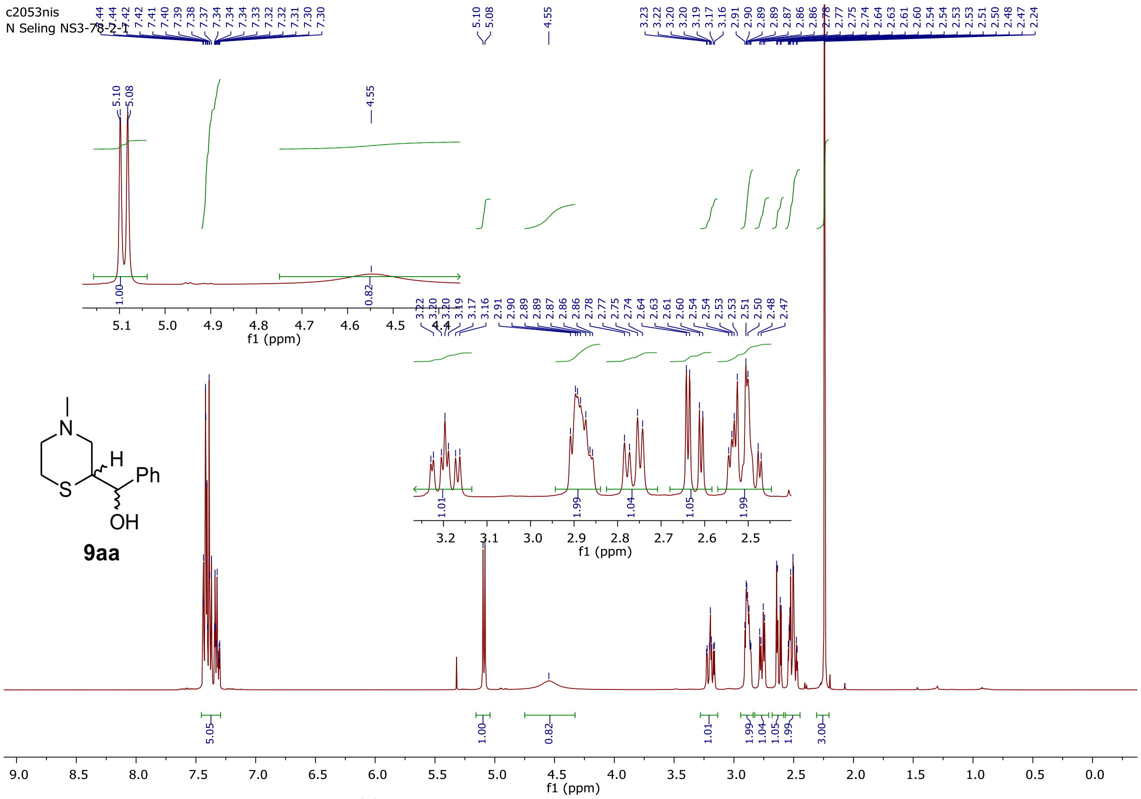

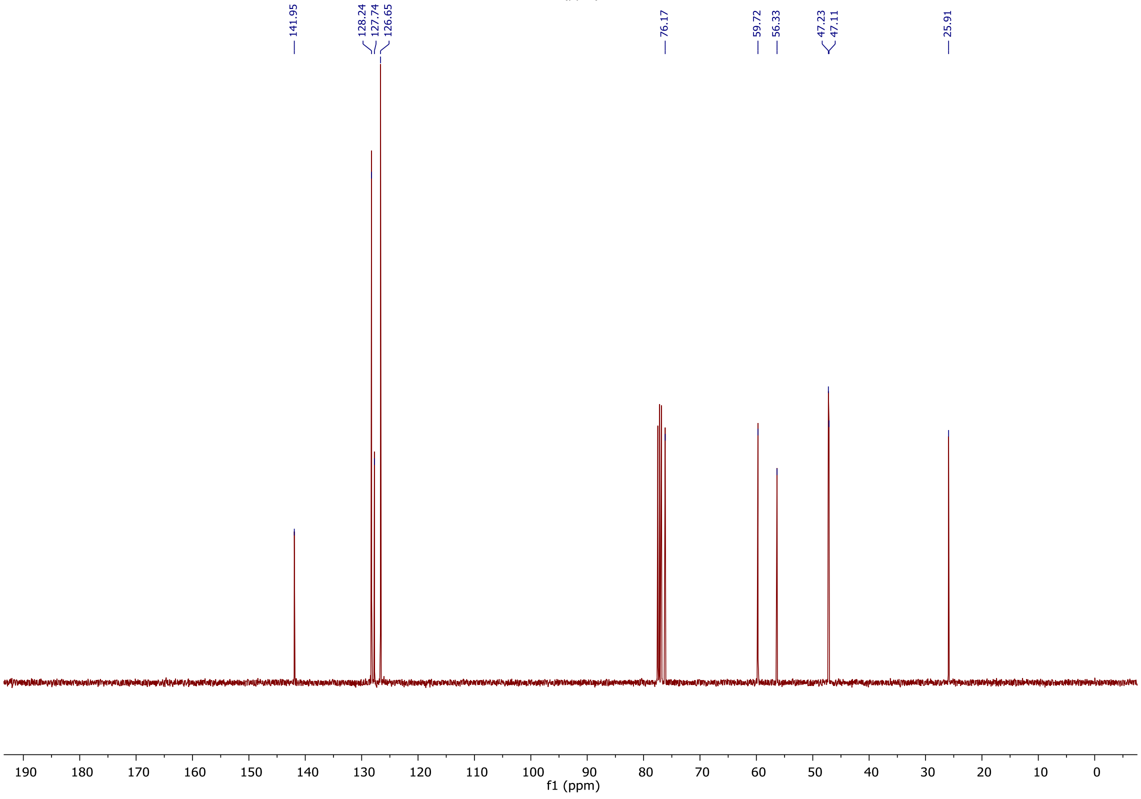

400 MHz ^{1}H NMR spectrum; 100.6 MHz ^{13}C NMR spectrum; $CDCl_3$ of **9ab**

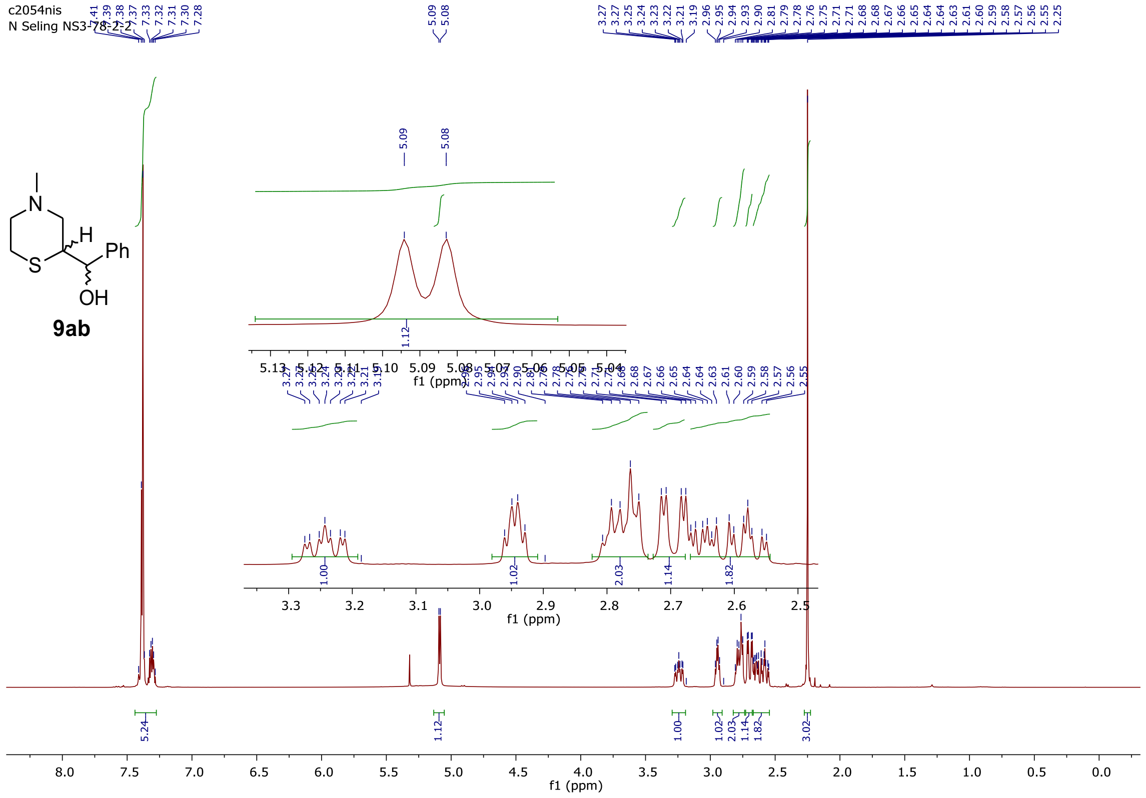

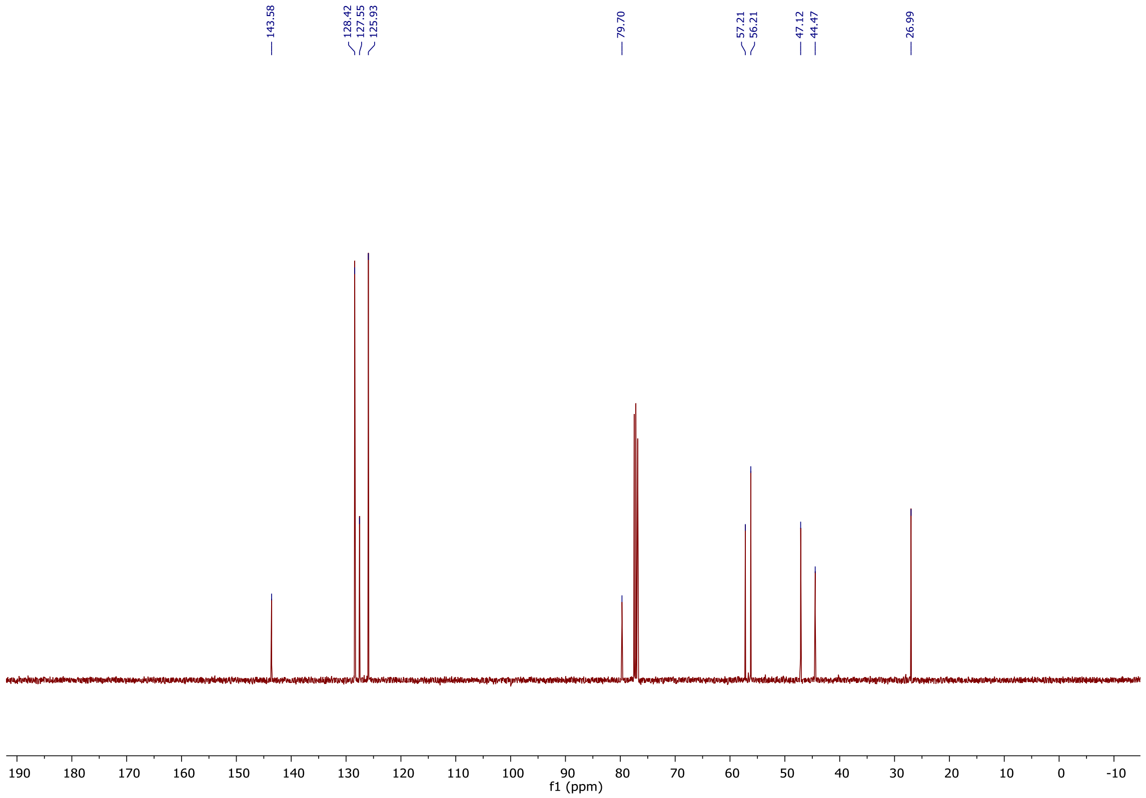

400 MHz ^{1}H NMR spectrum; 100.6 MHz ^{13}C NMR spectrum; $CDCl_3$ of **9b**

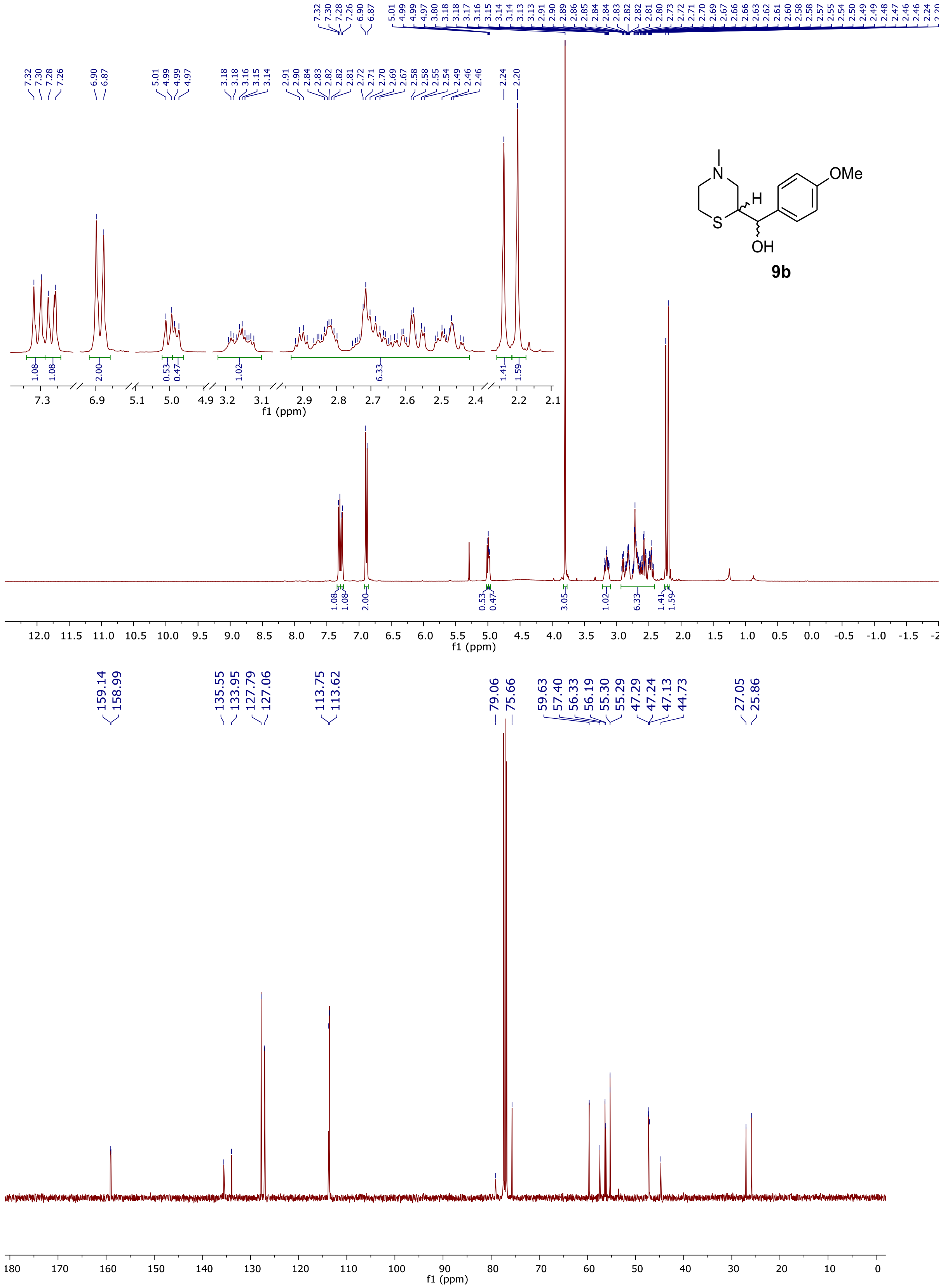

400 MHz ^{1}H NMR spectrum; 100.6 MHz ^{13}C NMR spectrum; $CDCl_3$ of **9c**

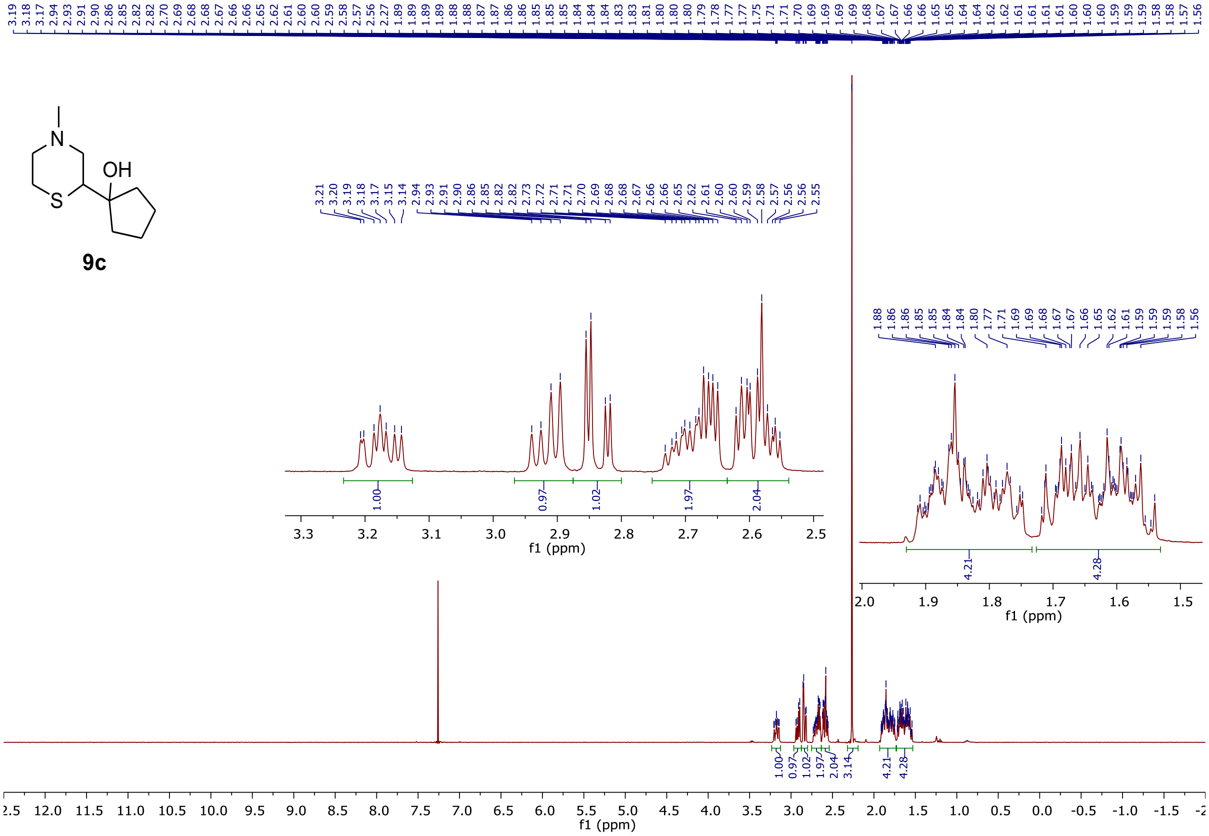

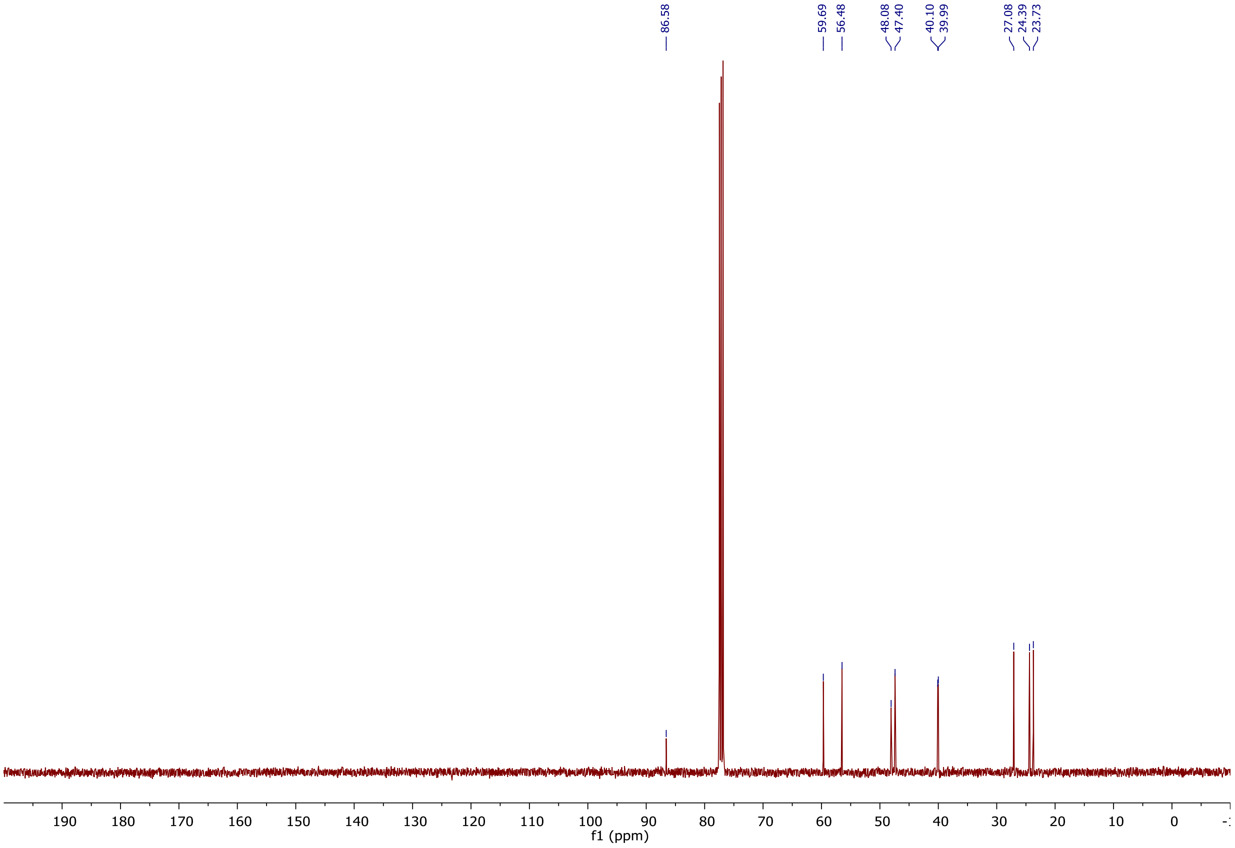

400 MHz ^{1}H NMR spectrum; 100.6 MHz ^{13}C NMR spectrum; $CDCl_3$ of **9d**

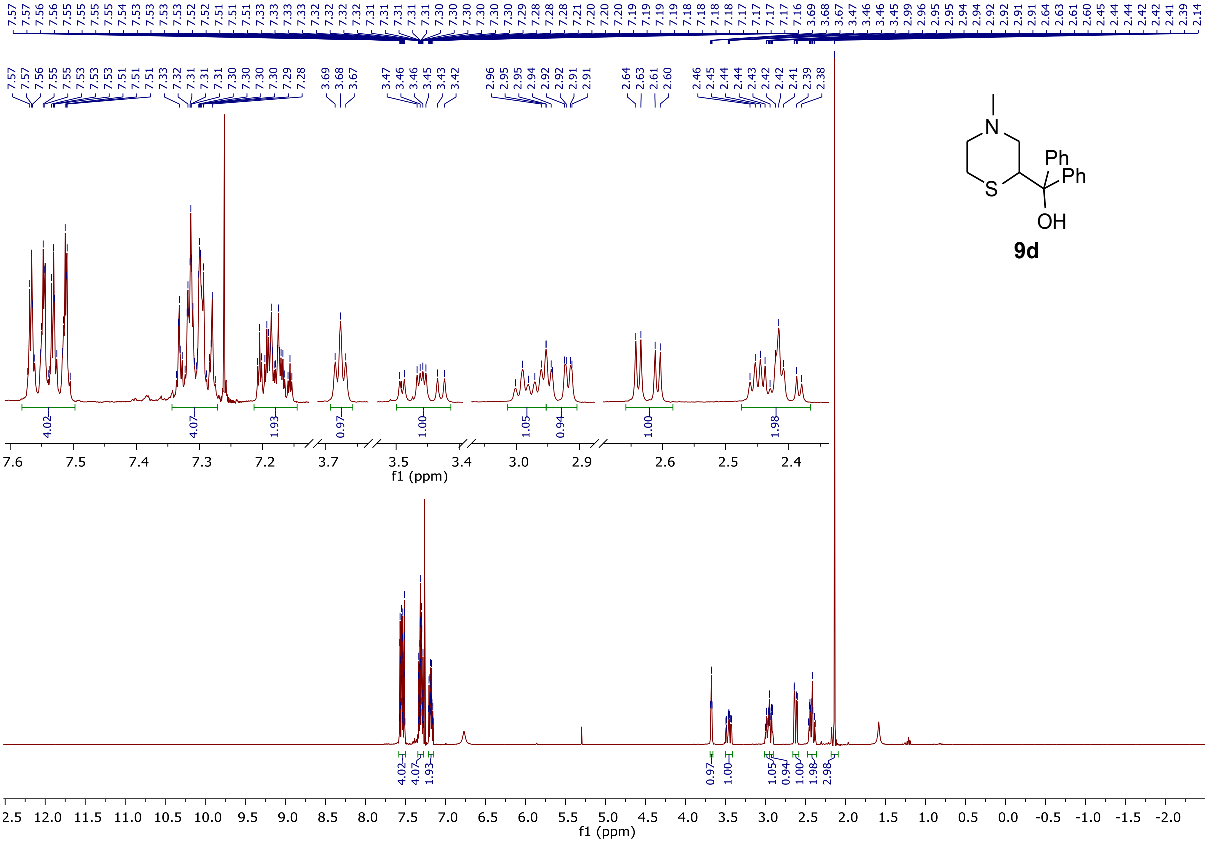

9d

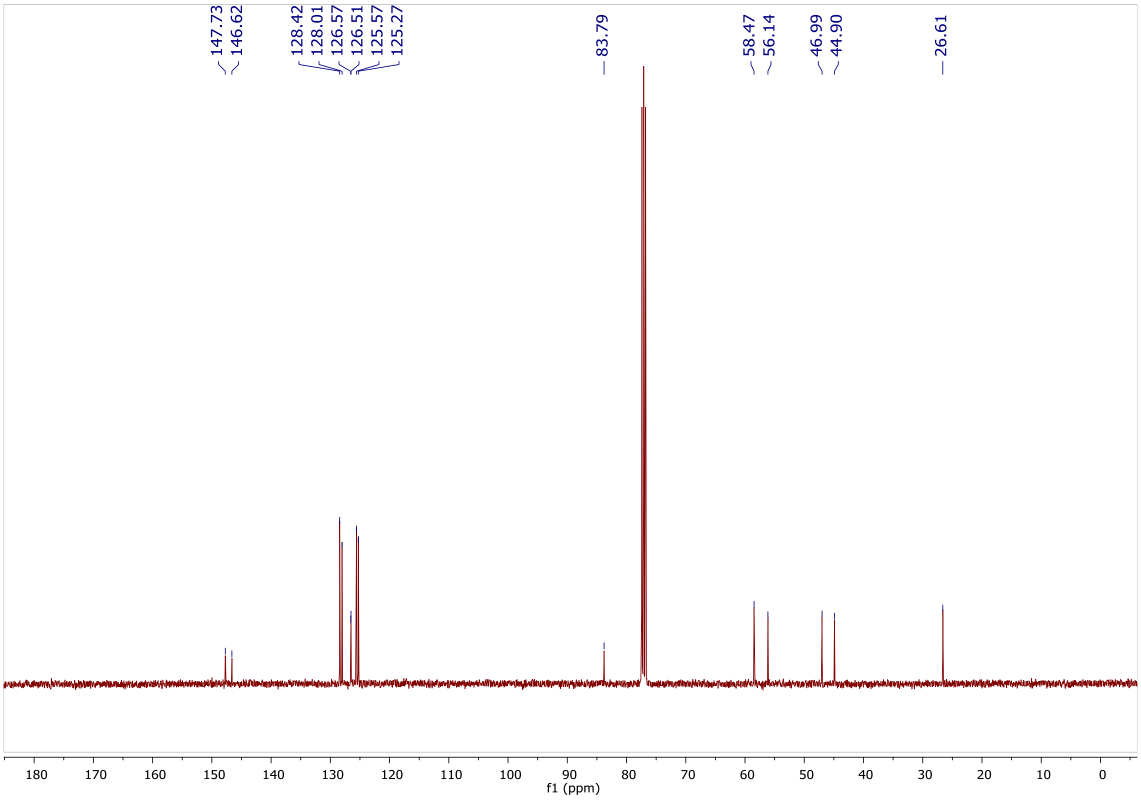

400 MHz ^{1}H NMR spectrum; 100.6 MHz ^{13}C NMR spectrum; $CDCl_3$ of **9e**

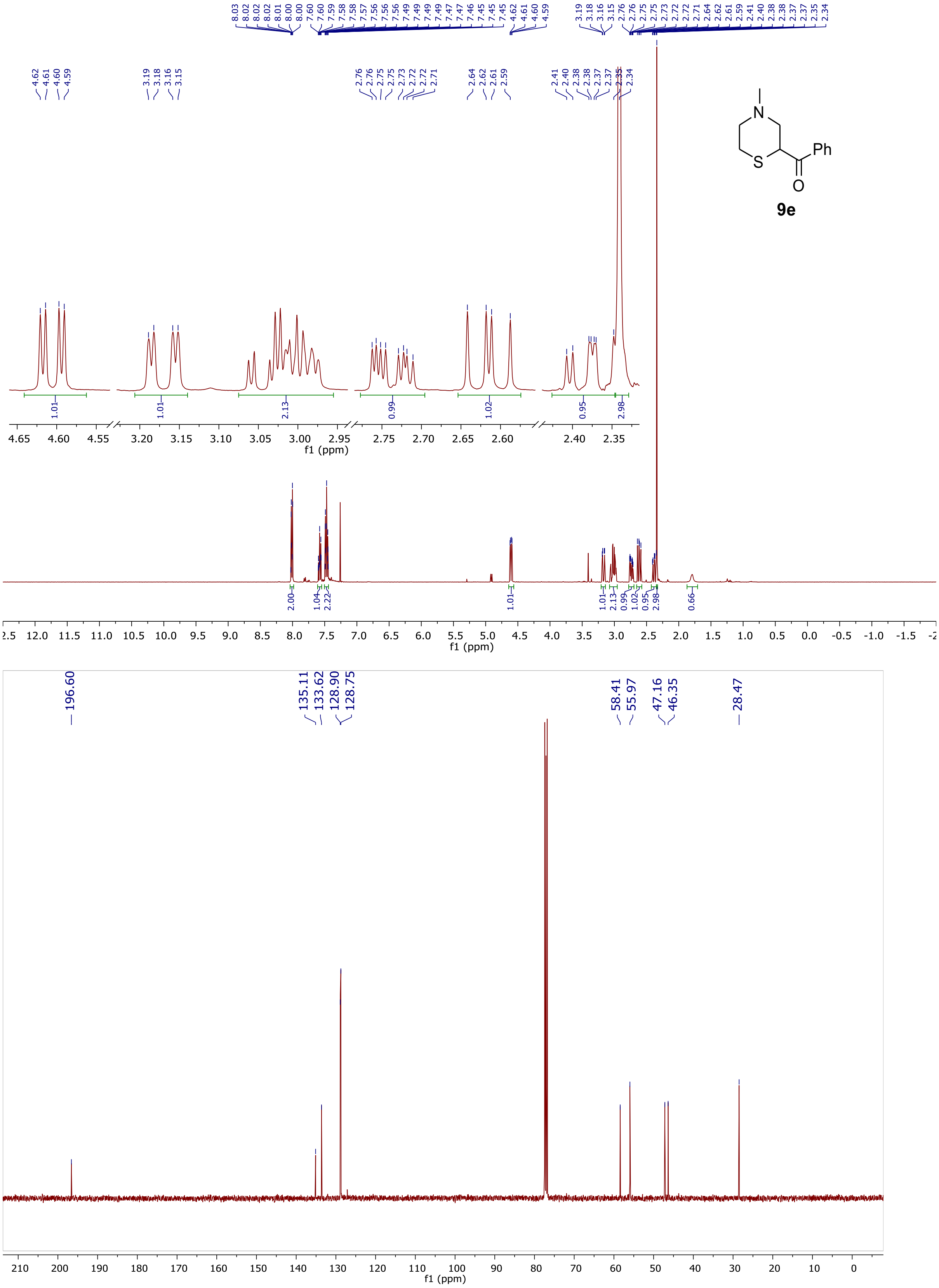

9e

400 MHz ^{1}H NMR spectrum; 100.6 MHz ^{13}C NMR spectrum; $CDCl_3$ of **9f**

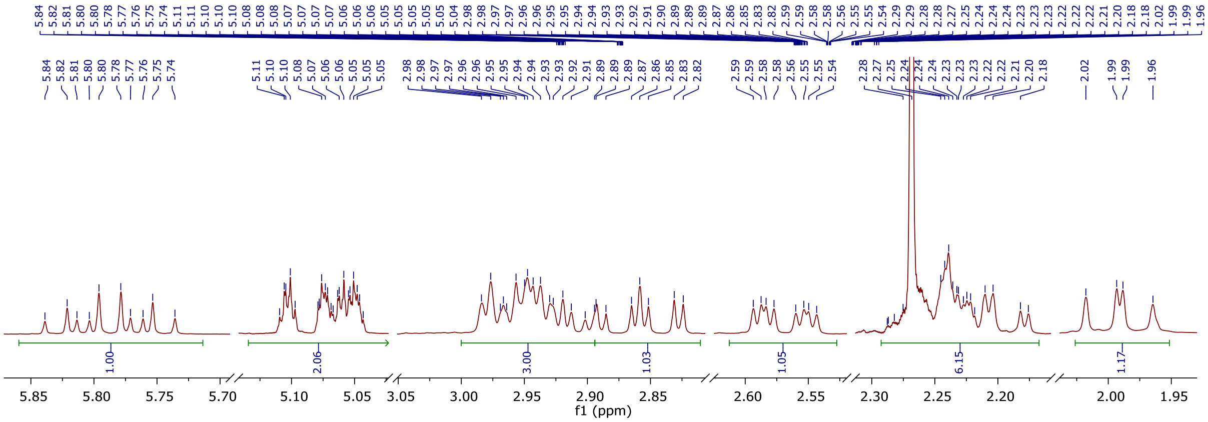

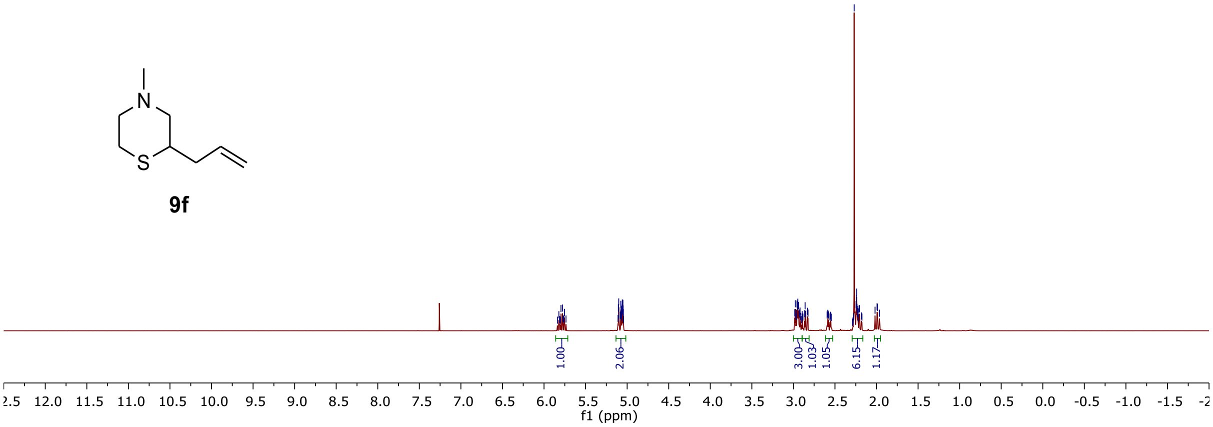

9f

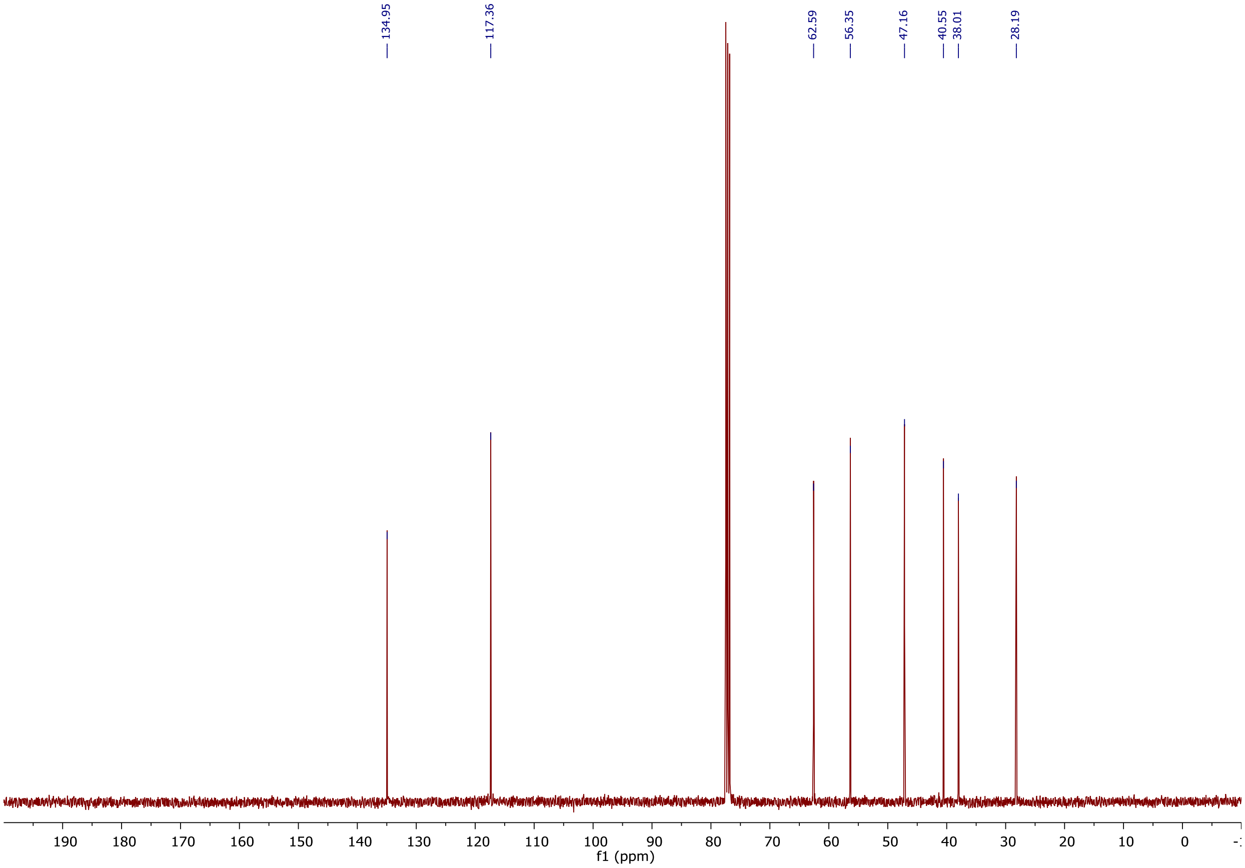

400 MHz ^{1}H NMR spectrum; 100.6 MHz ^{13}C NMR spectrum; $CDCl_3$ of **9g**

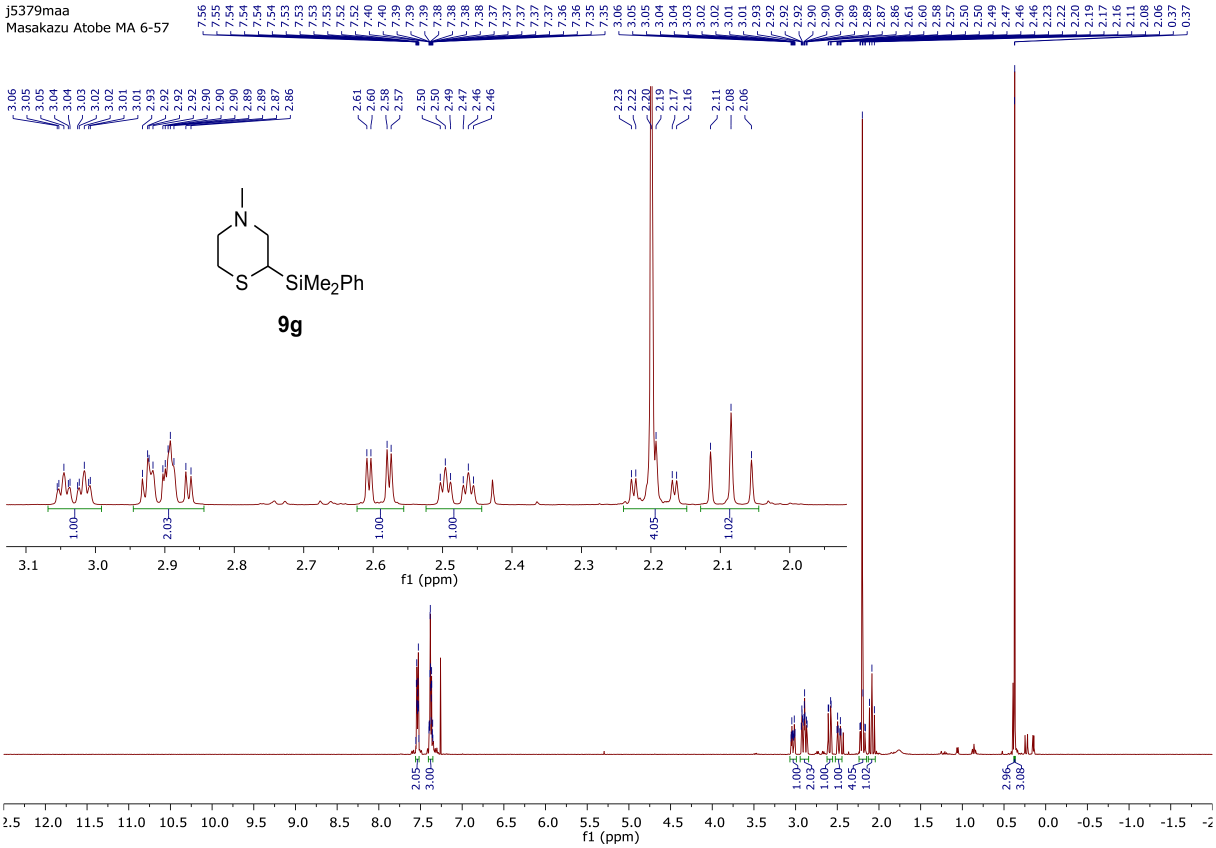

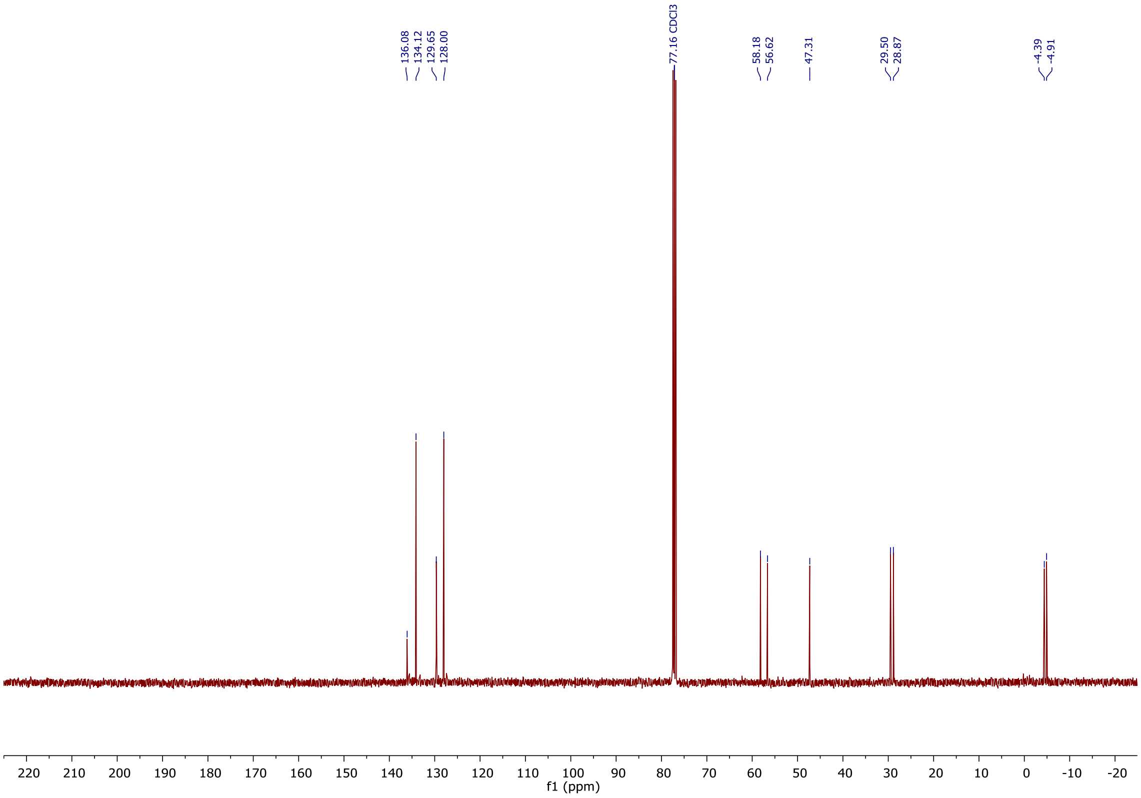

400 MHz ^1H NMR spectrum; 100.6 MHz ^{13}C NMR spectrum; $CDCl_3$ of **9h**

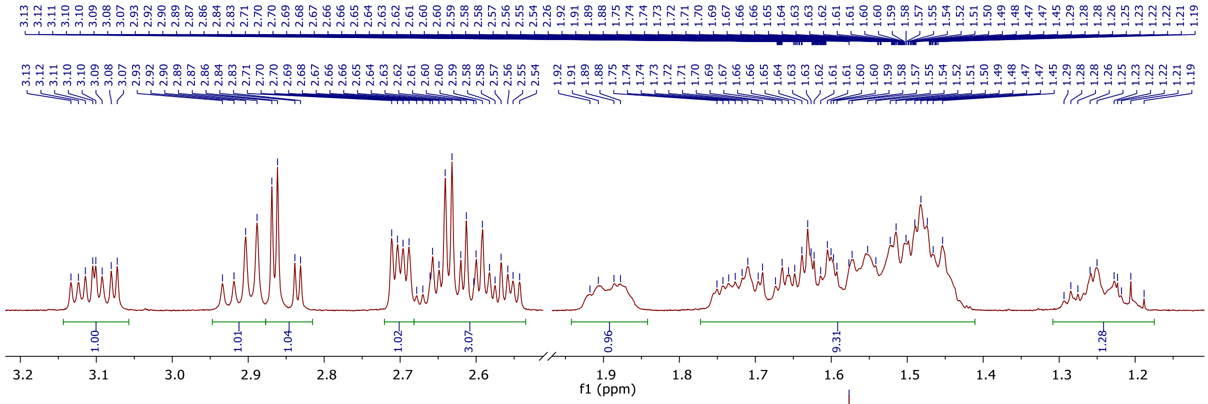

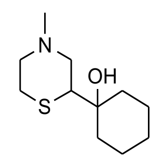

9h

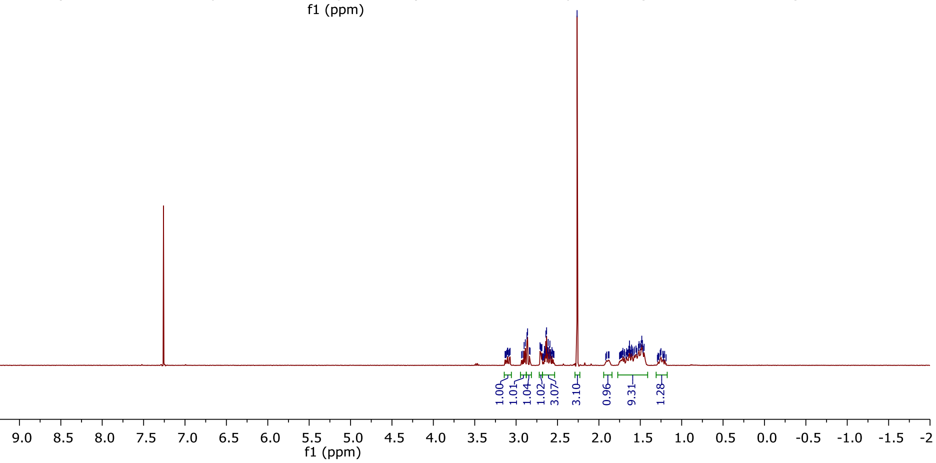

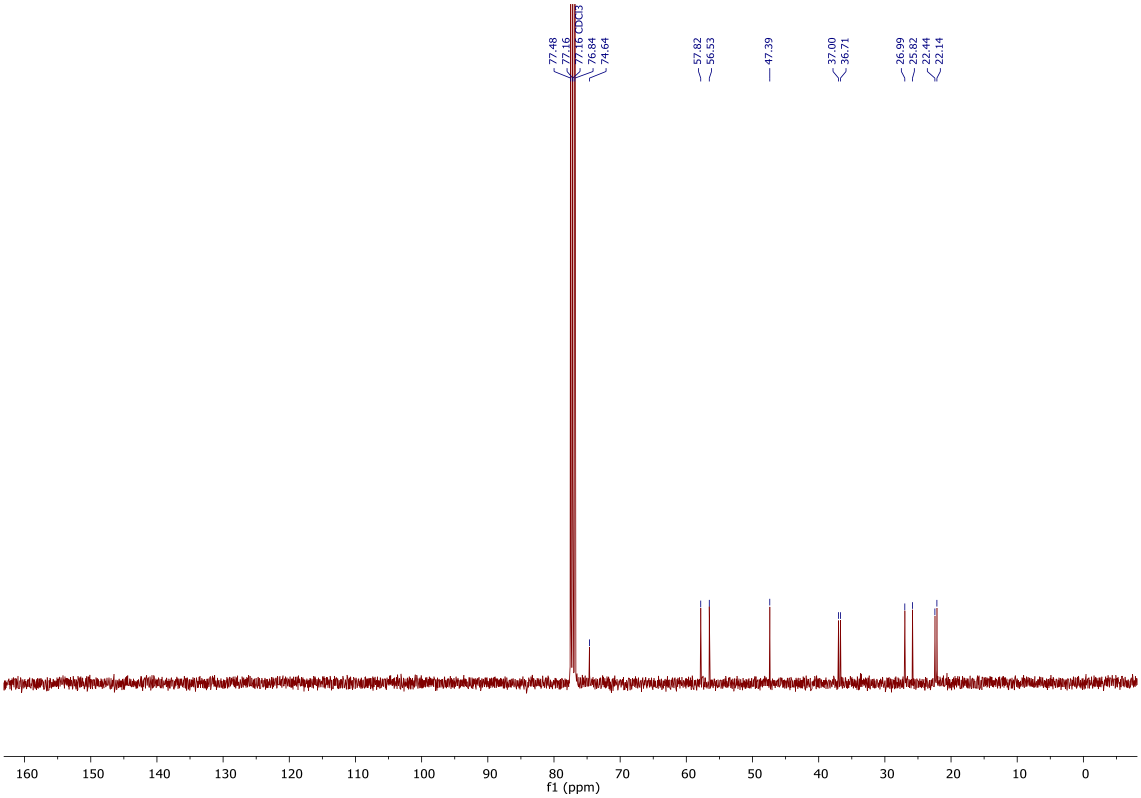

400 MHz ^{1}H NMR spectrum; 100.6 MHz ^{13}C NMR spectrum; $CDCl_3$ of **9i**

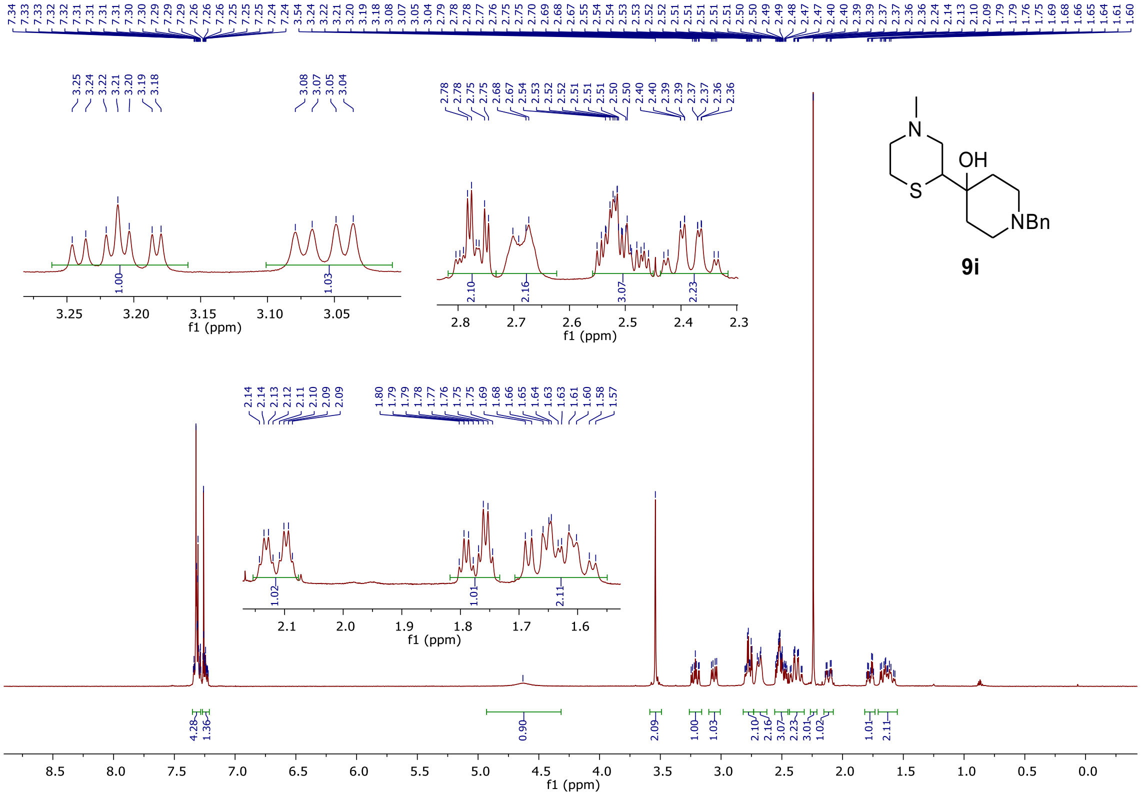

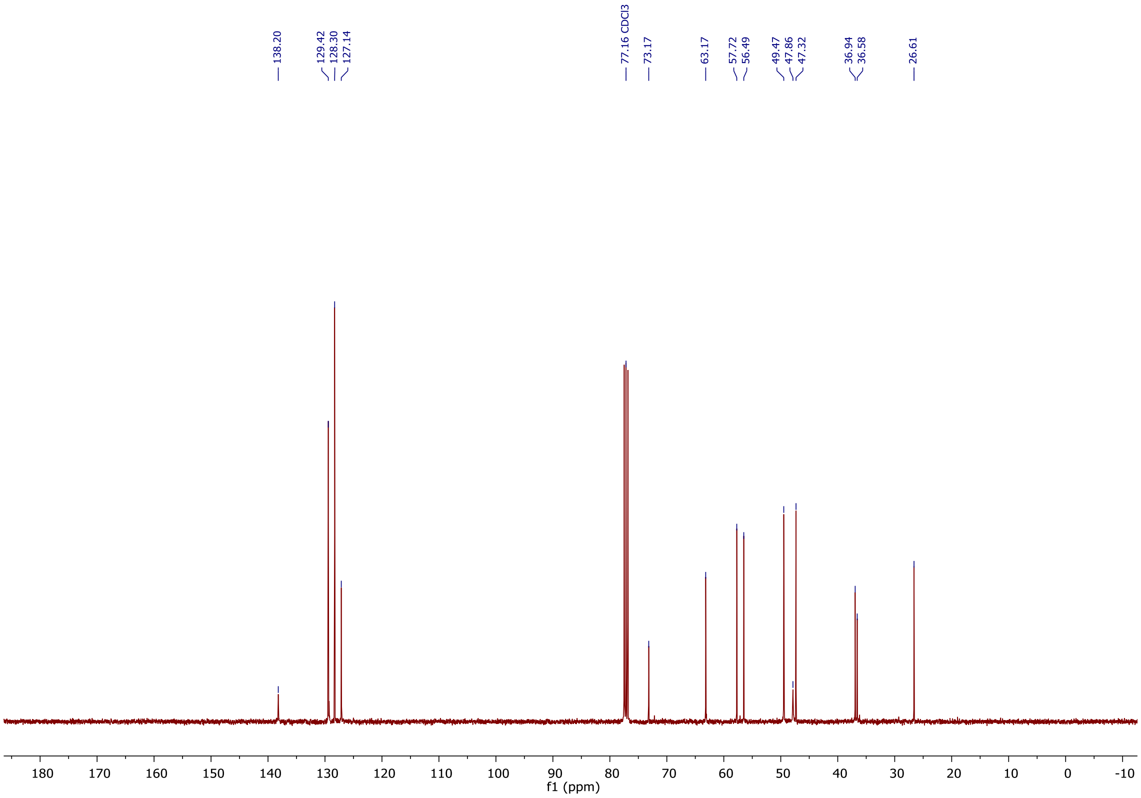

400 MHz ^{1}H NMR spectrum; 100.6 MHz ^{13}C NMR spectrum; $CDCl_3$ of **9ja**

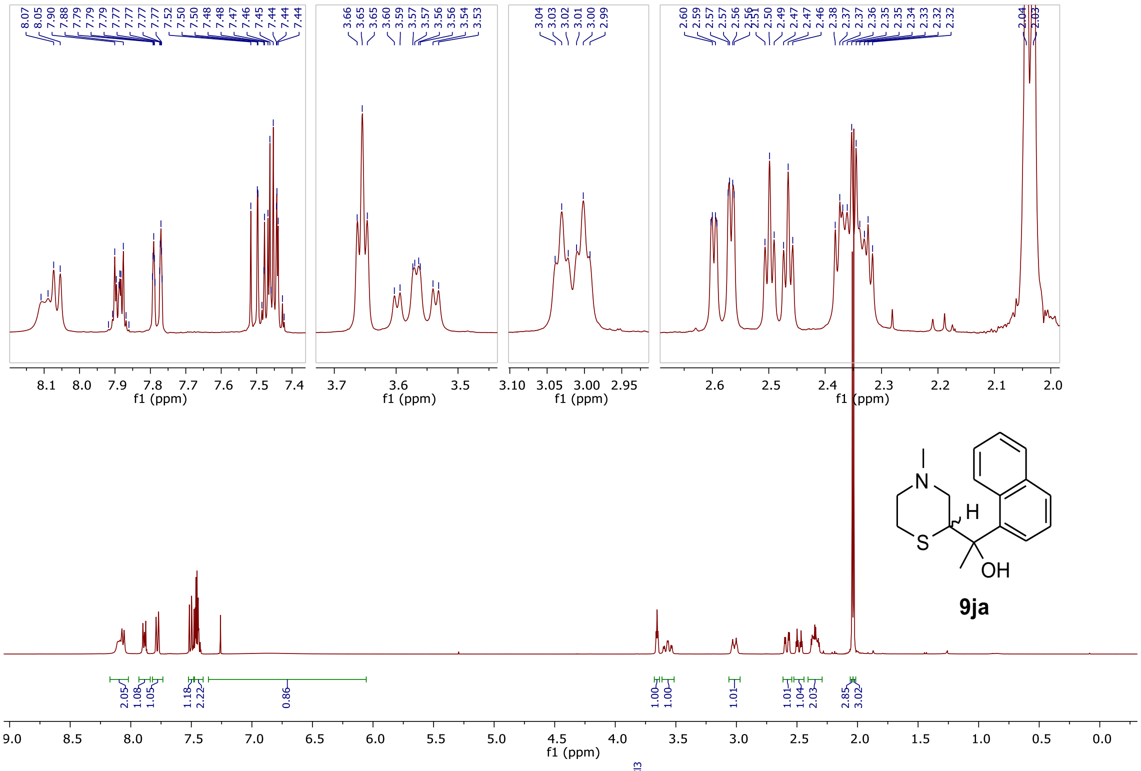

9ja

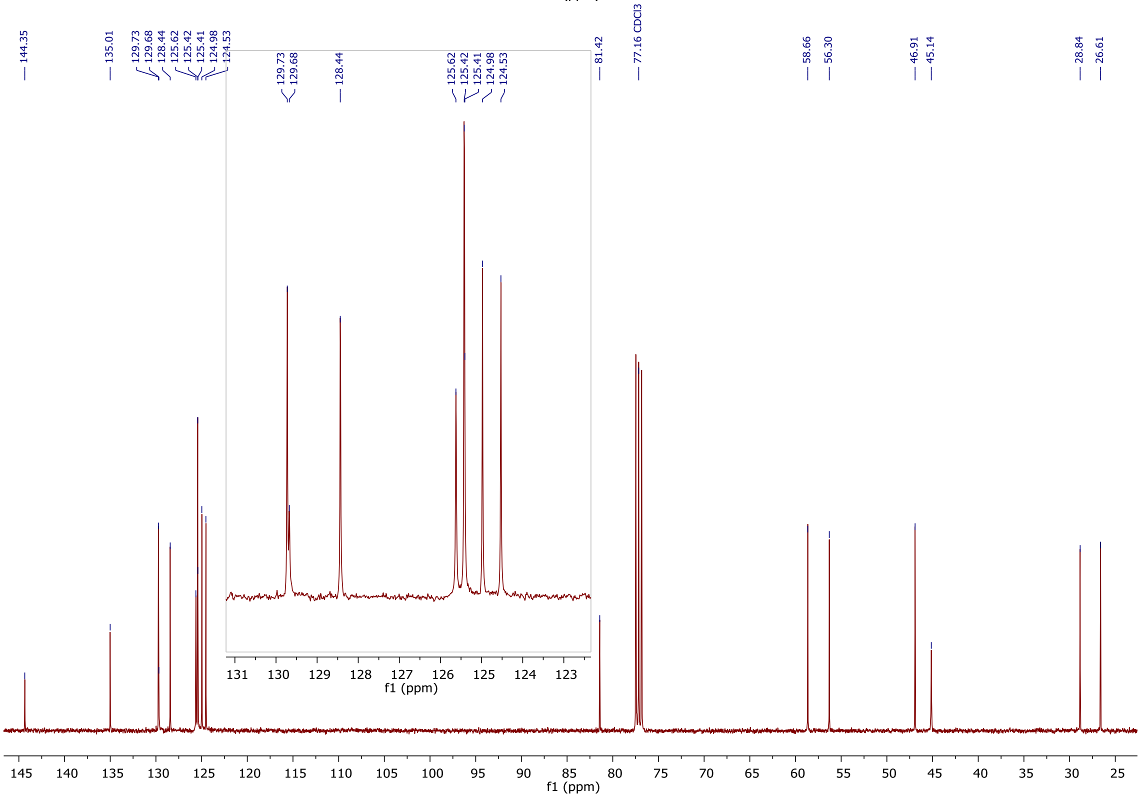

400 MHz ^{1}H NMR spectrum; 100.6 MHz ^{13}C NMR spectrum; $CDCl_3$ of **9jb**

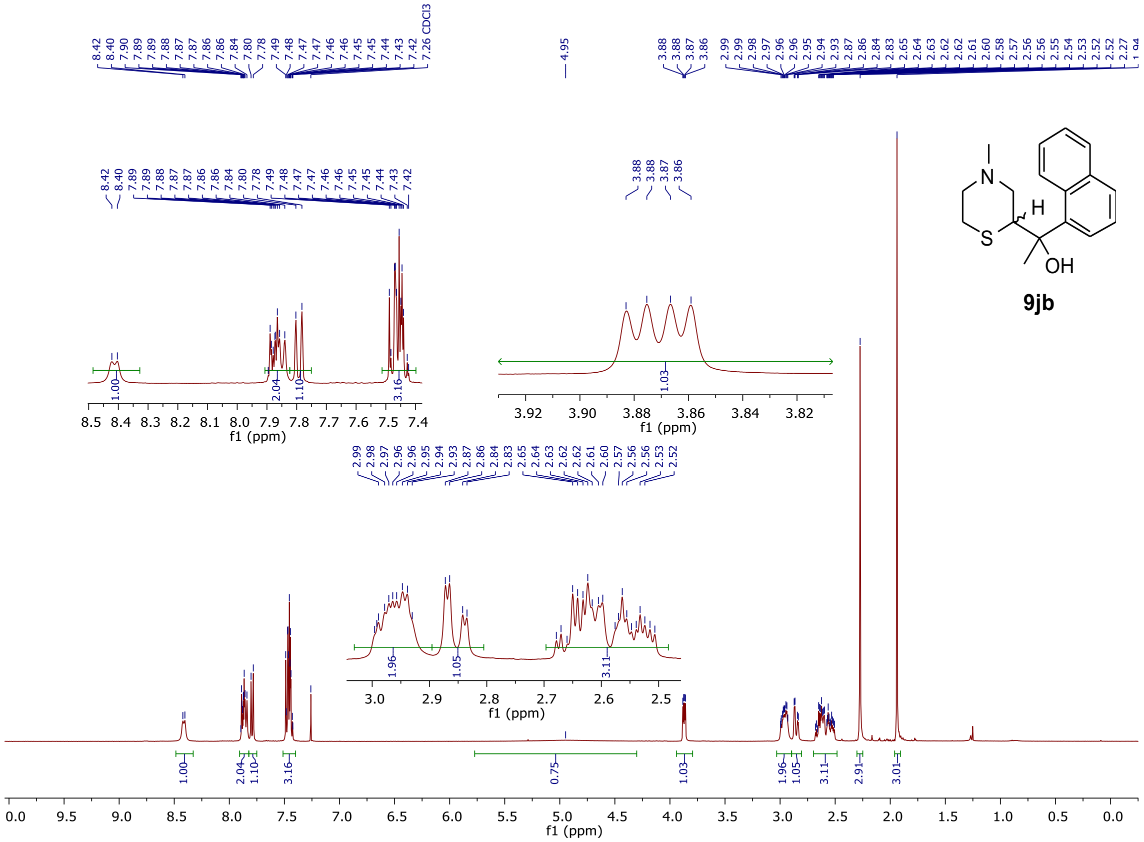

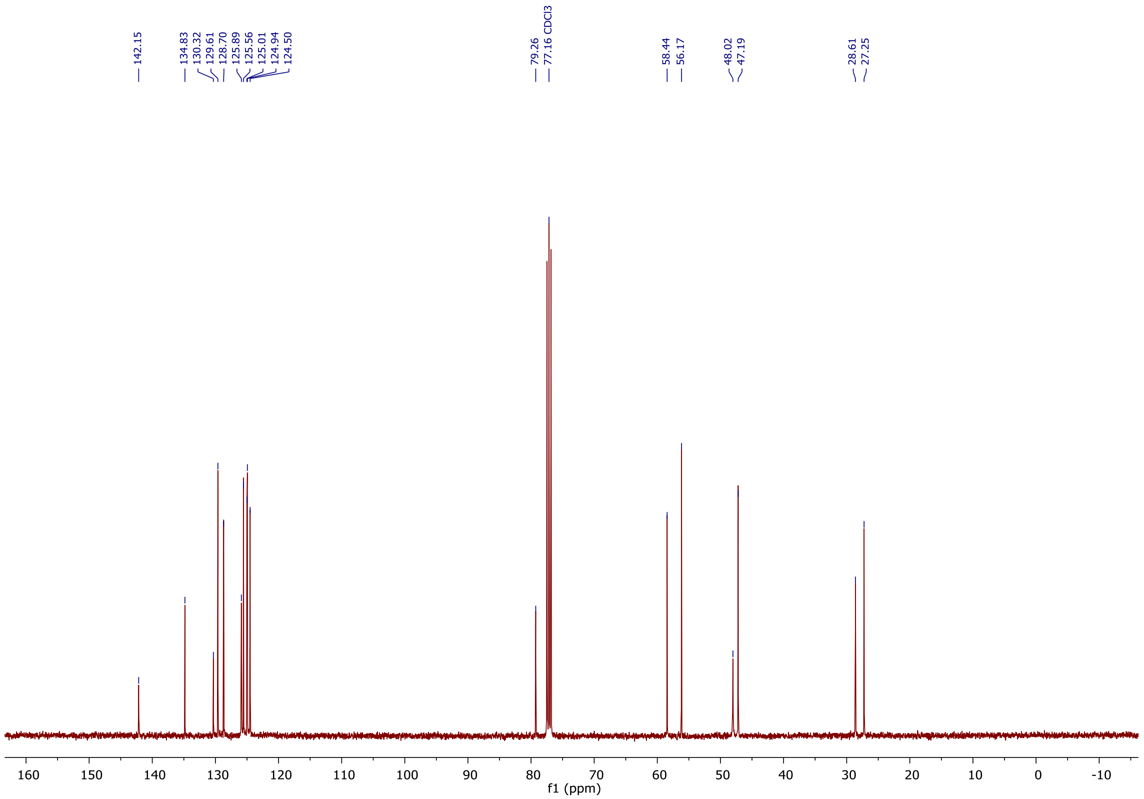

400 MHz ^1H NMR spectrum; 100.6 MHz ^{13}C NMR spectrum; $CDCl_3$ of **9k**

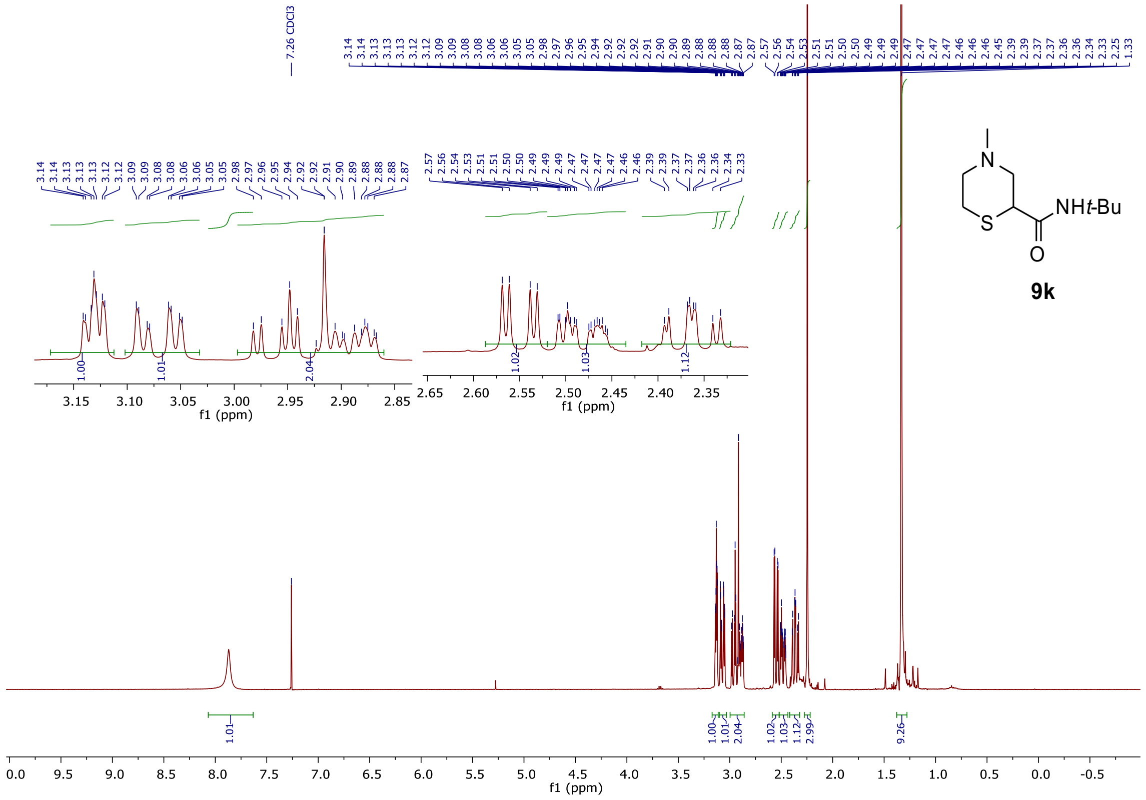

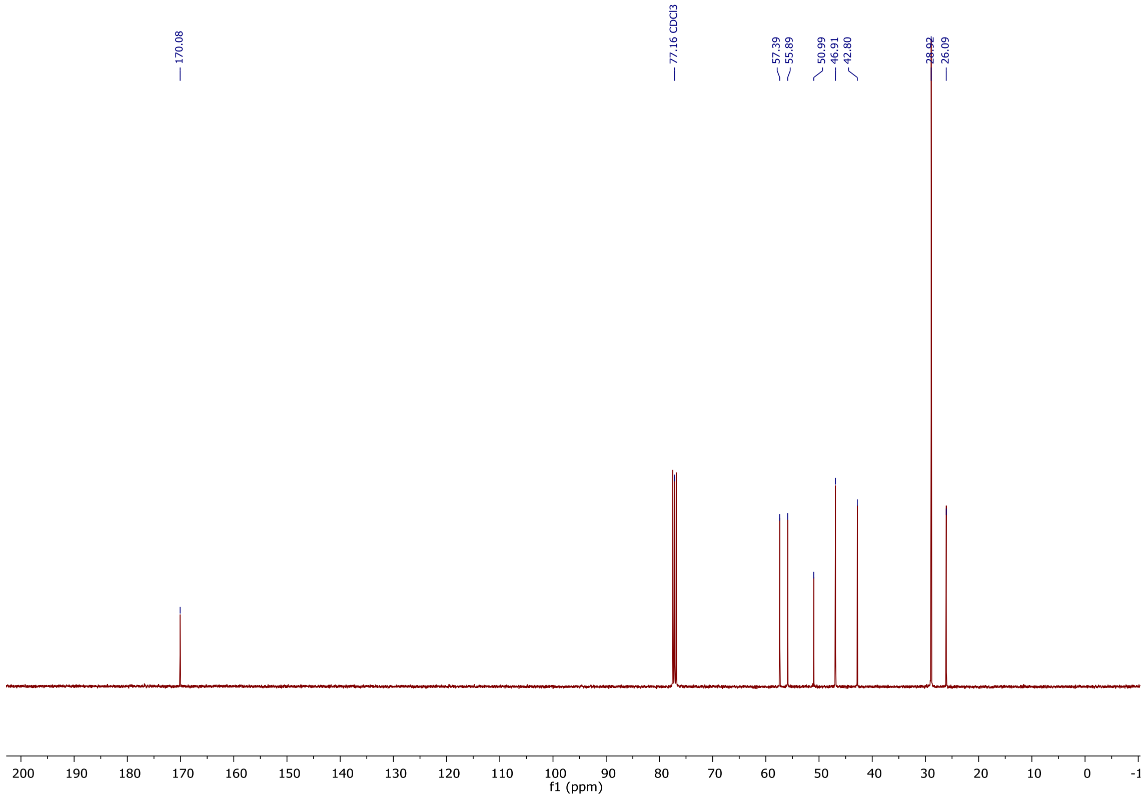

400 MHz ^1H NMR spectrum; 100.6 MHz ^{13}C NMR spectrum; $CDCl_3$ of **9l**

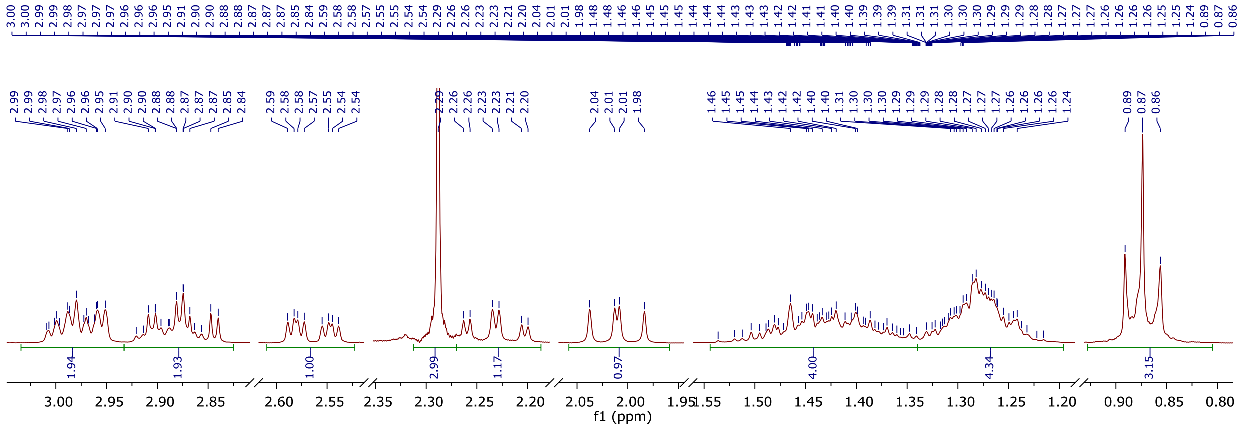

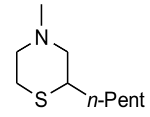

9l

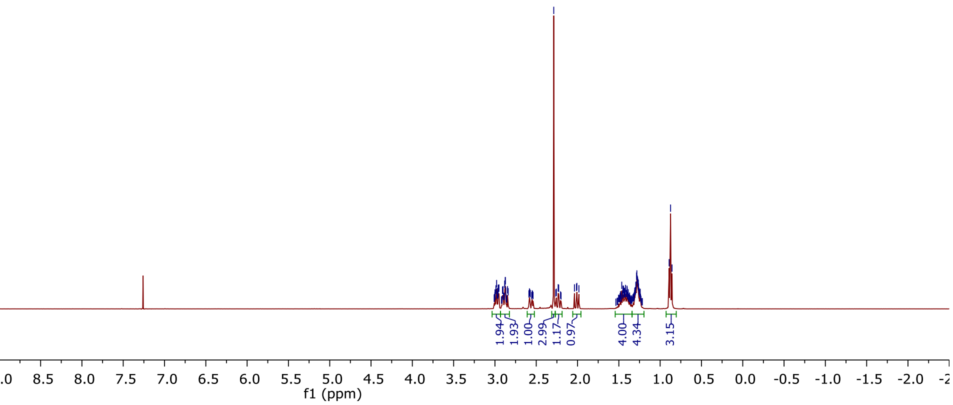

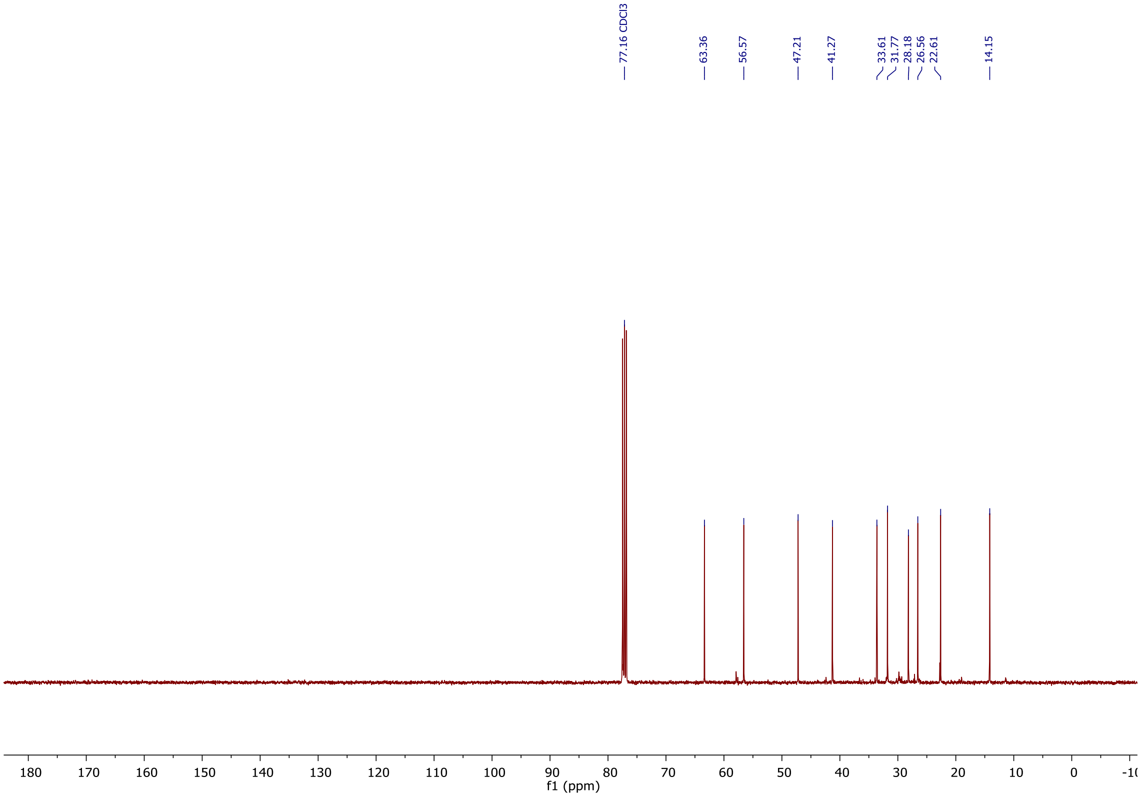

400 MHz ^{1}H NMR spectrum; 100.6 MHz ^{13}C NMR spectrum; $CDCl_3$ of **S3**

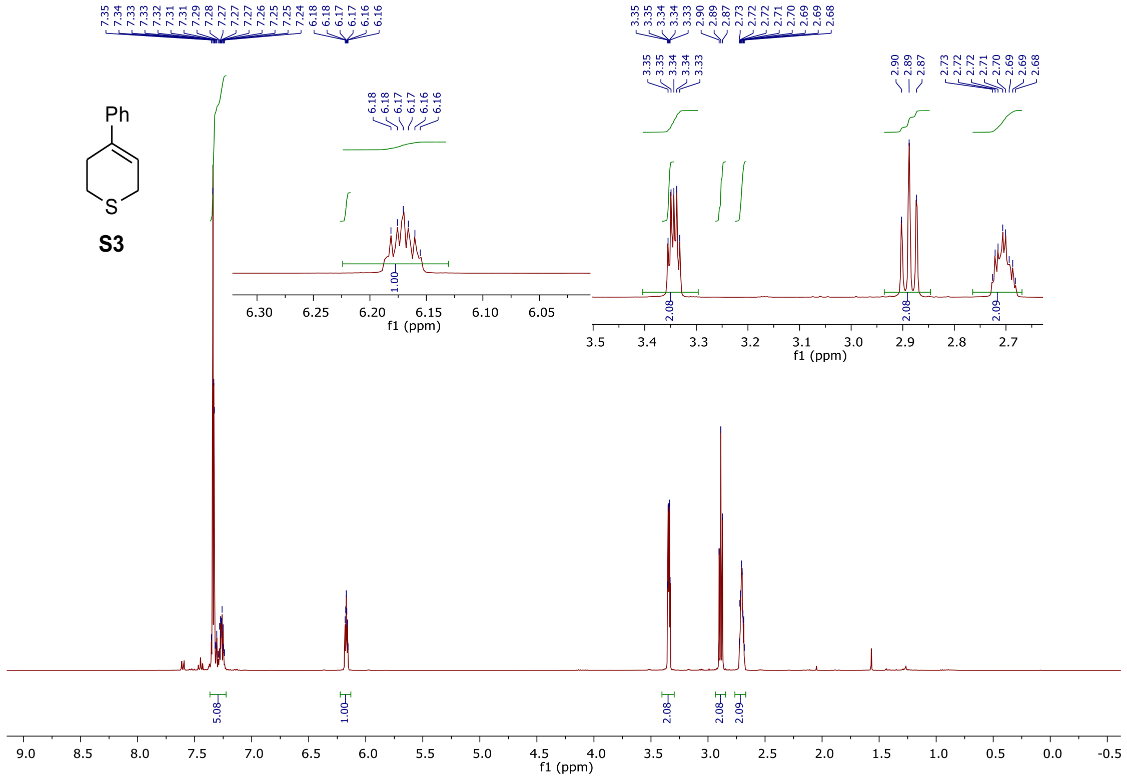

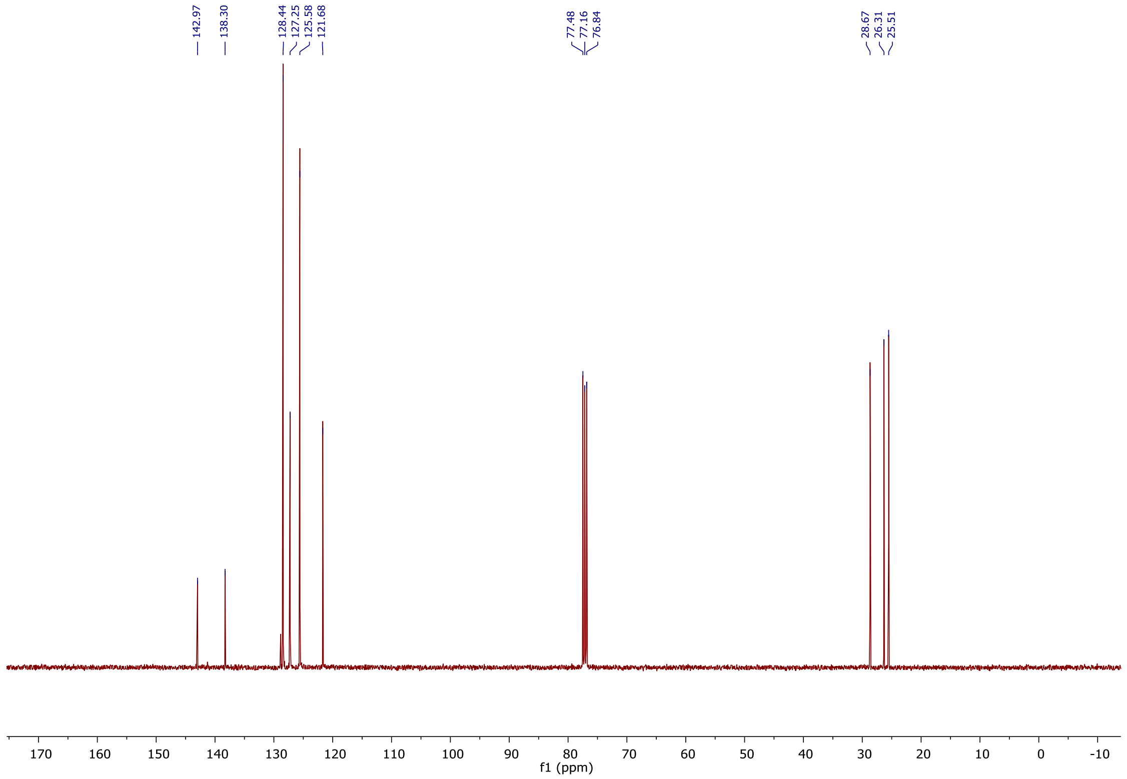

400 MHz ^{1}H NMR spectrum; 100.6 MHz ^{13}C NMR spectrum; $CDCl_3$ of **10a**

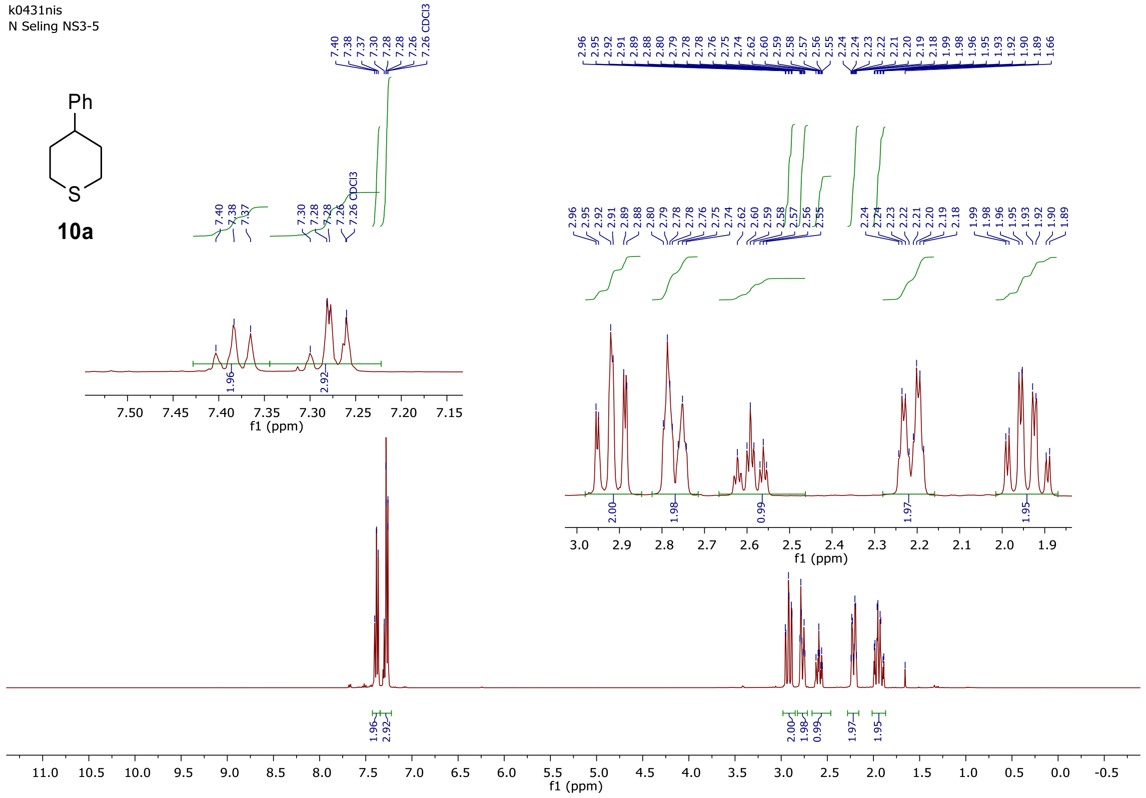

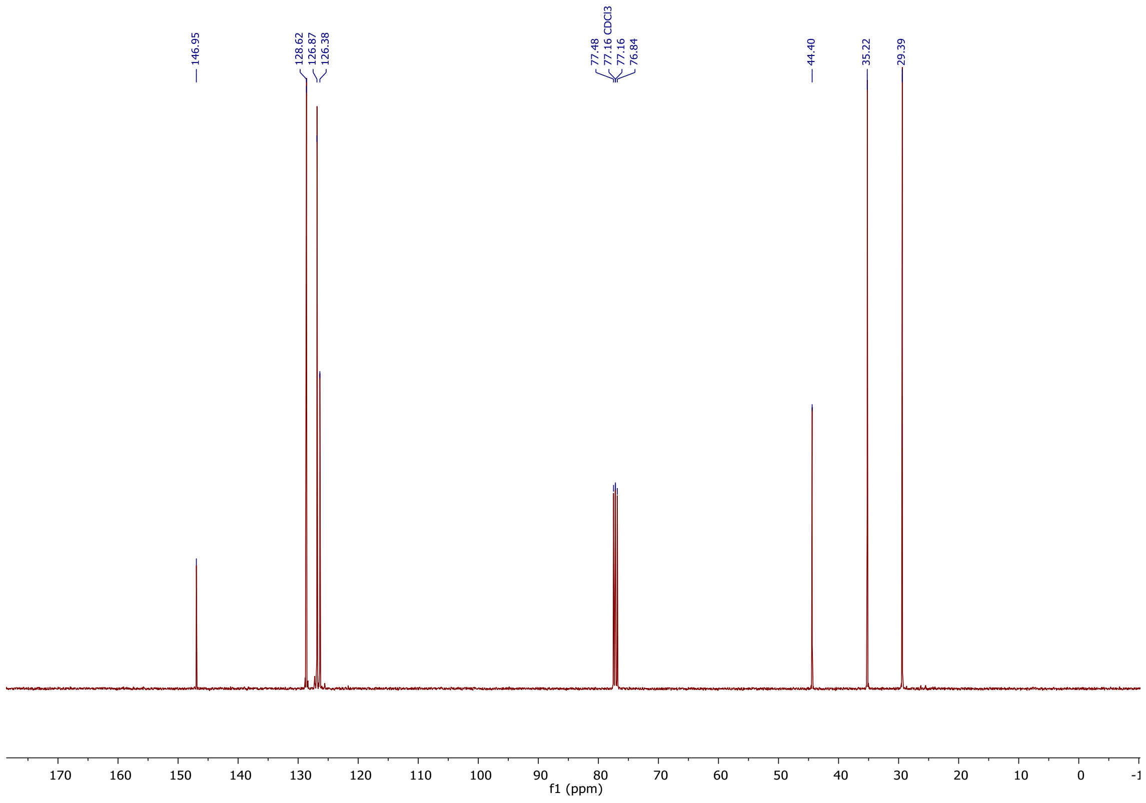

400 MHz ^1H NMR spectrum; 100.6 MHz ^{13}C NMR spectrum; $CDCl_3$ of **S4**

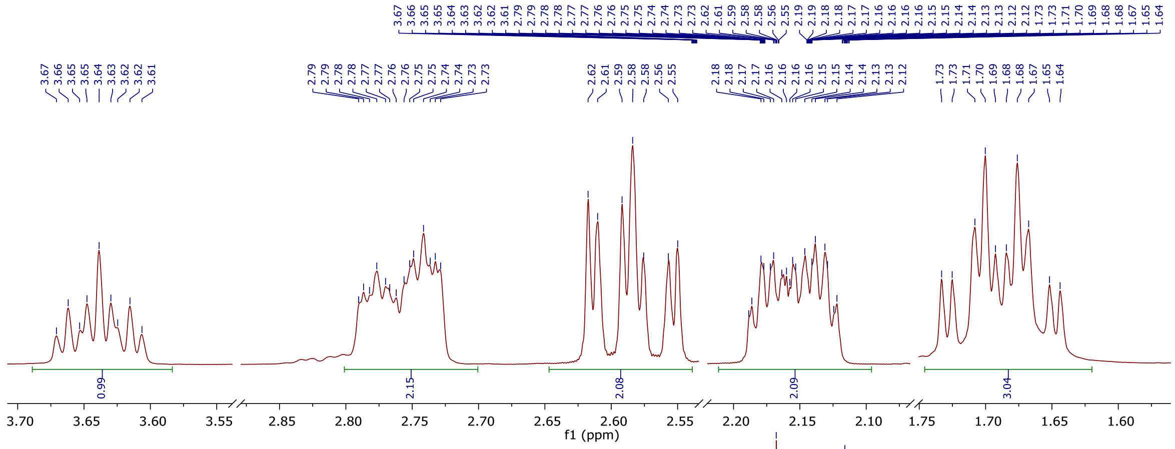

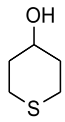

S4

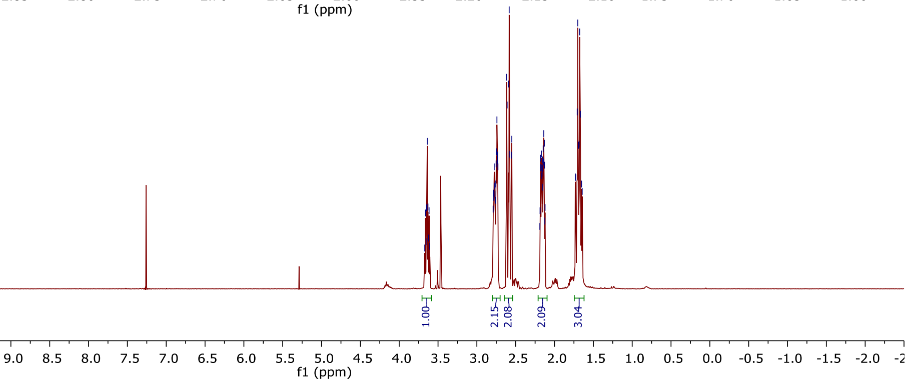

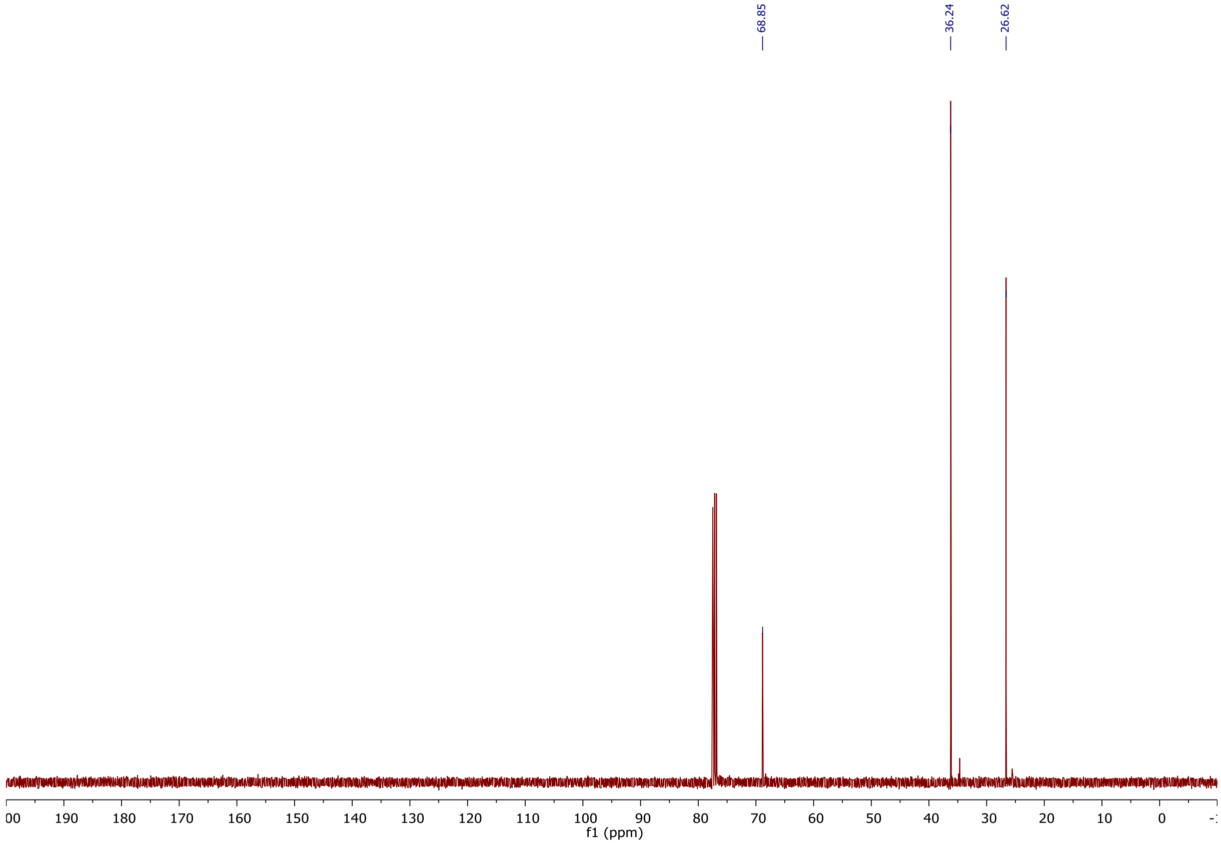

400 MHz ^{1}H NMR spectrum; 100.6 MHz ^{13}C NMR spectrum; $CDCl_3$ of **10b**

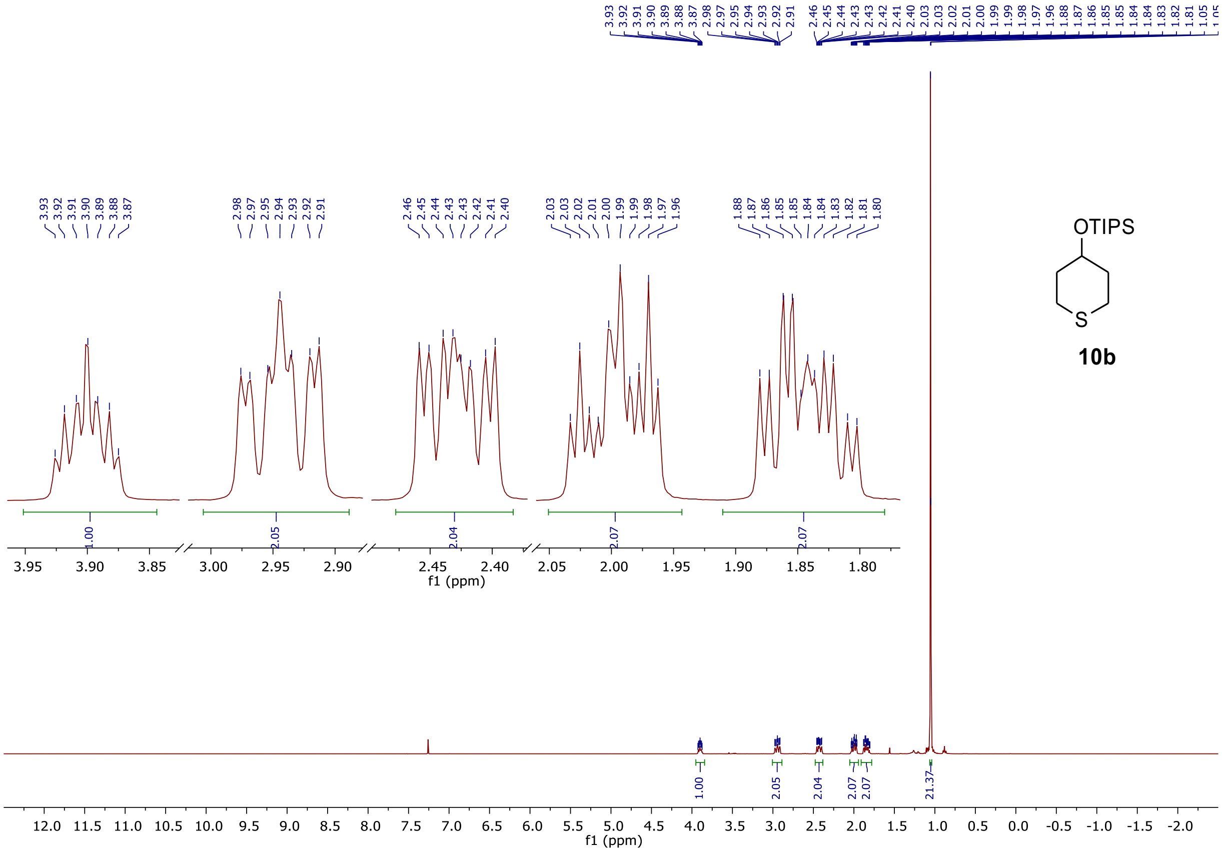

10b

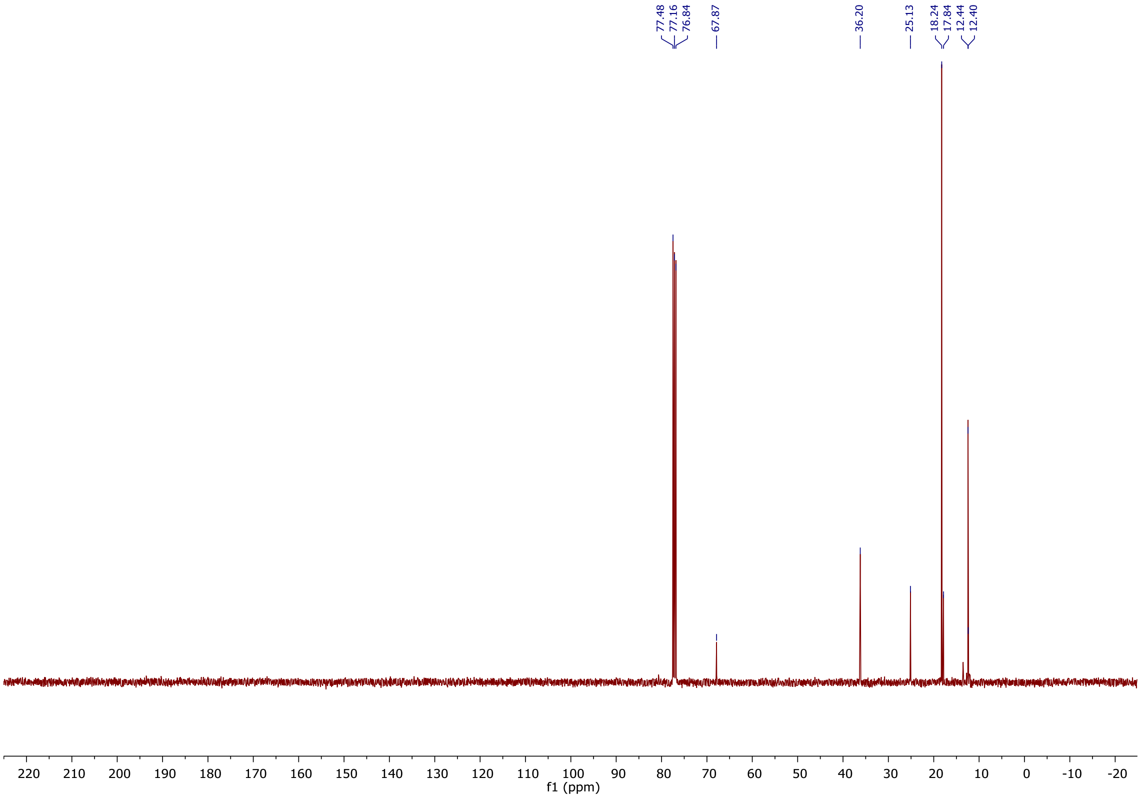

400 MHz ^{1}H NMR spectrum; 100.6 MHz ^{13}C NMR spectrum; $CDCl_3$ of **11a**

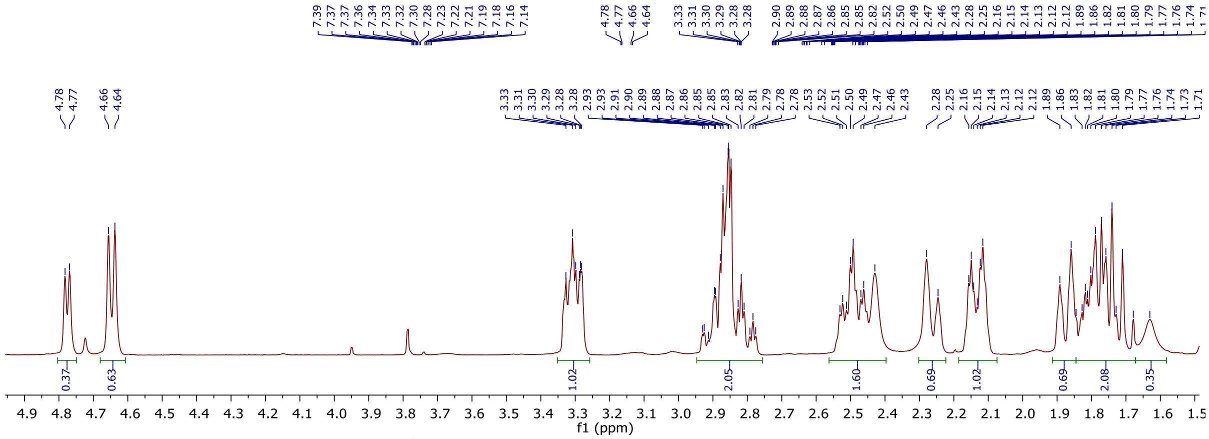

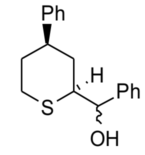

11a

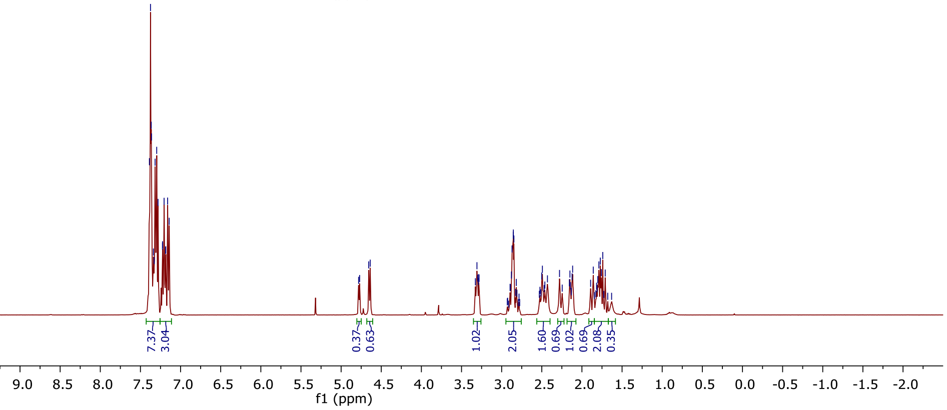

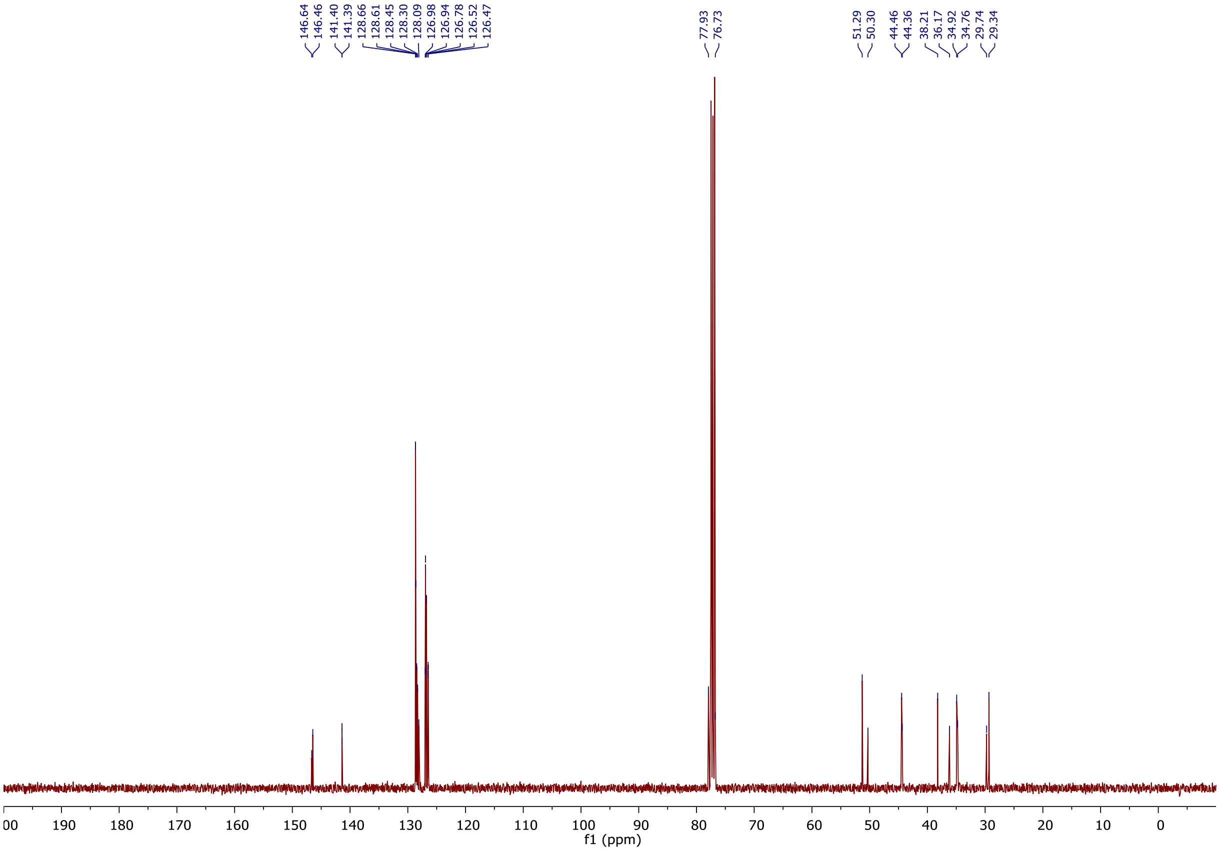

400 MHz ^{1}H NMR spectrum; 100.6 MHz ^{13}C NMR spectrum; $CDCl_3$ of **11b**

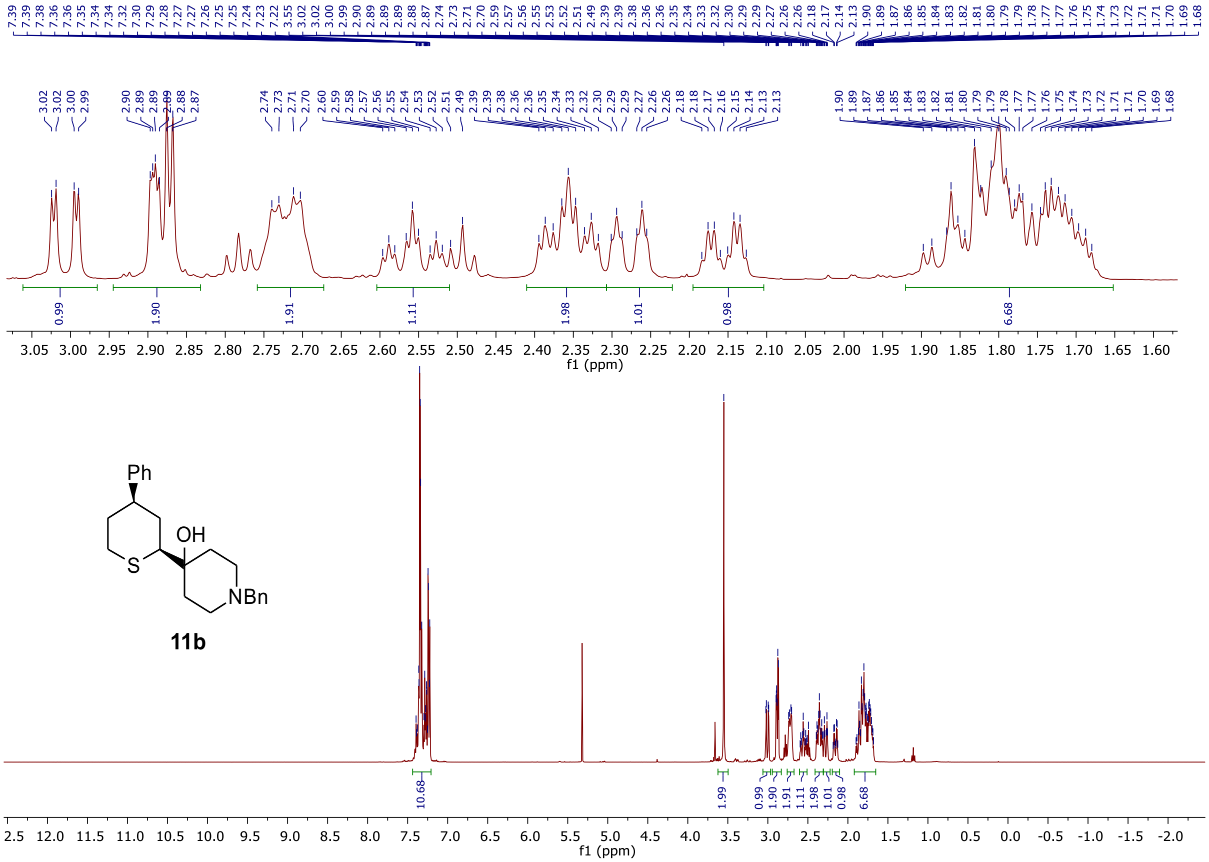

11b

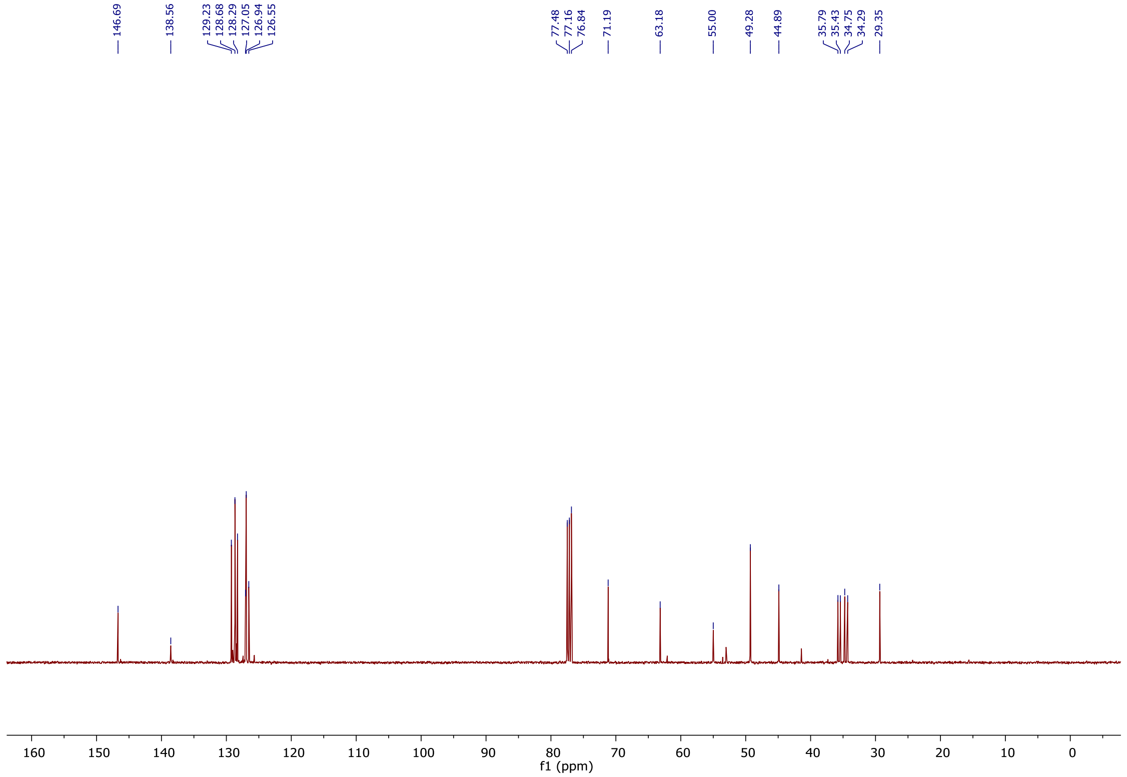

400 MHz ^{1}H NMR spectrum; 100.6 MHz ^{13}C NMR spectrum; $CDCl_3$ of **11c**

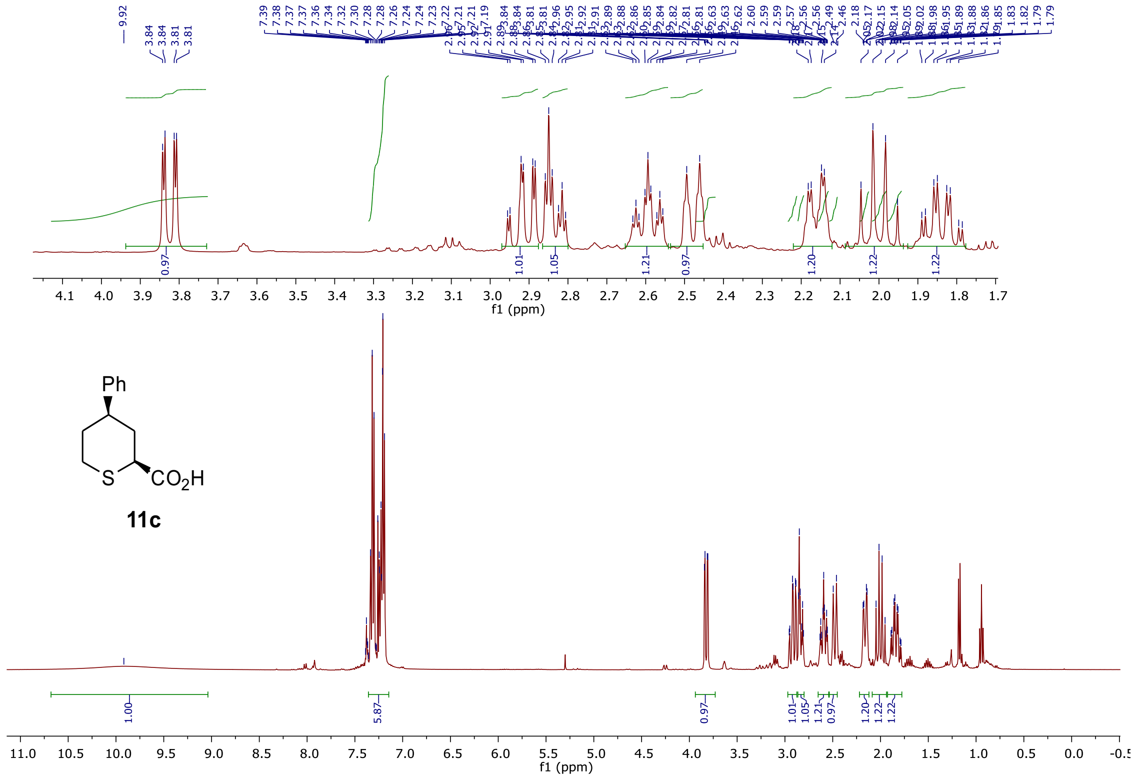

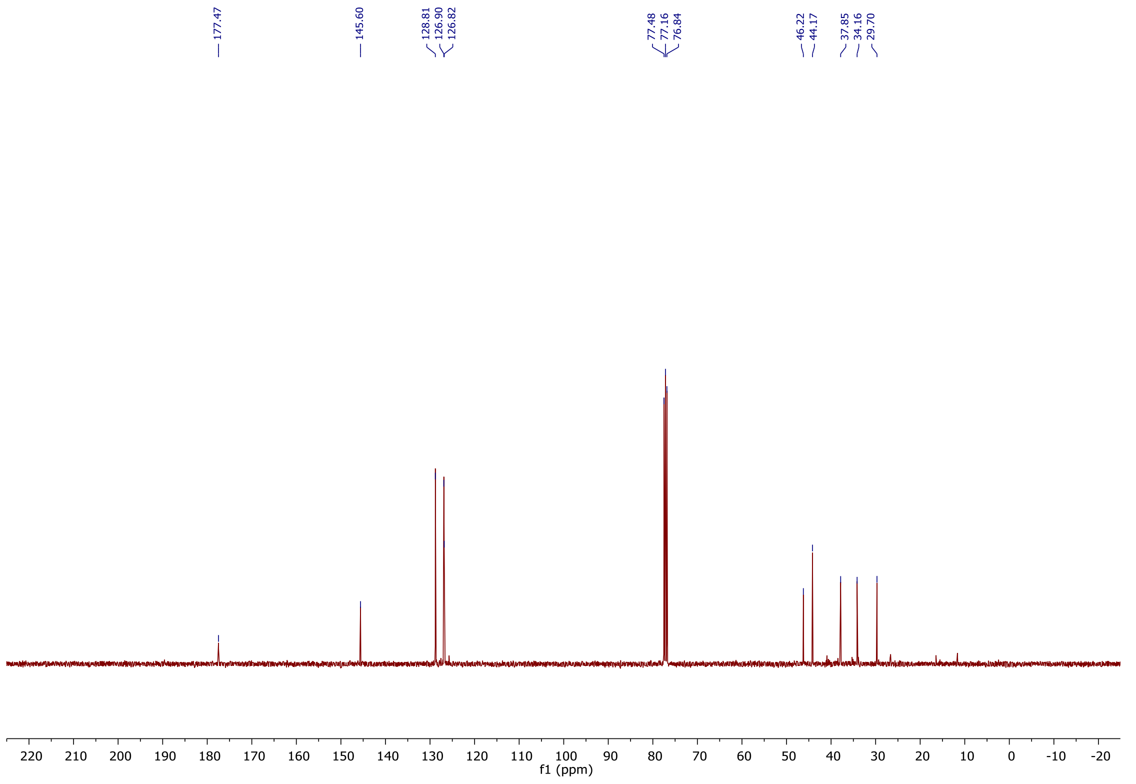

400 MHz ^{1}H NMR spectrum; 100.6 MHz ^{13}C NMR spectrum; $CDCl_3$ of **11d**

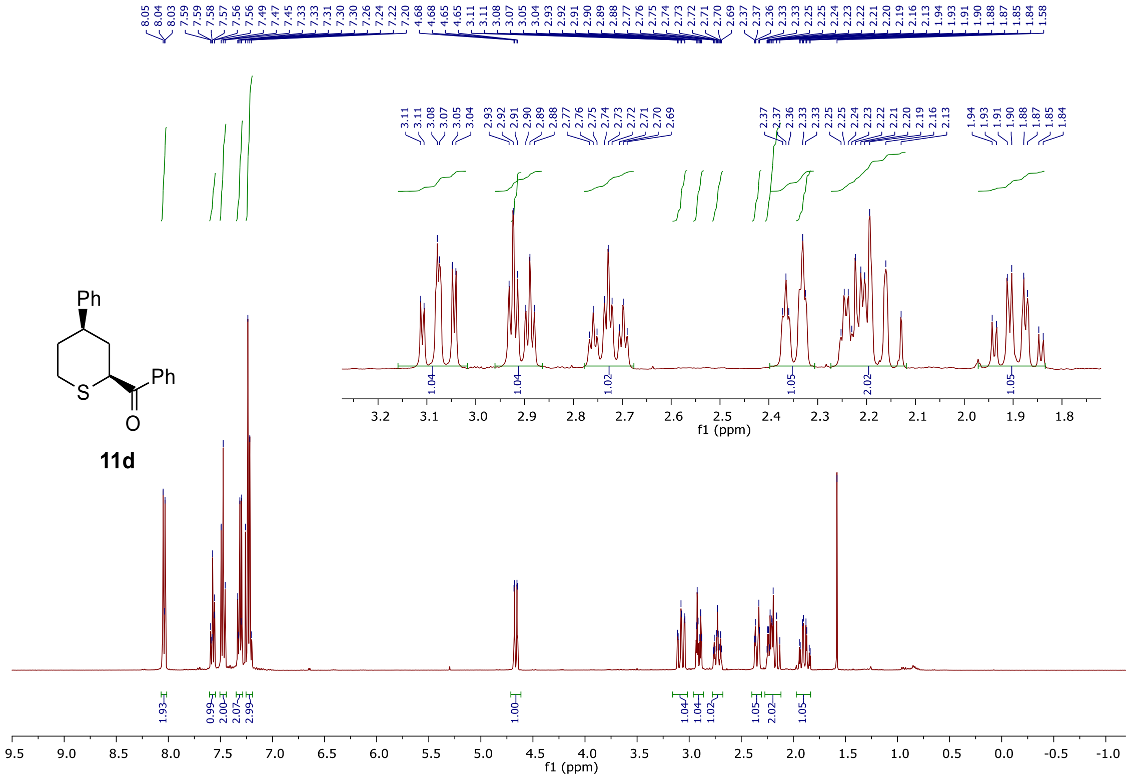

11d

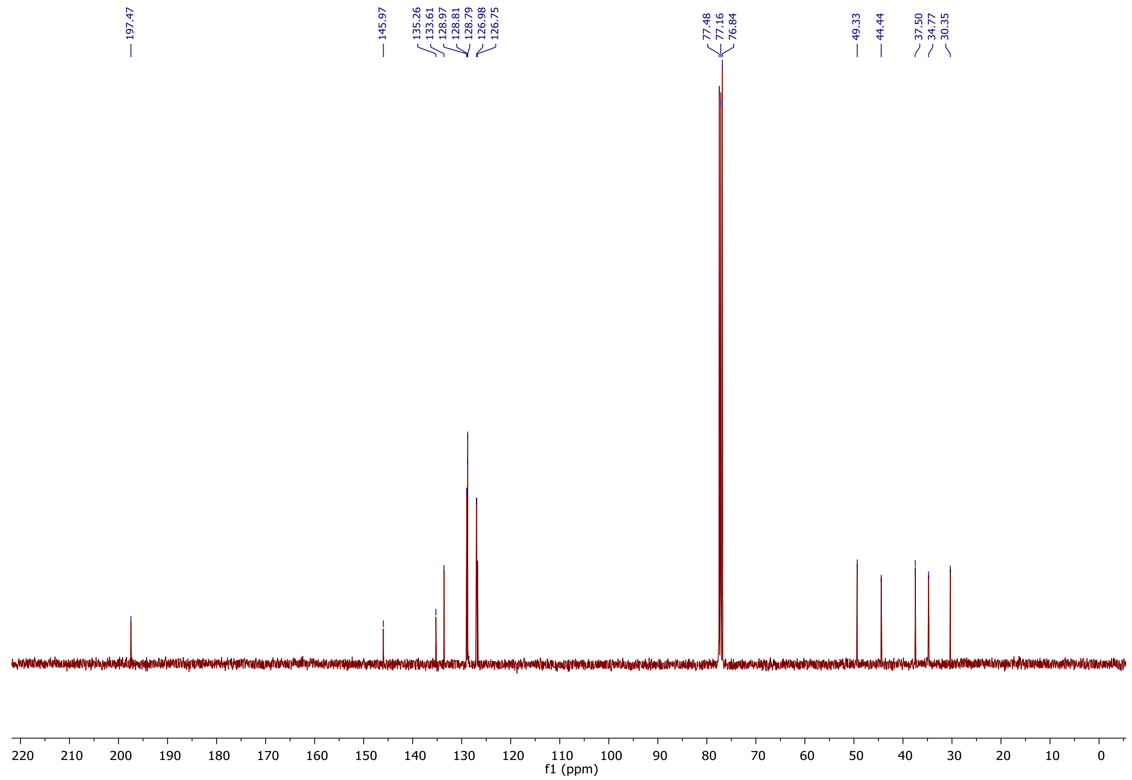

400 MHz ^{1}H NMR spectrum; 100.6 MHz ^{13}C NMR spectrum; $CDCl_3$ of **11e**

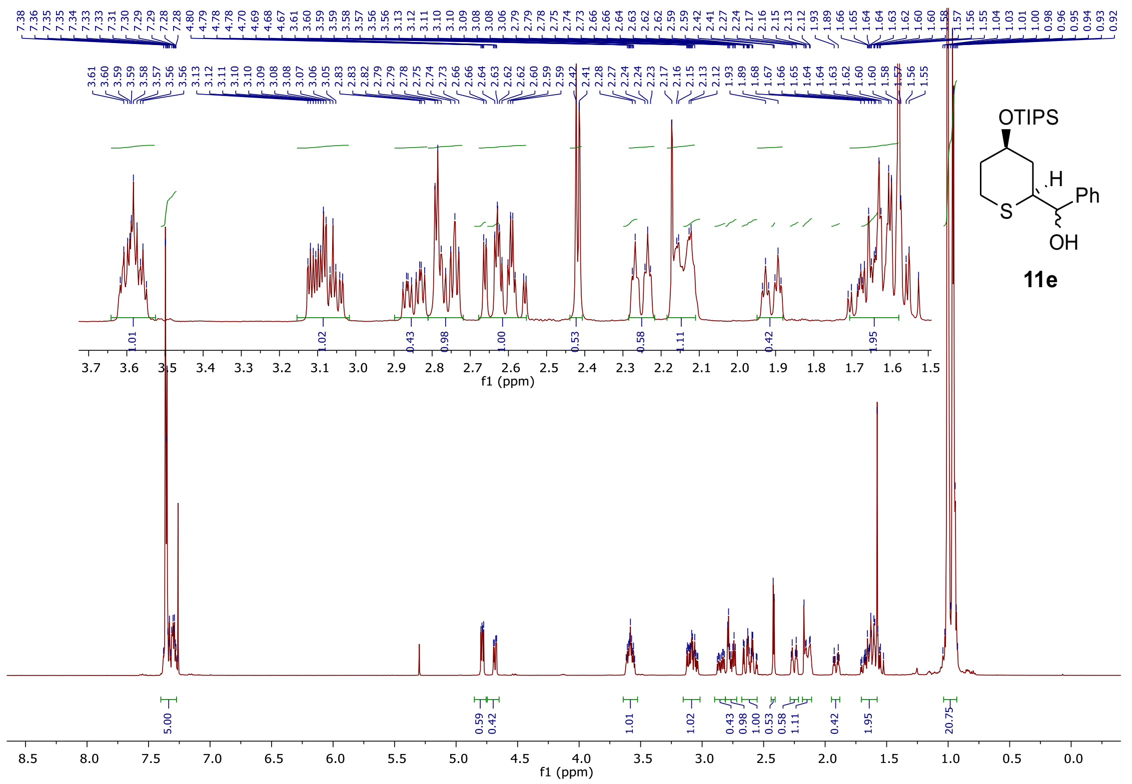

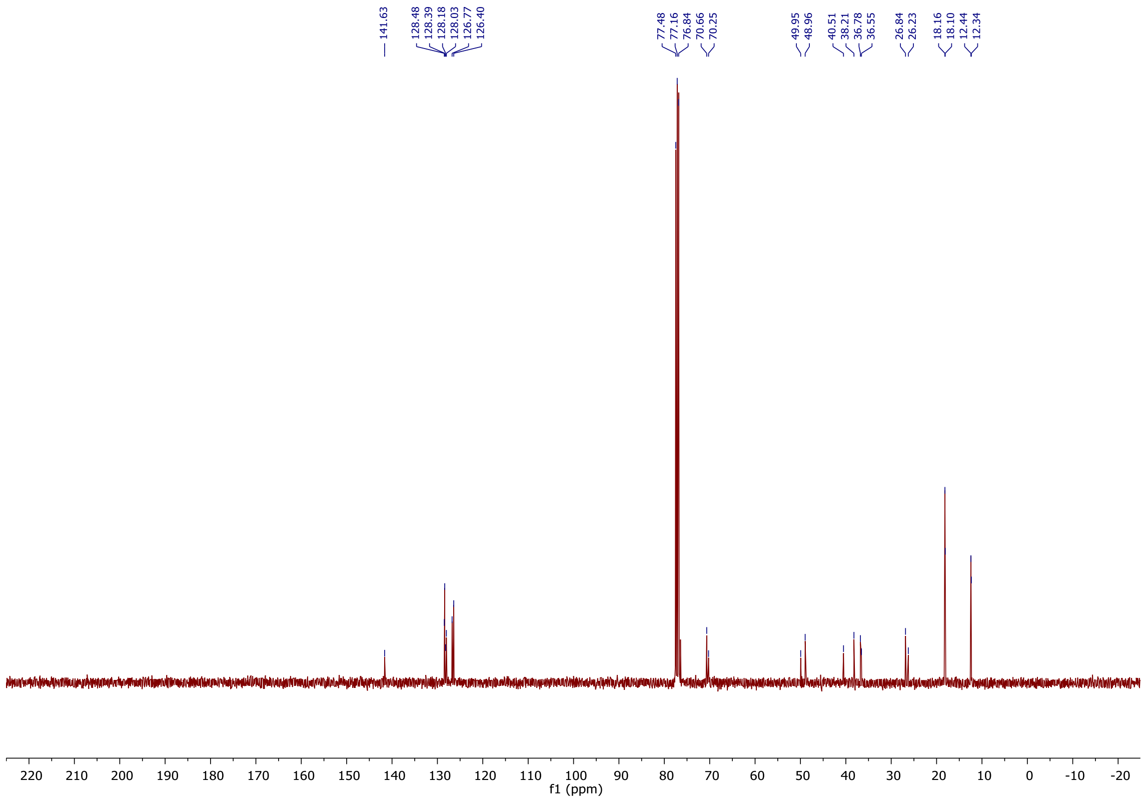

400 MHz ^{1}H NMR spectrum; 100.6 MHz ^{13}C NMR spectrum; $CDCl_3$ of **11f**

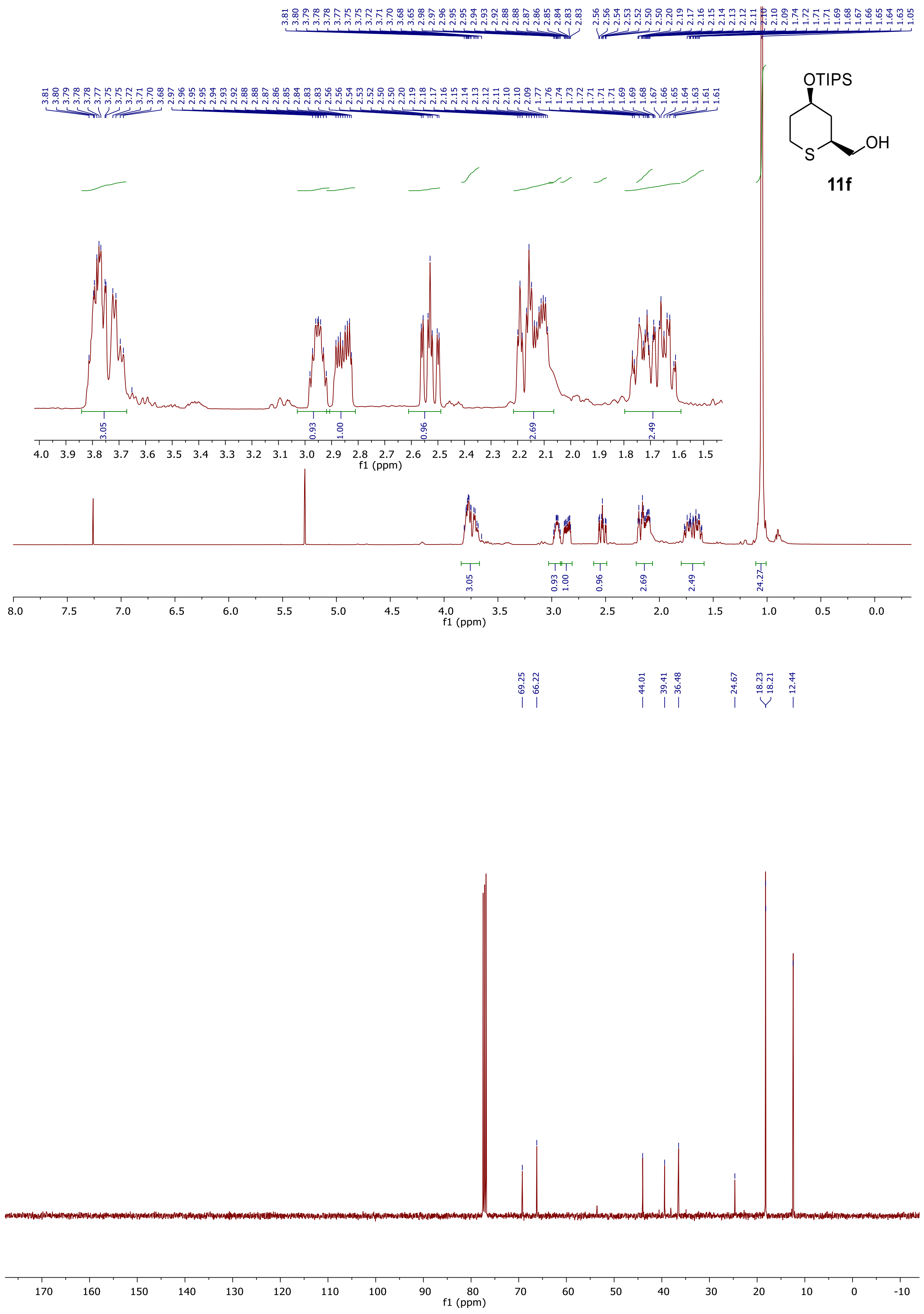

400 MHz ^{1}H NMR spectrum; 100.6 MHz ^{13}C NMR spectrum; $CDCl_3$ of **11g**

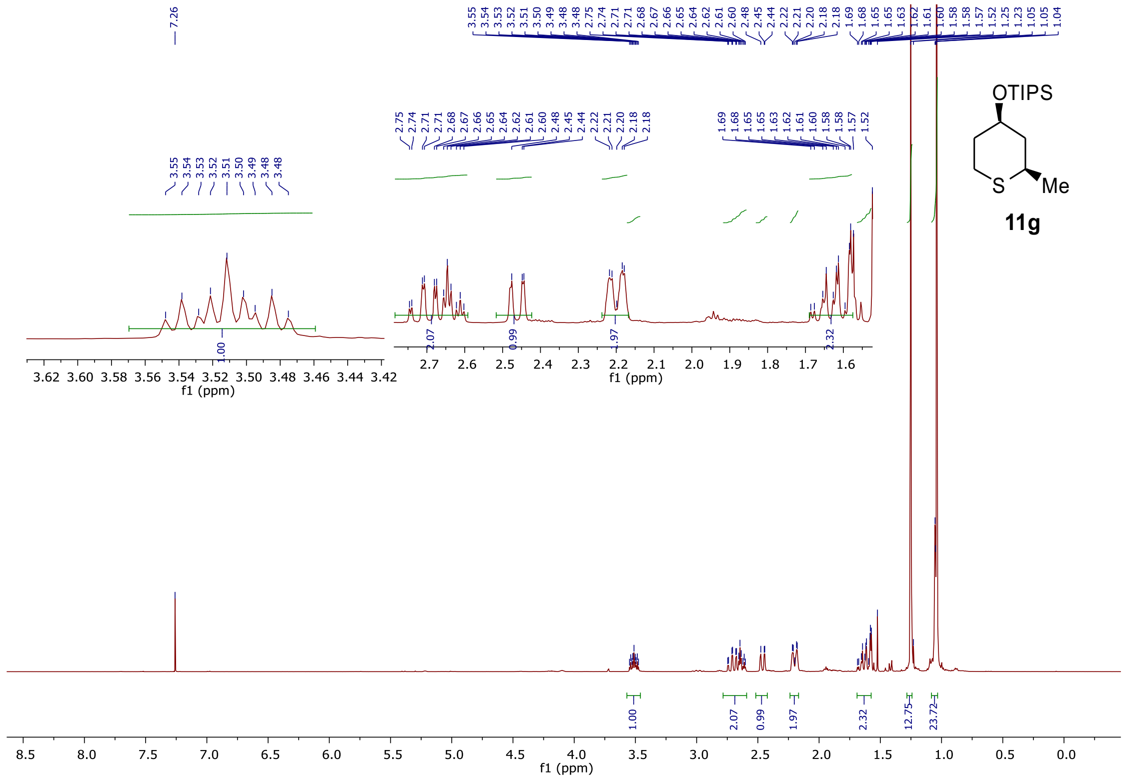

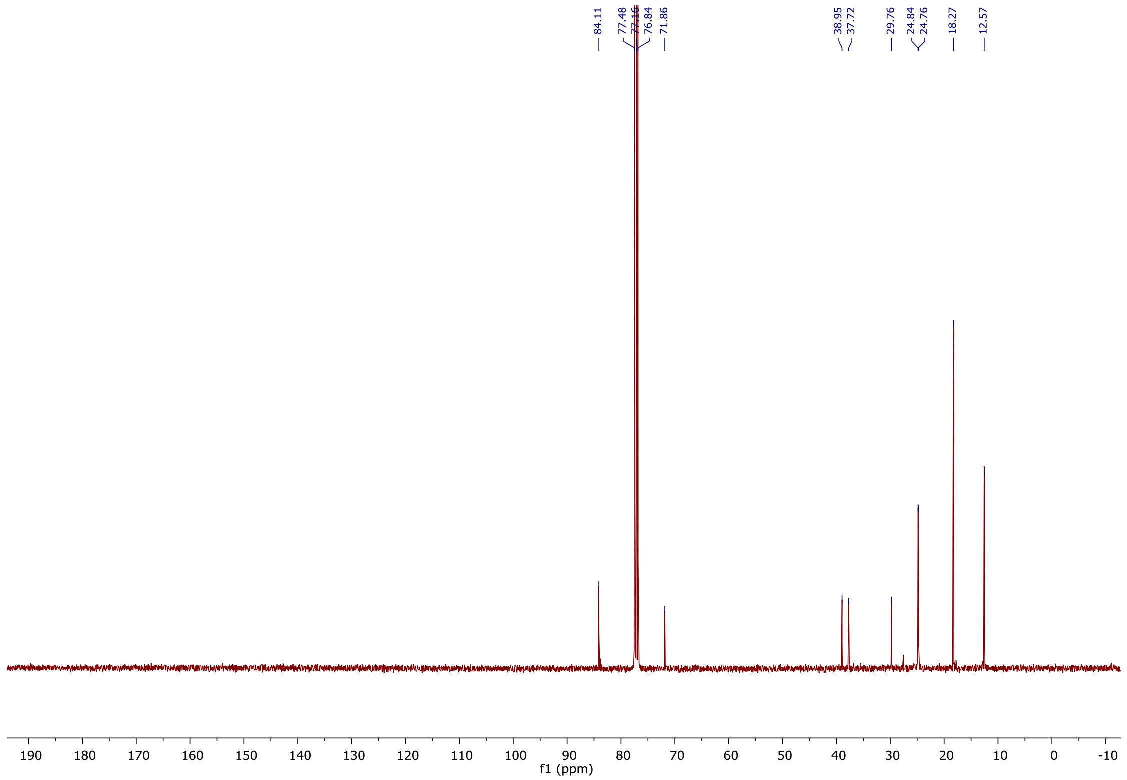

400 MHz ^{1}H NMR spectrum; 100.6 MHz ^{13}C NMR spectrum; $CDCl_3$ of **11h**

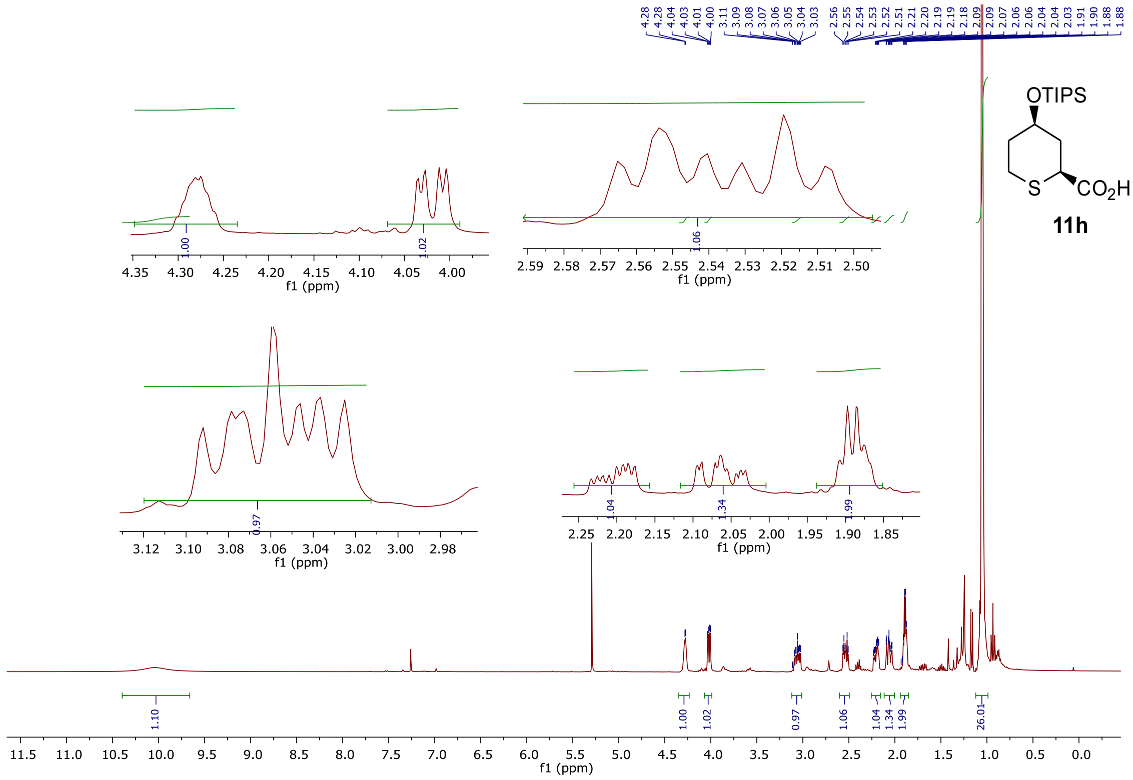

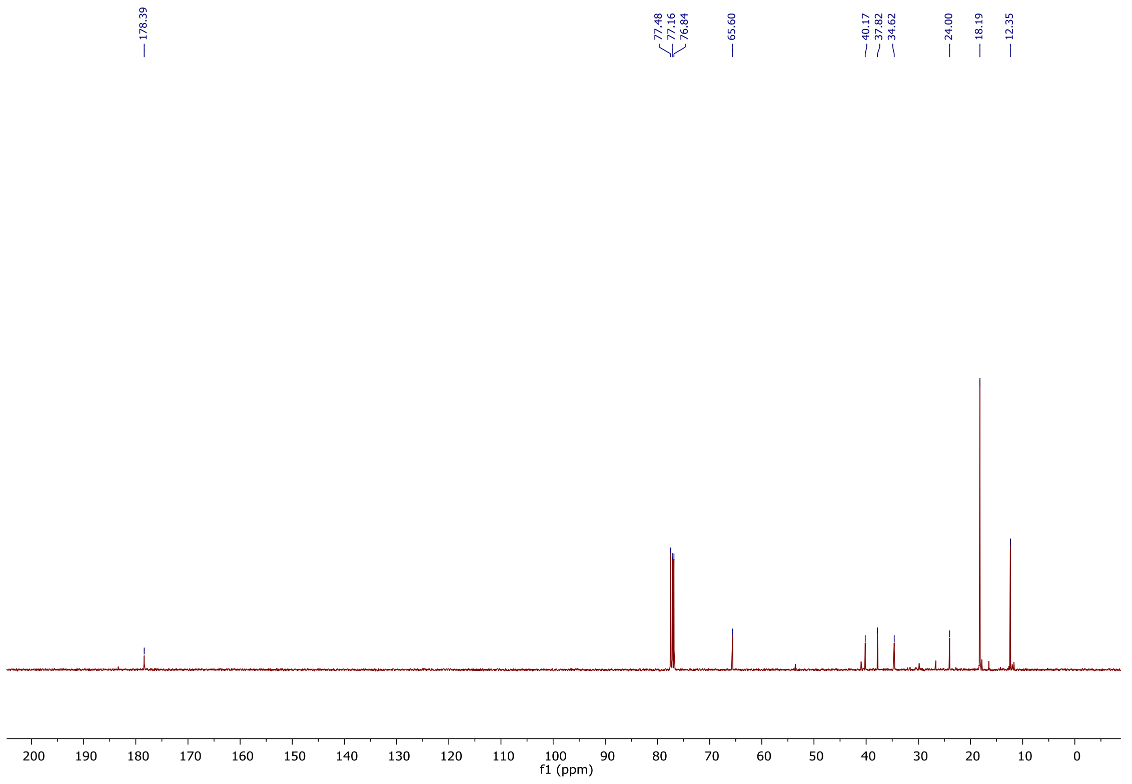

400 MHz ^{1}H NMR spectrum; 100.6 MHz ^{13}C NMR spectrum; $CDCl_3$ of **11i**

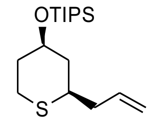

11i

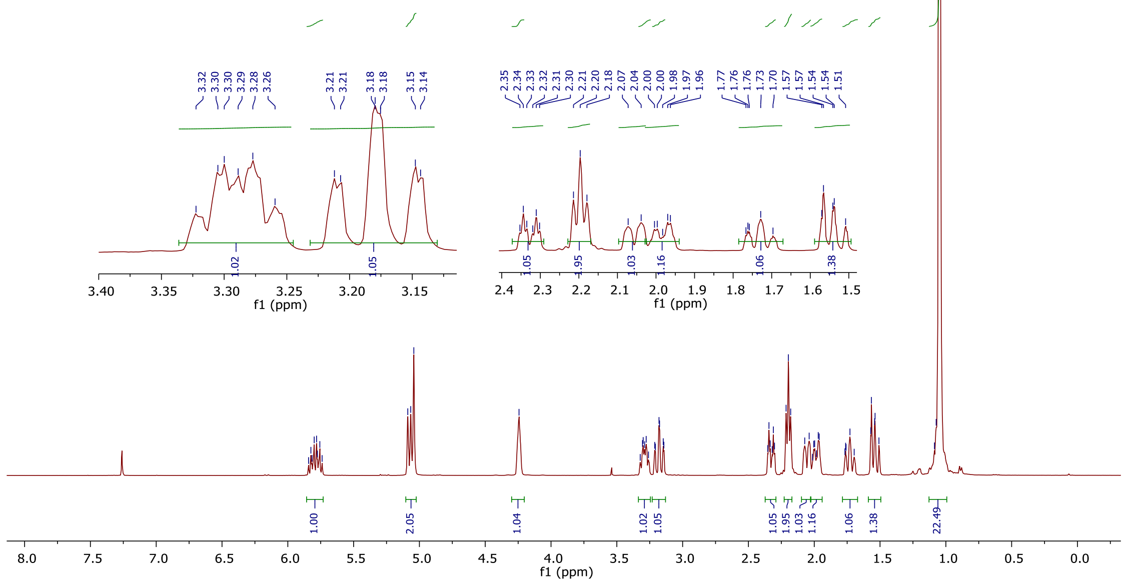

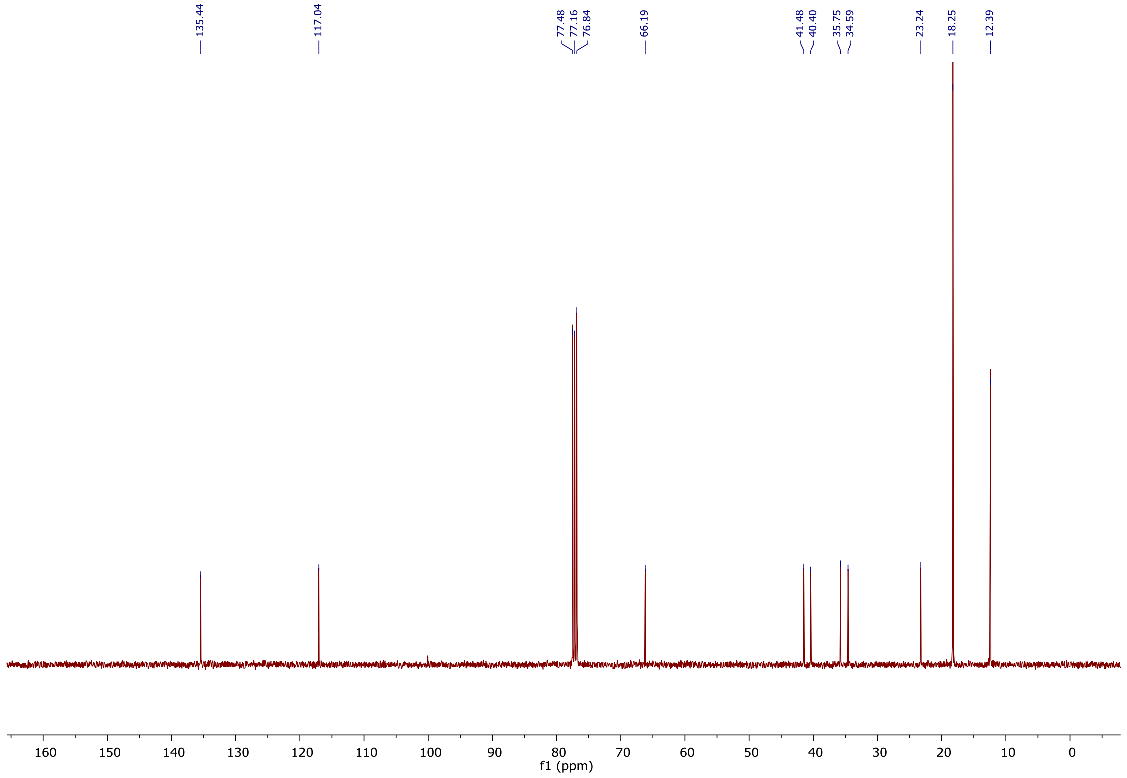

400 MHz ^{1}H NMR spectrum; 100.6 MHz ^{13}C NMR spectrum; $CDCl_3$ of **11j**

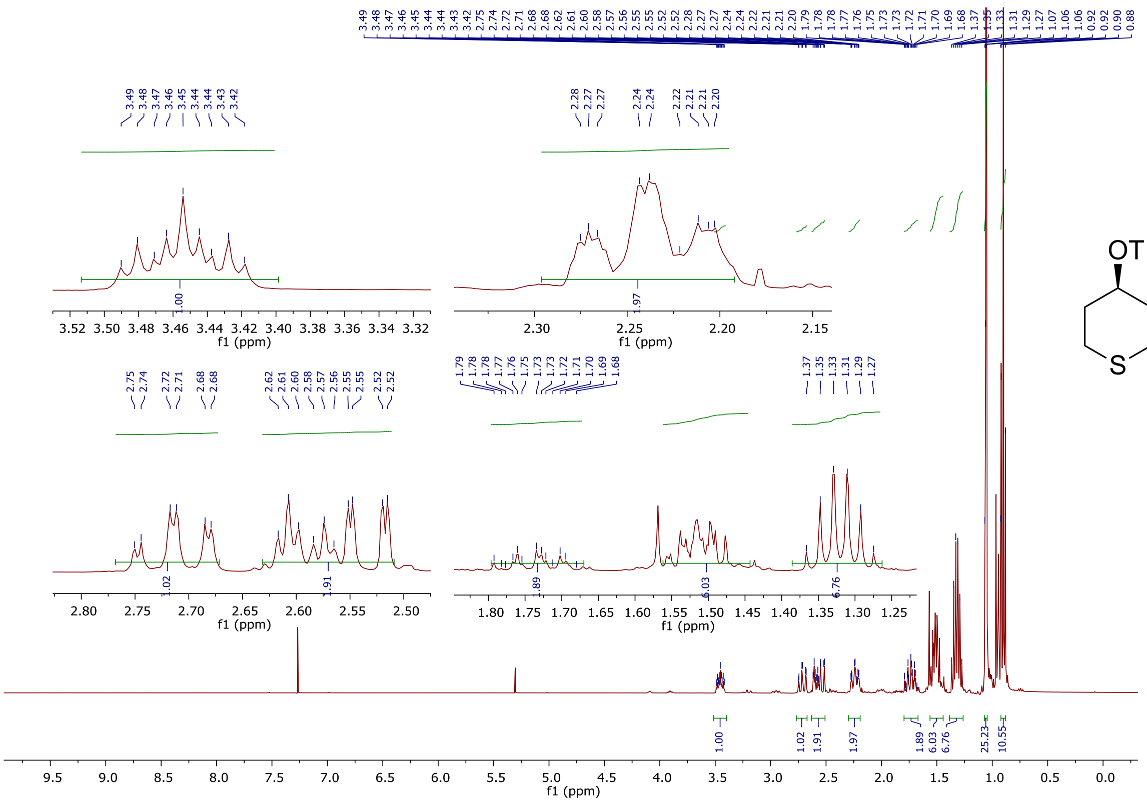

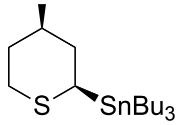

11j

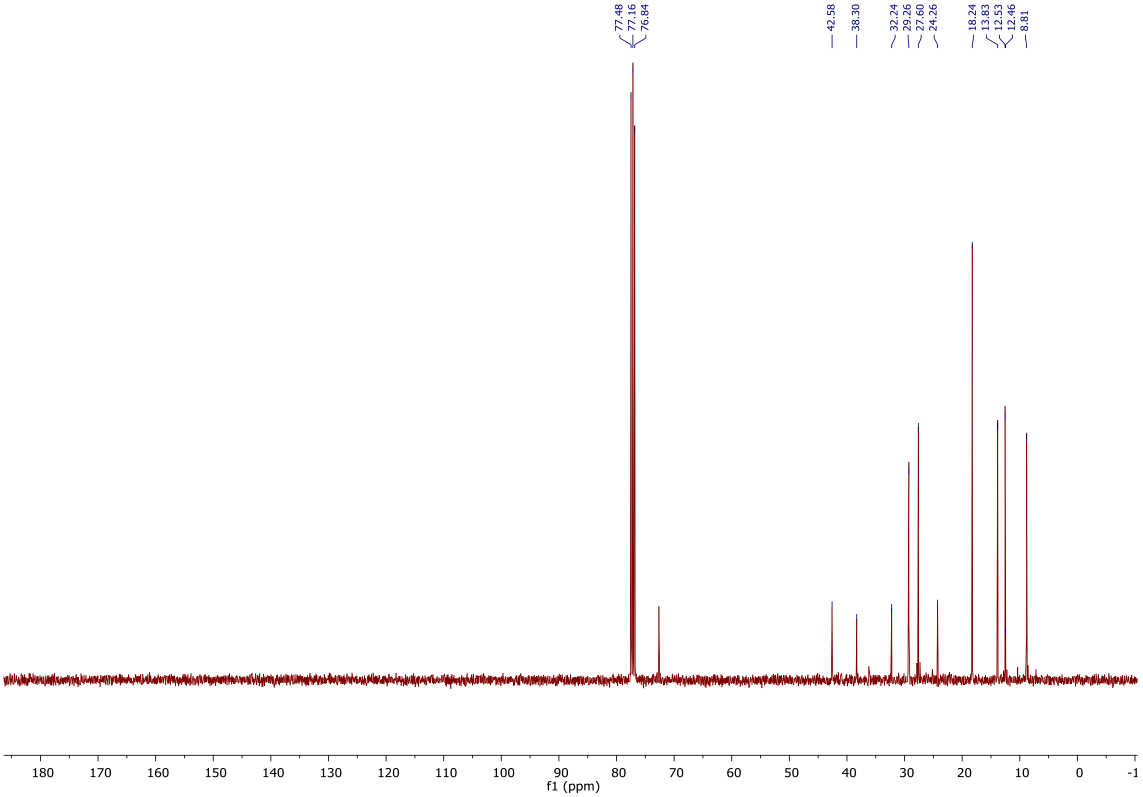

400 MHz ^{1}H NMR spectrum; 100.6 MHz ^{13}C NMR spectrum; $CDCl_3$ of **12**

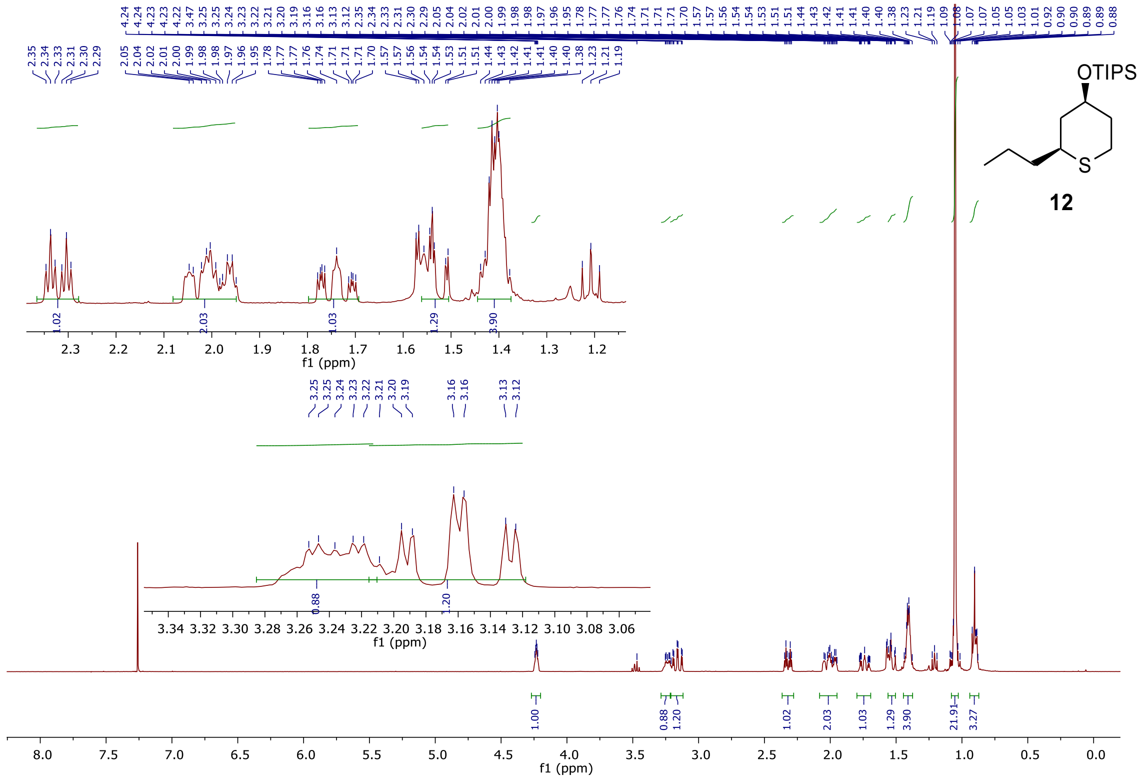

12

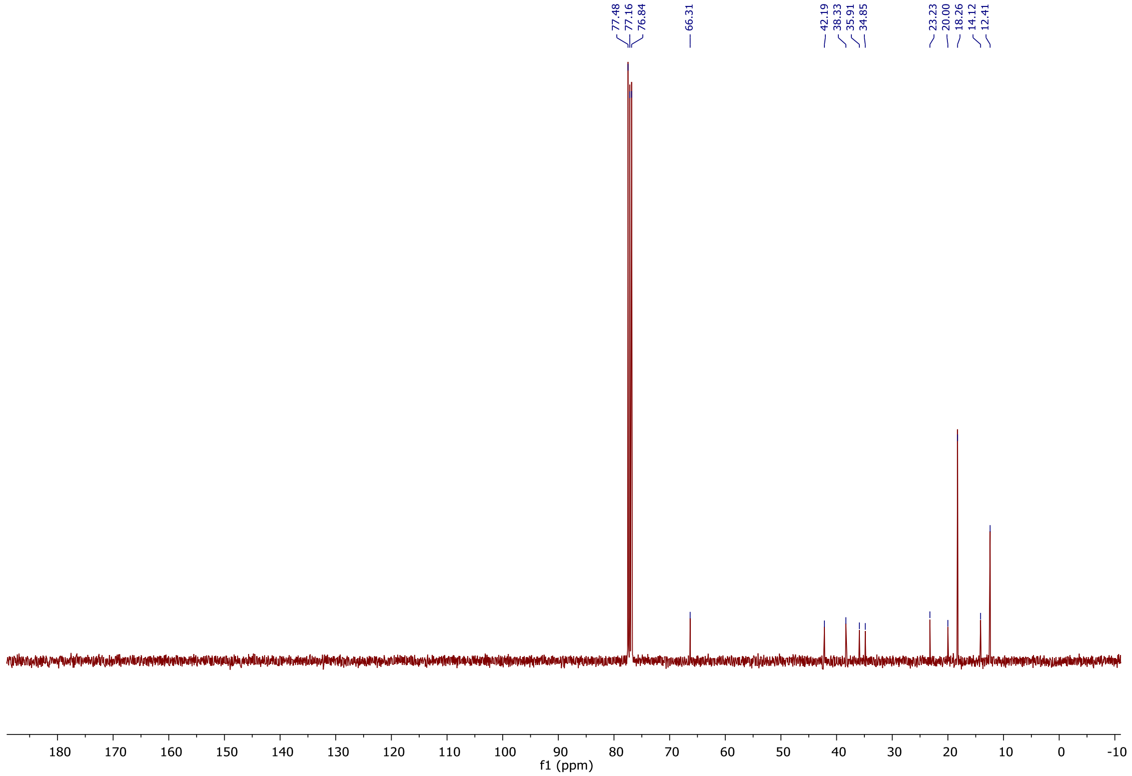

400 MHz ^{1}H NMR spectrum; 100.6 MHz ^{13}C NMR spectrum; $CDCl_3$ of **13**

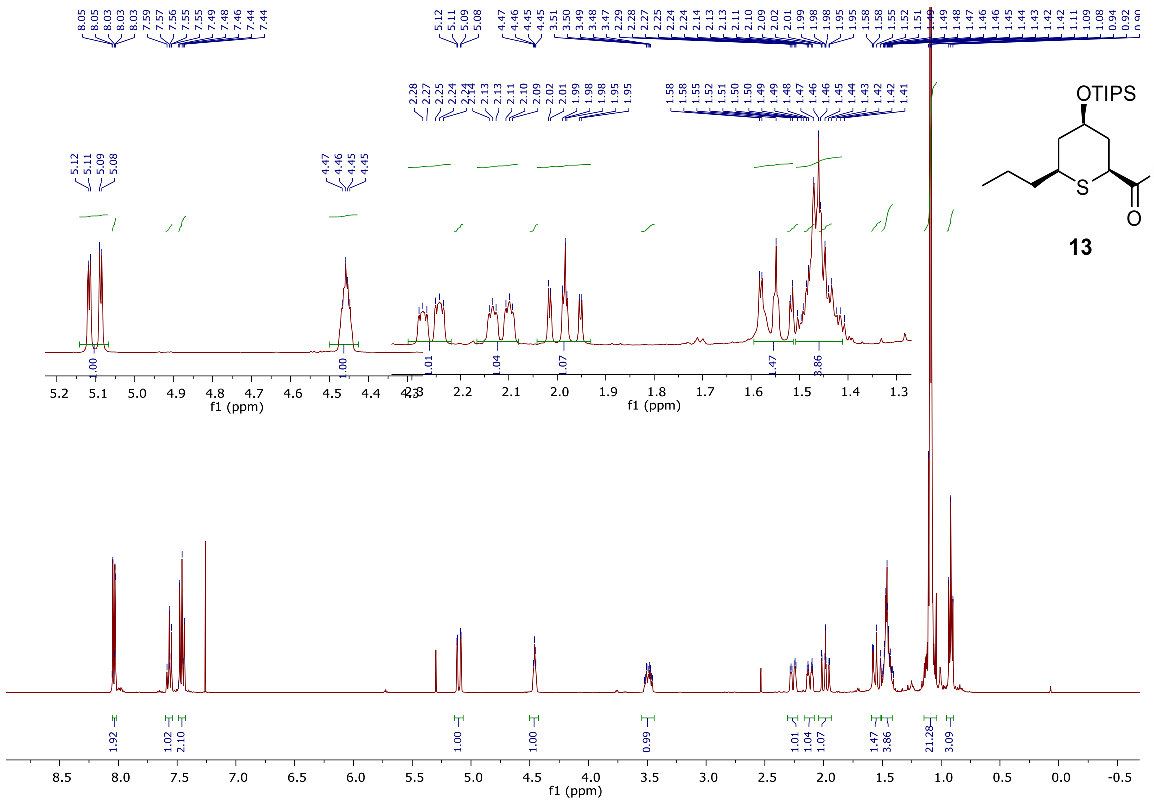

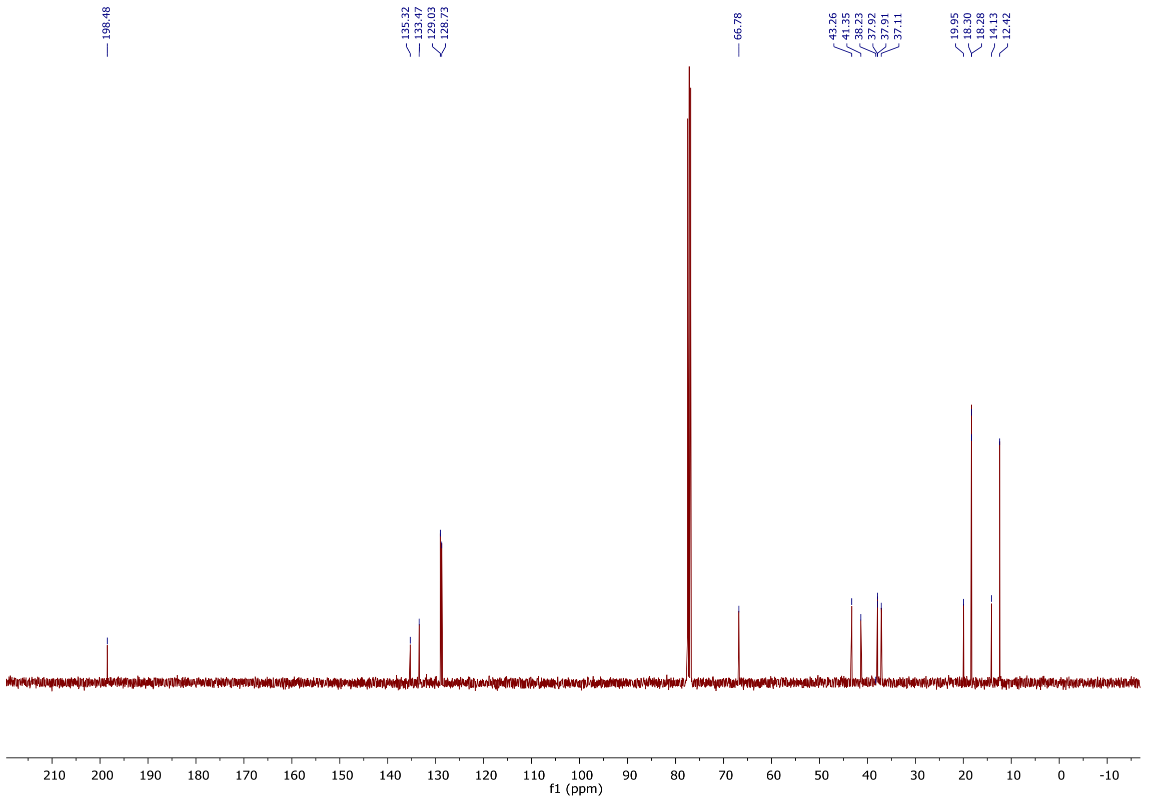

400 MHz ^{1}H NMR spectrum; 100.6 MHz ^{13}C NMR spectrum; $CDCl_3$ of **S5**

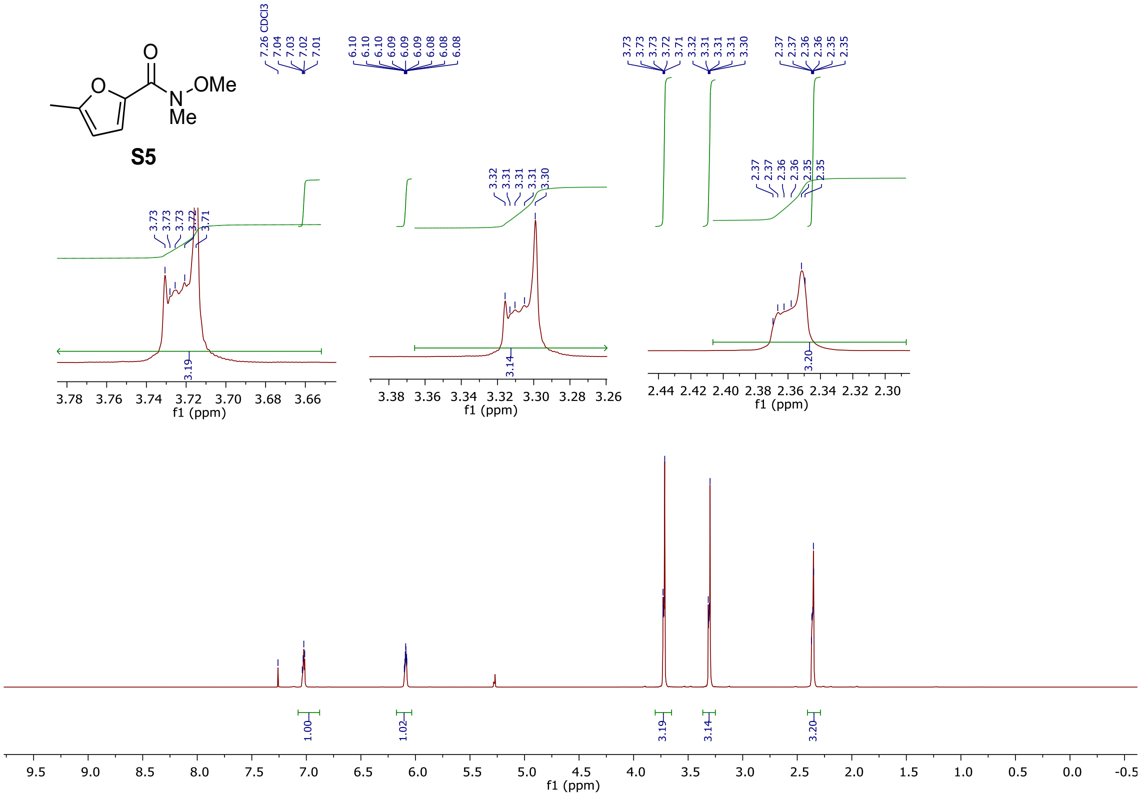

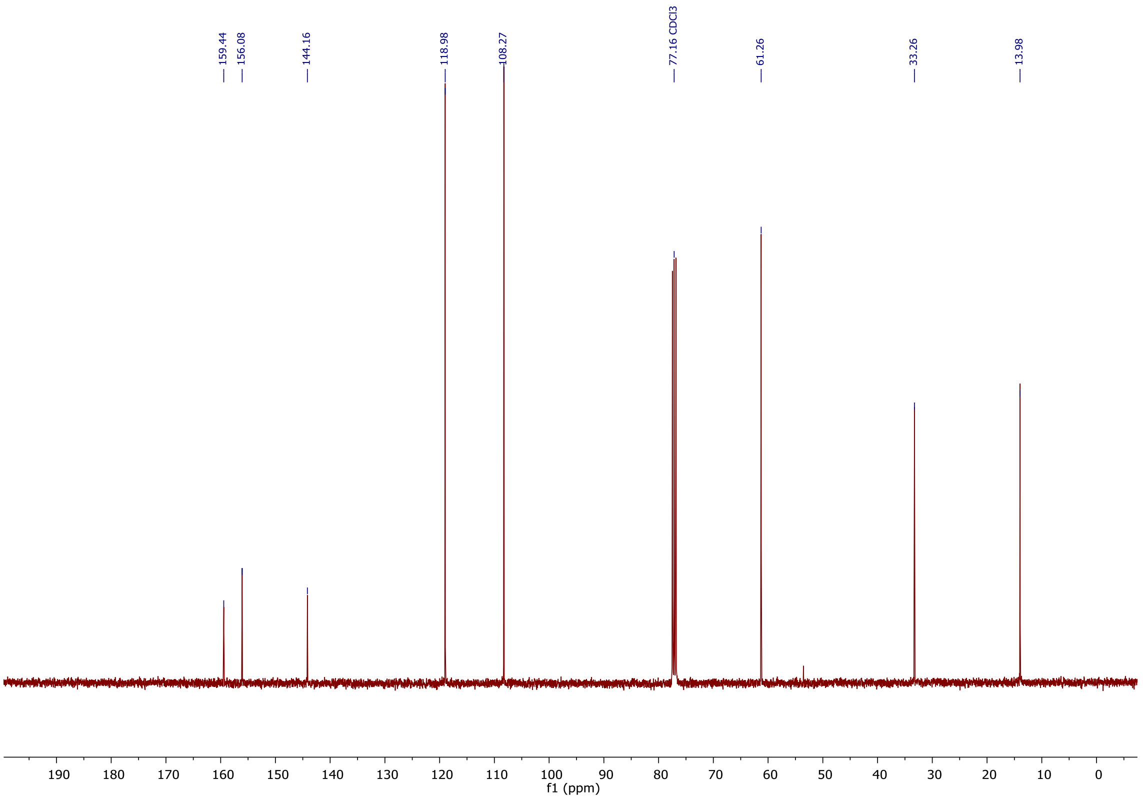

400 MHz ^{1}H NMR spectrum; 100.6 MHz ^{13}C NMR spectrum; $CDCl_3$ of **7p**

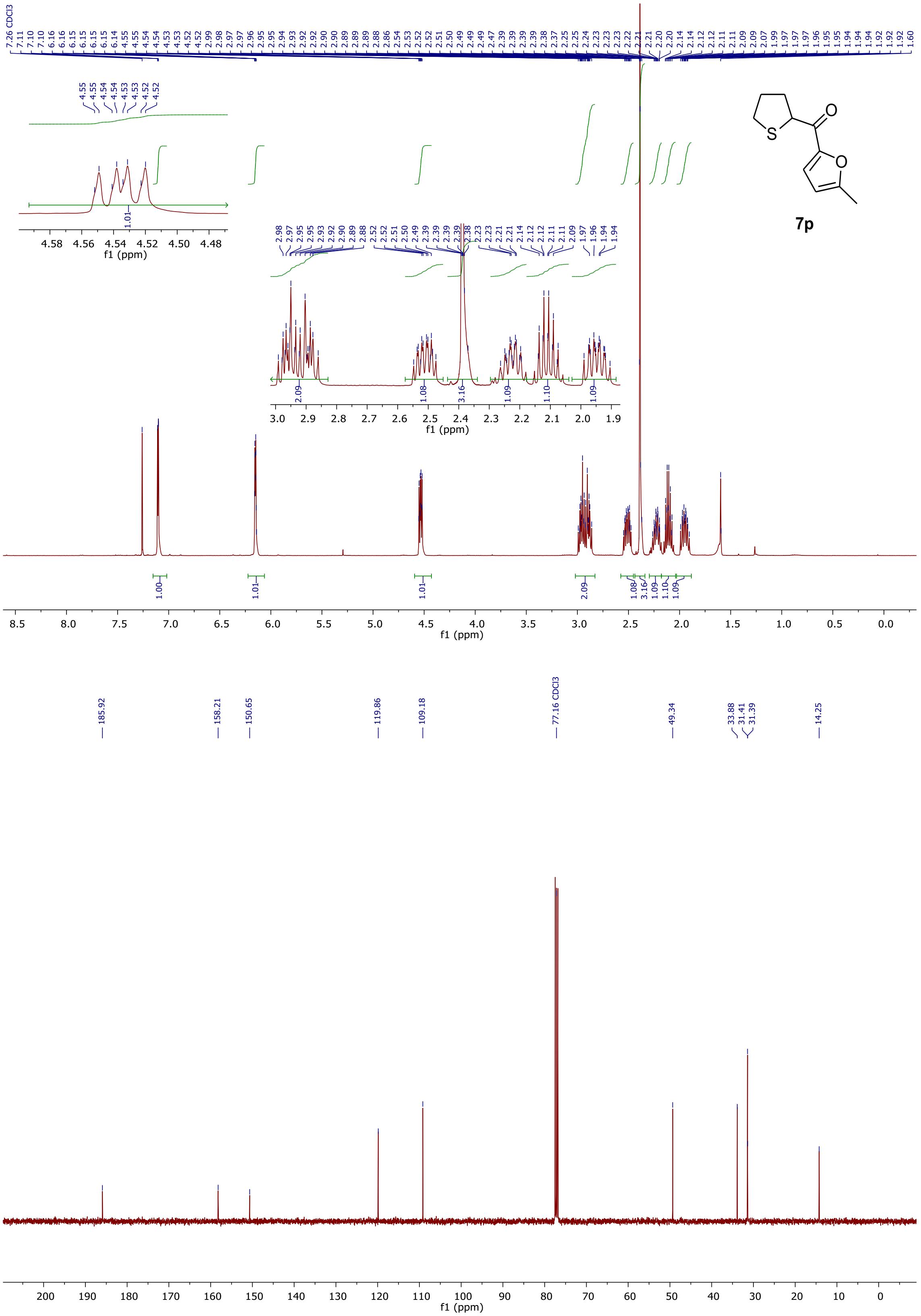

400 MHz ^{1}H NMR spectrum; 100.6 MHz ^{13}C NMR spectrum; $CDCl_3$ of **14a**

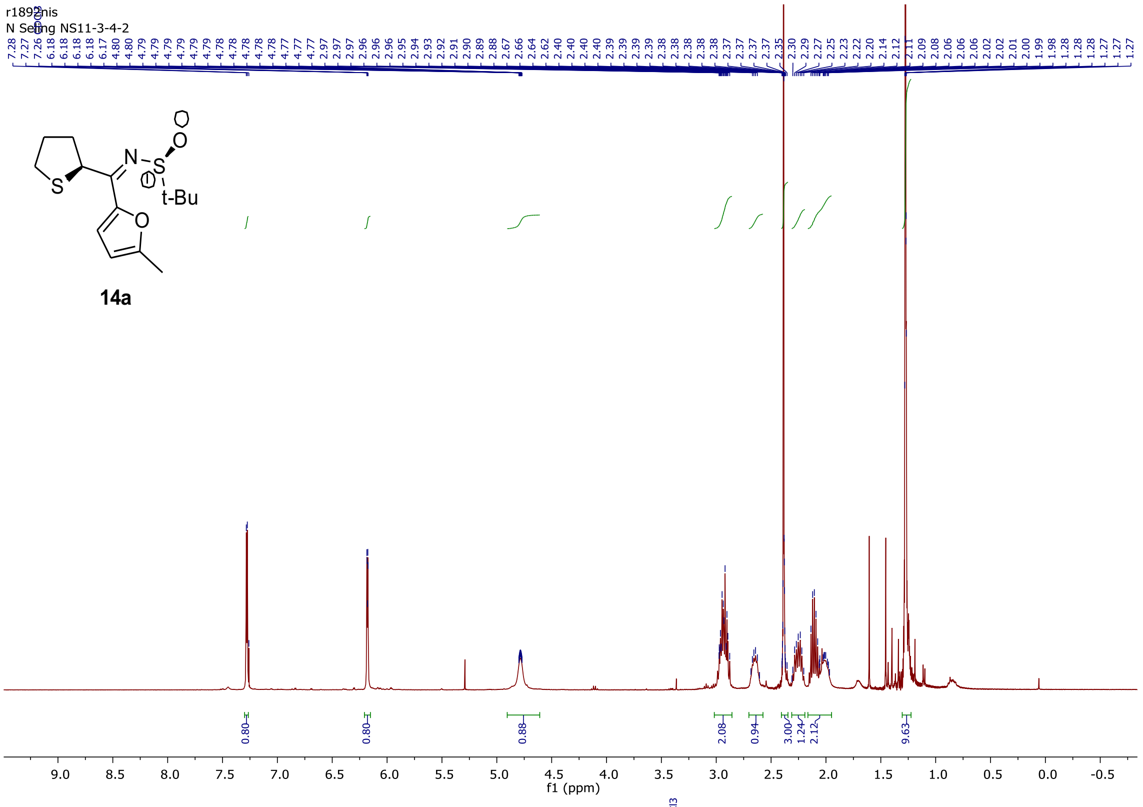

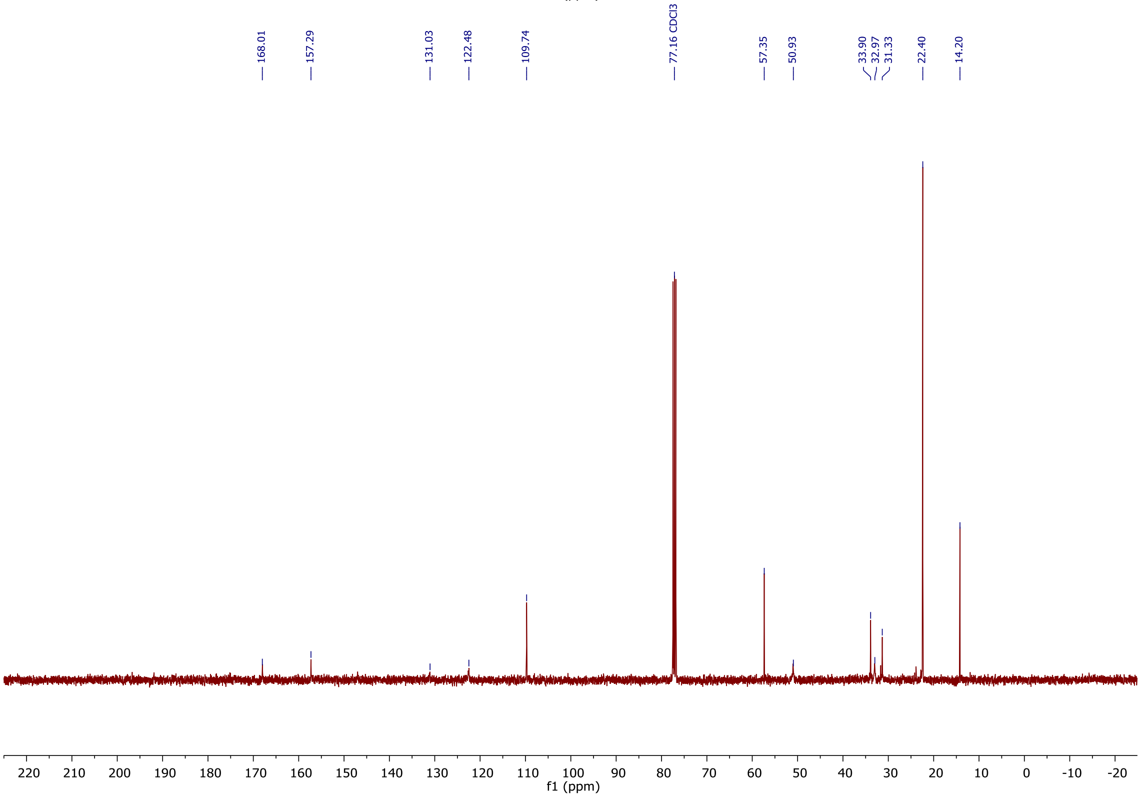

400 MHz ^{1}H NMR spectrum; 100.6 MHz ^{13}C NMR spectrum; $CDCl_3$ of **14b**

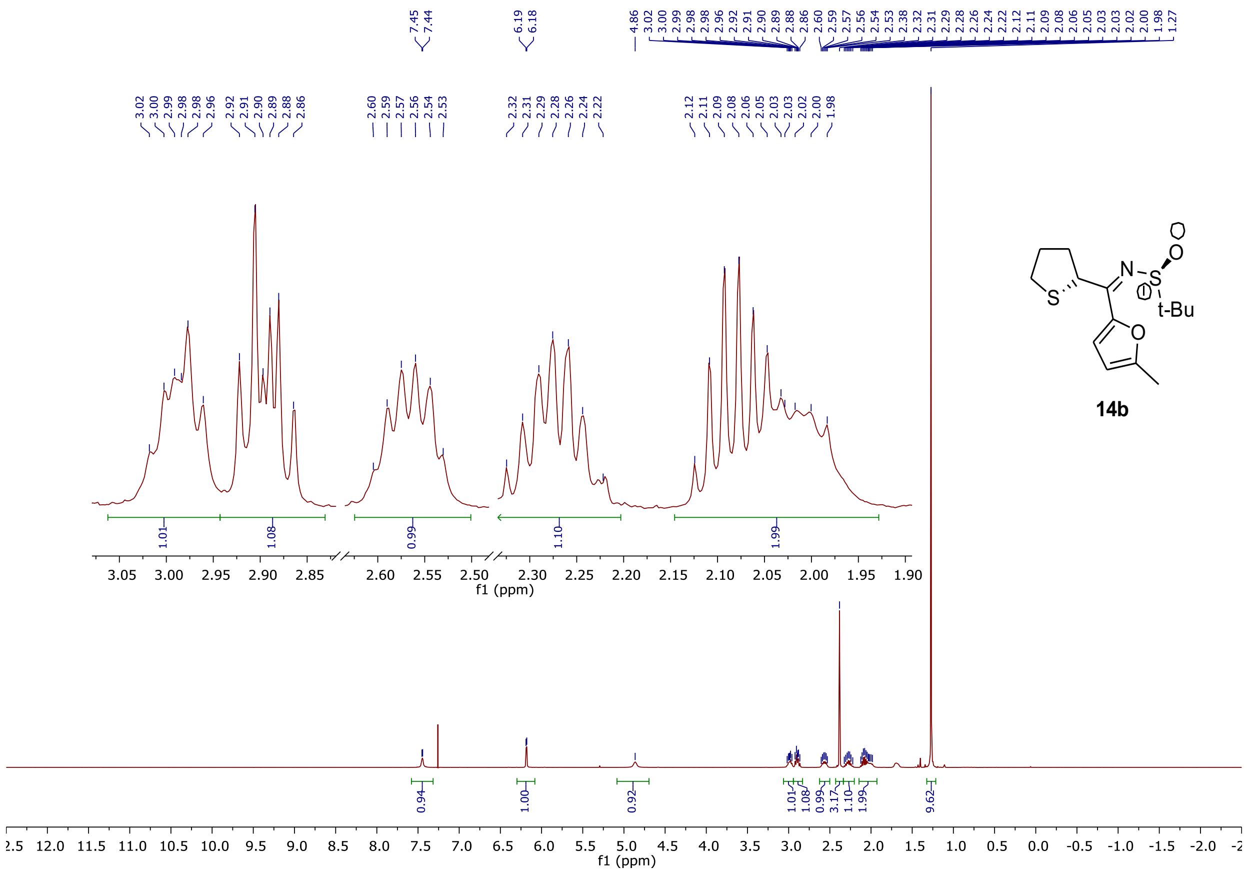

14b

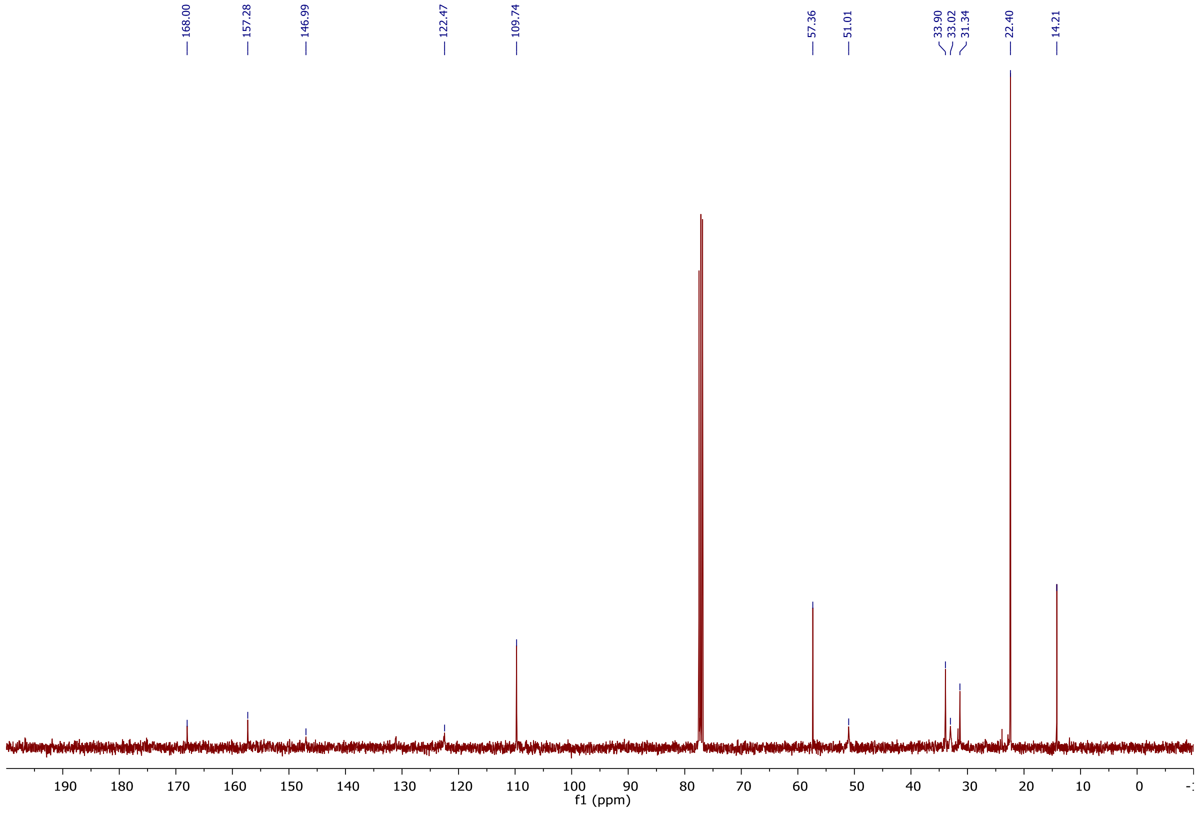

400 MHz ^{1}H NMR spectrum; 100.6 MHz ^{13}C NMR spectrum; $CDCl_3$ of **S6**

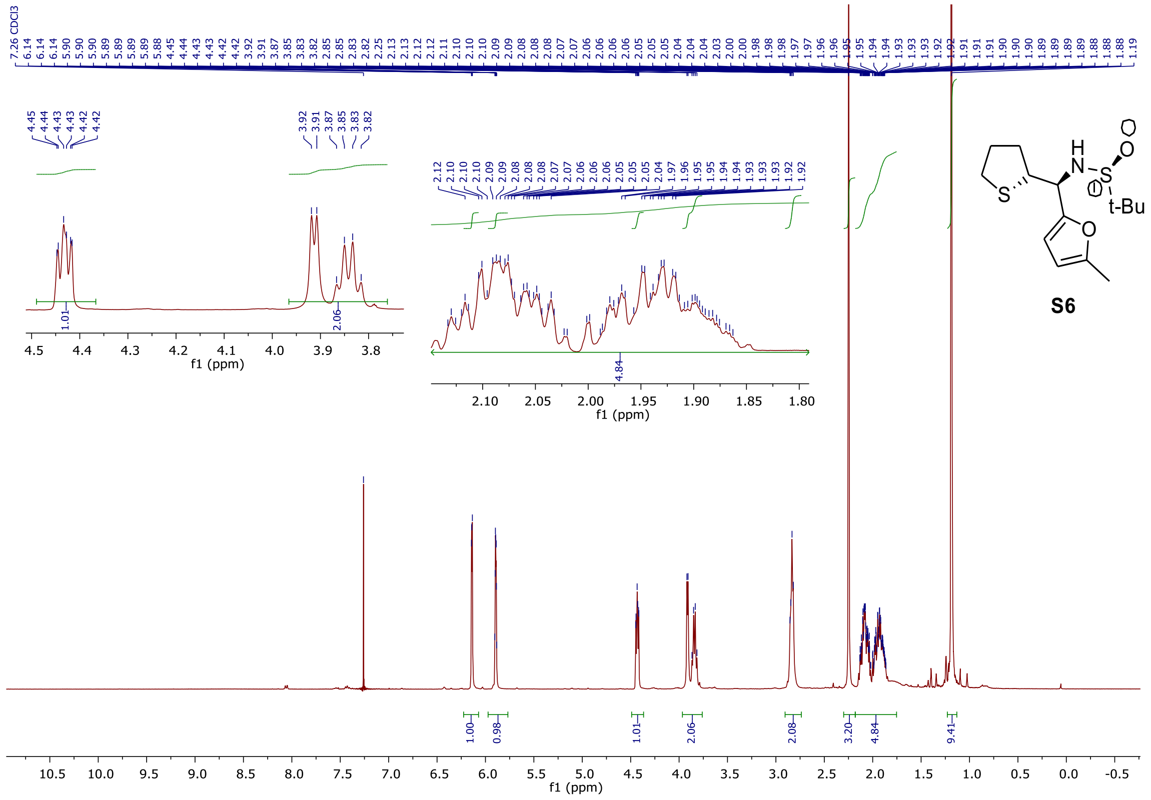

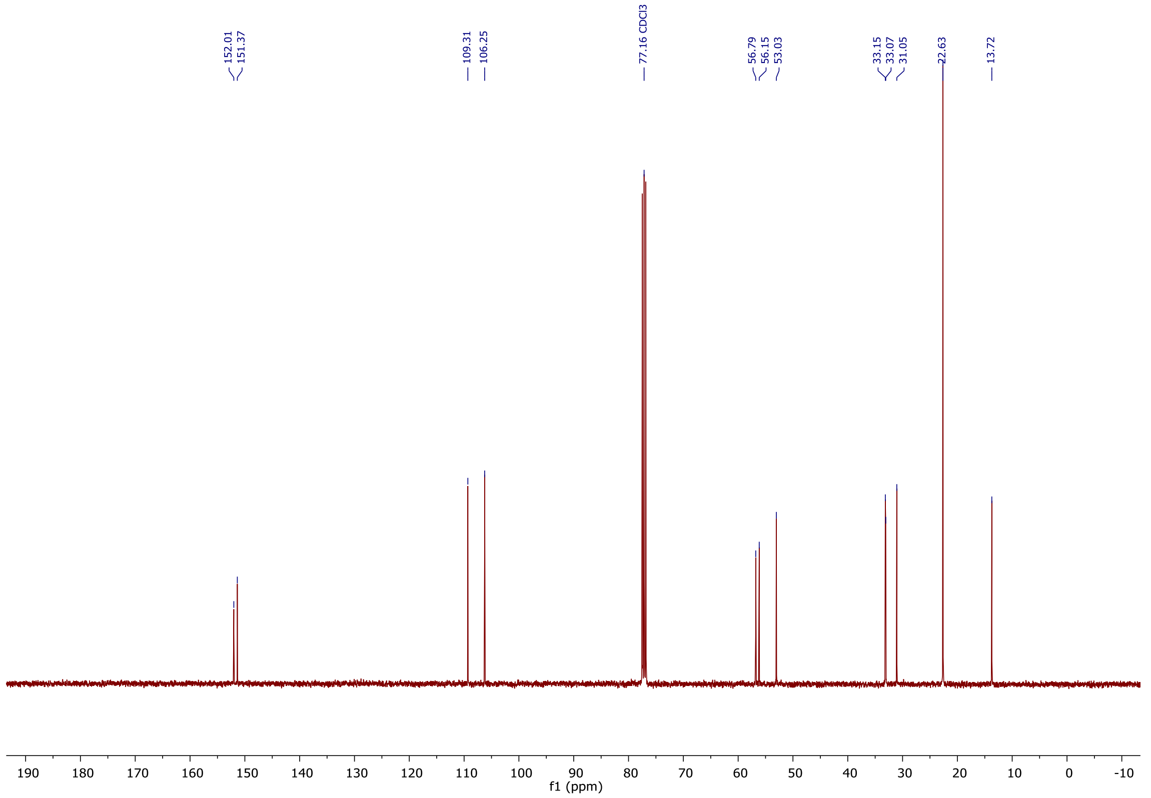

400 MHz ^{1}H NMR spectrum; 100.6 MHz ^{13}C NMR spectrum; $CDCl_3$ of **15•HCl**

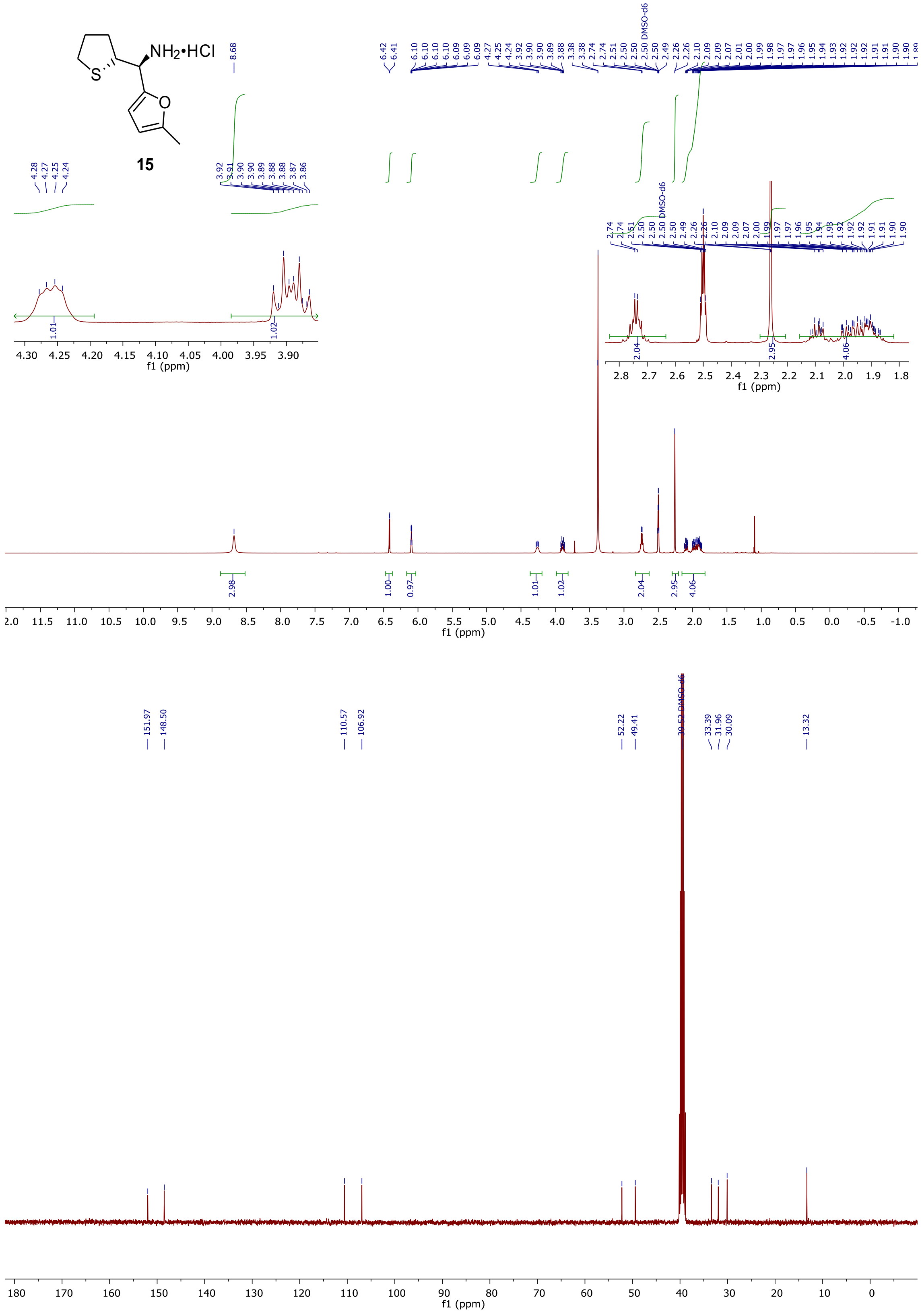

400 MHz ^{1}H NMR spectrum; 100.6 MHz ^{13}C NMR spectrum; $CDCl_3$ of **S7**

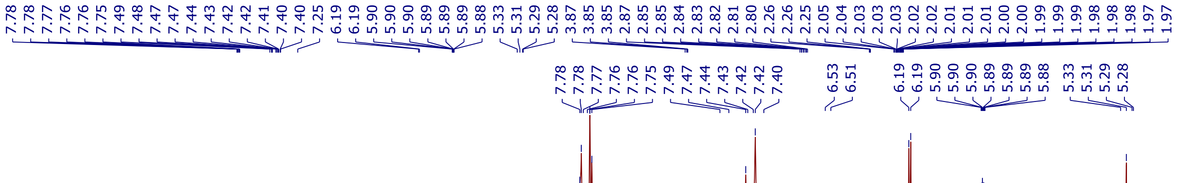

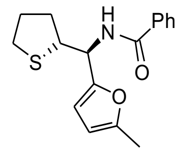

S7

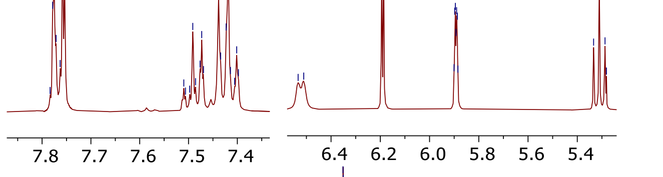

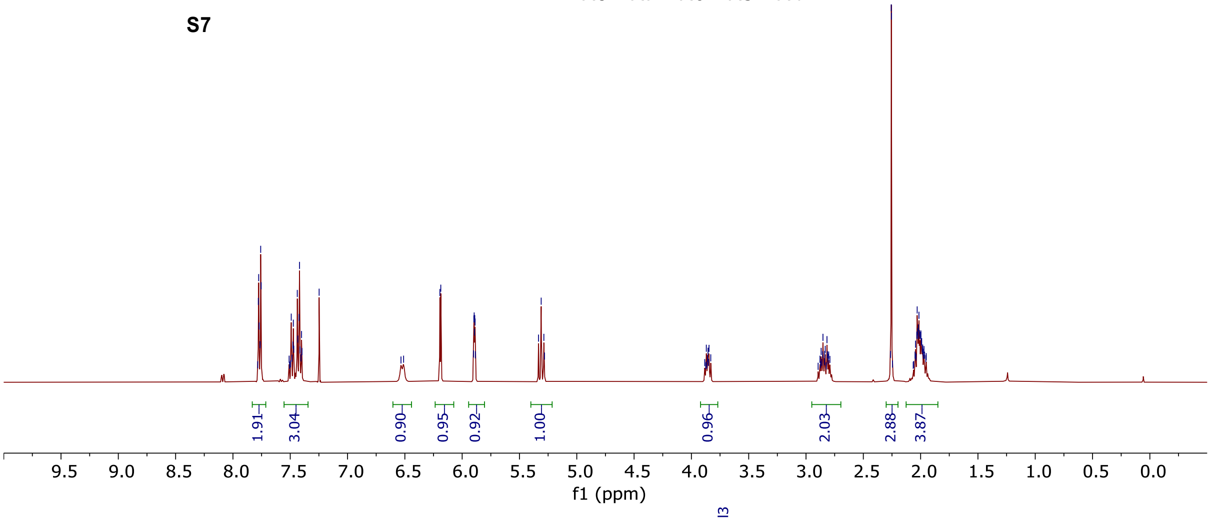

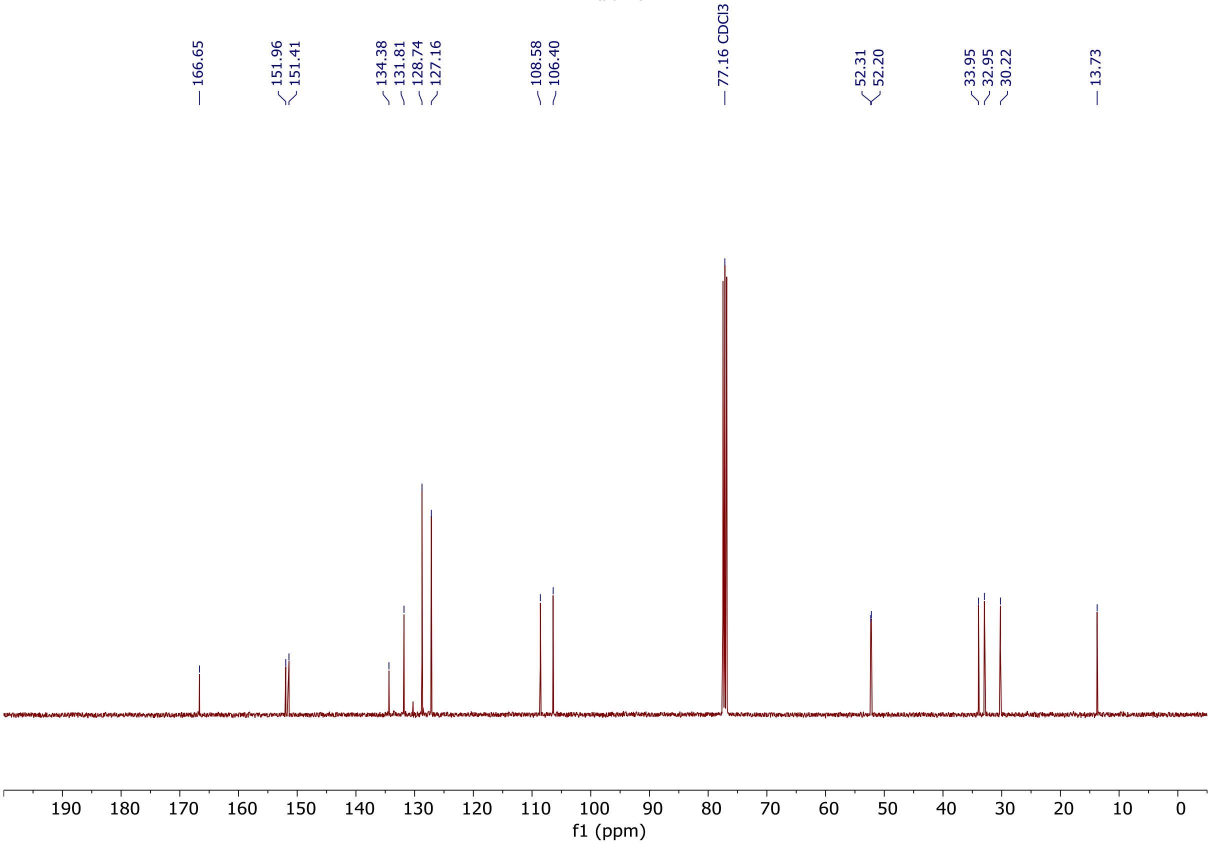

400 MHz ^{1}H NMR spectrum; 100.6 MHz ^{13}C NMR spectrum; $CDCl_3$ of **7q**

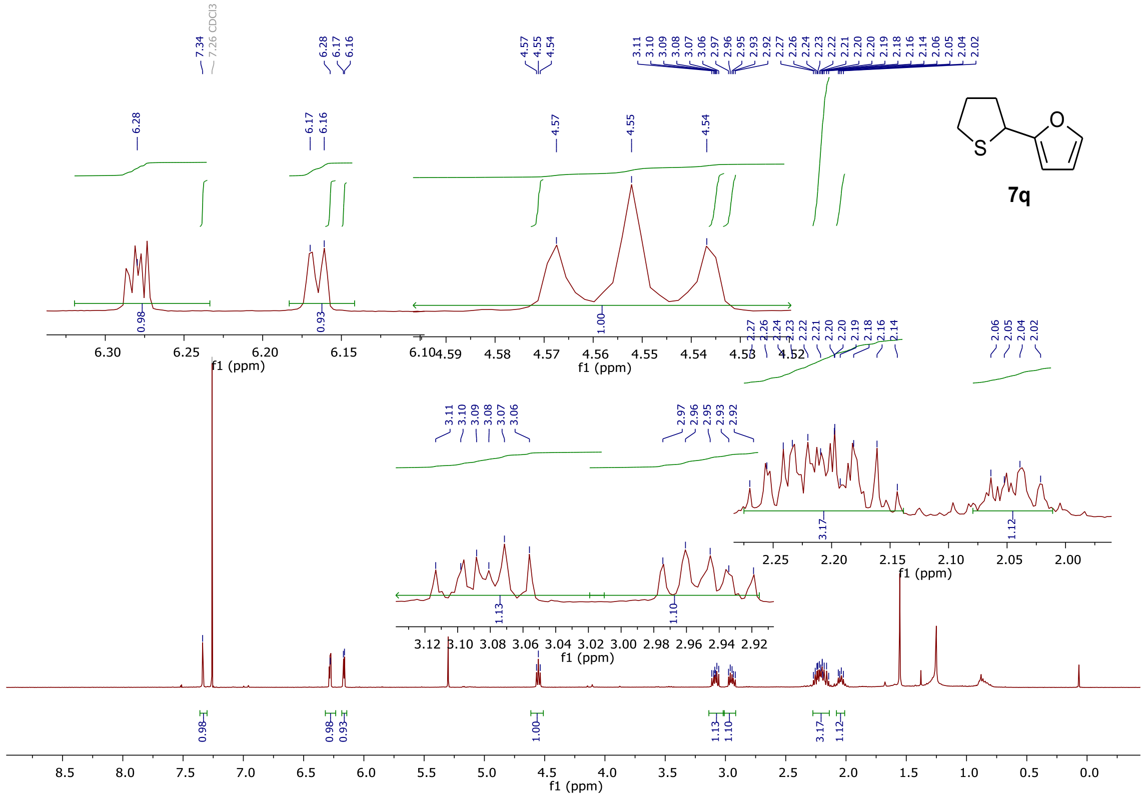

7q

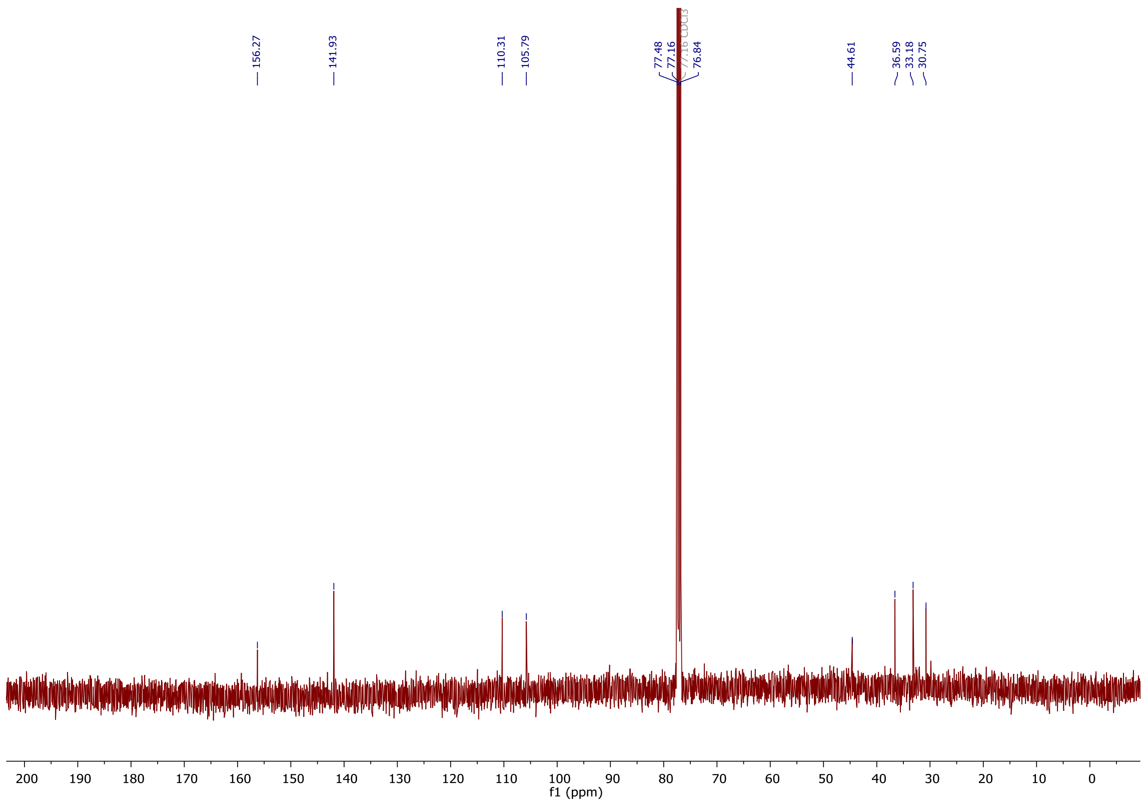

400 MHz ^{1}H NMR spectrum; 100.6 MHz ^{13}C NMR spectrum; $CDCl_3$ of **7r**

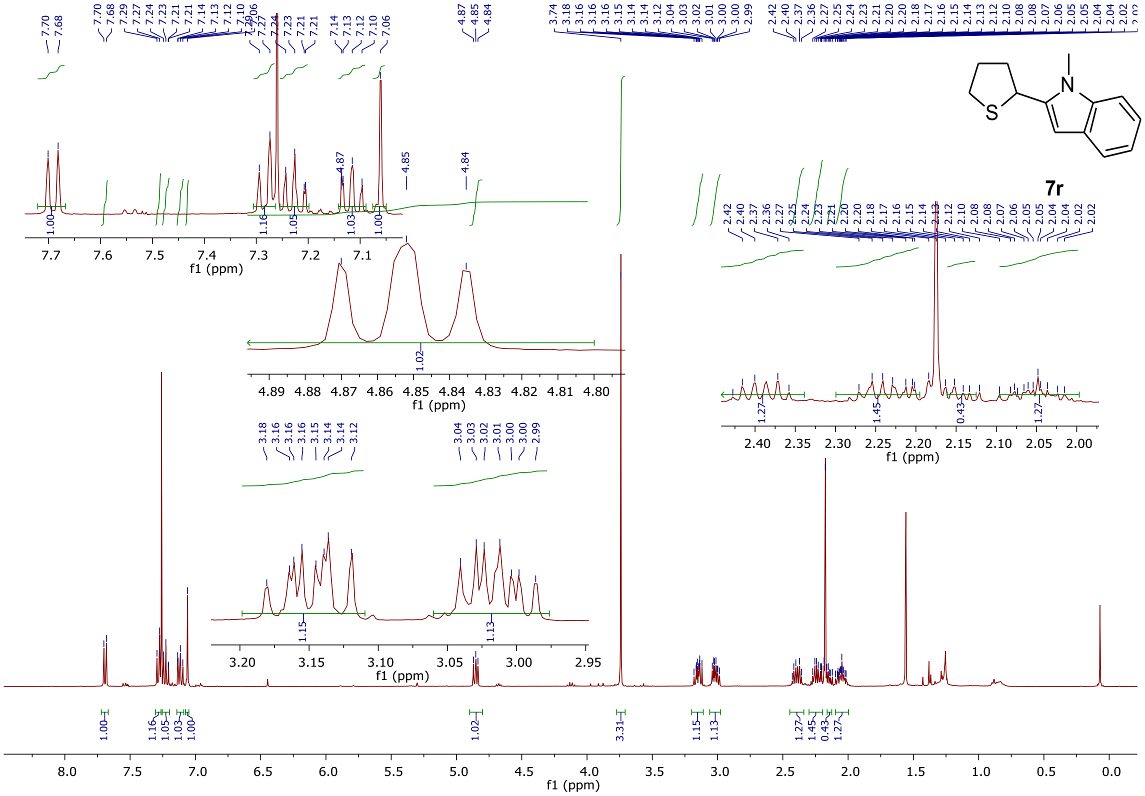

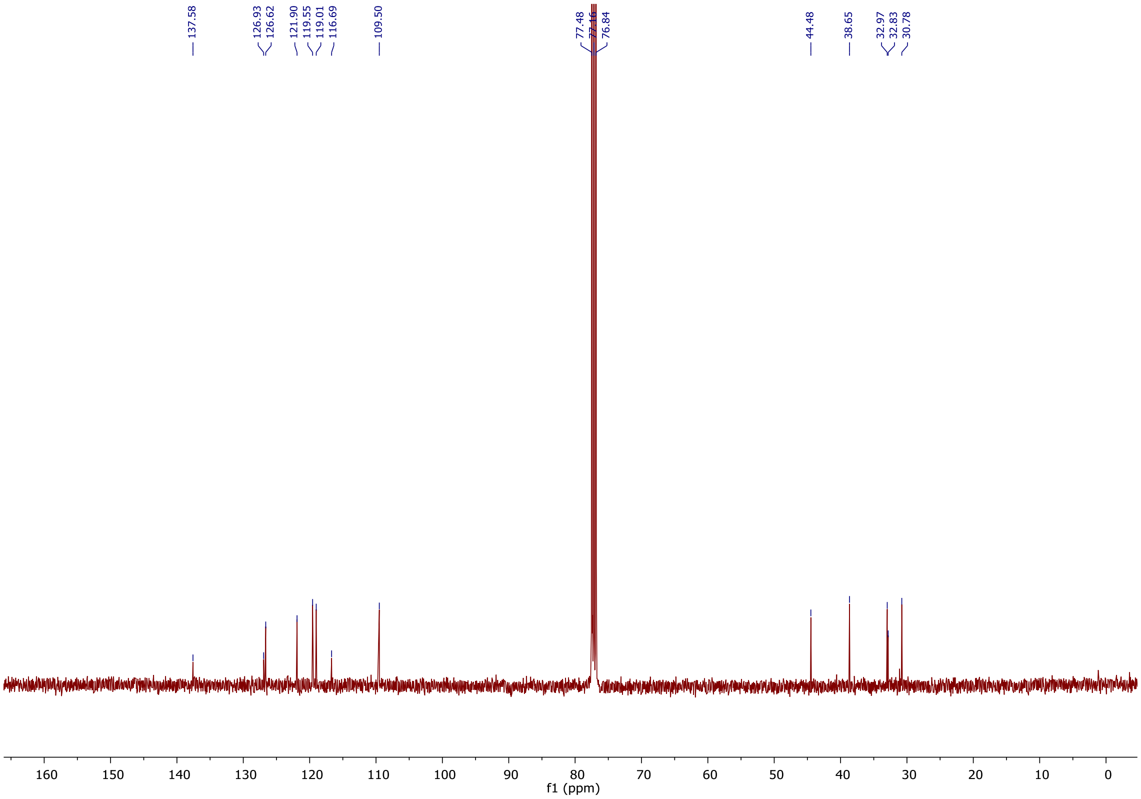

400 MHz ^{1}H NMR spectrum; 100.6 MHz ^{13}C NMR spectrum; $CDCl_3$ of **7s**

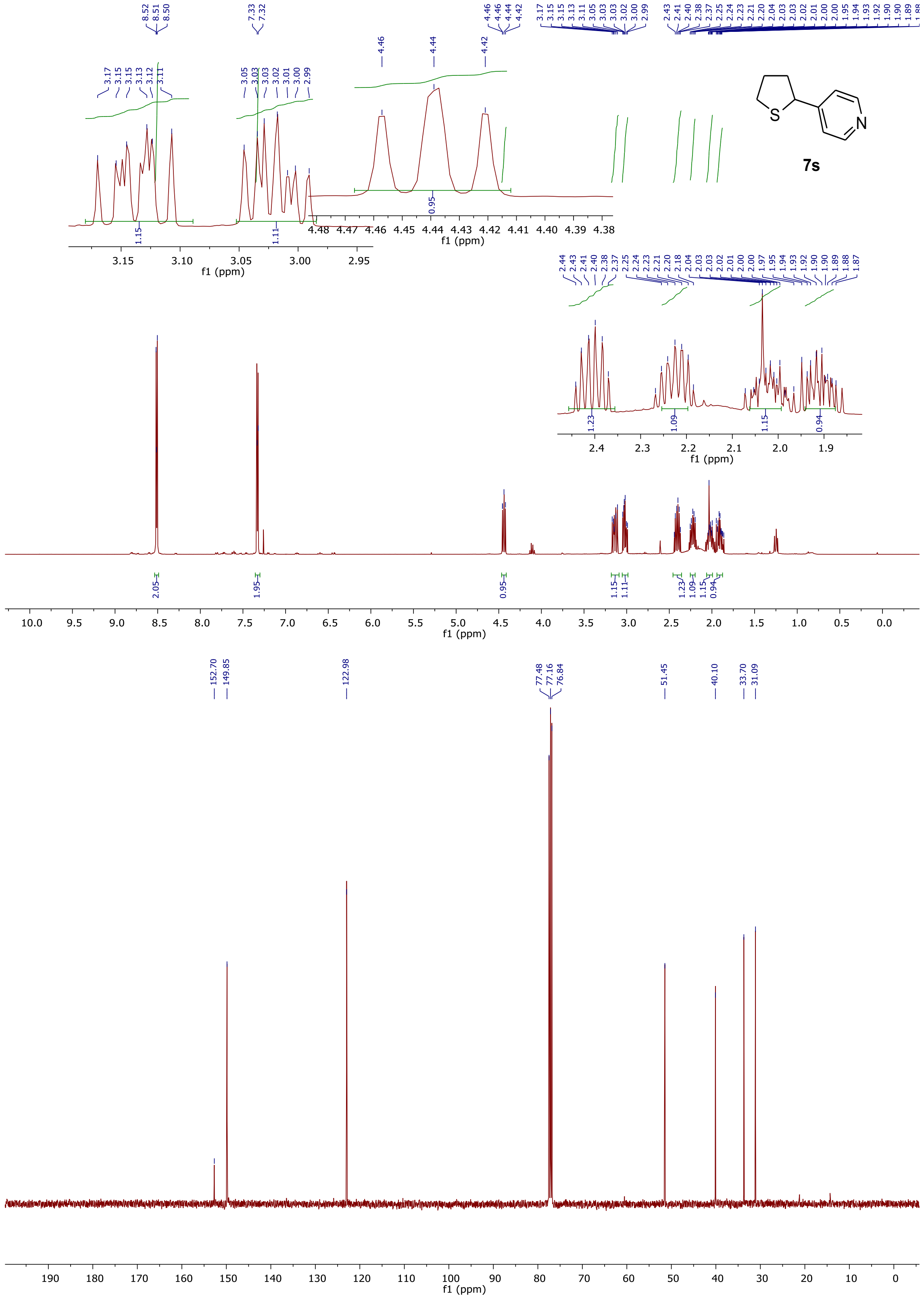

400 MHz ^{1}H NMR spectrum; 100.6 MHz ^{13}C NMR spectrum; $CDCl_3$ of **7t**

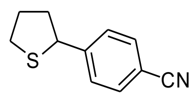

7t

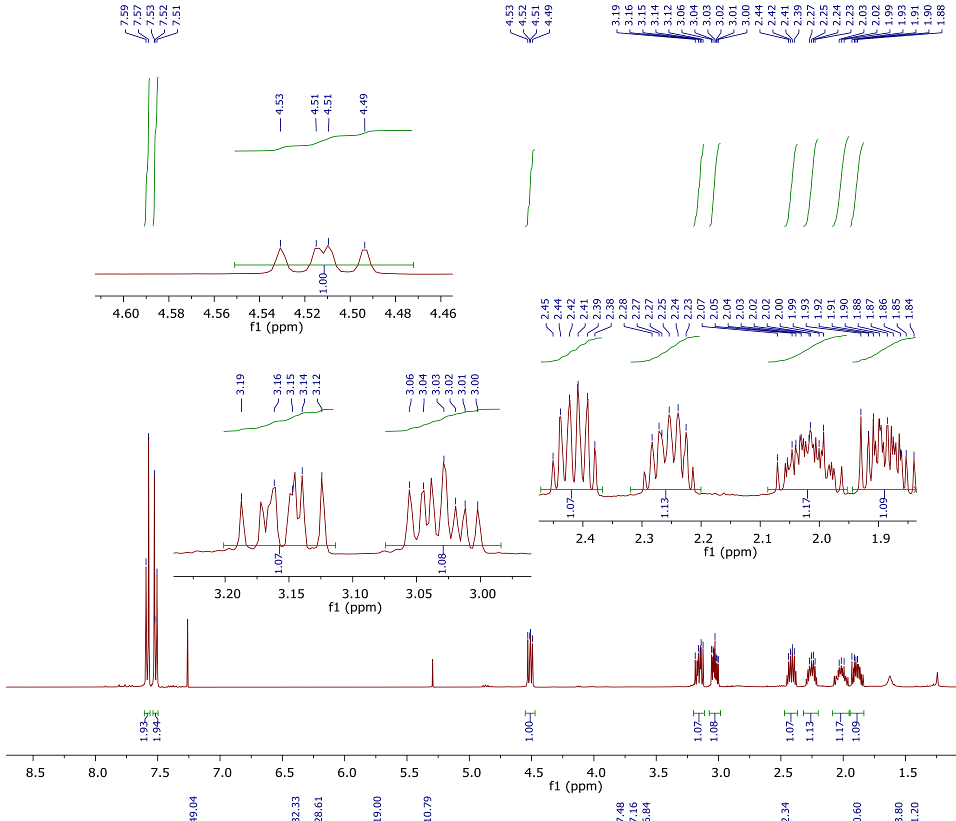

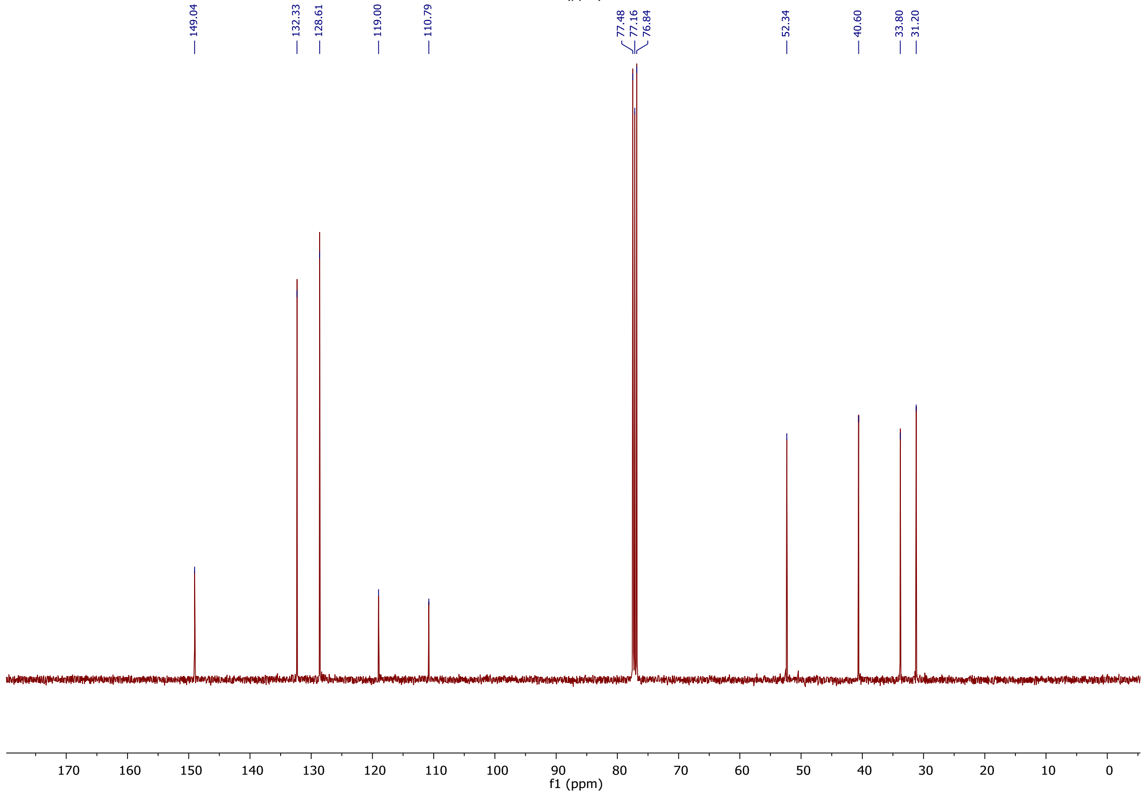

400 MHz ^{1}H NMR spectrum; 100.6 MHz ^{13}C NMR spectrum; $CDCl_3$ of **8m**

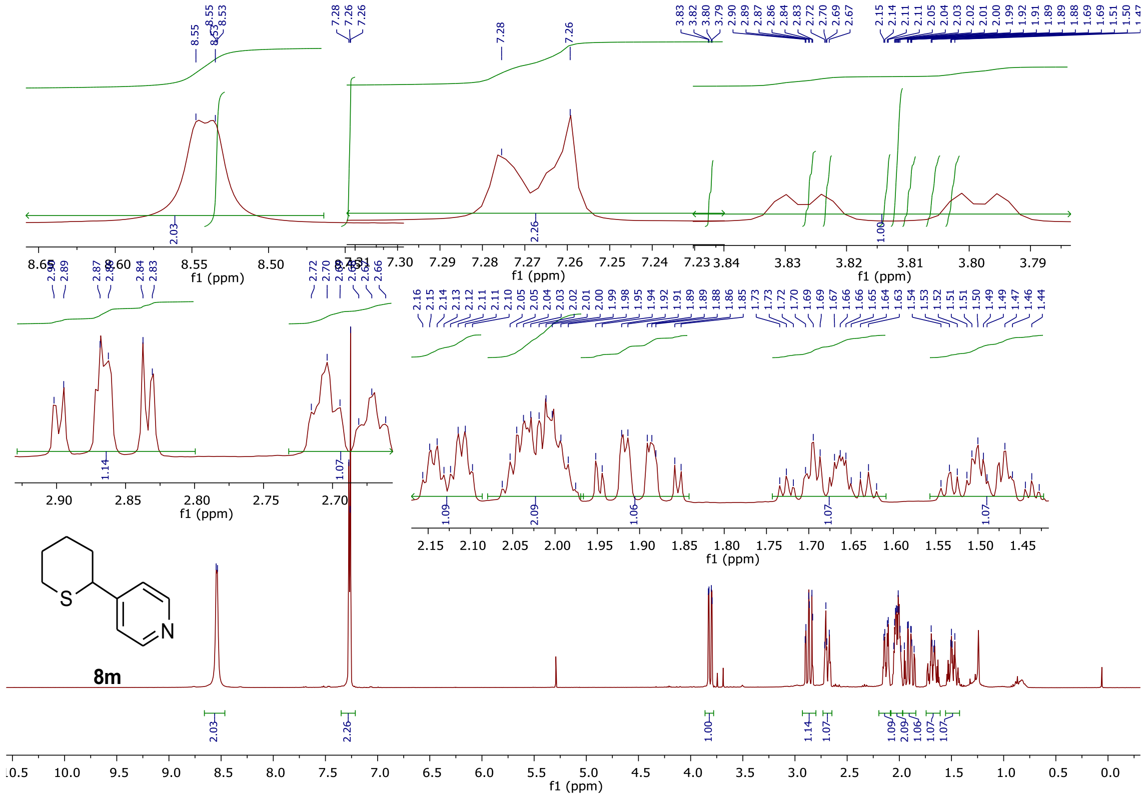

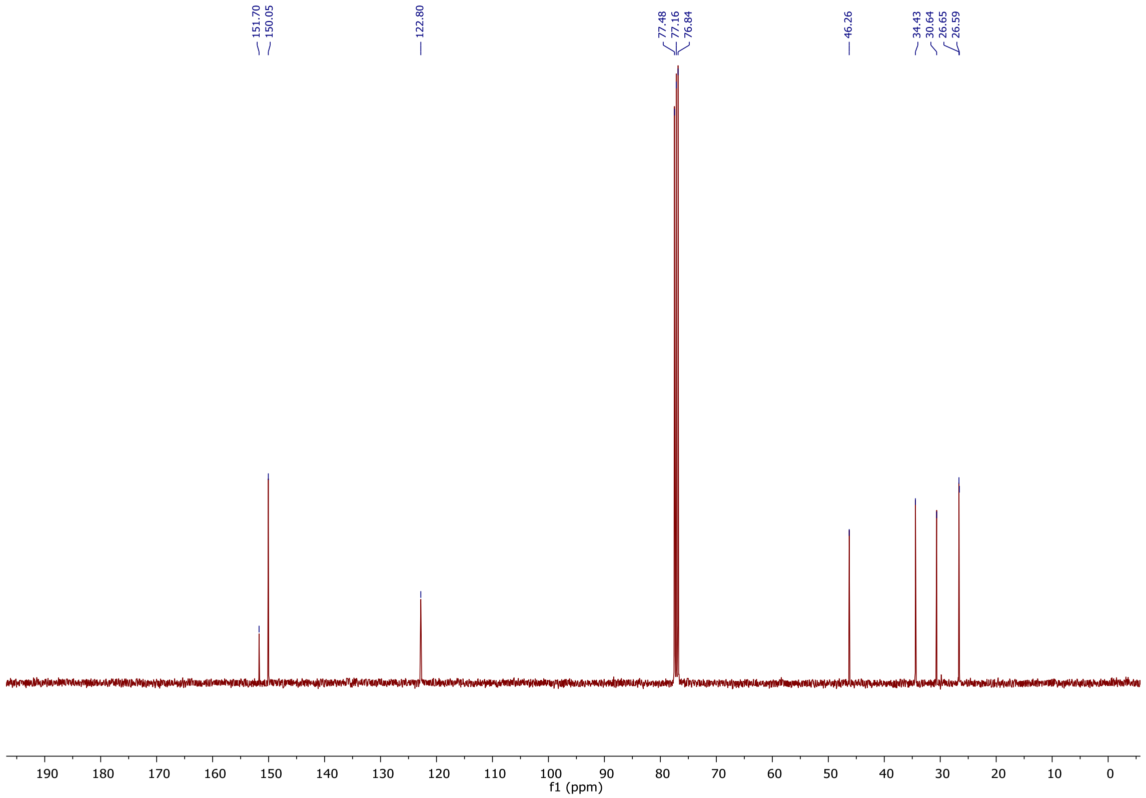

3. CSP-HPLC

CSP-HPLC of *N*-((1*S*)-(5-methyl-furan-2-yl)((*R*)-tetrahydrothiophen-2-yl)methyl)-benzamide (*S*,*R*)-S7 of 97:3 er

Chiralcel AD-H (90:10 hexane-*i*-PrOH, 1.0 mL min^{-1}) 15.1 min (major), 20.4 min (minor).

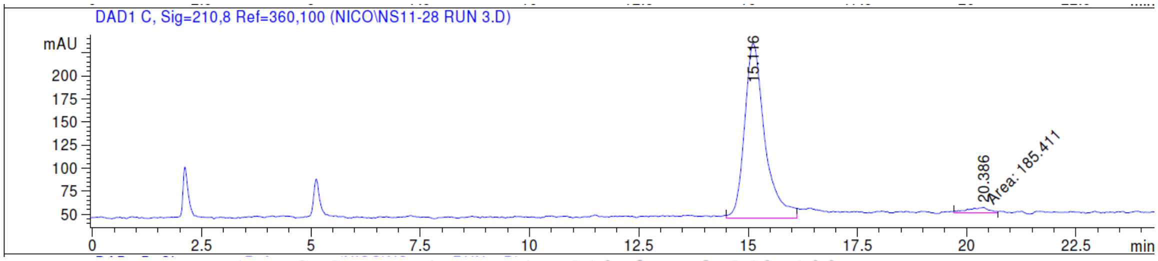

Signal 3: DAD1 C, Sig=210,8 Ref=360,100

Peak #	RetTime [min]	Type	Width [min]	Area [mAU*s]	Height [mAU]	Area %
1	15.116	VB	0.4444	6107.31543	189.29543	97.0536
2	20.386	MM	0.5469	185.41106	5.65004	2.9464
Totals :				6292.72649	194.94546	

Lab Book Reference: NS11-28

CSP-HPLC of racemic *N*-((1*S)-(5-methyl-furan-2-yl)((*R**)-tetrahydrothiophen-2-yl)methyl)-benzamide (*S**,*R**)-S7 of 46:54 er**

Chiralcel AD-H (90:10 hexane-*i*-PrOH, 1.0 mL min^{-1}) 15.3 min (minor), 20.8 min (major).

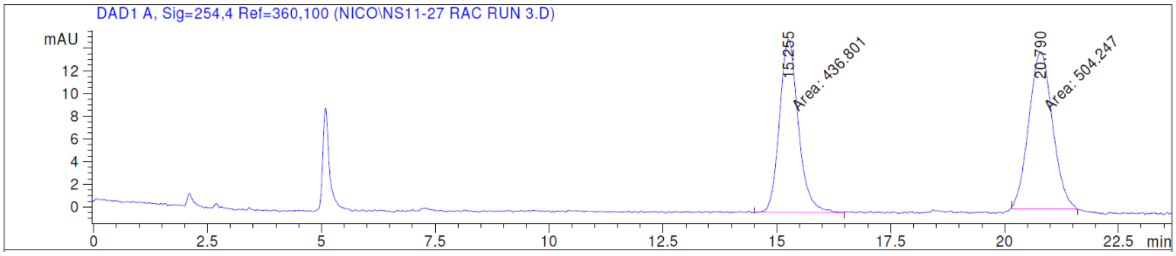

Signal 1: DAD1 A, Sig=254,4 Ref=360,100

Peak #	RetTime [min]	Type	Width [min]	Area [mAU*s]	Height [mAU]	Area %
1	15.255	MM	0.4776	436.80103	15.24334	46.4165
2	20.790	MM	0.6047	504.24680	13.89730	53.5835
Totals :				941.04782	29.14064	

Lab Book Reference: NS11-27

4. References

[1] J. D. Winkler, A. Isaacs, L. Holderbaum, V. Tatard, N. Dahmane, *Org. Lett.* **2009**, *11*, 2824.

[2] S. Voltrova, J. Srogl, *Eur. J. Org. Chem.* **2008**, 1677.

[3] J. T. Wróbel, E. Hejchman, *Synthesis* **1987**, 452.

[4] J. Almena, F. Foubelo, M. Yus, *Tetrahedron* **1995**, *51*, 11883.

[5] J.-J. Filippi, E. Duñach, X. Fernandez, U. J. Meierhenrich, *Tetrahedron* **2008**, *64*, 9999.

[6] Y. Okazaki, F. Ando, J. Koketsu, *Bull. Chem. Soc. Jpn* **2004**, *77*, 1687.

[7] J. Almena, F. Foubelo, M. Yus, *Tetrahedron* **1997**, *53*, 5563.

[8] F. Li, D. Calabrese, M. Brichacek, I. Lin, J. T. Njardarson, *Angew. Chem. Int. Ed.* **2012**, *51*, 1938.

[9] Z. Zhang, B. Górski, D. Leonori, *J. Am. Chem. Soc.* **2022**, *144*, 1986.

[10] R. M. Chabanenko, S. Yu. Mykolenko, E. K. Kozirev, V. A. Palchykov, *Synth. Commun.* **2018**, *48*, 2198.

[11] S. Tabet, N. Rodeville, A. Mathieu, C. Raffin, C. Millois-Barbuis, B. Musicki, F. Muller, T. Gerfaud, J.-G. Boiteau, I. Cardinaud, *Org. Process Res. Dev.* **2017**, *21*, 2032.

[12] F. J. Robertson, J. Wu, *J. Am. Chem. Soc.* **2012**, *134*, 2775.
